# Identification and development of the novel 7-genes diagnostic signature by integrating multi cohorts based on osteoarthritis

**DOI:** 10.1186/s41065-022-00226-z

**Published:** 2022-01-29

**Authors:** Yaguang Han, Jun Wu, Zhenyu Gong, Yiqin Zhou, Haobo Li, Yi Chen, Qirong Qian

**Affiliations:** 1 Department of Joint Surgery and Sports Medicine, Shanghai Changzheng Hospital, Second Military Medical University, Shanghai, 200003 China; 2 Department of Orthopaedic Surgery, Nantong Sixth People’s Hospital, Nantong Hospital Affiliated To Shanghai University, Nantong, Jiangsu Province, China; 3 Department of Emergency Medicine, Zhongshan Hospital, Fudan University, Shanghai, 200032 China

## Abstract

**Background:**

A chronic progressive degenerative joint disease, such as osteoarthritis (OA) is positively related to age. The medical economy is facing a major burden, because of the high disability rate seen in patients with OA. Therefore, to prevent and treat OA, exploring the diagnostic biomarkers of OA will be of great significance.

**Methods:**

Differentially expressed genes (DEGs) were obtained from the Gene Expression Omnibus database using the *RobustRankAggreg* R package, and a protein–protein interaction network was constructed. The module was obtained from Cytoscape, and the four algorithms of degree, MNC, closeness, and MCC in CytoHubba were used to identify the hub genes. A diagnostic model was constructed using Support Vector Machines (SVM), and the ability of the model to predict was evaluated by other cohorts.

**Results:**

From normal and OA samples, 136 DEGs were identified, out of which 45 were downregulated in the normal group and 91 were upregulated in the OA group. These genes were associated with the extracellular matrix-receptor interactions, the PI3K-Akt signaling pathway, and the protein digestion and absorption pathway, as per a functional enrichment analysis. Finally, we identified the 7 hub genes (*COL6A3*, *COL1A2*, *COL1A1*, *MMP2*, *COL3A1*, *POST*, and *FN1*). These genes have important roles and are widely involved in the immune response, apoptosis, inflammation, and bone development. These 7 genes were used to construct a diagnostic model by SVM, and it performed well in different cohorts. Additionally, we verified the methylation expression of these hub genes.

**Conclusions:**

The 7-genes signature can be used for the diagnosis of OA and can provide new ideas in the clinical decision-making for patients with OA.

**Supplementary Information:**

The online version contains supplementary material available at 10.1186/s41065-022-00226-z.

## Introduction

Worldwide, the most common musculoskeletal disease osteoarthritis (OA) is seen in people aged 60 years and above. Recently, because of the increase in life expectancy, the vigorous development of sports, and the increase in the number of people with obesity, the number of young patients with OA is significantly increasing [1]. In the United States, OA has become a major cause of disability, resulting in huge social and economic burdens [2, 3]. A sensitive diagnosis of OA can delay the progression of the disease and can improve the prognoses of patients [4]. Presently, OA is diagnosed mainly by joint aspiration, X-ray, and magnetic resonance imaging (MRI) [5–7]. However, the local cartilage damage cannot be detected by joint aspiration and X-ray before any structural damage occurs [8, 9]. Although MRI is sensitive, it still cannot detect the localized degeneration of the cartilage tissue. Moreover, degeneration of the cartilage tissue is usually related to early pathogenesis of OA [10, 11]. Therefore, there are still certain limitations. A few researchers have proposed that the diagnosis can be done using optical probes based on hyaluronic acid [12], but whether they can be used in clinical practice is not yet confirmed.

xploring the sensitive and specific diagnostic biomarkers of OA is of great significance. In this study, we conducted a comprehensive analysis using multiple public microarray datasets (expression profiles and methylation data) to determine the potential transcriptome biomarkers of OA. Further, we established the gene regulatory networks based on the protein–protein interaction (PPI) network involved in these DEGs to identify the diagnostic biomarkers. Based on the Support Vector Machine (SVM) model, we have identified and established a diagnostic model for patients with OA.

## Methods and materials

### Data sources and downloads

We downloaded the chip expression dataset of OA (GSE129147 [13], GSE57218 [14], GSE51588 [15], and GSE117999 [16]) and the methylation dataset (GSE73626 [17]) from the Gene Expression Omnibus (GEO) database. We selected the chip dataset containing normal samples and OA samples.

### Preprocessing of the data

The GEO datasets were processed as follows: 1) the normal and the OA samples were retained, 2) the probe was converted into a gene symbol, 3) the probe corresponding to multiple genes was removed, and 4) the median of the multiple gene symbol expressions were obtained. In the preprocessed datasets, GSE129147 had 9 normal and 10 OA samples, GSE57218 had 7 normal and 33 OA samples, GSE51588 had 10 normal and 40 OA samples, GSE117999 had 10 normal and 10 OA samples, and GSE73626 had 7 normal and 11 OA samples. The clinical statistics of these samples can be found in Table 1.

**Table 1:** Clinical information

Data set	Expression	Methylation
**GSE129147**		
Normal	9	
OA	10	
**GSE57218**		
Normal	7	
OA	33	
**GSE51588**		
Normal	10	
OA	40	
**GSE117999**		
Normal	10	
OA	10	
**GSE73626**		
Normal		7
OA		11

### Identification of differentially expressed gene and functional analysis

Limma R package was used to calculate the differentially expressed genes (DEGs) between the normal and the OA samples of the GSE129147, GSE57218, and GSE51588 datasets. The DEGs were filtered according to the threshold FDR < 0.05 and |FC|> 1.5. A volcano map was drawn after obtaining the DEGs from the three datasets. The DEGs in the three datasets were analyzed using the RobustRankAggreg [18] R package. According to the score, the heat maps were drawn, and the DEGs were retained. Kyoto Encyclopedia of Genes and Genomes (KEGG) pathway analysis was used to analyze the DEGs in the OA samples, and WebGestaltR (v0.4.2) R package was used to analyze the Gene Ontology (GO) functional enrichment analysis.

### MCODE module screening and functional analysis

To study the interaction network between proteins and help mine core regulatory genes, the Search Tool for the Retrieval of Interacting Genes/Proteins (STRING) database (https://string-db.org/) can be used. The STRING database was used to analyze the PPI network of the DEGs. The resulting files were screened using Cytoscape (v3.7.2), and the network module was obtained using the MCODE plug-in algorithm. The KEGG pathway analysis and the GO functional enrichment analysis were performed on the genes in the MCODE module using the WebGestaltR (v0.4.2) R package.

### Identification of the hub genes

The four algorithms of degree, MNC, closeness, and MCC of the cytoHubba plug-in in Cytoscape (v3.7.2) were used to calculate the PPI network constructed by the DEGs, and the top 10 genes were selected as the hub genes. The hub genes obtained by these four algorithms were intersected with the genes of the functional module MCODE. A Wayne diagram was drawn after the final hub genes were obtained.

### Construction and validation of the diagnostic model

The GSE51588 dataset was used as the training cohort, and the GSE57218, GSE129147, and GSE117999 datasets were used as the validation cohort. The hub genes were used as a feature in the training cohort to obtain the corresponding expression profiles; to build an SVM classification model; to calculate the classification accuracy, sensitivity, and specificity of the model; and the area under the receiver operating characteristic (ROC) curve.

## Results

### Flowchart

**Fig. 1:**
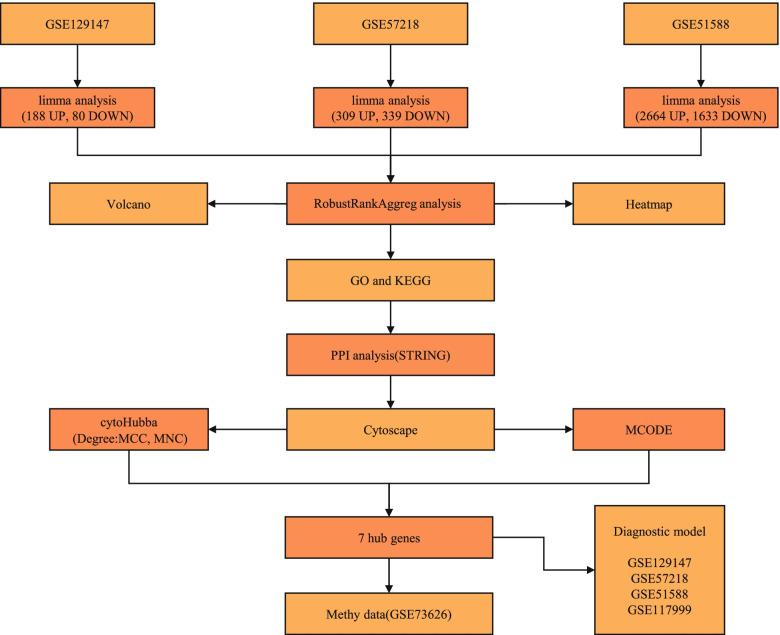
Flowchart displaying the characteristic genes and the diagnostic model of osteoarthritis

To analyze the hub genes and the diagnostic model of OA, we designed a flowchart (Fig. 1).

### Identification of 136 DEGs

The DEGs between the normal and the OA samples of the GSE129147, GSE57218, and GSE51588 datasets were calculated using the limma R package.

**Fig. 2:**
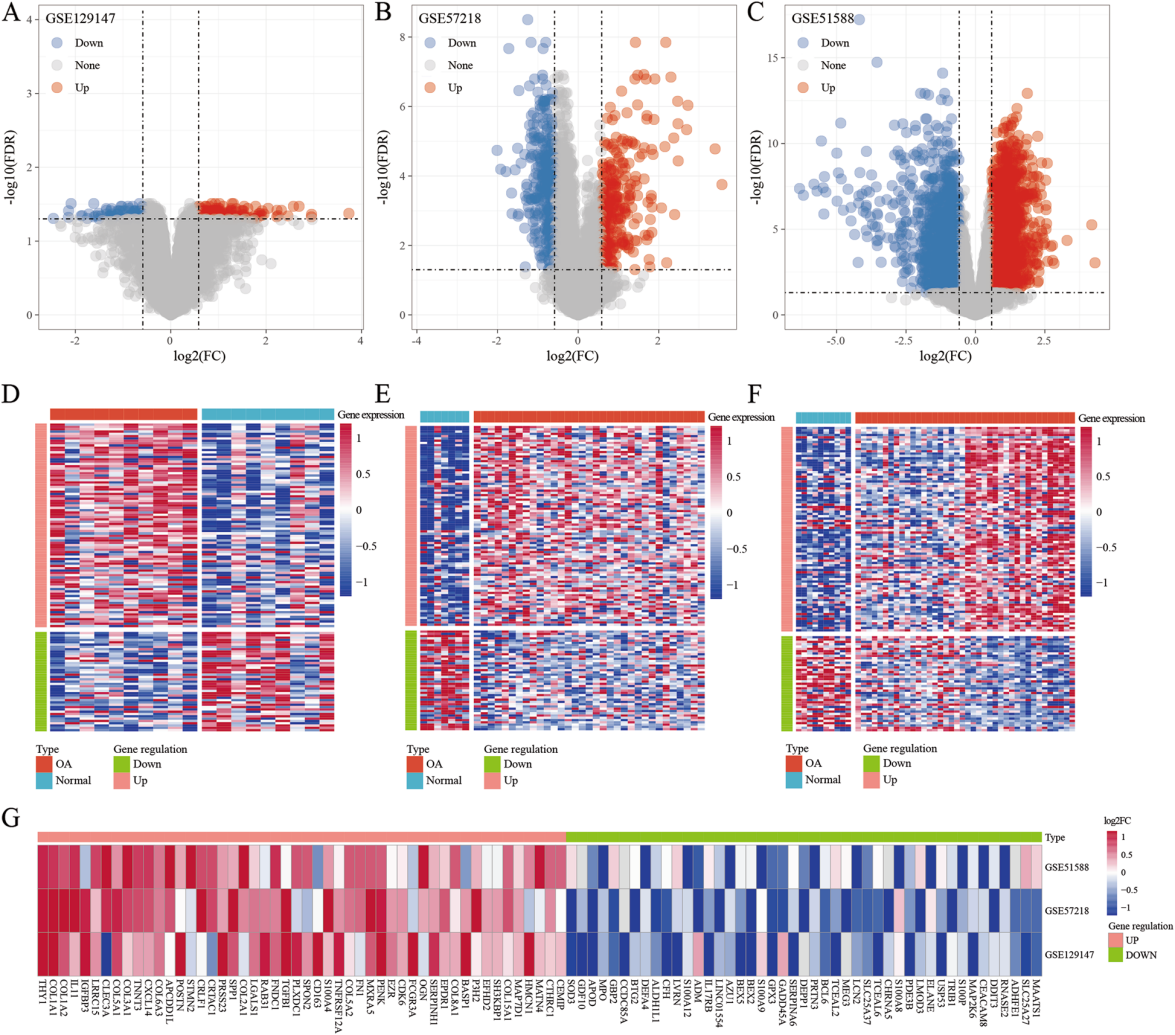
**A**: A volcano map of the differential genes in the GSE129147 dataset. **B**: A volcano map of the differential genes in the GSE57218 dataset. **C**: A volcano map of the differential genes in the GSE51588 dataset. **D**: A heat map of the differential genes in the GSE129147 dataset. **E**: A heat map of the differential genes in the GSE57218 dataset. **F**: A heat map of the differential genes in the GSE51588 dataset. G: A heat map of the partial differential genes in the three datasets

The results showed 268 DEGs in the GSE129147 dataset, out of which 188 were upregulated and 80 were downregulated (S1_Table). There were 648 DEGs in the GSE57218 dataset, out of which 309 were upregulated and 339 were downregulated (S2_Table). There were 4297 DEGs in the GSE51588 dataset, out of which 2664 were upregulated and 1633 were downregulated (S3_Table). The volcano maps of the upregulated and the downregulated DEGs in the normal and the OA samples of the three datasets are shown in Fig. 2A–2C. Further, integration and analysis of the DEGs in the three datasets showed that 136 DEGs were obtained, out of which 91 were upregulated in the OA group (S4_Table) and 45 were downregulated in the normal group (S5_Table). The heat maps of the 136 DEGs are shown in Fig. 2D–2F. The heat map of the top 50 genes that were significantly upregulated and downregulated in each dataset is shown in Fig. 2G.

### Functional analysis of the DEGs

**Fig. 3:**
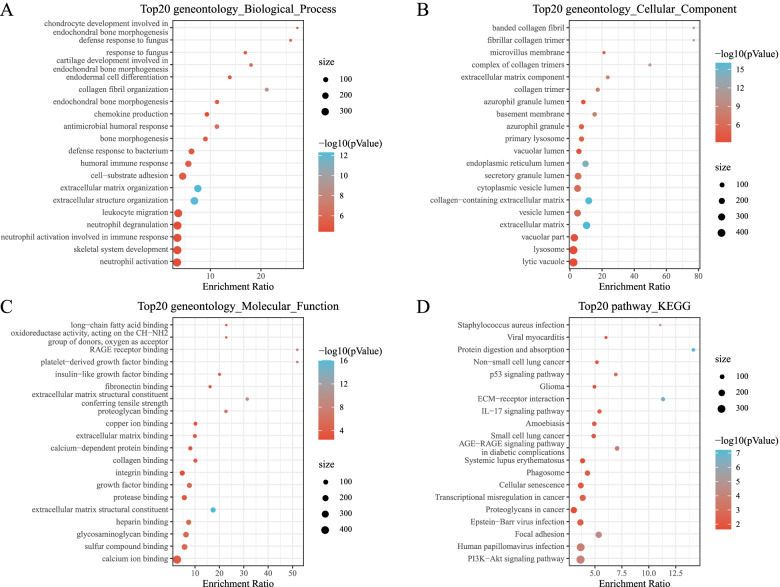
**A**: A biological process annotated map of the differential genes. **B**: A cellular component annotated map of differential genes. **C**: A molecular function annotated map of the differential genes. **D**: A KEGG annotated map of the differential genes

The KEGG pathway analysis and the GO functional enrichment analysis were performed on the 136 DEGs. From the GO annotations of the DEGs in the OA samples, 211 items had significant biological process (BP) differences. The first 20 enriched annotations are shown in Fig. 3A. Humoral immune response, cartilage development involved in endochondral bone morphogenesis, bone morphogenesis, chondrocyte development involved in endochondral bone morphogenesis, endochondral bone morphogenesis, and other BP were enriched. Thirty-six items with significant molecular function (MF) differences were enriched, and the first 20 annotations are shown in Fig. 3C. There were significant differences in the 38 cellular components (CC) annotations (*P* < 0.05). The first 20 are shown in Fig. 3B. For the KEGG pathway enrichment in the OA samples, 36 items were annotated significantly. The first 20 are shown in Fig. 3D. Extracellular matrix (ECM)-receptor interactions, PI3K-Akt signaling pathway, and protein digestion and absorption were significantly enriched.

### Identification and functional analysis of the MCODE module

**Fig. 4:**
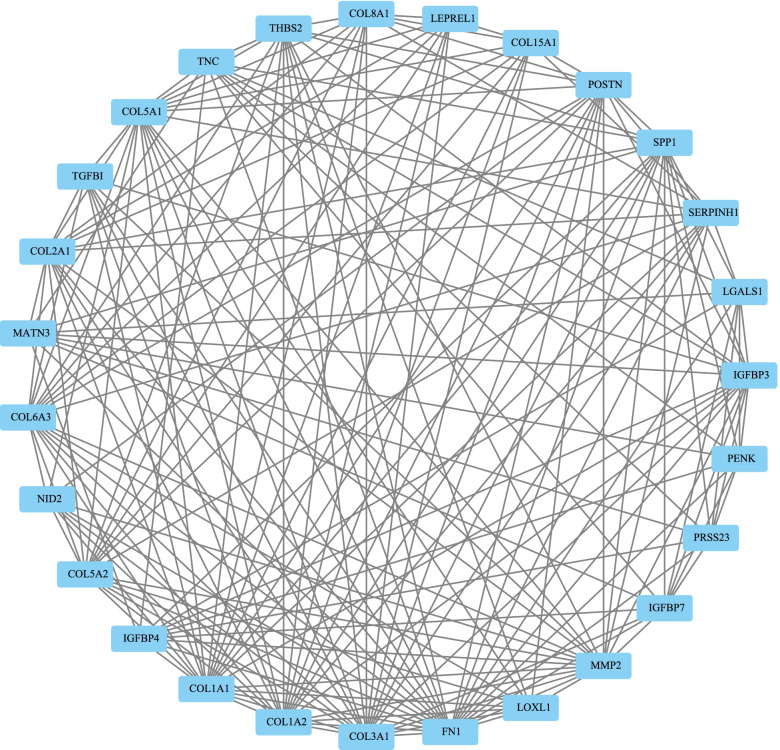
**A** gene protein–protein interaction map of functional modules mined by MCODE

**Fig. 5:**
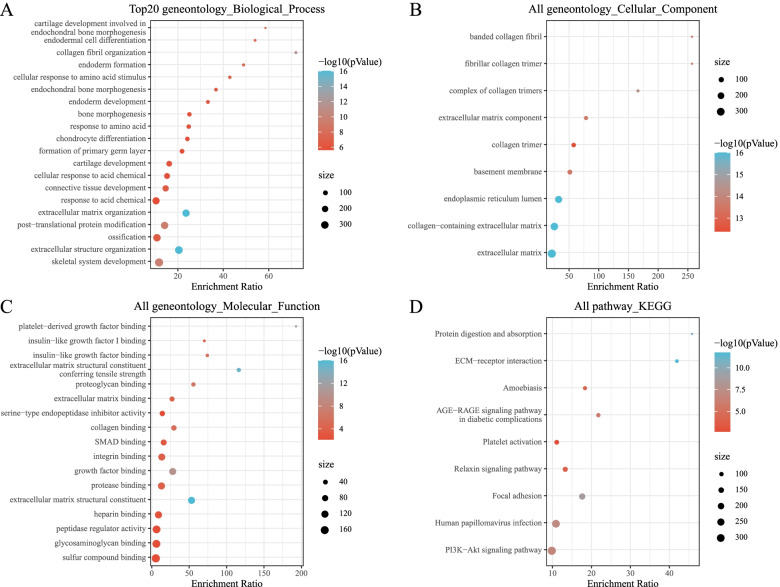
**A**: A biological process annotation map of the MCODE module genes. **B**: A cellular component annotation map of the MCODE module genes. **C**: A molecular function annotation map of the MCODE module genes. **D**: A KEGG annotation map of the MCODE module genes

The 136 DEGs in the PPI network were analyzed using the STRING database and were visualized using Cytoscape (v3.7.2). MCODE1 was found using the MCODE algorithm (Fig. 4). Furthermore, 27 genes in the MCODE1 module were analyzed by the pathway and the functional enrichment analysis. For the GO functional analysis, the first 20 annotations in BP are shown in Fig. 5A. Cartilage development involved in endochondral bone morphogenesis, cartilage development, bone morphogenesis, and bone development were significantly enriched. Seventeen annotations in MF were enriched (Fig. 5C). Nine annotations in CC were enriched (*P* < 0.05) (Fig. 5B). For the KEGG pathway enrichment of the MCODE module, nine significant annotations were enriched (Fig. 5D). The ECM-receptor interactions, the PI3K-Akt signaling pathway, the protein digestion and absorption, and the focal adhesion pathway were significantly enriched.

### Construction and validation of the 7-genes diagnostic model

**Fig. 6:**
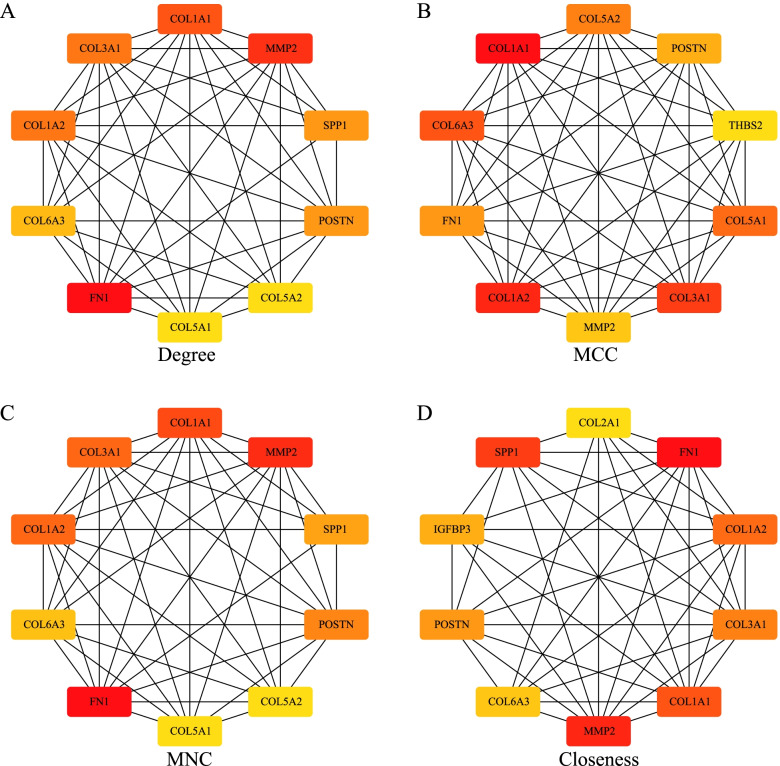
**A**: A protein–protein interaction (PPI) network diagram of the hub genes using the degree algorithm. **B**: A PPI network diagram of the hub genes using the MCC algorithm. **C**: A PPI network diagram of the hub genes using the MNC algorithm. **D**: A PPI network diagram of the hub genes using the closeness algorithm

**Fig. 7:**
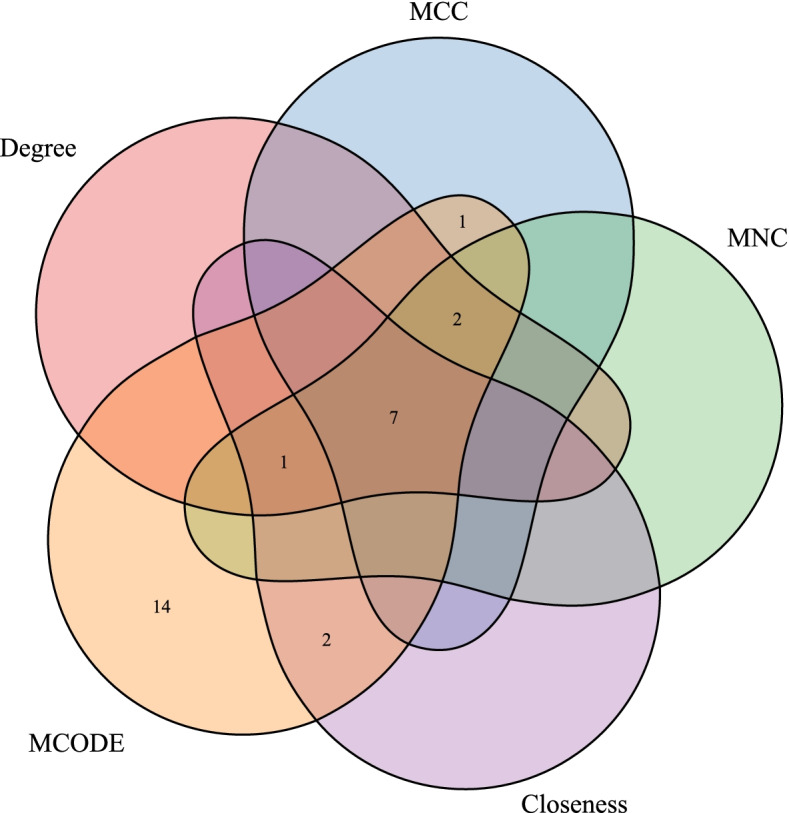
Wayne diagram displaying the hub genes

**Fig. 8:**
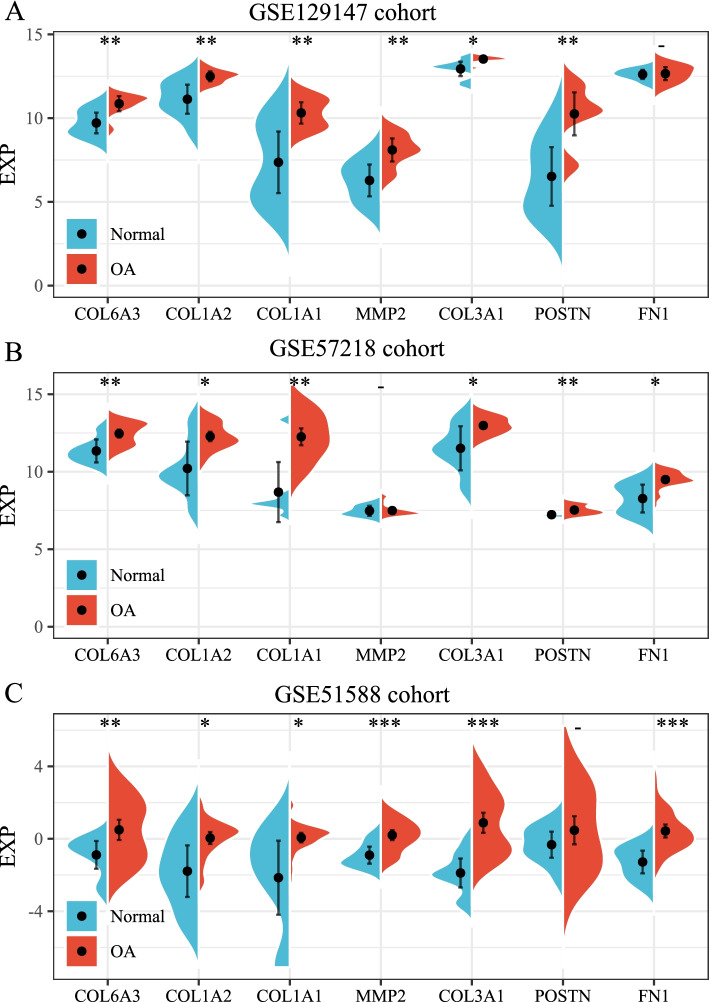
**A**: Expression of the hub genes in the GSE129147 dataset. **B**: Expression of the hub genes in the GSE57218 dataset. **C**: Expression of the hub genes in the GSE51588 dataset

We used the four algorithms of degree, MNC, closeness, and MCC of cytoHubba to calculate the 136 DEGs in the PPI network, and then selected the top 10 genes as the hub genes. The PPI network of these hub genes is shown in Fig. 6. The hub genes obtained by these four algorithms were intersected with the genes in the MCODE module, and thus, the 7 hub genes, including *COL6A3*, *COL1A2*, *COL1A1*, *MMP2*, *COL3A1*, *POSTN*, and *FN1* were obtained (Fig. 7). Additionally, we compared the expression of these 7 hub genes in the normal and the OA samples with other datasets (GSE129147, GSE57218, and GSE51588), and the results showed that all the 7 genes were highly expressed in the OA samples (Fig. 8).

**Fig. 9:**
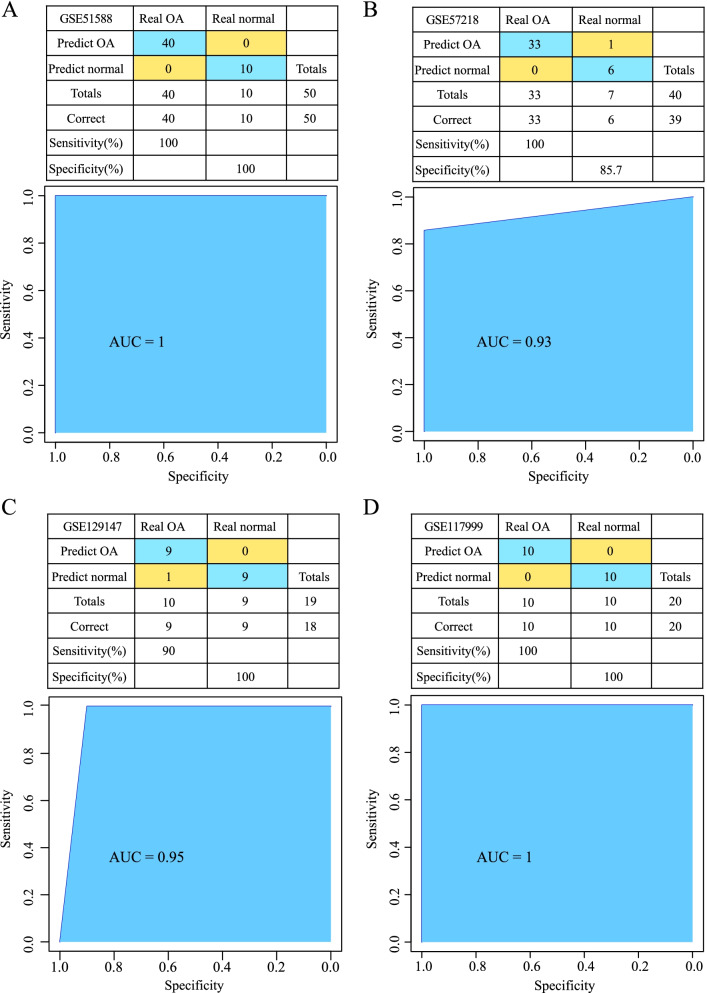
**A**: Classification result and receiver operating characteristic (ROC) curve of the diagnostic model in the GSE51588 dataset. **B**: Classification result and ROC curve of the diagnostic model in the GSE57218 dataset. **C**: Classification result and ROC curve of the diagnostic model in the GSE129147 dataset. **D**: Classification result and ROC curve of the diagnostic model in the GSE117999 dataset

The SVM classification model was constructed using the 7 hub genes as a feature in the training cohort. A 100% classification accuracy rate was obtained. Of the 50 samples, all 50 were correctly classified, the sensitivity and specificity of the model were both 100%, and the area under the ROC curve (AUC) was 1 (Fig. 9A). The GSE57218 dataset was used for verification. Thirty-nine of the 40 samples were correctly classified; the classification accuracy rate was 97.5%, the sensitivity of the model was 100%, the specificity was 85.7%, and the AUC was 0.93 (Fig. 9B). The GSE129147 dataset was used for verification. Eighteen of the 19 samples were correctly classified; the classification accuracy rate was 94.7%, the sensitivity of the model was 90%, the specificity was 100%, and the AUC was 0.95 (Fig. 9C). Finally, the GSE117999 dataset was used for verification. Twenty of the 20 samples were correctly classified; the classification accuracy rate was 100%, the sensitivity of the model was 100%, the specificity was 100%, and the AUC was 1 (Fig. 9D). These results show that the diagnostic prediction model constructed in this study could effectively distinguish between the normal and the OA samples, and the 7 hub genes could be used as reliable biomarkers for diagnosing OA.

### Methylation analysis of the hub genes

**Fig. 10:**
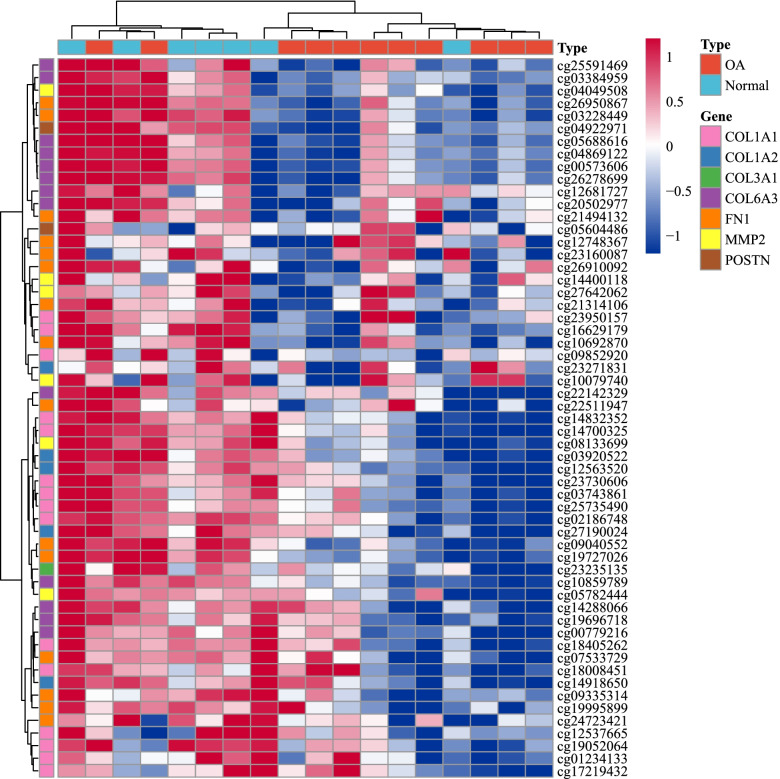
Methylation sites of a few of the hub genes undergoing methylation

The GSE73626 dataset was used to analyze the methylation of the 7 hub genes in the normal and the OA samples. Information on the annotation of the methylation sites and the distance between the probe CpG site and the transcription start site is compiled in S6_Table.The results showed that the methylation sites of *COL6A3*, *COL1A2*, *COL1A1*, *MMP2*, *COL3A1*, *POSTN*, and *FN1* were methylated in the normal samples. The normal samples had higher overall methylation than the OA samples, which is consistent with the fact that the expression of these 7 genes in the normal samples was lower than that in the OA samples. Fig. 10Methylation was negatively correlated with gene expression.

## Discussion

OA is a chronic progressive degenerative joint disease having an incidence positively correlated with age. Joint pain, swelling, and limited mobility are the main clinical manifestations of OA. The current treatments are mainly aimed at alleviating joint pain and reducing joint disability, which has become a major burden on the medical economy. In 1986, Poulter et al. proposed that immunocytological examination of the synovial fluid might have a diagnostic and prognostic value for OA [19]. However, so far, the pathogenesis of OA is still unclear. Investigating the pathogenesis of OA in-depth and exploring the biomarkers for diagnosing OA have important health and economic benefits.

In this study, we identified DEGs between the normal and the OA samples in three datasets (GSE129147, GSE57218, and GSE51588) using the RobustRankAggreg R package, and 136 DEGs were identified. A PPI network was constructed based on these DEGs, and MCODE1 was selected. The genes in this module were mainly involved in endochondral bone morphogenesis, cartilage development, and bone morphogenesis.

Endochondral bone morphogenesis results from chondrocyte differentiation, hypertrophy, death, and bone replacement. This process is extremely important for normal bone growth and development and fracture repair. Endochondral bone formation can be promoted by inhibiting apoptosis signal-regulated kinase 1 and by increasing the survival rate of chondrocytes, thereby slowing the progression of OA [20]. Pathway analysis showed that the ECM-receptor interactions, the PI3K-Akt signaling pathway, the protein digestion and absorption, and the focal adhesion pathway were significantly enriched.

Studies have shown that the above three signaling pathways play an important role in the occurrence and development of OA. Among them, ASG-IV might regulate the Hippo signaling pathway by upregulating VTN and collagen type I alpha 1 (COL1A1) involved in the ECM-receptor interactions, thus playing an important role in chondrocyte apoptosis in human OA [21]. Fibroblast growth factor 18 exerts an anti-OA effect by fusing and dividing the PI3K-Akt signaling pathway and the mitochondria [22]. Downregulation of microRNA 34a (MiR-34a) promotes chondrocyte proliferation and inhibits apoptosis by activating the PI3K-Akt signaling pathway in chondrocytes in rat OA [23]. Resveratrol inhibits the development of obesity-related OA through toll-like receptor 4 (TLR4) and the PI3K-Akt signaling pathway [24]. OA can be reduced in mice by deleting the focal adhesion mechanosensitive connector, hydrogen peroxide-inducible clone-5 (Hic-5) [25].

We used the four algorithms of Degree, MNC, Closeness, and MCC of the CytoHubba to calculate the 136 DEGs in the PPI network, and then selected the top 10 genes as the hub genes. The hub genes obtained using these four algorithms were intersected with the genes in MCODE1, and thus the 7 hub genes, including *COL6A3*, *COL1A2*, *COL1A1*, *MMP2*, *COL3A1*, *POSTN*, and *FN1*, were obtained. These genes are widely involved in immune response, apoptosis, inflammation, and bone development. Collagen type VI-related myopathies are inherited myopathies characterized by muscle weakness, atrophy, and joint contracture as the main clinical manifestations, because of the functional defects in the type VI collagen [26]. The α1, α2, and α3 peptide chains are composed of heterologous peptide chain monomers through triple-helix structures. The active type VI collagen is formed when the monomers form tetramers. These three peptide chains are encoded by the *COL6A1, COL6A2,* and *COL6A3* genes, respectively [26, 27]. Collagen-related myopathies occur when any of these genes have pathogenic variants. COL1A1 is closely related to Caffey disease and type I osteogenesis imperfecta, and its related pathways include the integrin pathway and collagen chain trimerization [28–32]. Collagen type III alpha 1 (COL3A1) exists in the skin, lungs, intestinal walls, and blood vessel walls, and it can strengthen and support many tissues in the body [33–35]. Multicentric osteolysis, nodulosis, and arthropathy are caused by at least eight mutations in the matrix metalloproteinase-2 (MMP2) gene, which is a rare inherited bone disease characterized by loss of bone tissue (osteolysis), especially in the hands and feet. Each known *MMP2* gene mutation eliminates the function of the *MMP2* and prevents the normal cutting of type IV collagen. During bone modeling, losing enzyme activity might disrupt the balance between new bone formation and rupture of existing bones, leading to the gradual loss of bone tissue [36–38]. Periostin (POSTN) can promote accumulation and differentiation of the osteoblasts and their precursor cells in the periosteum [39], the damaged blood vessel reconstruction [40], and the proliferation and fibrosis of inflamed tissues [41]. In vivo and in vitro experiments have shown that adding a certain concentration of POSTN can increase the proliferation rate of knee tibial plateau chondrocytes. Intra-articular injection of a certain amount of PSTN may lead to new ideas to treat knee OA in the middle and the late stages. [42]. In terms of synovial chondropathy cases, 57% show Fibronectin-1 (FN1) and/or activin receptor type-2A [43]. The *FN1*-*EGF* gene fusion is common in calcified aponeurotic fibroma [44]. Spinal hypoplasia, some corner fractures, and glomerulopathy with fibronectin deposits are diseases associated with FN1 [45, 46]. Combined with the existing research, it can be speculated that the above 7 genes play an important role in the occurrence and development of OA, but their specific mechanisms still need to be explored.

We used these seven genes to construct a diagnostic model using the SVM method. The model displayed good performance in different datasets. Additionally, we verified the expression of a few hub genes, and the methylation was negatively correlated with gene expression, showing that to a certain extent, the expression of these genes is related to methylation regulation.

There are a few limitations to our study. The sample size in this study was limited, and the cohort was not large enough, which might have affected the statistical validity and accuracy of our results. Moreover, this study is based only on bioinformatic analysis. Therefore, complementary and basic experiments are still required to reveal the specific mechanism of action of the signature gene markers in the progression of OA. Further studies will be necessary to explore the underlying molecular mechanism of action of these genes to demonstrate their applicability in clinical applications.

In conclusion, we have systematically analyzed the expression of multiple GEO cohorts and found the expression characteristics of the 7 hub genes, according to the PPI network. These genes are associated with the occurrence and development of OA and have a high accuracy in predicting OA (AUC > 0.93), which provides a theoretical basis for diagnosing OA by clinicians . 

## Supplementary Information

**Additional file 1.** Tableshowing the 268 differentially expressed genes in the GSE129147 dataset, ofwhich 188 were upregulated and 80 were downregulated

**Additional file 2.** Tableshowing the 648 differentially expressed genes in the GSE57218 dataset, ofwhich 309 were upregulated and 339 were downregulated

**Additional file 3.** Tableshowing the 4297 differentially expressed genes in the GSE51588 dataset, ofwhich 2664 were upregulated and 1633 were downregulated

**Additional file 4.** Tableshowing the integration and analysis of the differentially expressed genes inthe three datasets, 136 differentially expressed genes were obtained, of which91 were upregulated in the OA group

**Additional file 5.** Tableshowing that in the three datasets, 136 differentially expressed genes wereobtained, of which 45 were downregulated in the normal group

**Additional file 6.** Informationon the annotation of the methylation sites and the distance between the probeCpG site and the transcription start site

## Data Availability

The data sets used and/or analyzed during the current study are available from the corresponding author on reasonable request.

## Abbreviations

OA: Osteoarthritis
DEGs: Differentially expressed genes
SVM: Support Vector Machines
MRI: Magnetic resonance imaging
PPI: Protein–protein interaction
BP: Biological process
AUC: Area under the ROC curve
ASK1: Apoptosis signal-regulated kinase 1
COL1A1: Collagen type I alpha 1
FGF18: Fibroblast growth factor 18
TLR4: Toll-like receptor 4
