# Supplementary material for: Identification and development of the novel 7-genes diagnostic signature by integrating multi cohorts based on osteoarthritis
Source: Hereditas. 2022 Jan 29;159:10. doi: 10.1186/s41065-022-00226-z (PMC8801091; doi:10.1186/s41065-022-00226-z)
Supplement: Supplementary file 1 — Additional file 1. Tableshowing the 268 differentially expressed genes in the GSE129147 dataset, ofwhich 188 were upregulated and 80 were downregulated [file 41065_2022_226_MOESM1_ESM.docx]

"","logFC","AveExpr","t","P.Value","adj.P.Val","B"

"HDAC9",-0.656823177777777,4.36520536842105,-6.36115583916472,3.44130532545668e-06,0.0309887642933919,4.43140047332974

"BZW2",0.934632296296302,6.69860377192982,5.83498689611198,1.07814171686346e-05,0.0309887642933919,3.42663850894976

"CYBB",1.02053200000001,4.90904210526316,5.82125134263775,1.11126802412208e-05,0.0309887642933919,3.3998501732956

"STXBP6",1.28499722222222,6.14230460526316,5.72394232931424,1.37785317556042e-05,0.0309887642933919,3.20929107485944

"PDE3B",-1.61340218518518,4.94623947368421,-5.71400405257921,1.40853303999728e-05,0.0309887642933919,3.18975312158015

"IL17RB",-2.08927722222222,6.91245263157895,-5.62206262299882,1.72773434724142e-05,0.0309887642933919,3.00834773783703

"PPP1R14B",0.859261333333338,8.54000947368421,5.58188744123294,1.8895946689433e-05,0.0309887642933919,2.92871410793976

"LOX",1.31533940740741,7.99316815789474,5.56223643822989,1.97433061598462e-05,0.0309887642933919,2.88968295905156

"CD163",2.69076566666667,5.69572578947368,5.39690651431989,2.86031398210706e-05,0.0337729587940212,2.55928260287465

"LDHD",-0.894662222222221,5.60556736842105,-5.26377509236654,3.86311990933084e-05,0.0337729587940212,2.29071395704542

"FCGR3A",2.572855,5.44527421052632,5.23262493771236,4.1455947291136e-05,0.0337729587940212,2.22756597207565

"OSTC",0.732971666666671,10.5542728947368,5.20978462119121,4.36601049330149e-05,0.0337729587940212,2.18119185696798

"ATP6V1A",1.10081282222222,6.58957694736842,5.19918160221137,4.47235275843123e-05,0.0337729587940212,2.15964344223728

"TMEM158",1.54027677777778,6.40859421052632,5.19781929684601,4.48620576666683e-05,0.0337729587940212,2.15687390815865

"TDRD6",-1.04292738888889,5.14991973684211,-5.15467705896535,4.94826286814267e-05,0.0337729587940212,2.06905792414072

"TRAF1",-0.594120962962962,4.43503771929825,-5.1478790832058,5.02536772361968e-05,0.0337729587940212,2.05520159522577

"CMIP",0.727636833333338,6.18256092105263,5.14752015066072,5.02947266619553e-05,0.0337729587940212,2.05446983947308

"TNFRSF12A",1.77985933333334,7.08953473684211,5.14519479053119,5.05614948579942e-05,0.0337729587940212,2.04972878173231

"FBN1",0.807341733333339,7.35223536842105,5.14098243850112,5.1048412618944e-05,0.0337729587940212,2.04113890742585

"WNK2",-0.798753111111109,5.12718473684211,-5.12110244438973,5.34115876107431e-05,0.0337729587940212,2.00057302014244

"IQCA1",-1.02480933333333,6.15041,-5.10117276347116,5.58924457171848e-05,0.0337729587940212,1.95986243485734

"CTNNAL1",0.593687037037038,7.13297315789474,5.09993982400241,5.60497180301232e-05,0.0337729587940212,1.95734248341469

"DOK5",0.99287705555556,6.06713631578947,5.0266631827837,6.62524548417691e-05,0.0356532853412777,1.80728615495959

"CD276",0.939318000000002,6.59334289473684,4.93710520274049,8.13261244218585e-05,0.0359518513877381,1.62314515822281

"SORBS1",-0.592372396825394,5.09157330827068,-4.9296955832658,8.27195635728111e-05,0.0359518513877381,1.60787508229608

"ARL4C",2.04354048148149,6.21256280701754,4.91142973003186,8.62589884879775e-05,0.0359518513877381,1.57020980101792

"CA12",1.61776293333333,5.76969084210526,4.90498129097317,8.7544894059708e-05,0.0359518513877381,1.55690526191322

"PLIN3",0.609121333333337,8.17470684210526,4.89447299955108,8.96821921587592e-05,0.0359518513877381,1.535216090798

"ARMC9",-0.875762333333332,6.67845078947368,-4.88515617709159,9.16213892546552e-05,0.0359518513877381,1.5159776127749

"SLC25A27",-1.24077440740741,5.7445249122807,-4.85922516373554,9.72455452508789e-05,0.0362641673968189,1.46239067729647

"NAV1",1.02330440740741,7.10701552631579,4.82353427086329,0.000105563805550341,0.0362641673968189,1.38853707792928

"TCEAL5",-1.350647,4.99803315789474,-4.81956314802958,0.000106532841076345,0.0362641673968189,1.38031295944531

"FHL2",1.26997148148148,5.84989456140351,4.80721649693304,0.000109603634720675,0.0362641673968189,1.3547346761411

"MMP14",1.37215469444445,7.52917723684211,4.80418728861365,0.000110370646545084,0.0362641673968189,1.34845715559981

"ATP5C1",0.661727592592598,8.38532842105263,4.79910043896097,0.000111670916326812,0.0362641673968189,1.33791378251594

"CACNA1A",1.00008733333334,5.68418175438596,4.74487450949885,0.000126534527917885,0.0382361741735868,1.22538864623608

"RHOU",-1.34786427777778,6.21467052631579,-4.73829936046,0.000128468040445696,0.0382361741735868,1.21172836722859

"LOXL1",2.22811144444445,7.71785578947368,4.72818740544332,0.00013150012173074,0.0382361741735868,1.19071355269273

"SH3KBP1",0.750633083333338,5.58653921052632,4.7160071001498,0.000135248725347708,0.0382361741735868,1.16538976013827

"HMCN1",2.32041977777778,7.75761578947368,4.71476474104246,0.0001356370986012,0.0382361741735868,1.16280616107437

"C1GALT1",1.26716833333333,4.42102736842105,4.71238957464855,0.000136382741379045,0.0382361741735868,1.15786645893178

"SLC38A5",1.27092255555556,6.08103105263158,4.70328391433266,0.000139279933557795,0.0382361741735868,1.13892519647388

"ST6GAL2",1.50418066666667,4.33613736842105,4.68982311418784,0.000143677273903176,0.0382361741735868,1.11091315147157

"CDKN1A",0.954291055555561,7.65388789473684,4.68183617699341,0.000146352530678662,0.0382361741735868,1.09428593044447

"GPR137B",0.899683222222228,6.50090526315789,4.68037986206301,0.000146845731879306,0.0382361741735868,1.09125366785384

"EVA1A",0.901463444444444,4.25728,4.67016761591609,0.000150351789141588,0.0382361741735868,1.06998594038512

"ADAM12",0.941365088888891,5.46626442105263,4.66472589596207,0.000152254476401328,0.0382361741735868,1.05865010497547

"METRNL",0.924864944444447,7.33181157894737,4.65689856580895,0.000155034059040959,0.0384219276583744,1.04234102226707

"IRAK1",1.01558177777778,6.64092894736842,4.64452031284,0.000159534818721912,0.038742775693641,1.01654079870253

"SLC7A9",-0.773881444444442,4.69646842105263,-4.62880870044771,0.000165438319910469,0.038742775693641,0.98377751826296

"KDELR1",0.692192444444449,7.39424070175439,4.60289583150241,0.000175661133697051,0.038742775693641,0.929705299701365

"SHISA3",-1.08811472222222,5.45423526315789,-4.59997430118339,0.000176853019789137,0.038742775693641,0.92360618542501

"C3AR1",1.91644288888889,5.98767526315789,4.59691837921139,0.000178108473764643,0.038742775693641,0.917225917364889

"WIPF1",0.595178222222225,5.51632947368421,4.59017473391778,0.000180910880363302,0.038742775693641,0.903144155875365

"GALNT1",0.878211977777781,7.93669305263158,4.58938780157806,0.000181240787753112,0.038742775693641,0.901500731297871

"UMODL1",-1.00798311111111,4.71462368421053,-4.58913534973426,0.000181346752343021,0.038742775693641,0.900973504084035

"TBX4",-0.740417944444444,4.92812578947368,-4.56635181036724,0.000191172124641387,0.038742775693641,0.853375240157496

"APOL3",-0.961361703703701,6.38844052631579,-4.56406209039853,0.000192188814568491,0.038742775693641,0.848589888011989

"LVRN",-2.14165566666667,6.05193947368421,-4.56219021839581,0.000193024023217825,0.038742775693641,0.844677570548689

"SNX7",1.52460204444445,7.02599768421053,4.55904946714381,0.000194433629958582,0.038742775693641,0.838112745537062

"MPPED2",-1.25814611111111,5.14412228070175,-4.55496292159029,0.000196283285598663,0.038742775693641,0.829570119823344

"TPM4",1.48702757777778,6.89919431578947,4.54056423533257,0.000202943096490157,0.038742775693641,0.79946276631188

"AQP6",-0.607017388888889,4.36618105263158,-4.53659128128433,0.000204820518487961,0.038742775693641,0.791153251389293

"WSCD2",-1.06958466666667,6.07792236842105,-4.53050056533497,0.000207732769915026,0.038742775693641,0.778412614812096

"BEND6",0.843134444444448,4.84945385964912,4.53036956942928,0.000207795861847364,0.038742775693641,0.778138572413885

"GPRASP1",-0.86170922222222,5.93223605263158,-4.51941067263063,0.000213143058583052,0.038742775693641,0.755209179371288

"CNTFR",-0.64876533333333,7.32117789473684,-4.51731576755213,0.000214180923706044,0.038742775693641,0.75082521791702

"LYVE1",0.70951588888889,5.15279052631579,4.51394887486663,0.000215859650682502,0.038742775693641,0.743778881365535

"SLC7A7",0.751163444444447,5.71203473684211,4.50776991653408,0.000218975067974843,0.038742775693641,0.730845728647567

"C3orf70",-1.11352096296296,5.03318929824561,-4.49837488843173,0.000223799129222039,0.038742775693641,0.711177019003294

"H2AFY",0.825062888888894,9.05727140350877,4.49441707426505,0.000225863291897679,0.038742775693641,0.702889812164755

"RALA",0.951056185185187,6.30132403508772,4.49265836604199,0.000226786679689286,0.038742775693641,0.699207010379529

"HYI",-0.749944777777774,7.27454,-4.49248457130857,0.000226878134812949,0.038742775693641,0.698843068646619

"LMAN1",0.659921833333337,6.30759394736842,4.48974974985516,0.000228322171595123,0.038742775693641,0.693115896053541

"CRNDE",1.25875011111111,9.49712894736842,4.45995956124264,0.00024466500641505,0.0400092374517702,0.630705085382942

"PLAU",1.06727807407408,5.08836456140351,4.45045714398581,0.00025012213637764,0.0400092374517702,0.610787995223109

"PAICS",0.615801222222228,7.05142368421053,4.44835314988465,0.000251346923563012,0.0400092374517702,0.60637742211217

"RRS1",0.595369111111115,6.18314105263158,4.43534053564323,0.000259057273295207,0.0400092374517702,0.579094552403427

"ALOX5AP",1.21293677777778,6.09566,4.42708443424785,0.000264072331200883,0.0400092374517702,0.56178028927922

"HMGN1",0.819935185185194,8.70580298245614,4.42189792885756,0.000267272688879321,0.0400092374517702,0.550901819508277

"CCDC112",0.995609888888891,4.89321298245614,4.41895602714369,0.000269105332516751,0.0400092374517702,0.544730766428471

"HSPB9",-0.764283555555554,4.51737105263158,-4.40526150723906,0.000277804206183155,0.0400092374517702,0.515999515741203

"GNB1",0.907090277777781,7.72680315789474,4.4026779994507,0.000279476738865218,0.0400092374517702,0.510578369052134

"COL7A1",1.36789244444445,7.65225894736842,4.4012678694378,0.000280393912590054,0.0400092374517702,0.507619278040551

"GARS",0.675415944444453,9.76572763157895,4.39121361584609,0.000287021781846549,0.0405373727725693,0.486518455399508

"SLC38A6",1.05757588888889,7.28458,4.35562920618762,0.00031177172139017,0.0413566411623498,0.411804628990865

"FAM32A",0.828963666666668,8.00367,4.3525799840826,0.000313989895737511,0.0413566411623498,0.405400137920118

"CYP3A5",-0.734891074074073,4.23910929824561,-4.3399204572001,0.000323370150615089,0.0417169642933918,0.378806827824144

"ARF6",0.636623111111119,7.96523701754386,4.33255512181549,0.00032895672699491,0.0419526157502215,0.363332166857472

"CFH",-1.51650972222222,11.6190592105263,-4.33042437187189,0.000330590913769584,0.0419526157502215,0.358855083855621

"MEX3D",1.87551766666667,6.71681789473684,4.3287524206151,0.000331878935321636,0.0419526157502215,0.355341909344647

"ALDH1L1",-0.631393166666665,4.66298447368421,-4.32118545021363,0.000337771672432198,0.0421641763809033,0.339440692027115

"LDLRAD2",-0.59668561111111,5.54466605263158,-4.31509932773619,0.000342587428210289,0.0421641763809033,0.326649949869159

"POSTN",3.73571548888889,8.48579115789474,4.31197495746705,0.000345086385961208,0.0421641763809033,0.32008323136994

"MTCH2",0.723000000000003,6.77811131578947,4.29923682164223,0.000355465706254673,0.0421641763809033,0.29330737395973

"VSIG4",1.15615918518519,5.26483298245614,4.29814239005384,0.000356371974173169,0.0421641763809033,0.29100662037296

"NRBF2",0.854791577777783,6.90099852631579,4.29508842203906,0.000358913173578994,0.0421641763809033,0.284586268152444

"CMTM3",1.20317511111112,8.03356894736842,4.29228086085481,0.0003612653952065,0.0421641763809033,0.27868369169663

"GGCT",0.662827222222224,7.80550421052632,4.27925644477185,0.000372381637300859,0.0421641763809033,0.251298359641912

"FRMD6",0.942855111111114,6.54441842105263,4.27222565043768,0.000378524482660591,0.0421641763809033,0.236513333372282

"TXN",0.867136800000003,10.2275557894737,4.27067086703719,0.000379896586667987,0.0421641763809033,0.233243605664577

"GPX8",0.75018355555556,9.29338289473684,4.26767104279997,0.000382558075595146,0.0421641763809033,0.226934758728892

"CDKN3",0.973579500000004,4.99886901315789,4.26739724901976,0.000382801919890335,0.0421641763809033,0.226358938835779

"TWIST1",1.84907900000001,8.62202947368421,4.2610231692927,0.000388523073039206,0.0425455353528689,0.212952964754993

"GNAI3",0.741012250000003,7.16837894736842,4.25462597308471,0.000394351287055133,0.0428802385369261,0.199497335972842

"RORC",-0.707774555555555,5.06370929824561,-4.23569129740707,0.000412121233792992,0.0428802385369261,0.159665080182316

"ITPA",0.667639444444447,7.35028973684211,4.22756279146853,0.000419994188535998,0.0428802385369261,0.142562933268903

"KLF10",0.694403277777784,9.39157763157895,4.22426687212046,0.000423229323765519,0.0428802385369261,0.135628013553633

"ABCA5",-1.11363940740741,6.86968807017544,-4.21724608620238,0.000430204215277552,0.0428802385369261,0.12085489258781

"BLNK",1.25293877777778,4.70589368421053,4.20983760866809,0.000437689329485841,0.0428802385369261,0.105264946709506

"TSHZ3",0.935461222222225,5.94065578947368,4.20939976804221,0.000438135765056763,0.0428802385369261,0.104343549298666

"NAP1L2",-1.11661751851852,4.46838842105263,-4.2065850107647,0.000441016701521199,0.0428802385369261,0.0984200538302193

"AP2S1",0.724070851851855,8.07383140350877,4.20639725162978,0.000441209549651219,0.0428802385369261,0.0980249203399008

"HLA-DRA",1.95708585185186,7.39503,4.20257586682557,0.00044515293584293,0.0428802385369261,0.0899827927088799

"ACTG1",0.818707333333341,12.0041312631579,4.20132790553226,0.000446448379721053,0.0428802385369261,0.0873563945386815

"CPAMD8",-1.23166418518518,6.33112087719298,-4.20106330297323,0.000446723535256977,0.0428802385369261,0.0867995214666415

"RHOJ",-0.599483916666663,6.64200131578947,-4.19591128404418,0.000452115045487512,0.0428802385369261,0.0759565273644176

"TCEAL6",-1.71528738888889,7.54603105263158,-4.19310910156816,0.000455074829878193,0.0428802385369261,0.0700588370175739

"PON3",-1.34110005555555,5.37555342105263,-4.19041710943555,0.000457936520087246,0.0429116137106631,0.0643929411877329

"PRSS23",2.95327664444445,8.90467168421053,4.1838435799311,0.000465000473133092,0.0433578411458505,0.0505570071804895

"LPCAT1",0.902211666666672,7.91340157894737,4.17329637994275,0.000476563484138544,0.0438949526287981,0.0283559689500343

"TYMS",1.53242516666667,6.16818289473684,4.16615930410529,0.000484550977810966,0.0438949526287981,0.0133321104505892

"NPTX2",1.49998866666667,5.74713105263158,4.16483381029342,0.000486049114657775,0.0438949526287981,0.0105418155284323

"EIF4EBP1",0.611776185185189,7.16839561403509,4.16392909191231,0.000487074334983743,0.0438949526287981,0.00863728175585265

"MAATS1",-0.944989777777777,5.07501473684211,-4.15534159631197,0.000496914155401447,0.0441823026144049,-0.00944084694157343

"TK1",1.04581972222222,5.64693421052632,4.15330179113644,0.00049928054882355,0.0441823026144049,-0.0137351048739323

"NPDC1",-0.83595544444444,7.57109789473684,-4.15298772890813,0.000499645896303066,0.0441823026144049,-0.014396281950396

"GRK5",-0.620013977777776,4.93521063157895,-4.13750846494367,0.000517988666256948,0.0447580961564793,-0.0469851113040285

"CMAHP",-0.825671066666665,7.33746094736842,-4.13671231170477,0.000518950127536894,0.0447580961564793,-0.0486613300257241

"TENM3",0.79430255555556,5.0217850877193,4.12385383988144,0.00053472827618346,0.0450726178919612,-0.0757342129043383

"TTK",0.780414666666669,4.28082789473684,4.1218423722297,0.000537239549500888,0.0450726178919612,-0.0799693666201051

"APLN",1.19463055555556,6.24354736842105,4.11673826209845,0.000543665035866757,0.0450726178919612,-0.0907162050158323

"FAM71A",-0.592529777777775,3.59144,-4.1160408756809,0.000544548917894639,0.0450726178919612,-0.0921845823042764

"IGFBP3",1.88187527777778,7.13846789473684,4.11235478330908,0.000549244696291231,0.0450726178919612,-0.0999458528381796

"LOXL2",0.991007722222224,5.86563578947368,4.11225043552481,0.000549378215038238,0.0450726178919612,-0.100165563868047

"NFIA",-0.763449253968251,6.95211714285714,-4.10834654037753,0.000554396875301608,0.0450726178919612,-0.108385505941622

"LAMTOR2",0.680373722222225,7.34023421052632,4.10480083749791,0.000558994810429621,0.0450726178919612,-0.115851306118389

"THY1",2.24945955555556,7.70074552631579,4.09515811759418,0.000571693046633807,0.0450726178919612,-0.13615516051816

"TTLL7",-0.650365666666662,6.04095894736842,-4.09420923777311,0.000572958093281558,0.0450726178919612,-0.138153150715098

"ACTR3",1.35069438888889,7.79331289473684,4.09377004515732,0.000573544572673807,0.0450726178919612,-0.139077928769545

"HLA-DRB1",1.91243744444445,8.25369157894737,4.09236149228013,0.000575429550022717,0.0450726178919612,-0.142043825818487

"SERPINA6",-1.85377855555555,6.29657263157895,-4.09008110861097,0.000578494391418778,0.0450726178919612,-0.146845488240065

"CMYA5",-0.863854888888886,4.97139842105263,-4.08056618305063,0.000591459684258893,0.045119720894379,-0.166880556470535

"RRAGC",0.990951583333339,7.19388513157895,4.08039558879102,0.000591694773608262,0.045119720894379,-0.167239768342493

"NCBP2",0.603652155555556,5.14247610526316,4.073581667818,0.000601161583802268,0.045426428346538,-0.181587504570857

"PSAT1",0.78962229166667,5.21900401315789,4.06706770510342,0.000610353232042322,0.045426428346538,-0.19530360619935

"ARPC3",1.18568383333334,8.75095078947368,4.06598432973242,0.000611895521048418,0.045426428346538,-0.197584806057011

"MAP2K6",-1.57158244444444,5.79037078947368,-4.06468873065648,0.000613745048101347,0.045426428346538,-0.200312870161884

"LRR1",0.710200888888894,6.01787105263158,4.06444573602764,0.000614092556037214,0.045426428346538,-0.200824528803316

"NID2",1.83264716666667,7.10138447368421,4.06380391510157,0.000615011373951006,0.045426428346538,-0.202175970543905

"MCCC2",-0.598850277777776,6.62794342105263,-4.05805969274651,0.000623296127414652,0.0455086699256769,-0.21427117339924

"RRBP1",0.705011925925929,6.35127631578947,4.05790106934791,0.000623526482294802,0.0455086699256769,-0.214605174226153

"FXYD1",-1.27899127777777,7.31216447368421,-4.05327896101187,0.000630276265020253,0.0455086699256769,-0.224337549366114

"LY96",1.33938066666667,7.03299052631579,4.04762046997998,0.0006386390203838,0.0455086699256769,-0.236252038434982

"PCDHB5",-1.19103577777778,6.64465684210526,-4.04366275743679,0.000644553996276272,0.0455086699256769,-0.244585293214527

"ZNF204P",-1.51620188888889,5.98104789473684,-4.03652367801192,0.0006553624549922,0.0455086699256769,-0.259616941839715

"PNMA1",1.023168,7.19664947368421,4.03069283911867,0.000664324482204221,0.0455086699256769,-0.271893801231673

"NRAS",0.758799444444448,7.08952368421053,4.02815719215566,0.00066825988693094,0.0455086699256769,-0.277232545135376

"CKS2",1.44269811111112,6.29215368421053,4.02744419377136,0.000669370672427009,0.0455086699256769,-0.27873373764306

"ADHFE1",-0.646604222222221,5.08957921052632,-4.02151009806064,0.00067868729029174,0.0455086699256769,-0.291227609263368

"PICALM",0.858995037037041,5.37958061403509,4.01987487229066,0.000681277307651439,0.0455086699256769,-0.294670425123454

"LIMCH1",-0.749652111111111,7.14835536842105,-4.01665105482814,0.00068641243198372,0.0455086699256769,-0.301457804428667

"RAB10",0.694566277777779,6.97021842105263,4.01437483800136,0.000690061417282446,0.0455086699256769,-0.306250061694861

"EVI2A",1.24422866666667,7.44321052631579,4.012702980931,0.000692753892651677,0.0455086699256769,-0.309769892380222

"OLFM1",-0.625191472222219,5.53439407894737,-4.0104404292264,0.000696414377946283,0.0455086699256769,-0.314533293520038

"ZNF385C",-0.797086777777777,4.76228842105263,-4.00654196766214,0.000702766900470469,0.0455577521712625,-0.322740689903731

"SPON2",0.960406111111112,8.55265578947368,4.0043985304407,0.000706284238598813,0.0455577521712625,-0.327253180281057

"BASP1",2.4577952962963,8.3040801754386,3.99437084755234,0.000722974357463999,0.0455853243881357,-0.348363349718467

"LRRC15",0.963334740740742,5.60388087719298,3.992809805215,0.000725607747608771,0.0455853243881357,-0.35164952905318

"SOD3",-1.29075694444444,9.28458368421053,-3.99237563322766,0.000726341871575075,0.0455853243881357,-0.352563507060029

"NOP10",0.691323666666673,9.02249789473684,3.98817666135914,0.000733480112781668,0.0455853243881357,-0.361402659929797

"KNSTRN",0.652340444444445,5.54471263157895,3.98779888247754,0.000734125758969045,0.0455853243881357,-0.362197901445139

"BEX5",-1.88044822222222,7.24741263157895,-3.98689545752823,0.000735672066200334,0.0455853243881357,-0.364099643642655

"SELENBP1",-0.657616555555552,7.80540157894737,-3.98556076707657,0.000737962480701412,0.0455853243881357,-0.366909194726655

"KLF15",-1.24717533333333,6.0678852631579,-3.9840758096038,0.000740519127939441,0.0455853243881357,-0.370035031733924

"TMEM50A",1.02346558333334,8.59489026315789,3.97997620579197,0.000747623386195036,0.0455853243881357,-0.378664537798774

"ZBTB16",-1.45745655555555,6.48363315789474,-3.97990819630086,0.00074774181247752,0.0455853243881357,-0.378807693049793

"FAN1",-0.662688888888883,7.01191026315789,-3.97977994210717,0.000747965195002697,0.0455853243881357,-0.379077659024758

"COL8A1",0.799807148148151,4.75361964912281,3.97845537710015,0.000750276111511658,0.0455853243881357,-0.381865759981063

"SLC39A7",0.967560481481485,6.89791421052632,3.97635312099275,0.000753958471124308,0.0456617614264512,-0.386290782592771

"CEMIP",0.712903388888891,6.50071342105263,3.9702468355086,0.000764756958897861,0.0458596340415443,-0.399143464160635

"SLC26A11",-0.585177555555553,7.39388736842105,-3.96456380650357,0.000774945531558482,0.0458596340415443,-0.411104715939506

"PLXDC1",1.7537885,6.92303407894737,3.96052416710747,0.000782270051977935,0.0458596340415443,-0.41960673603554

"CD300A",1.02711688888889,5.30542894736842,3.95942918275489,0.000784267304429571,0.0458596340415443,-0.421911243078115

"KCNT2",-0.754033444444438,5.58279140350877,-3.95845581752211,0.000786046993276821,0.0458596340415443,-0.42395977200261

"EPDR1",0.812403629629629,4.77207789473684,3.95704698639459,0.000788630019219411,0.0458596340415443,-0.426924744867348

"BLMH",1.241902,6.62391263157895,3.95267871276805,0.000796692985899731,0.0459910457907179,-0.436117814018009

"TMEM101",0.692907333333336,6.35717473684211,3.94493718939407,0.000811184770865851,0.0464397117302684,-0.452409010288937

"STOX2",-1.05851044444444,5.61143368421053,-3.94153663058814,0.000817633269369922,0.0466670382684318,-0.459564735790573

"THBS2",2.22444600000001,8.50368105263158,3.93670935923044,0.000826875116431059,0.0467864729557252,-0.469722246011342

"HTATIP2",0.873168222222224,6.09797333333333,3.93655212545111,0.000827177886607724,0.0467864729557252,-0.470053087813673

"COL5A1",1.48334652777778,10.9425509210526,3.92703070453149,0.00084571984661337,0.0469039451222881,-0.490086461620237

"ARPC1B",1.55276233333334,7.91287,3.92535797794589,0.000849019825570886,0.0469039451222881,-0.493605718065503

"STX2",0.77505222222223,8.12072,3.92382263638499,0.000852060063341834,0.0469039451222881,-0.496835871677939

"FAM49B",0.849972518518518,6.35442877192982,3.92342536970922,0.000852848484313369,0.0469039451222881,-0.497671658611589

"USP53",-1.60843888888888,8.61667710526316,-3.92310973157554,0.000853475422415898,0.0469039451222881,-0.498335709182534

"MAP7D1",0.733033000000004,7.12277184210526,3.92153281913205,0.000856614459773443,0.0469039451222881,-0.501653237122594

"GPM6A",-1.23896392592593,4.40495631578947,-3.9208522242016,0.00085797281927517,0.0469039451222881,-0.503085062664638

"MYO5A",0.954261555555559,6.73782736842105,3.91848104351872,0.00086272210765956,0.0469039451222881,-0.508073429426228

"COL1A1",2.94957055555556,8.915626,3.91826881386926,0.000863148462100285,0.0469039451222881,-0.508519900418685

"ARHGAP22",1.00119611111112,5.99638421052632,3.91778641868095,0.00086411834124948,0.0469039451222881,-0.50953471894853

"AIM1",0.658062518518518,4.47837350877193,3.90964181095542,0.000880658380032857,0.0474756554660966,-0.526667709974044

"MS4A6A",1.6754735,5.54139315789474,3.90412229433055,0.00089204632087566,0.0474756554660966,-0.538277583487085

"ITGAM",0.805931296296295,4.43974140350877,3.8999614892462,0.000900727812037262,0.0474756554660966,-0.547028971360569

"KLF13",0.657112055555556,6.79720789473684,3.89815085954649,0.000904531908179944,0.0474756554660966,-0.550837105447668

"PDGFC",0.703149740740743,6.7791747368421,3.89519928747924,0.000910767432853227,0.0474756554660966,-0.557044683804396

"AIF1",1.22506504444445,5.40315863157895,3.89244355596249,0.000916627858363873,0.047561117664693,-0.56284015793596

"LGALS1",1.17921777777778,11.6958131578947,3.89017722147576,0.000921475646367085,0.0476391112346098,-0.567606235540471

"TSPAN5",0.894799111111114,5.78804078947368,3.88713183250994,0.000928030091390543,0.0476391112346098,-0.574010419501281

"CCDC85A",-2.14565477777778,7.10214578947368,-3.88088458493028,0.000941621381585721,0.0476759105434598,-0.587146960158593

"MSANTD3",0.868331666666669,7.22108,3.87496789757031,0.000954676169756274,0.0482073073923845,-0.599587300186148

"DR1",0.624265018518522,6.38505535087719,3.87337997151081,0.000958210387159793,0.0482563974389163,-0.602925862275337

"MMP2",1.81906602777778,7.23662289473684,3.86893294541658,0.000968177504634659,0.048522215176438,-0.612275155581104

"ADAMTS4",0.861046055555557,4.93181157894737,3.86872726583081,0.000968640982550607,0.048522215176438,-0.612707554552054

"MID1",0.680252177777781,4.88571368421053,3.86583863253633,0.000975173597817646,0.048590991309247,-0.618780163799854

"SERPINH1",1.63722133333334,8.45576877192982,3.86335830811393,0.000980817800474593,0.0486706419009658,-0.623994186945025

"KLKB1",-0.674020444444442,4.09042684210526,-3.85966635168229,0.000989279442446457,0.0486706419009658,-0.631754861795155

"S100A16",0.992304055555558,7.40562473684211,3.85948698814149,0.000989692373138832,0.0486706419009658,-0.63213188096611

"COL1A2",1.36701048148149,11.8505927192982,3.85033999409241,0.00101097951763782,0.0487394759619389,-0.651357222337086

"NPR3",0.849319000000002,4.91155,3.84999635024441,0.00101178807232117,0.0487394759619389,-0.652079442332127

"BCL6",-0.895747111111111,8.44508526315789,-3.84844192551386,0.00101545349923949,0.0487394759619389,-0.655346249981815

"MGST3",0.604054444444449,10.6888689473684,3.8466198607817,0.0010197668565806,0.0487495145779077,-0.659175423285034

"ARF5",0.782172222222231,7.81113736842105,3.84465693782479,0.00102443406039178,0.0487762813399377,-0.663300482431967

"CREB3L1",0.714818055555555,6.23076947368421,3.84286842245411,0.00102870507404142,0.0487762813399377,-0.667058902448019

"ADAM9",0.956771805555563,6.96749605263158,3.84204164215107,0.00103068542465066,0.0487762813399377,-0.66879627407357

"INHBB",1.33668511111111,6.01216736842105,3.83640443772263,0.00104428940288405,0.0491729772583026,-0.680641443512759

"TUBB",0.81164894444445,8.77274587719298,3.83185593634209,0.00105539611320728,0.0492658062458402,-0.690198091655041

"ZHX2",-0.995262888888885,8.04806631578947,-3.82826657868964,0.00106424359801177,0.0492658062458402,-0.697738957234004

"NUSAP1",0.97541551851852,4.2066347368421,3.82418744699884,0.00107438788643481,0.0492658062458402,-0.706308166085494

"CCND1",0.667802472222227,5.3428002631579,3.82372524633423,0.00107554336750427,0.0492658062458402,-0.707279088920488

"TMEM200A",1.06411416666667,4.90925342105263,3.82284165581182,0.00107775574374067,0.0492658062458402,-0.709135181218917

"TMEM65",0.986105722222226,5.71051578947368,3.81989826729465,0.00108515822634145,0.0492658062458402,-0.715317907272879

"RAB31",1.77392305555556,8.49793565789474,3.81337859923821,0.0011017353152981,0.0492658062458402,-0.729011502500925

"WISP1",0.883932472222224,4.97280789473684,3.81028243122516,0.00110969562019147,0.0492658062458402,-0.735513918293458

"TPST2",1.73406233333334,6.42488105263158,3.80651497349324,0.0011194589467636,0.0492658062458402,-0.74342558951044

"TUBB3",1.14911344444445,6.53582210526316,3.80440945005202,0.00112495252045602,0.0492658062458402,-0.747846926491953

"PFN1",1.11395966666667,7.53679789473684,3.80184354707118,0.00113168350555879,0.0492658062458402,-0.753234740633999

"CDK6",0.825449250000003,6.17280947368421,3.79608993014653,0.00114692244014107,0.0492658062458402,-0.765314968006733

"TCEAL2",-1.74895977777778,9.60558263157895,-3.78941680661055,0.00116485223568608,0.0492658062458402,-0.779323903391508

"ARHGAP18",1.02819492592593,5.03059263157895,3.78004170860121,0.00119051309038602,0.0492658062458402,-0.799001611054124

"SOX4",0.8901937936508,8.55549939849624,3.77981297408864,0.00119114613134279,0.0492658062458402,-0.799481657420164

"RAB13",0.909258222222226,9.07820473684211,3.77872168607957,0.00119417096207033,0.0492658062458402,-0.801771914836915

"NNT",0.781621200000004,6.58951957894737,3.77593824246332,0.00120192068707844,0.0492658062458402,-0.807613193844054

"PPFIBP1",0.65538803703704,6.61467403508772,3.77515836601346,0.00120410097756935,0.0492658062458402,-0.809249759348186

"GMDS",-0.591441222222219,9.05150736842105,-3.77256321368197,0.00121138452388423,0.0492658062458402,-0.814695454512063

"MYOF",1.1849773888889,8.86704842105263,3.77029941706784,0.001217773796389,0.0492658062458402,-0.819445557975227

"C1QB",1.23994527777778,6.977545,3.76733424321445,0.00122619324628735,0.0492658062458402,-0.825666968736478

"EFHD2",0.762517500000001,5.736185,3.766185808691,0.00122946966497004,0.0492658062458402,-0.828076450210466

"TNFRSF19",0.947923074074077,5.37549947368421,3.76137899012888,0.0012432778088638,0.0492658062458402,-0.838160705427055

"EZR",1.14416173333334,5.98513305263158,3.75880126103462,0.00125074592213216,0.0492658062458402,-0.84356805228501

"CALD1",1.2798381,7.279584,3.75611190761698,0.00125858488354632,0.0492658062458402,-0.849209188488619

"COL6A3",1.1519605925926,10.3181757894737,3.75599634662211,0.0012589228124943,0.0492658062458402,-0.849451578569213

"RBBP8",0.792981000000003,7.26757842105263,3.75577081938569,0.00125958256928759,0.0492658062458402,-0.849924621680163

"F3",0.955305407407412,4.8510449122807,3.75408048555022,0.00126453840219483,0.0492658062458402,-0.853470010002402

"GDF10",-2.45913233333333,9.17164315789474,-3.75047255434835,0.00127518118849097,0.0492658062458402,-0.861036955970055

"BNIP3L",0.604083203703707,9.3772898245614,3.74968531440955,0.00127751519864018,0.0492658062458402,-0.862687949345446

"ABCA10",-0.710123583333331,4.90067407894737,-3.74798516930184,0.00128257026213326,0.0492658062458402,-0.866253367133225

"ZNF267",0.795080703703705,4.85424315789474,3.74174096629972,0.00130130709760047,0.0495903722936131,-0.879346905426809

"CLMP",0.649285777777781,6.2009249122807,3.73453635043438,0.00132326339792228,0.0496487372507294,-0.894451683646602

"S100B",-1.21843099999999,9.99594719298246,-3.73131243712923,0.00133320699008772,0.0497135842069959,-0.90120981263635

"WIPI1",0.846157388888892,7.40601710526316,3.73003704180371,0.00133716114564566,0.0497135842069959,-0.903883197915612

"CFAP70",-1.50530722222222,5.69429526315789,-3.72816894128441,0.00134297388779443,0.0497135842069959,-0.907798798712767

"DNAJB11",0.60005211111112,8.69090578947368,3.7280106055206,0.00134346771231011,0.0497135842069959,-0.908130666560513

"COX7A2L",0.645729888888893,8.31884394736842,3.72500687093573,0.00135287007136021,0.049768179285292,-0.914426146900958

"IFNLR1",-0.946205222222219,5.33832947368421,-3.72060579611078,0.00136676436285877,0.0497959486264993,-0.923649346240655

"GPR183",1.14143377777778,4.28007684210526,3.72014372241044,0.00136823132082405,0.0497959486264993,-0.924617634446319

"GBP2",-0.915561222222219,7.55230789473684,-3.71783681101876,0.00137557851644115,0.0498981588086214,-0.92945164294839

"SUSD3",-0.891910555555555,5.18239894736842,-3.71720512425068,0.00137759716381647,0.0498981588086214,-0.930775253688907
