# Supplementary material for: Identification and development of the novel 7-genes diagnostic signature by integrating multi cohorts based on osteoarthritis
Source: Hereditas. 2022 Jan 29;159:10. doi: 10.1186/s41065-022-00226-z (PMC8801091; doi:10.1186/s41065-022-00226-z)
Supplement: Supplementary file 2 — Additional file 2. Tableshowing the 648 differentially expressed genes in the GSE57218 dataset, ofwhich 309 were upregulated and 339 were downregulated [file 41065_2022_226_MOESM2_ESM.docx]

"","logFC","AveExpr","t","P.Value","adj.P.Val","B"

"IRS2",-1.25071485334199,7.94310874385,-10.930717168597,1.47265059646063e-13,3.1589827944677e-09,20.5310849395636

"PLOD1",1.42114740281385,11.95761701875,9.92763729710084,2.49138935084301e-12,1.42725672252855e-08,17.8684606030091

"LENG8",-0.802797244887446,7.343096276825,-9.86776217600033,2.96177789216478e-12,1.42725672252855e-08,17.7049169647346

"ALDH1L1",-1.16860292274459,7.54329082645,-9.8565405268914,3.05950976323836e-12,1.42725672252855e-08,17.6742088514461

"TNC",2.17380606045454,11.084575985875,9.82761730691916,3.32678365234382e-12,1.42725672252855e-08,17.5949772070808

"BCL6",-0.86948103448918,9.017030723975,-9.59927131514124,6.46866173403776e-12,2.14571585790175e-08,16.9652666717029

"BEX2",-1.72071641268831,8.036750706675,-9.57221573308832,7.00200969899411e-12,2.14571585790175e-08,16.8901657256913

"ANGPTL2",1.63018624748918,13.23582272275,8.94556520233883,4.50078853577612e-11,1.20683018601167e-07,15.1222782704679

"ABCB9",-0.883758638367969,7.729440248775,-8.87575217576694,5.55399948044753e-11,1.25419642957208e-07,14.9220230592437

"CRTAC1",1.47712028831168,13.709193575,8.83285694699816,6.32177915517717e-11,1.25419642957208e-07,14.7986608519458

"SMOC2",2.30084994435498,11.79662026095,8.72232149779748,8.83449411586597e-11,1.42596752290776e-07,14.4796632195449

"CALCOCO1",-0.875146284376623,8.208500339675,-8.60132142459021,1.27642495745866e-10,1.61610473130355e-07,14.1286572269608

"KHDC4",-1.08039688741992,7.96105718845,-8.57270898913924,1.39281409781045e-10,1.61610473130355e-07,14.0453835653341

"APOD",-3.76178528467966,11.647558014425,-8.55603795953984,1.46552457972951e-10,1.61610473130355e-07,13.9968165520417

"CAPS",1.40751002493722,9.1600742332875,8.54293931042989,1.52534118732612e-10,1.61610473130355e-07,13.9586322844334

"ARHGDIB",1.68498496375324,10.660272453525,8.53003886903667,1.5866687952985e-10,1.61610473130355e-07,13.9210047794102

"LOXL3",1.90336675272727,10.04940393,8.5157597855142,1.65746604301329e-10,1.61610473130355e-07,13.8793318181893

"SIN3B",-0.79635768380087,7.62194853015,-8.37061968013986,2.58650062654267e-10,2.41230543217247e-07,13.4543132647163

"RAI1",-1.20737147639394,7.900063274975,-8.33961551518206,2.8453093252803e-10,2.54311376402449e-07,13.3631887204998

"DDIT3",-1.469349312671,8.251204383475,-8.21171945742752,4.22143939344458e-10,3.48284986264537e-07,12.9860714840547

"P4HA2",1.07786189183838,9.72722063276667,8.18792862968067,4.54376008087041e-10,3.60993324054634e-07,12.9157081066624

"ZNF394",-0.996952624584415,8.580173578075,-8.13964373339505,5.27654663713085e-10,3.86326515235692e-07,12.7726990632184

"PHF1",-0.637342195552673,8.05601538305,-8.10682924903032,5.84171120701176e-10,4.04227571295514e-07,12.6753564822003

"CASC3",-0.821491447034635,9.139715070625,-7.91943566465418,1.04669339220516e-09,6.14882235136312e-07,12.1171390285202

"TULP3",-0.673809824290045,7.949096413675,-7.90667099679477,1.08925108084378e-09,6.14882235136312e-07,12.0789742666669

"BET1L",-0.717173216025978,7.85602195435,-7.89457409736937,1.13119504204791e-09,6.22186278127431e-07,12.0427898328677

"LRATD2",-0.616606817614721,7.346106290325,-7.87177993552531,1.21471625975862e-09,6.47397808118802e-07,11.9745649134831

"STAT5B",-0.770846805415587,8.756694900675,-7.84362502443273,1.3265343373603e-09,6.62699391049933e-07,11.8902183640119

"TBC1D2B",-0.947719373359311,8.53749436655,-7.84316952892939,1.32842635845169e-09,6.62699391049933e-07,11.8888530989483

"MCCC1",-0.830613319558445,8.27270175565,-7.81561858511972,1.44808337284625e-09,7.05866665947596e-07,11.8062335148542

"WASF2",-0.870771369021648,8.8183896277,-7.79642221115038,1.53783651002643e-09,7.05866665947596e-07,11.7486205537335

"MXRA5",2.46970187935497,11.535117562325,7.79076059873435,1.56536526335659e-09,7.05866665947596e-07,11.7316213579331

"TSPYL2",-0.81910622840116,7.89104076311667,-7.78789496014779,1.57948813414221e-09,7.05866665947596e-07,11.7230159063517

"C2orf68",-0.85197898414286,7.605333705225,-7.78120885272513,1.6129419667062e-09,7.06106492404382e-07,11.7029343597179

"PXYLP1",1.47456714572727,10.812297162225,7.67982131967638,2.2175317204992e-09,8.97514583706195e-07,11.3978594303309

"TGFBI",2.72327442410822,13.024222315175,7.66210860621105,2.34458043327125e-09,9.24349545926871e-07,11.344455727697

"MRPL2",-0.834706080090914,8.129818756925,-7.65618644074559,2.38867736783418e-09,9.24349545926871e-07,11.3265935094888

"VPS37B",-0.946217118861478,8.594570840225,-7.65295278495,2.41310776056616e-09,9.24349545926871e-07,11.316838814384

"SNRPA",-0.831457985761908,8.312580845175,-7.62135677850988,2.66549868711551e-09,1.0031160059178e-06,11.2214718085127

"CLMP",-0.881823961108231,7.7697101248,-7.61129816138527,2.75131280117478e-09,1.01755880858621e-06,11.1910911965787

"OAZ1",0.797513582380944,14.07504066975,7.58946539338195,2.94729435044671e-09,1.05370685185721e-06,11.125114779009

"H1-10",-0.935934182251087,9.0805715595,-7.54939862826715,3.34435473924267e-09,1.17606153297532e-06,11.0039183527105

"SLC25A27",-0.779020896939396,7.357165026025,-7.51404736786563,3.73931170021428e-09,1.25354888384077e-06,10.8968598570647

"HTRA1",1.21191370458874,14.448624082,7.44958794114091,4.58467013187187e-09,1.51301167690436e-06,10.7013523487722

"BX470102.1",-1.10872066334199,7.4443262706,-7.44238603652232,4.69037158394767e-09,1.52444183101911e-06,10.6794852982013

"TBX15",-0.772034996350655,9.385529382725,-7.42042629028681,5.02808791973407e-09,1.6098136412868e-06,10.6127805569769

"SH3KBP1",0.912769455135641,8.64401082505833,7.36670779107594,5.96129092790303e-09,1.8267950242064e-06,10.4494263856301

"COL5A1",1.70638446471428,12.502502698675,7.35950258018359,6.09909171586544e-09,1.83167963189262e-06,10.4274967634901

"HERC5",-1.15997270158875,7.895378214475,-7.35698425209521,6.14800864744155e-09,1.83167963189262e-06,10.4198309827276

"MPHOSPH8",-0.873548235238104,8.4919638145,-7.30760367364869,7.19128033848184e-09,2.05680206054365e-06,10.2694077309054

"WASH3P",-1.01561563469697,8.509104040375,-7.29076866001516,7.58634143574048e-09,2.14124487023775e-06,10.2180780784555

"DDA1",0.722856275238092,9.3720974245,7.28241210084877,7.7904922767645e-09,2.17030973803734e-06,10.1925903707528

"C4orf48",1.7021845850606,10.766480902675,7.25553836194028,8.4853122813657e-09,2.30403080693134e-06,10.1105860468402

"SWAP70",-0.751520875051948,8.761866867225,-7.24756718133024,8.70317493578067e-09,2.33364756934289e-06,10.0862509778898

"SYT11",1.88208924293073,9.336733263275,7.23872696400203,8.95138711475503e-09,2.36438054859629e-06,10.059256863566

"ATN1",-0.657726383997119,7.56746492058333,-7.23569199510177,9.03823621205985e-09,2.36438054859629e-06,10.049987966604

"GALK1",0.847341416264069,10.242504827275,7.22588053939323,9.32485292129708e-09,2.40996891584029e-06,10.020018423889

"CLUHP3",-0.643028806268402,7.2330784134,-7.15417160304592,1.17173210282547e-08,2.99224111163205e-06,9.8007511976112

"TGFBR3",-1.11156443372727,10.431273932175,-7.13283928497628,1.2541965764901e-08,3.12834543747549e-06,9.73544654100475

"P3H2",2.47680064059307,10.737469482775,7.12884941671233,1.27025708076331e-08,3.1319867401671e-06,9.72322856871734

"KANSL3",-0.856260943502168,8.380014526325,-7.12438784672863,1.28846172093597e-08,3.14077186088609e-06,9.70956472621688

"PNISR",-1.02026294459741,8.83962480185,-7.08054064868111,1.48196402346252e-08,3.40538595696927e-06,9.57520212706748

"GRIPAP1",-0.650357727385284,7.94887388005,-7.07505615506497,1.50814258501739e-08,3.40538595696927e-06,9.55838598807373

"SERPING1",-1.20574120018183,10.46568425985,-7.06570144075829,1.55387319552852e-08,3.47209728305024e-06,9.52969831666761

"YPEL3",-0.775628617748923,8.6218290225,-7.04530642850896,1.65847243024439e-08,3.66761774238891e-06,9.46713241158834

"ETFDH",-0.767866995025976,8.390670345675,-6.98308249507773,2.02348527183517e-08,4.34057825661361e-06,9.27607023002717

"DMKN",-0.771549919317464,7.96595719699167,-6.9586351685474,2.18812353570098e-08,4.62798148908589e-06,9.20093231141052

"S100A4",2.69209875489176,12.9381579785,6.95685638699486,2.20061587751975e-08,4.62798148908589e-06,9.19546377123273

"OGT",-0.683353808825402,8.19245374476667,-6.89751251688212,2.66119256615368e-08,5.48896555159256e-06,9.01290642227059

"NPHP3",-1.08867873370997,8.867364601975,-6.89322965418736,2.69796406950386e-08,5.51181211951689e-06,8.99972271553827

"LRIG1",-1.15535901127922,8.2871033518375,-6.87294066856427,2.87922592166204e-08,5.82662974014834e-06,8.93725309551009

"WDR19",-0.973410157238098,8.84990398685,-6.80707396064588,3.55649787821412e-08,6.912355702433e-06,8.73428313618049

"MED12",-0.638966251774896,7.906576311,-6.80529529135711,3.57685647741394e-08,6.912355702433e-06,8.72879869128116

"PLCD3",-0.63160882027273,7.288006770275,-6.78907227864674,3.7680369024451e-08,7.1977524448495e-06,8.6787677099438

"GADD45A",-1.089357643171,8.6143902733125,-6.77378027605019,3.9576419211612e-08,7.38220668268077e-06,8.63159477431107

"LIMA1",-0.934883923311693,8.534614134125,-6.75222243647332,4.2413394999476e-08,7.64545996751059e-06,8.56507170003324

"APP",0.94497710125685,9.351218600775,6.74674876107056,4.31656961795767e-08,7.67440532204939e-06,8.54817717528006

"SUPT5H",-0.96178729075325,9.3506891037,-6.72606414415682,4.61316909551319e-08,8.07172137118537e-06,8.48432008359221

"MATN3",2.37186254017749,10.160868894075,6.72501706608325,4.62871640566479e-08,8.07172137118537e-06,8.48108698847953

"HYKK",-0.992074814051947,7.39611474555,-6.72252359840997,4.66595240327717e-08,8.07172137118537e-06,8.47338760955956

"PNPLA2",-1.18379983889178,8.2588179492,-6.71499265018144,4.78025241315183e-08,8.20329556116159e-06,8.45013150706765

"TTC14",-0.703290952036799,7.8302678972125,-6.69559894812485,5.08771720306594e-08,8.66163664467996e-06,8.39022946736997

"FOXJ3",-0.722292394320352,9.2244568034,-6.69010896152423,5.1783090668116e-08,8.68031735468716e-06,8.37326901526849

"CAVIN3",1.11633413336797,10.9999881106,6.69003013351681,5.17962156263091e-08,8.68031735468716e-06,8.37302547793453

"ZNF83",-0.86286841961472,7.771938494675,-6.66379613304783,5.63551724407561e-08,9.37112251183457e-06,8.29195974789098

"SLC6A16",-1.07626435711689,7.28429582795,-6.65639025668571,5.77136496340717e-08,9.51280835581998e-06,8.26906901840339

"PRPF3",-1.03460939307792,9.180310773425,-6.65434722151912,5.80941631911061e-08,9.51280835581998e-06,8.26275378383269

"TMEM94",-0.764260501419916,7.9450568499,-6.64036765062311,6.07663329928757e-08,9.86627358930045e-06,8.21953639276941

"VKORC1",1.07965210177489,13.58560832825,6.63620380615184,6.15858524757457e-08,9.86627358930045e-06,8.20666230609126

"TF",1.22579985236796,9.732641454775,6.63596802677906,6.1632588735549e-08,9.86627358930045e-06,8.20593328266706

"EPDR1",1.32878421890908,8.7037269056,6.6173439064474,6.54390520954388e-08,1.03215669595534e-05,8.14834028828149

"ITGB5",0.656908860777481,9.49557177277,6.58812745991995,7.1891702943424e-08,1.09711460808734e-05,8.05796177780453

"LMNA",1.35641484845887,10.07136418705,6.57310767115695,7.54537560631798e-08,1.1398299445854e-05,8.01148563606428

"CISH",-0.773825765367969,7.213724279,-6.56974023979112,7.62763775979359e-08,1.14419900409323e-05,8.001064455214

"DDX51",-0.876376114025978,9.166830474,-6.56119616742069,7.84042156481683e-08,1.16238092217227e-05,7.97462112142436

"DMTF1",-0.587686160231605,8.0162435692375,-6.5597350446714,7.87740180955497e-08,1.16238092217227e-05,7.97009875678926

"NAPB",-0.644860716865804,7.8645819993,-6.55062324857539,8.11199962593346e-08,1.18058358532802e-05,7.94189469379149

"THBS3",0.868553841432897,8.049717329325,6.53716415431195,8.47141661012352e-08,1.19571820257363e-05,7.90022847370832

"RFTN2",0.742162358909082,10.1912676921,6.53608264897482,8.50098209430496e-08,1.19571820257363e-05,7.89688008875465

"S100A10",1.21277039796536,13.58477458975,6.53507937700003,8.52850146817233e-08,1.19571820257363e-05,7.89377387847343

"KLF15",-0.665992306363636,7.61550863825,-6.52790112810477,8.72802387611917e-08,1.21574571536774e-05,7.87154835637059

"SRGN",1.4169383333961,10.3353354433375,6.51905114954146,8.98047262082424e-08,1.24283947218904e-05,7.84414416988972

"OSTC",0.697977252088744,9.4490999871875,6.50219054884943,9.48188417994241e-08,1.30381985605093e-05,7.79192710763988

"ELL2",-1.15720743338961,8.276969065025,-6.46925250380977,1.05439154182597e-07,1.42864746748389e-05,7.68989015692511

"IGFBP7",2.15214645487012,11.222130693125,6.46791489437963,1.05894805524189e-07,1.42864746748389e-05,7.6857456898475

"FSCN1",0.837153025294368,11.203752725725,6.4514532751693,1.11666807840351e-07,1.4786201820885e-05,7.63473611082626

"SRPX2",1.64106249216016,10.743565773175,6.43226216362806,1.18795519081318e-07,1.54173319887034e-05,7.57525805862188

"THOC1",-0.673536099727276,7.753012920725,-6.43092720485413,1.193080560405e-07,1.54173319887034e-05,7.57112027586282

"LMOD3",-1.50128155318616,8.46654368505,-6.41723420090992,1.24695043686044e-07,1.59538572699511e-05,7.52867500662338

"MRPL17",1.0972425673593,9.9446240135,6.41660722262344,1.24947462652174e-07,1.59538572699511e-05,7.52673138403485

"FMC1",-0.60161279112338,8.2111968880375,-6.40983125083305,1.27708353625692e-07,1.62098928616848e-05,7.50572527213656

"SPP1",3.39793315137662,11.8806509251,6.40093622379178,1.3142574358487e-07,1.65836095625827e-05,7.47814796186927

"SERPINA5",1.82379676209957,12.396708980875,6.37515869621783,1.42822906456982e-07,1.71846578101222e-05,7.39821776643995

"ADHFE1",-0.783525609445893,7.70737714785,-6.37467988055106,1.43043727685732e-07,1.71846578101222e-05,7.3967329064922

"HNRNPU",-0.81402084985498,8.2930379630125,-6.37391090088502,1.43399083866108e-07,1.71846578101222e-05,7.39434820406461

"NDUFA8",0.905803988727266,10.3738649237,6.35944157260314,1.50253030472518e-07,1.76124467577377e-05,7.34947427492644

"PRRC2C",-0.906771880956716,9.106896250925,-6.34689418906069,1.56461909325451e-07,1.80444323491411e-05,7.31055683133545

"BTG2",-2.01186242311255,10.078585728075,-6.34084542912113,1.59546277586345e-07,1.83017497353192e-05,7.29179446575888

"MOAP1",-0.728864151536797,7.747223015125,-6.33597570866729,1.62073661061101e-07,1.84686262983207e-05,7.27668870921442

"TMED9",0.954803684506491,11.213151141575,6.28351996918293,1.91978749028945e-07,2.11186468995892e-05,7.11394096279477

"DDX23",-0.641101088649352,8.49227781615,-6.25926045263977,2.07621510849433e-07,2.24395988605218e-05,7.03865673119739

"ORC3",-0.596933711341994,7.600481807,-6.25688939817133,2.09217275283407e-07,2.24395988605218e-05,7.03129815034416

"FAP",1.58580993774891,10.4244613585,6.23990610760056,2.21012266232302e-07,2.31233658263524e-05,6.97858790680935

"THY1",1.85953047207792,9.11455114675,6.22589551662493,2.31242492643191e-07,2.38895339401583e-05,6.93510078261587

"MBTD1",-1.32508996842857,7.824780027475,-6.2223423034729,2.33911513858303e-07,2.38895339401583e-05,6.92407163611361

"TLE2",-0.996281834008665,7.7786427438,-6.22164551810443,2.34438516306428e-07,2.38895339401583e-05,6.92190880063142

"MGRN1",-1.1366503669394,8.148195434275,-6.22092288398126,2.34986325177073e-07,2.38895339401583e-05,6.91966572359115

"PI4KAP2",-0.804146574034631,8.32741361685,-6.21849667112907,2.36834964573023e-07,2.39639001181883e-05,6.91213464121081

"WSB1",-0.874547444591634,8.22545566578333,-6.20702441907818,2.45775380158977e-07,2.45391399568644e-05,6.87652324303447

"ZNF512",-0.715141489688316,8.60802919515,-6.20680208967908,2.45951941202081e-07,2.45391399568644e-05,6.87583308764995

"RELA",-0.728041140852819,8.099700594225,-6.20122779676657,2.50420473864702e-07,2.48693036336654e-05,6.85852917345019

"MINDY1",-1.06430492996104,8.329403419925,-6.18371752556982,2.64992950710703e-07,2.61290720719122e-05,6.80417103037335

"XRCC2",-0.837814678616888,8.0130589322125,-6.17793389547129,2.69990407401653e-07,2.64454987633464e-05,6.78621591349453

"SLITRK4",-1.16818382015152,7.718934685375,-6.15726436223392,2.88634074596423e-07,2.81431342462176e-05,6.72204559128625

"MAATS1",-0.778679216000001,7.6341841598,-6.15424345794809,2.91464784084903e-07,2.82905478887116e-05,6.71266666163592

"TMED3",1.01900643290043,11.91150231,6.15207848441784,2.93510530437543e-07,2.83099129296242e-05,6.70594508079604

"KANSL1",-0.600377230413429,8.4431801588375,-6.1504666238509,2.95042952748444e-07,2.83099129296242e-05,6.70094072601789

"OMD",1.51898291816449,11.4457021072,6.14985813078322,2.95623537188747e-07,2.83099129296242e-05,6.69905152885536

"CENPT",-1.17711413615152,8.530406972675,-6.1242802054675,3.21091062764129e-07,3.03423981821732e-05,6.61963742809716

"TMEM17",-0.943877201664504,9.2265465724125,-6.11776854413213,3.27918056185054e-07,3.08336293986063e-05,6.59941957552923

"TANC1",-0.617330002095239,7.9604126847,-6.11659461994562,3.29164194316389e-07,3.08336293986063e-05,6.59577467770262

"ZNF500",-0.670575894515157,7.781980019025,-6.11443714125427,3.31466761344306e-07,3.09143195547683e-05,6.58907594481346

"PACS1",-0.927564192000002,7.9260196586,-6.10528467549397,3.41415304738239e-07,3.15676711290516e-05,6.56065838323284

"PFKP",0.997078176493503,11.45711018275,6.09168404060683,3.5675348469602e-07,3.27039273513433e-05,6.518429282243

"SAMD4B",-0.748859694134201,7.730986575625,-6.08389686829546,3.65843940346561e-07,3.29999315918163e-05,6.49425047916875

"CCDC14",-0.81341015998269,8.3449676173,-6.07896919458592,3.71715677327445e-07,3.32236374764626e-05,6.47895025972963

"GTF3C5",-0.633672970337667,8.2824790429,-6.06489290212844,3.89013267927739e-07,3.44823289682559e-05,6.43524396601508

"ZNF69",-0.832813744932906,8.4082474747875,-6.05553822847801,4.00951812745439e-07,3.52492513737804e-05,6.40619819454905

"IRF3",-0.917232677190478,8.132735973175,-6.04324658479902,4.17197466952985e-07,3.60359464584378e-05,6.36803354462014

"CRLF1",2.48214977745887,10.412198687975,6.03126941065604,4.33660577599871e-07,3.67685891308096e-05,6.33084573191396

"ARGLU1",-0.753469623887451,9.90822668715,-6.01887358392692,4.51383619682087e-07,3.81205906527577e-05,6.29235866790688

"COA8",-0.800796409558442,8.7593285254,-6.01025273409992,4.64134632674084e-07,3.90437333548697e-05,6.26559276808038

"GHDC",-0.931940518619053,8.509576110425,-6.00091286387318,4.78355936620988e-07,3.97721441723132e-05,6.23659497096739

"COLGALT1",0.776983848675322,10.2802673183,5.99414788640828,4.88927902405469e-07,4.04941792837827e-05,6.21559189823792

"HSD17B14",-0.587921327086586,7.930285871725,-5.98449929660698,5.04411667534147e-07,4.12982239705152e-05,6.18563674311563

"CFL1",1.09494279185714,10.298667569425,5.98115570536124,5.0989095590002e-07,4.15881022624004e-05,6.1752563591216

"FXYD6",1.12004270172293,10.73839889235,5.97596772694756,5.18510741942276e-07,4.20065638607402e-05,6.15915015712605

"CYBRD1",-0.797024496103898,11.5271204,-5.9727051536057,5.24005933973731e-07,4.2257335675453e-05,6.1490215474247

"ITCH-IT1",-1.41535535519914,8.463393958675,-5.96674454457141,5.34196232575894e-07,4.27136047344301e-05,6.13051719155214

"CHST6",1.26168982935064,9.8876493545,5.96288407909641,5.40901561852388e-07,4.29736274196132e-05,6.11853279685623

"TGOLN2",0.613676835116875,10.1065399154,5.95058486968808,5.62830431160136e-07,4.4224452669656e-05,6.08035230894697

"RETREG2",-0.661541114203463,8.086259598925,-5.94291344876995,5.7695586656461e-07,4.51689061813046e-05,6.05653876604647

"HCFC1R1",0.833978922891768,8.9774706006,5.9382303075871,5.85752556028796e-07,4.55252828962815e-05,6.04200176486288

"ZNF444",-0.649334851649351,7.747997027675,-5.92963167880126,6.02254437252715e-07,4.63045158907096e-05,6.01531138657748

"FAXDC2",-0.682216779500003,8.1382787354125,-5.89871567980521,6.65518189550488e-07,5.0445338106175e-05,5.91935607062292

"CREBRF",-0.759465742906935,8.1543449808875,-5.89032969586428,6.83795233762888e-07,5.11083329597481e-05,5.89333075807512

"KDELR3",0.766554503841268,9.05694254105,5.88657305560337,6.92144541588397e-07,5.13743687253034e-05,5.88167268420286

"CBX7",-0.984823461515155,9.44607840825,-5.87606140198764,7.16052272847974e-07,5.27015364464491e-05,5.84905296773492

"EPB41L4A-AS1",-0.804291505021654,10.8826587955,-5.86873536876208,7.33200685189165e-07,5.34962173401115e-05,5.82632008209997

"OGFR",-0.693525112337671,8.20016770875,-5.86154889460568,7.50420878821604e-07,5.45670449884821e-05,5.80402127514645

"METTL21A",-0.78172649671429,8.470926663425,-5.85582063787483,7.64435728101194e-07,5.50988435324724e-05,5.78624790041248

"HLA-A",-0.90548516074243,11.8715250623875,-5.85491867729873,7.66666175462985e-07,5.50988435324724e-05,5.78344940014638

"UGGT2",0.700575047155841,9.008887810475,5.84614543476643,7.88703352828013e-07,5.62075602043644e-05,5.7562296873758

"IFNGR2",0.629232629987008,11.029983957025,5.83945117005305,8.0594291466102e-07,5.70570345293516e-05,5.73546130454251

"DLL1",-0.606410988441561,7.66594224725,-5.83786813844332,8.10074360053131e-07,5.7160872031249e-05,5.7305502311538

"ZNF385B",-1.03806136555845,8.7777021277,-5.83030859162059,8.30096988214133e-07,5.8190883967913e-05,5.70709887609827

"CILP",1.16338157575757,14.46815727,5.82076293247766,8.56087540757271e-07,5.94302065915347e-05,5.67748809055642

"TDRD1",-0.847075483376625,8.0041381365,-5.81612253279956,8.69014150914409e-07,5.98037916206325e-05,5.66309429599178

"GRAMD2B",-0.586984583627709,7.99013338265,-5.81583098519822,8.69832781018942e-07,5.98037916206325e-05,5.66218997869237

"RBL2",-0.70620044414286,9.129949099725,-5.81107554964328,8.83294675039213e-07,6.05353165312018e-05,5.64743995201572

"SAT2",-0.758904574060607,8.0019201104,-5.80968247529775,8.87277495327605e-07,6.0614616408511e-05,5.64311913464114

"TP53I13",1.09224164897403,9.338737044975,5.78902524237834,9.48484819005282e-07,6.35810870390072e-05,5.57905376980786

"SOD3",-1.9207200000736,11.411564924225,-5.78680026603881,9.55323821192633e-07,6.3840035166365e-05,5.5721540216398

"ARHGAP32",-0.671975832281389,7.880813097225,-5.78300545149517,9.67101844586636e-07,6.42868593426677e-05,5.56038645628236

"SLC25A23",0.820714688826833,9.146444611425,5.77865033341609,9.80797593639746e-07,6.49354604356981e-05,5.5468819147436

"CHRNA5",-1.56263238547187,9.8636012477,-5.76931122656409,1.01082280591738e-06,6.67174154145653e-05,5.51792464997101

"HPS6",0.810761215467529,8.959597007475,5.76694573812305,1.01857233752198e-06,6.7022684699951e-05,5.51059051534761

"GALNT16",0.890073189753245,8.603752380975,5.74672318483439,1.0872898809348e-06,7.08919612034417e-05,5.44789800094605

"NCOR2",-0.760074084354983,9.02183643521667,-5.73030036430648,1.14648458143433e-06,7.42998210161566e-05,5.3969947501103

"LINC01554",-1.79096911594805,7.6658408942,-5.72757235700851,1.15662422102222e-06,7.4731163148035e-05,5.38854004171593

"CTIF",-0.645889220047623,8.143414342175,-5.71157676338881,1.21790351404408e-06,7.79858157604766e-05,5.33897126818094

"CIC",-0.800423452125545,8.045600385425,-5.70636609801757,1.23855670182051e-06,7.88376255511919e-05,5.32282588209725

"POLM",-0.668377458103899,7.89062256535,-5.70211230120695,1.25567591275002e-06,7.92220706011782e-05,5.30964610896305

"TYMP",0.998743140006491,8.9829717108625,5.69306723657981,1.29286575050397e-06,8.08565940847629e-05,5.28162350762284

"CEP19",-0.766775683906934,9.1941693235625,-5.69081760874969,1.30228467383115e-06,8.12072922626509e-05,5.27465439027038

"TSPAN2",0.690737571238093,7.8427855337,5.67811630067776,1.3567617778442e-06,8.31872267189908e-05,5.23531067462489

"NPIPB15",-1.04610925605195,8.2912730879,-5.6779924413233,1.35730405816264e-06,8.31872267189908e-05,5.23492703736512

"ALKBH5",0.59755406376622,12.21584340975,5.66700728979994,1.40626878116911e-06,8.59426542018761e-05,5.20090447062989

"TMEM50B",-0.596996401268399,8.207233852025,-5.66168551578406,1.43061915212739e-06,8.69354431509478e-05,5.18442393408536

"BEST1",-0.729223429731603,7.4035697739,-5.65740172567684,1.45052512374904e-06,8.7648491350819e-05,5.17115868404477

"DNASE2",0.935011212831169,9.7651490773,5.64863573834193,1.49212375436688e-06,8.99088389183255e-05,5.14401618037857

"PAN3",-0.885074892575762,8.704106523625,-5.64716013603386,1.49924218258509e-06,9.00847172510721e-05,5.13944752879594

"UBALD1",-0.854489220761908,7.7575846423,-5.64004001752336,1.53406884302973e-06,9.14091965328632e-05,5.11740405285246

"GSE1",-0.867176727757575,7.8104553516,-5.63156360454211,1.57658240799809e-06,9.34233956739423e-05,5.09116444376213

"MAN2C1",-0.98397755267966,8.998557627325,-5.62857686729267,1.59184053790824e-06,9.40676897484013e-05,5.08191944760793

"KIAA0355",-0.665831203008661,9.632303348375,-5.62447327566433,1.61304454988232e-06,9.48268570788953e-05,5.0692180478169

"VAT1",-0.678253295662344,8.44305266565,-5.61895618878445,1.64199662447441e-06,9.56898804344798e-05,5.05214281050081

"RHOT2",-0.677240302904767,8.605059222675,-5.61823161130035,1.64583727073572e-06,9.56898804344798e-05,5.04990036556447

"NT5C3A",-0.640776533718618,8.664267873825,-5.61819023291265,1.64605686822633e-06,9.56898804344798e-05,5.04977230716951

"NUP160",-0.626348755787884,8.420407311475,-5.61556517298179,1.66004806082854e-06,9.62424079806297e-05,5.04164839697717

"BTAF1",-0.837017515683987,8.753436277275,-5.61358117036909,1.6707010850184e-06,9.65989460235304e-05,5.0355086113069

"ZC3H12A",-0.892889137943724,7.597164854625,-5.60086657318242,1.74060743343824e-06,9.98336097718817e-05,4.99616580507936

"OCIAD1",-0.685149640595247,10.1899515329375,-5.59871934391825,1.75269752617859e-06,0.000100258972357485,4.98952238505726

"NSMCE1",0.628202479060604,8.938425570225,5.5941154248497,1.77890290573944e-06,0.000101087625987952,4.97527883736373

"PPP1R14C",1.18226551570129,8.811111749025,5.59368936757296,1.78134765724908e-06,0.000101087625987952,4.97396075849268

"TNNT3",1.42876329453896,8.7872067626375,5.58519159122237,1.8308138305202e-06,0.000103078182358239,4.94767333118772

"COPB2",0.712965502004325,10.096463028725,5.5793226181408,1.86577414694138e-06,0.000104771521534135,4.92952005384393

"EPM2AIP1",-0.731802573982687,9.19757758575,-5.57578681243166,1.88715643609314e-06,0.000105695542325415,4.91858431244252

"GRINA",-0.85742952231602,8.935250578875,-5.57131102560867,1.91457352987636e-06,0.0001066740695828,4.90474225162113

"SLC5A8",-0.77165824641126,7.494198386425,-5.55910060123091,1.99140460523257e-06,0.000109813933642272,4.86698486679328

"SBNO2",-0.885038582471863,7.718242620175,-5.55470181622455,2.01982887256193e-06,0.000110811634642777,4.85338473817676

"MBD4",-1.09744608199568,9.384358863925,-5.54471380788234,2.08587908470275e-06,0.000113563939710555,4.82250771460642

"CALU",0.855095371688314,10.2179082835,5.52959805632592,2.18995152476402e-06,0.000118627904438669,4.77578904101614

"SULF1",1.63566054839393,10.062601853425,5.52035132754764,2.25614775846212e-06,0.000121294800919225,4.74721618380503

"AHDC1",-0.651985321329009,7.705151153475,-5.51845062077844,2.26999956426256e-06,0.000121686228352199,4.74134350409534

"HDAC4-AS1",-0.70377187470996,7.55235514665,-5.51779815759834,2.27477402308665e-06,0.000121686228352199,4.73932761348851

"NKX3-2",1.12485655215584,10.8104475161,5.49964045174766,2.41173075353918e-06,0.000127935027263345,4.68323632505804

"FRMD6-AS1",-0.60643897423377,7.4405152864,-5.49916258623225,2.41544385071347e-06,0.000127935027263345,4.68176040328298

"TBC1D3L",-0.601096119082254,7.5151815879,-5.49816330053518,2.42322687344352e-06,0.000128031132172997,4.67867408185588

"CNOT3",-0.602726484034634,7.8416932941,-5.49160395787912,2.4749382377355e-06,0.000130442015080256,4.6584168507935

"NUP214",-0.66854693119048,8.411930546625,-5.47821749006064,2.58390465618829e-06,0.000134725675755312,4.61708347207752

"TBC1D17",-0.662496183978364,7.634607581075,-5.47318822418341,2.62606569235116e-06,0.000135179721430772,4.60155746122411

"BFSP1",0.785674537164499,7.989717889875,5.47259592218116,2.6310758864419e-06,0.000135179721430772,4.59972904995353

"ACIN1",-0.651201451489186,8.71516974695,-5.46864715513555,2.66472187858693e-06,0.000136422312691094,4.58753993397139

"EID2B",-0.730164179619052,8.6077043021,-5.46500646344994,2.69612206853193e-06,0.000137701224981139,4.57630265894126

"COL15A1",1.40619231422943,11.160598606525,5.46259357034796,2.717135345872e-06,0.000138444822575535,4.56885554102039

"UBAC2",0.936439714155842,8.76753209775,5.46100932933073,2.73102064200418e-06,0.000138822568226615,4.56396616412691

"PLBD1",0.870744239060599,9.610168287225,5.46022676446624,2.73790556701604e-06,0.000138843527938679,4.56155102593942

"ZNF14",-0.772891220887452,8.511082869125,-5.43783500818483,2.94239287642199e-06,0.000147860571489014,4.4924627774868

"S100A6",0.827347330822509,11.6603475365,5.41494076527108,3.16717928232391e-06,0.000157266580521135,4.42185866010086

"MYL5",-0.632667597216455,7.799607876725,-5.4130956027836,3.18602278950398e-06,0.000157836893435681,4.41616988090449

"S100A3",0.67191264034199,7.966181630425,5.41174315206595,3.19990530777062e-06,0.000157947567586955,4.41200032064636

"KBTBD11",-1.04607326882684,8.046509637075,-5.41110041283746,3.20652391304697e-06,0.000157947567586955,4.41001882180317

"ZNF549",-0.825030113259745,9.428697245775,-5.39920060096643,3.33155102884726e-06,0.000162790663143058,4.37333816784924

"HOXB2",0.932270977532466,8.24876191075,5.39175533219221,3.41222887281824e-06,0.000165554253886432,4.35039356473374

"TMCO1",0.628663648073591,10.596753347375,5.38654503757269,3.46984074725426e-06,0.000166886892083747,4.33433901146666

"TPI1",0.861205977012986,12.21292996675,5.3766560905031,3.58185856812381e-06,0.000171889145737861,4.3038735062158

"EXO5",-1.27248559735498,8.295734906325,-5.37334684784048,3.62014135510868e-06,0.000172952454807208,4.29368012185235

"GFRA2",0.79303733696536,8.263614021925,5.37098383484756,3.64772639190841e-06,0.00017388306407295,4.28640188508897

"DIO2",1.302711515329,9.351306486575,5.36657847239536,3.69971220291009e-06,0.000175216005690831,4.27283419053093

"CDKN2AIPNL",-0.724674486891779,9.4460954526,-5.36530308175128,3.71489954670786e-06,0.000175216005690831,4.26890649714468

"FOXD2",-0.821078384060609,9.60916070215,-5.36516647856897,3.71652988622107e-06,0.000175216005690831,4.26848582120702

"COL1A1",3.56749110274026,11.626689935475,5.36203746439414,3.75406956250104e-06,0.000176211260799146,4.25885026020133

"SLC38A3",-0.882680476506498,7.928732564025,-5.35079109550903,3.89213386017154e-06,0.00018032432707244,4.22422407227343

"SHROOM4",-0.753199700021645,8.261721645125,-5.35005969469002,3.90128557814926e-06,0.000180358786501896,4.2219725149407

"PLPP3",-0.821787454883124,8.13544310481667,-5.34603244899351,3.95206125295238e-06,0.000181645945482564,4.20957571107389

"MAP7D1",0.899225026718609,9.6171058819,5.34597485245159,3.95279217565117e-06,0.000181645945482564,4.20939842457336

"SNRPA1",-0.669105059484853,9.484580495925,-5.3400447773357,4.02877059162457e-06,0.00018387480417221,4.19114659010483

"DYSF",1.44857599489177,9.3303066515,5.32982674137448,4.1631078448517e-06,0.000188402587299396,4.15970357634815

"FOPNL",-0.62614228305195,9.012359053625,-5.32372756129674,4.24540577170716e-06,0.000189330975486258,4.14093909393782

"ZNF324",-0.717387671800869,8.09945795605,-5.31666877325312,4.34267163569266e-06,0.000193266907172704,4.11922606105161

"MYO5C",-0.923669231432907,7.816175182925,-5.3121474778702,4.40613225482793e-06,0.000195280873963459,4.10532055492626

"SMAP2",0.693301898398268,8.84369009075,5.29690044215501,4.62700740419298e-06,0.000203453737692064,4.05843982677491

"DYNC1I1",-0.915591298341997,7.939160588725,-5.29643354254243,4.63394190224421e-06,0.000203453737692064,4.05700453539352

"NCOA7",-0.667061108034636,8.2382100983,-5.29616332854312,4.63795989610831e-06,0.000203453737692064,4.05617388162345

"CDK6",0.973176644705623,8.124952474025,5.29482249414264,4.65794891042165e-06,0.000203913596076438,4.05205216830476

"MAPKAPK3",0.930224413584412,9.05638028435,5.29006924056902,4.72950169254808e-06,0.000205369515803338,4.03744192824744

"MSRB2",0.920305124238089,10.653502639925,5.28443479615521,4.81573457759842e-06,0.000208691560452654,4.02012562134766

"PITX1",-1.2731541211645,10.042515434325,-5.28158532224934,4.8599373149868e-06,0.000210182490612463,4.01136937999251

"TTC19",-0.640943068683987,8.87256837105,-5.27686813236832,4.93400049301083e-06,0.000212528603565412,3.99687530556144

"KHNYN",-0.772652977861474,8.32767126255,-5.27465131870163,4.96919208675272e-06,0.000213028027351423,3.9900645696604

"RERG",-1.09603377141126,9.5748753383,-5.27426265433495,4.9753877070096e-06,0.000213028027351423,3.98887051658701

"AKNA",-0.686307910238098,7.652360092625,-5.27023489338433,5.04004679762974e-06,0.00021451199177769,3.97649721849652

"ELP2",-0.59987946938962,8.947016861325,-5.26239486977503,5.16830765650481e-06,0.000218669363983599,3.95241669924962

"ANOS1",1.21950527026407,9.063672316825,5.25355368662472,5.31683860026441e-06,0.000224069754055544,3.92526764174598

"ALPK1",-0.659355682865802,7.80900998435,-5.25013747877461,5.37535890486739e-06,0.000225649361777515,3.91477918964178

"MIB2",-0.610522194562778,8.5905192312,-5.24585332927597,5.44965172576824e-06,0.000226991221688261,3.90162746208722

"CAPRIN2",-0.641022343103897,7.794974143725,-5.24030142168633,5.54744738166595e-06,0.000229726435876672,3.88458636640124

"TMEM43",0.638544250987012,10.94672530335,5.23759685588627,5.59571676015868e-06,0.000231278844358697,3.8762859530934

"EMP3",0.685424066666654,12.338321555,5.23296269061774,5.67939545852613e-06,0.00023382656319318,3.86206508280524

"GLA",0.625923160294368,8.7258467161,5.23237698663778,5.69005948379284e-06,0.00023382656319318,3.86026787245475

"BLZF1",-0.93055296009741,8.6970565870625,-5.22282851557084,5.86673980609912e-06,0.000237441561921609,3.83097322664812

"CEP95",-0.72406742374026,8.2898642037,-5.22224828151992,5.87764996412168e-06,0.000237441561921609,3.82919334551476

"PPIC",1.27467900155195,10.2864011666375,5.21003917917612,6.11194475404943e-06,0.000243693916206532,3.791748977655

"IFT122",-0.602109522627711,7.62718684464167,-5.20624884462005,6.18654982046202e-06,0.000245980512731063,3.78012718516837

"DBNL",0.58983932401082,9.2159120097375,5.18857607646074,6.54650545845101e-06,0.000257196132947313,3.72595777870483

"VAMP2",-0.98436891887013,8.808289050075,-5.18716887399565,6.57604452319994e-06,0.000257884334674885,3.72164581758477

"INO80E",-0.765259435930743,8.5965715265,-5.18273897129487,6.66990009604055e-06,0.000260612070965694,3.708072942566

"LPIN1",-0.684681881796539,8.890818300375,-5.1744883744586,6.84825990791671e-06,0.000266609842621999,3.68279892462372

"CCDC84",-0.895095089268402,8.204007545925,-5.17190425993903,6.90508968621625e-06,0.000267836266637384,3.67488440924586

"KAT6A",-0.814447689272733,8.64058935635,-5.14953290717565,7.41704279873899e-06,0.000281100680345848,3.6063945446007

"XAB2",-0.866021844731605,8.535981808525,-5.14258110601299,7.58366627738419e-06,0.000286481753164545,3.58512204058442

"TMCO3",0.720449264186144,10.767740864525,5.1424956847521,7.58573659957399e-06,0.000286481753164545,3.58486068275514

"UAP1L1",-0.599356360627711,7.824046574625,-5.12723989073018,7.96461625760527e-06,0.000297646312442318,3.53819576951327

"HSBP1",0.830632235424243,10.219839447225,5.1241699544109,8.04309596258284e-06,0.000298793774857362,3.52880831481157

"NFIB",-0.952226621281392,9.5561804068,-5.123319722798,8.06496646507915e-06,0.000298793774857362,3.52620859735373

"ANXA2",0.688879676743143,10.0013893814083,5.11756229136256,8.21462390606839e-06,0.00030381361622254,3.50860637107059

"DEPP1",-1.65119004606927,9.90187530285,-5.11005169776512,8.41399711822217e-06,0.000310117959077292,3.48564954110009

"MDC1",-0.621139872541128,7.854076183725,-5.09948260930102,8.70270710034531e-06,0.000319975595283892,3.4533544950651

"PNN",-0.775590243134202,8.3027489807,-5.09917397675106,8.71128374648234e-06,0.000319975595283892,3.4524116167694

"ELOVL4",0.639652314190474,8.88505989335,5.09740789236258,8.76052312948563e-06,0.000321234156667686,3.44701639666311

"RCOR3",-0.83709174048918,9.106632644525,-5.09263460406748,8.89498927307181e-06,0.000325403774647154,3.43243617309607

"MTND5P10",-1.23117085225109,11.81273880975,-5.0896388872255,8.98042269572812e-06,0.000326506859739091,3.42328690809139

"STAT5A",-0.678512551333336,8.55430235015,-5.08609044155194,9.08267154465202e-06,0.000329108762338396,3.41245083522652

"PRDX1",0.771649756337663,11.17528002355,5.0815671326821,9.21468504328902e-06,0.000332768028389887,3.3986398091093

"TBC1D32",-0.695867652034636,8.2075663335,-5.06960650042979,9.57298742377959e-06,0.00034282162475375,3.36213148236938

"PTH1R",1.29636057550216,10.556371380075,5.05443335449537,1.00474454724901e-05,0.000356834027864876,3.31584081934054

"MKNK2",-0.614836906922079,8.278912213075,-5.05243617008392,1.01116065555349e-05,0.000357993349083587,3.30974972947487

"SERPINE2",1.81439914376622,13.74103979075,5.0462708015621,1.03122445341217e-05,0.00036382887746948,3.29094927951777

"NGEF",1.2550617401645,9.56288444635,5.04402560315184,1.03862845413679e-05,0.000365839391948906,3.28410395663643

"SPOCK1",1.47357398469697,10.216417505375,5.04231707620488,1.04429792039542e-05,0.000367233355580362,3.27889527268052

"CWF19L1",-0.656819189597405,9.127252804725,-5.03812040358777,1.05835430719744e-05,0.00037156723803097,3.26610258618887

"RCAN2",-1.30558881750217,8.052891393275,-5.02846447237266,1.09141139266721e-05,0.000381922769724378,3.2366764015477

"CSPG4",0.956096236515151,10.472116585125,5.02574055352868,1.10092020941805e-05,0.000383997388816694,3.22837734350774

"COX17",0.898572563523796,11.23537439805,5.02006762223665,1.12098768173043e-05,0.000389729445069683,3.21109628580621

"BAALC",0.936699738898267,8.0232826486625,5.01624365319353,1.13471850691234e-05,0.000392245909874287,3.19944978366146

"WDR59",-0.851202824320351,9.10314956665,-5.01551086856727,1.13736868184144e-05,0.000392245909874287,3.19721817441038

"CTHRC1",1.00184695293938,9.086813547175,5.00538836133043,1.1746105853385e-05,0.000401219294046117,3.16639796132557

"OSTCP2",0.9744306357619,10.434902749075,5.00174130386015,1.18832222977978e-05,0.000403973061030206,3.15529676249714

"YWHAQ",0.616403561155831,11.427308459525,4.99896539008991,1.19886475099764e-05,0.000406269317119279,3.14684831756106

"CHCHD2P9",0.764483097623373,9.917083774825,4.98218356705492,1.2645998437031e-05,0.00042500012186694,3.09579353518054

"STING1",-1.03001619012121,9.51728215115,-4.97928620557732,1.27630455556833e-05,0.000425346869068503,3.08698256318551

"RAB3IL1",-1.21966335770563,9.14269864575,-4.97912164325847,1.27697255922855e-05,0.000425346869068503,3.08648215556152

"PDE1B",-0.597523626683985,7.4338484687,-4.97679296855176,1.28646245549189e-05,0.000427843505934211,3.07940139663499

"ZNF395",-1.0608169822684,10.7404858082,-4.97457470027882,1.29556730108455e-05,0.000430204553801312,3.07265698803199

"GBP2",-1.08524493385715,10.615212122425,-4.97216606997945,1.30552568950474e-05,0.000432173326629109,3.06533451236008

"EFEMP1",0.657451156102809,8.84233502885625,4.96679783473381,1.32799374342928e-05,0.000436758327008563,3.04901720698014

"DICER1-AS1",-0.678740493311691,8.082838380875,-4.96628304045534,1.33016834433445e-05,0.000436758327008563,3.04745263044183

"CLIC3",1.1607496808658,8.34112618,4.96189388586026,1.34885269870504e-05,0.000440554091583748,3.03411437726436

"GLTP",0.650302109740256,11.39680024625,4.96009802523906,1.35657219725242e-05,0.000441560510380601,3.02865763082723

"RHOBTB3",-1.06190878596537,9.83464923515,-4.95963157123135,1.35858438698055e-05,0.000441560510380601,3.02724037223988

"ID2",1.2780894272987,10.52584706945,4.95577394414467,1.37533898979751e-05,0.000446329752952289,3.01552056380875

"GLB1",0.721669972705621,9.574480706125,4.95233067080432,1.39046648704838e-05,0.000449227631657018,3.00506122973805

"TNKS1BP1",-0.701336615043294,8.381636483875,-4.94874995772094,1.40637236713061e-05,0.000452599573515576,2.99418604559865

"SBSPON",1.81264558194372,10.689247169675,4.94857940410834,1.40713445665477e-05,0.000452599573515576,2.99366808947253

"YRDC",-0.627282396841997,8.6838063239625,-4.93767299354505,1.45672549866008e-05,0.000463931532475723,2.96055422731984

"GSDMB",-0.748256562373741,7.91073144604167,-4.9374637568039,1.45769359418506e-05,0.000463931532475723,2.95991909960457

"CHSY3",0.947016918095231,8.918502334,4.93612492945709,1.46390315108123e-05,0.000465217577686571,2.95585529313442

"TXNIP",-1.1704191652381,8.82960188525,-4.93560240578503,1.46633373571595e-05,0.000465300665160397,2.95426931660922

"PGGHG",-0.975434660458879,8.13975589155,-4.92986087123609,1.49330545968421e-05,0.000471071991407146,2.93684486286444

"GAS8",-0.602926229497839,7.70106867195,-4.92873441131945,1.49865445306829e-05,0.000472065149379851,2.93342678929307

"CATSPER2",-1.14952352371429,10.60568264865,-4.92168012153042,1.53258516775316e-05,0.000480635737331476,2.91202547214603

"POLR2A",-1.01093358119481,9.9520723298,-4.91032339043014,1.58881272951673e-05,0.000493221734600049,2.87758550890253

"MED20",0.606509886030302,8.567441796975,4.90874245305544,1.59680000461741e-05,0.000494984926286823,2.87279261220489

"PDK4",-1.2786168028052,7.7188384964,-4.90592083630187,1.61115424897697e-05,0.000497917414704281,2.86423922124934

"PAPSS2",1.26516101298268,11.764169622425,4.90551610626125,1.61322364094669e-05,0.000497917414704281,2.86301242032875

"PXDN",0.636445353999997,8.12671325905,4.90224224292746,1.63005982790762e-05,0.000501062454974501,2.85308964789773

"BCYRN1",-0.668411855242426,8.094431351425,-4.89666791867836,1.65912688163794e-05,0.000506257905234927,2.8361977899602

"TMEM14A",0.68877045132467,9.1961373632,4.89563378768883,1.66457536835407e-05,0.000507198952081864,2.83306453807583

"CMAHP",-0.618255874671,8.232401284825,-4.89275170863177,1.67985356075388e-05,0.000511128208960731,2.82433308079342

"MAB21L2",-0.991699351424248,7.860255990075,-4.89213792308537,1.6831251563788e-05,0.00051139826812297,2.82247372412042

"DHRS3",-0.857269934069272,9.69968987425,-4.88838081721379,1.70328894078236e-05,0.000516062868202293,2.8110933677076

"FMOD",0.897620879696962,13.12683968575,4.88163613281585,1.7400872690683e-05,0.000525726929701185,2.79066851973416

"NDUFB2",0.802056991398263,11.021626033475,4.88092395110644,1.74401842019469e-05,0.000525907215327125,2.78851220085001

"HERC1",-0.623950901285717,8.566174836725,-4.85834165386902,1.87332441739052e-05,0.000558896830006177,2.72017560359835

"NOP10",0.786540769458872,10.927286898375,4.85587123274039,1.88803232684331e-05,0.000562502520043277,2.71270425558889

"CNMD",1.73100290268614,9.8563152837875,4.85027195763531,1.92179176012225e-05,0.000570974446625796,2.69577351319479

"VCAN",0.924735248142851,8.289870010575,4.83856828770184,1.99429339462949e-05,0.000589309303311666,2.66039941709547

"LPAR4",0.689853952255407,8.018302016325,4.81933701669742,2.11933571297147e-05,0.000622672994604477,2.60231714539315

"SGK1",0.885177650666662,8.9893699538,4.79365972795302,2.29842842369704e-05,0.000668976772275783,2.52485322798158

"PRDX4",0.95172345598268,11.3994893949,4.79146038355466,2.31444534351183e-05,0.000671815521835889,2.51822286606576

"DUSP14",1.02992021373159,8.9173503679,4.76504290051021,2.51565666730655e-05,0.000721435176074771,2.43864082156712

"OSTF1",0.754178483571424,9.278969922375,4.76155858075371,2.5434518215265e-05,0.000728432376816623,2.42815256865199

"GINS3",0.699950760523804,8.105566241575,4.75934163814732,2.56129430678327e-05,0.000732564322330774,2.4214802873097

"CDR2L",0.757631847891769,8.807828032225,4.75827731413789,2.56990405059568e-05,0.00073404809306695,2.41827729334088

"LHPP",0.721761954606061,8.75192213355,4.75656295132672,2.58383228093077e-05,0.000736066218569002,2.41311844131326

"GPR88",-1.23771249694806,8.731508326875,-4.74167174463191,2.7079894138437e-05,0.000765337034471163,2.36832769636722

"SGSH",-0.77227550255844,9.714100879675,-4.73930622882795,2.72824642948576e-05,0.000769035665688555,2.36121583187779

"ATP6V0A1",-0.590266266341995,9.595628316125,-4.73229866270215,2.78913654362899e-05,0.000782088470554057,2.3401530910202

"SLC35E1",-0.748742573415587,9.507590441575,-4.73099139290068,2.80064306175094e-05,0.00078398784594724,2.33622469713152

"IGFBP4",1.83384080002164,10.288201097875,4.7302893964938,2.80684129833044e-05,0.00078398784594724,2.33411528731411

"CYGB",-0.776195094861471,9.054309360025,-4.7301766341789,2.80783818662762e-05,0.00078398784594724,2.33377645842678

"EFHD2",0.869954573056269,8.6455539812,4.72945973944761,2.81418414702986e-05,0.00078398784594724,2.33162237744056

"PRRX2",1.33369573996537,9.1410385819,4.71632922658417,2.93294878813365e-05,0.000810283558140226,2.29218356139558

"NDUFA12",0.647778565220776,9.61635131645,4.7104995192045,2.98724915988162e-05,0.000816299130300899,2.27468258619744

"RCN2",0.613730131259738,10.671161051575,4.70927654918498,2.99876554339042e-05,0.000817363655289301,2.27101190712062

"COL1A2",2.06702180694371,11.9147730978,4.70091667022843,3.07866874018499e-05,0.000831744624001362,2.24592687457728

"CCDC106",0.850273799363628,8.178926332475,4.69944688495373,3.09293213610255e-05,0.000832663264226279,2.24151778201669

"ZFPM1",-0.704909347121216,8.244888459625,-4.69625840644105,3.12409868592833e-05,0.000839787480098353,2.2319541685469

"TIMP3",1.14198835064069,11.08926418985,4.6911223813518,3.17495396881018e-05,0.000846036491738474,2.21655264023845

"TNFAIP6",1.15973379682035,8.8764962256625,4.68609318802009,3.22554162625471e-05,0.00085738653562317,2.20147580783694

"MYDGF",1.08169956563203,9.918384387075,4.68168399858225,3.27054558060174e-05,0.000866129299376393,2.18826120839475

"GLRX5",0.624498022683973,9.915060647,4.66746573516053,3.41992465229693e-05,0.000903711043273312,2.1456709728922

"CYBA",1.01824227836796,10.249015975225,4.66565565848607,3.43941743695496e-05,0.000905263109694734,2.14025146714509

"SAFB2",-0.841391277056278,8.545642043,-4.66234525446462,3.47535080288872e-05,0.000913599878342719,2.13034134129623

"TPI1P2",0.612003214848484,8.08115296925,4.66094653497079,3.49064434018649e-05,0.000916497083737337,2.12615466566914

"KPNA6",-0.718659094740262,9.525490300125,-4.65772702894769,3.5260986015156e-05,0.000923152952089308,2.11651927239194

"EFNA1",-0.740615338482687,8.0168149360375,-4.65747354690827,3.52890504271704e-05,0.000923152952089308,2.11576072390647

"LINC01720",-0.933428895199139,8.476561707175,-4.64774826885475,3.63825629420816e-05,0.000948289620498897,2.08666621182943

"P3H1",0.742967696692634,8.9402865202,4.63953961022539,3.73314616346549e-05,0.000969256308626634,2.06212186592411

"MYBPH",-1.04591037572295,7.4085649966,-4.62900452616647,3.85850628193562e-05,0.000994157881062899,2.03063895265906

"MYCBP2",-0.634488363186151,8.4635704778,-4.62883290953257,3.86058232681644e-05,0.000994157881062899,2.03012626011816

"RBM5",-0.718473811411259,10.1221800433,-4.6266497470124,3.88708818425867e-05,0.000999783317032766,2.02360467859129

"TRNP1",0.61642583955411,8.356693069775,4.62114429664106,3.9547281304682e-05,0.00101474728620423,2.00716251248063

"SERF2",0.997542830471856,12.129687140425,4.61903776337742,3.98091430549849e-05,0.00102024603067202,2.00087274619576

"LRP1",-0.713357024917755,9.0406645523,-4.61188670459395,4.07108802853357e-05,0.00103839368965605,1.97952685166228

"COL5A2",1.16930459245021,12.0146984942,4.61056029434871,4.08803348632792e-05,0.00104147750968195,1.97556854336334

"EFEMP2",0.79100619117748,10.74569741015,4.60889353360515,4.10942550989473e-05,0.00104444652384777,1.97059500755165

"TSPO",0.714245210080808,10.73851617765,4.60700713057274,4.13376944051353e-05,0.00104939039370953,1.96496668216482

"NLRP8",-0.695222874759745,8.3991097180375,-4.60381267285627,4.1753177805583e-05,0.00105494395419029,1.95543709448576

"HSP90B1",0.685194321991334,12.5783953885,4.59782800692596,4.25426563262655e-05,0.001072364889371,1.93758890929036

"EFNB3",0.603700221506487,7.6733162306,4.59239344395334,4.32722805620756e-05,0.0010881989335722,1.92138703411675

"ERH",0.822996255142856,8.99923612235,4.58597770893515,4.4149490241147e-05,0.00110538913960949,1.90226707791904

"NUBPL",-0.778492169731605,9.2947149444,-4.57737210053035,4.53536369775482e-05,0.00112471776509293,1.87663299964759

"CEBPD",-1.22044397707792,11.121980614625,-4.56998982689108,4.64123058475325e-05,0.0011443567502706,1.85465403456672

"GPX3",-1.07151265212122,13.191139322,-4.56342735375658,4.73737899164903e-05,0.00116404944730657,1.8351244835893

"GNG11",1.16145504311254,9.505222640425,4.56261396588097,4.74943173436556e-05,0.00116567574523885,1.83270445671842

"GDF5",0.735014153212119,8.2170840074,4.55460125782341,4.86978689053841e-05,0.00118706589305613,1.8088714457764

"MEG3",-1.63999619072295,9.885600262225,-4.54614507528609,5.00005560054038e-05,0.00121056650888478,1.78373271296255

"CFH",-0.983813182334419,10.5179310654313,-4.54202494111412,5.06476210804931e-05,0.00121798443923504,1.77148928012818

"SPEN",-0.701909464064938,9.510392468575,-4.5397529564752,5.10079489471872e-05,0.00122097917245089,1.76473923361564

"DMC1",-0.608949790885283,8.3087210846625,-4.53838777604544,5.12256719300838e-05,0.00122365466433433,1.76068377635787

"TSHZ1",-0.602742445805198,8.431394975925,-4.53460491311026,5.18337577934096e-05,0.00123394491858788,1.74944815277947

"ITPK1-AS1",-1.19372893306927,10.361359473075,-4.53089210097105,5.24374802708847e-05,0.00124291313733784,1.73842330303195

"ADM",-1.47923716243723,10.396062995275,-4.52952677059459,5.26612236059993e-05,0.00124393693038855,1.7343697593543

"FN1",1.22381551873881,9.27853319948333,4.52807720652021,5.28997976091642e-05,0.00124698193243317,1.7300665329269

"PRSS23",0.882399263246753,8.09194490975,4.52411262450762,5.35577401176315e-05,0.00125945137388368,1.71829924252426

"PTGES",2.39377040987013,10.640510787,4.51372384595105,5.53201937735355e-05,0.00128846197246049,1.68747896586243

"TFDP1",0.656816552893934,9.0915206241375,4.50879186735858,5.61767305691784e-05,0.00130699245926187,1.67285480553206

"ZBTB4",-0.630516491805201,9.189695837975,-4.50728537229494,5.64409517543096e-05,0.00130811367804607,1.66838875555449

"GALNT18",0.756618822558435,10.615431976325,4.5072021913505,5.64555762087509e-05,0.00130811367804607,1.66814217632827

"PGRMC1",0.628479623956704,10.22895173405,4.50626869833055,5.66199537777948e-05,0.00130866150814829,1.66537505088865

"ATP6AP1",0.813126974978349,11.8436218615,4.50044109430327,5.76568102190317e-05,0.00132561225724378,1.64810439857927

"NDP",0.71163505424242,9.48788747475,4.50002089267906,5.77322899613952e-05,0.00132592650102986,1.64685935462341

"AHI1",-0.827295568112559,8.97129320945,-4.49581458989305,5.84932263242613e-05,0.00133767398494854,1.63439816977414

"PLAAT3",-1.00684664045022,8.9018333422,-4.48794418991102,5.99435636155308e-05,0.00136402690389532,1.61109167057763

"HSD17B7",-0.652321885982689,8.81552912085,-4.48681630699224,6.01542795713472e-05,0.00136402690389532,1.60775271474731

"SLC16A10",0.707589294021645,8.354300402425,4.46871869697308,6.36365272486411e-05,0.00143238945016852,1.55421249161217

"TRIB1",-1.48898616422944,9.301421056225,-4.46608633230282,6.41592492114895e-05,0.00143962348832182,1.54643046524262

"ENOSF1",-0.766590057714287,8.1004428381,-4.46149278360829,6.50815273648391e-05,0.00145574957612426,1.53285404428664

"ASS1",-0.657346487606063,9.078052732225,-4.45239982331313,6.69457236571538e-05,0.00148725909361549,1.50599234220055

"GPM6B",-0.650593972562773,7.50318790501667,-4.45225642240461,6.69755388761625e-05,0.00148725909361549,1.50556885656855

"UBB",0.81888087473809,12.8991822435875,4.44467466849066,6.8570597502137e-05,0.00151639988352406,1.48318486304073

"DCXR",-0.623043026528142,8.9918365934,-4.43448415090489,7.07733432335884e-05,0.00155230980133303,1.45311792809071

"COL2A1",1.04092292078571,11.7712008603625,4.42335149896985,7.32591162455254e-05,0.00159703384408818,1.42029639864374

"DRD4",-0.669309939770566,7.403236637975,-4.41882319809039,7.4294552723495e-05,0.00161304903893896,1.40695352991069

"COL3A1",1.46966885241125,12.722567620525,4.41639728934948,7.48551496375577e-05,0.00162193718674268,1.39980727545345

"YIF1A",0.833056265333331,9.7257486419,4.40833310562806,7.67486631263538e-05,0.0016479835562797,1.37606086887159

"SMOC1",0.984572130240256,9.3021798221625,4.39911292928077,7.89712039655319e-05,0.00169063003619224,1.34892768628507

"GATAD2B",-0.650233203900438,7.650590691925,-4.39420973407872,8.01787081555134e-05,0.00171287976368757,1.33450607705345

"EZR",0.932613586956705,10.378576750525,4.3941974591859,8.01817536785644e-05,0.00171287976368757,1.33446997990205

"ZNHIT1",0.683309728541124,9.982306576475,4.39076057837005,8.10389547702335e-05,0.00172628264029422,1.324364337985

"GAS1",1.06087999835713,10.4251360662875,4.38783752760551,8.17750650608792e-05,0.00174023504029853,1.31577157703777

"MYOC",-0.797357471627709,7.40595371355,-4.38715304231053,8.19483835497857e-05,0.00174219502034336,1.31375969776948

"SHC4",0.987054351727267,8.377256497175,4.3803862026268,8.36813078408772e-05,0.00177376258349274,1.29387576299251

"TNFRSF11B",1.79055783848485,11.22611241875,4.3771721712971,8.4516923763422e-05,0.00178442178311926,1.28443507831657

"SEC16A",-0.686320447922084,9.58158291275,-4.37262028521519,8.57143776046337e-05,0.00180260697450686,1.27106858846358

"TCEAL6",-0.834210964008664,8.10046609055,-4.37050170645031,8.62773622471592e-05,0.00181266963522411,1.26484901126906

"ALPP",-1.05966451762338,9.692809632675,-4.3667827583897,8.72744007424147e-05,0.00183003242456064,1.25393360162752

"SRRM2",-0.692367043030305,11.1790913495,-4.35211567687034,9.13178452857088e-05,0.00190180495070266,1.21091478235422

"CCDC102A",0.697984413155837,8.795007645425,4.3315591751098,9.72952732746193e-05,0.00200295672458144,1.15070419728372

"USP49",-1.11769338997403,8.8419075457,-4.31596227240568,0.000102084597975385,0.00208070698087317,1.10508513034927

"CENPBD1",0.646621990926404,8.6184738158,4.31578914136747,0.000102139035516267,0.00208070698087317,1.10457905910583

"SCARB1",-0.588931172554116,8.2164716935,-4.31382599735409,0.000102758297891219,0.00209133609873295,1.09884117259

"C5orf15",0.665822162510819,11.2945810055,4.30555781418584,0.000105407032074297,0.00213310023115637,1.07468475710308

"ALDH18A1",0.629178021077923,9.263185279675,4.2907316301749,0.000110324983631613,0.00220557429998297,1.03140861389095

"CLDND2",-0.594164166692639,7.71177100405,-4.28760173890703,0.000111391537255021,0.00221964754289702,1.02227941755589

"COMMD7",0.617814988519479,9.0916911411,4.28518781986124,0.000112220998075105,0.00223307294036092,1.01524014260974

"NPIPB11",-0.719291729610392,11.4448092945,-4.27244234460899,0.000116701734849292,0.0022987776990378,0.978095879689092

"ADTRP",1.0218741232987,8.48807272815,4.26767484709864,0.000118422356132507,0.00233053023981506,0.96421192912995

"CDC42EP4",-0.733572688493508,9.82448852885,-4.26033151026072,0.000121121263158742,0.00237927858609724,0.942837328197259

"SPARC",1.38672008650649,11.845322181225,4.25761174410623,0.000122136051245841,0.00239263966691738,0.934924090297129

"FNBP4",-0.767448275528146,10.175806366975,-4.25701438311924,0.000122360047213746,0.00239484066859678,0.933186291969978

"MRPL33",0.668807405339825,10.4739046527625,4.24997136079852,0.00012503148871662,0.00244266891116595,0.912703805429504

"NKTR",-0.703466342313858,8.9347463646625,-4.24518752768469,0.000126878445962352,0.0024652803843645,0.898798393249734

"CITED2",-0.654063312069266,7.7736743614,-4.24143608460972,0.000128345449436575,0.0024915278152615,0.887897807806401

"RCN3",0.813520727004327,9.78844580735,4.24073167964562,0.000128622750378625,0.0024946533619999,0.88585140076239

"FUT4",0.781143248199131,9.05626724605,4.22980760334226,0.000132998912082778,0.00255412682460847,0.854130913107312

"GUSBP3",-0.598924209290047,7.41010800505,-4.22431751141777,0.000135252841134539,0.00258583662671746,0.838200371681174

"CNIH1",0.63989926742207,10.0491102993375,4.2237235572931,0.000135498912059019,0.00258823433889405,0.836477350802542

"NDN",0.621057196264061,10.819506292775,4.19745780072498,0.000146829097257594,0.00275558264678273,0.760370611797596

"PGK1",0.623288494584409,10.422886693675,4.18311910665548,0.000153399503477309,0.00285639995580882,0.718897040397411

"ERAP2",-0.729569962435066,9.8009636015625,-4.18099603561914,0.000154396443188058,0.00287247016723941,0.712760692028968

"TAGLN2",0.850329865924238,10.0154415313875,4.14808225775585,0.000170685495499816,0.00309499117833184,0.617778738032302

"STC1",-0.81844042039827,7.2713713636,-4.14726435489287,0.000171110917149853,0.00309997952175886,0.61542205195351

"RAB32",0.616247847701295,8.790422123925,4.14619456189371,0.000171668917933692,0.00310494937486985,0.612339841425488

"LRRFIP1",-1.32633531757143,10.420234311575,-4.13748694325178,0.000176277298849265,0.00317225196108691,0.587263314600671

"PPDPF",-1.05778917739394,8.70736009065,-4.13082693992273,0.000179883237546124,0.00322631716438287,0.568097147808975

"GRN",0.645724208335491,9.1131309121625,4.12008098252509,0.000185853644392573,0.00331400376214886,0.537197351472735

"ETS2",-0.826542614545458,9.265486614,-4.11675731575374,0.000187739071546666,0.00334484287686672,0.527646475046214

"ZNF786",-0.798235838831172,8.40068610725,-4.10975133139462,0.000191774663965381,0.00340825046952891,0.507523846753628

"RPN1",0.770760022380948,11.44522620275,4.10327991832828,0.000195577385414519,0.00345423956620071,0.488948404237427

"F13A1",-0.743360997463206,8.16112262695,-4.10265779889484,0.000195946803069268,0.00345662407289381,0.487163280731375

"GPR137",0.637255449753242,10.012730316475,4.08703239747854,0.000205451514659155,0.00359473119164237,0.442362006049554

"FKBP14",-0.661465699987016,8.986179260725,-4.07631811388517,0.000212226937626377,0.0036981966198403,0.411680633635962

"SCNN1A",-1.06271583288745,8.545187284725,-4.07305751761152,0.00021433170511404,0.0037257936842798,0.402349897654404

"CFAP69",-0.647594342125541,7.790696883175,-4.07277123909706,0.000214517470462689,0.00372600344849808,0.401530804970451

"CCND1",1.06062250374891,10.71768898245,4.07104915703734,0.000215638238302576,0.00374244000795191,0.396604108038002

"BMS1P10",-0.765373348948056,7.663435576975,-4.06549832212408,0.000219289796327377,0.00379659840275913,0.380729358502209

"DKK3",0.80236849466991,8.74478552778125,4.06327048440249,0.00022077222944028,0.0038130314764279,0.374360411965641

"TSEN15",0.677858079870131,8.37500966975,4.06048948824698,0.000222636440969048,0.00383596328933899,0.366412039461921

"APBB3",-0.749440577512992,9.1122909298375,-4.02892589008643,0.000244898711144568,0.00413973384772429,0.276352616112059

"COLGALT2",0.957203700874457,11.21290430665,4.02630251911307,0.000246843592892387,0.00416064660652541,0.268880163394042

"CA9",1.82919939541558,10.224613948075,4.02237643182936,0.000249782369929225,0.00419229016259808,0.25770071330133

"PLAAT4",-0.970071310458879,8.1924624703,-4.00355348397514,0.000264351201602335,0.00437883986530633,0.204164418064404

"TMEM204",-0.751689806186153,8.966548532325,-4.00327362352385,0.000264573927660892,0.00437914762519583,0.203369211623001

"HAPLN1",0.789786925796525,10.293647197925,3.98555985810122,0.000279048844246477,0.00453475511964482,0.153083129051589

"HLA-B",-1.06875447281819,10.338873709925,-3.97264910918544,0.00029008101301594,0.00468211272400672,0.116489999539313

"TMEM267",-0.59180202314286,8.30704104305,-3.96789921422124,0.000294245488040897,0.0047330478047324,0.103039720429749

"STT3A",0.664166875740252,10.3818532342,3.96253926507793,0.000299014601757536,0.00479404031979687,0.0878700139953743

"TPPP3",0.588196818779219,7.73840464435,3.9571775919354,0.000303860347166512,0.0048570106610051,0.0727040112959045

"RAI14",1.04457508144588,9.99517769905,3.95600026685519,0.000304934553432379,0.00486330937225127,0.0693749877015106

"MFSD3",0.748363393831164,8.401677658625,3.94967859485463,0.000310765905262384,0.00494161559213003,0.0515068412625483

"CLEC3A",1.65893609171645,10.7179653637375,3.94140358008104,0.000318562977640046,0.00505562552894059,0.0281357843107868

"SERPINE1",1.74428525607792,9.32688966855,3.93815228731732,0.000321678183116727,0.00509248612991655,0.0189588514921306

"SSTR2",-0.795088032467532,8.3714600045,-3.93761314032564,0.000322197614414545,0.00509694765988673,0.0174373925789482

"NDRG2",-0.694622966116889,8.666430435025,-3.9317892661933,0.000327860683477081,0.00516368540474807,0.00100819997058377

"CDC25B",-0.594455724367966,8.794849648825,-3.91785159083131,0.000341808648008519,0.00535192504265017,-0.0382680571867713

"UBE2M",0.663820793575757,10.2219081417,3.91090773408087,0.000348970872569307,0.00541270729391483,-0.0578135359007357

"FLOT2",-0.656877649471866,9.8621884139,-3.89409355971088,0.000366922225074304,0.00564784390859394,-0.105080050787045

"MYLIP",-0.632532715303034,8.209032229875,-3.89399785935551,0.000367026917560018,0.00564784390859394,-0.105348823471714

"RNF24",-0.695133315242426,9.251011952925,-3.8906269654126,0.000370733083583668,0.00570078521573711,-0.114814091899543

"SNORC",1.21971270402596,13.40393610225,3.88792043447112,0.000373735053085965,0.00572642187410503,-0.12241129637153

"FNDC1",1.09001221148701,9.0050919232625,3.86914803252029,0.000395214944525767,0.00594096410302889,-0.175041843832202

"GOLGA8B",-1.14288348838096,9.2558050858,-3.85604226896538,0.000410915227653225,0.00614215890571103,-0.211719166087089

"H19",1.2400603669264,13.662383257,3.82788346741722,0.000446718978174154,0.00659048748336574,-0.2903370288175

"C4BPA",-0.807838383125544,7.47514141435,-3.82031078765731,0.000456851810276381,0.00670309725187322,-0.311435704254619

"SNRPN",0.600696565212115,10.2560733133,3.81871341207327,0.000459017462226055,0.00672751367547757,-0.315883851857165

"PPP1R13L",-0.628553336961043,8.06719275615,-3.81583660621176,0.000462942798986187,0.00675345304331038,-0.323892670683444

"IGFBP3",1.70474044548051,9.05386210745,3.80963825233118,0.000471511035583848,0.00685256316010103,-0.341139210427803

"MALL",0.723141149038955,9.9890198241,3.79610305801838,0.000490758027381815,0.00705107196608661,-0.37875603790912

"CXCL14",1.40676934335498,8.310868540125,3.79092107146596,0.000498326181404116,0.00712708715283076,-0.393141676751315

"GYG1",-0.65058237736797,9.1136995101,-3.79088825369146,0.00049837446875419,0.00712708715283076,-0.39323275324687

"FASN",0.684963805502161,9.691596409825,3.78720689294733,0.000503819965838691,0.00720016128394787,-0.403447055832078

"ECHDC2",-0.721261909666675,10.410154494525,-3.78038292703313,0.00051406661000877,0.00730764933816973,-0.422368861156575

"RBP4",-0.930200808367967,8.429077427525,-3.77888648628369,0.000516340408101343,0.00733330377746846,-0.426516176761165

"NGF",1.45229952450216,9.194321458,3.77312198328287,0.000525190626152101,0.00743130878732765,-0.442485202135831

"OGN",0.838954462761905,9.07696500235,3.77071432335194,0.000528930358060014,0.00747436436807995,-0.449151675870805

"TUBB2B",0.949912235926401,11.560118479925,3.7636022633624,0.000540128209864265,0.00760754447130555,-0.46883255398431

"COL6A3",1.12295853415584,12.26579094425,3.75782627500962,0.000549390715596907,0.0076825164538913,-0.484803585172402

"SNHG5",-0.713201063363643,9.932909948225,-3.75004667365924,0.000562109164996409,0.00780945835384584,-0.506296832934808

"TXN",0.60976442117965,11.0004967006875,3.74959932974147,0.000562849088341375,0.0078146768893274,-0.507532114053327

"UBE2S",0.687939607476185,8.860992595025,3.74854862795069,0.000564590690439929,0.00782872327125205,-0.510433219487192

"ACKR2",1.10441648433333,8.949767073575,3.74349618622924,0.000573038387151163,0.007925368435061,-0.524378306023586

"RARRES2",-1.07710573285715,8.31876828325,-3.74017514944269,0.00057865753734903,0.00799277709830911,-0.533539832923956

"PDLIM3",0.860646044874455,8.85339949445,3.73833752363873,0.000581789609355043,0.00801404785125698,-0.53860754402095

"PMP22",0.710143739629141,10.5161258022417,3.72822197324453,0.00059932596758783,0.00820430206172721,-0.566482879438877

"ZNF486",-0.986780344839834,10.66409010265,-3.72038698560104,0.000613258397838053,0.00836832435879394,-0.588049379618299

"CHST13",1.08063445223809,8.028586448525,3.7160462374578,0.000621111319575167,0.0084579810295609,-0.599988499504193

"MPDZ",-0.599092477549787,8.82382162645,-3.71336407927207,0.000626012080349682,0.00850987651177505,-0.607362429132832

"ANKRD30B",-0.799189189112555,9.737785837125,-3.70828106390116,0.000635402193189376,0.00862113374200209,-0.621330031862111

"NOTCH3",0.764955961272723,7.78038378505,3.6984339713642,0.000653981402819989,0.00877882044548909,-0.648363078033122

"UBE2SP1",0.604010197233765,8.238269063575,3.6974369814322,0.000655891450896425,0.00879895404201326,-0.651098197000768

"SCRG1",0.799862950909084,14.0266467745,3.69492939052872,0.000660719350812598,0.00882508766767188,-0.657975915365681

"RAB31",1.09303792205194,10.07242436155,3.68329243311887,0.000683576405056519,0.00901253685609551,-0.689864205831801

"LRRC15",0.70931451081385,7.88386582185,3.67852626569138,0.00069315648377142,0.00909412827729708,-0.70291087932597

"MYO1D",-0.615314626848489,9.84736249985,-3.67569326636907,0.000698912065407092,0.00914168458234605,-0.71066196058586

"IRX3",1.15752504676623,8.240254344725,3.66459249842947,0.00072191177166183,0.00939098205816732,-0.741006006500379

"TUBA3FP",-0.71748120155844,12.964879493,-3.66166428815697,0.000728099294672582,0.00944284036881594,-0.74900293083671

"ZNF358",0.715875549580083,8.928859251975,3.65698912975658,0.000738084318737655,0.00953773898869967,-0.761764364420647

"CILP2",1.24146618150649,9.8725904746,3.65036176251738,0.000752465166545244,0.00967113857852728,-0.77984108388917

"LGALS1",1.15595232722943,12.27253190425,3.64302508837326,0.000768699941687693,0.00982095440687475,-0.799833949375006

"CENPB",0.904320117424239,10.353463588875,3.63883334845326,0.000778126423604777,0.00992365630959932,-0.811247904795288

"WDR18",0.659868391839828,8.839721669125,3.63478105343406,0.000787345087017929,0.0100130312351304,-0.822276068320726

"UQCRQ",0.722740812303021,11.62107680815,3.62296459820725,0.0008148309674337,0.0102938392711545,-0.85439978568695

"NT5DC2",0.779904593943719,8.887328030575,3.62031889685769,0.000821110405583563,0.0103305802405707,-0.861585252568147

"NPDC1",-0.616593990671003,8.415593415125,-3.61640204405106,0.00083049244937885,0.0104241623941637,-0.872218306604919

"TRAPPC3",0.587654722610386,9.455005038725,3.58883438446167,0.000899504376481651,0.0111083870926355,-0.946895072996422

"CLPTM1",-0.625411520683988,9.68982573415,-3.5842857507202,0.000911408857391967,0.0112102244265568,-0.959189377947955

"CD55",0.66652022724675,8.45191764155,3.5739242211771,0.000939094719979958,0.0114783594520171,-0.987166024086652

"DNER",1.22298788624242,9.15260848315,3.57174550329087,0.000945018214244672,0.0115244944364767,-0.993043511086741

"TNFRSF12A",0.964226620073588,8.401478903275,3.56861078070029,0.000953603928131578,0.0116028121737666,-1.00149683939836

"SLC16A3",0.871260677874455,10.838452900675,3.56425852769278,0.000965648713543643,0.0117294057498441,-1.01322726830509

"PFN1",0.633070548987012,10.6794318527,3.55293389590816,0.000997678307692025,0.0120403584360079,-1.04371622343562

"SEMA3E",-0.674121539523817,9.39170264775,-3.53989657857713,0.00103581569058291,0.0123509073811529,-1.07875537926848

"RNMT",-0.698005961766242,9.2011335274,-3.53508114033868,0.00105025291419759,0.0124814267382008,-1.09168082475885

"TMEM59L",1.26893536206493,8.205850278275,3.53063389297461,0.00106375767880564,0.0126213104427597,-1.10361002132217

"CTSZ",0.852369762147184,8.7572709162,3.52671900188856,0.00107578365832198,0.0127424821947348,-1.11410489378523

"PLOD2",0.645516283274162,10.0671970535583,3.51207123141761,0.0011219471584068,0.0131873361616352,-1.15331909746623

"EFHD1",-0.964946910787882,9.5954342746,-3.48818597671696,0.00120132450600177,0.0139521450883833,-1.21708287158075

"CPQ",0.640298087409088,9.7294415846125,3.48798339426612,0.00120202022698059,0.0139526709355847,-1.21762271923403

"ABCA1",-0.777055886190478,8.16724234175,-3.47315627335926,0.00125400215640812,0.0144311160177631,-1.25709013300326

"KLHL35",0.606804815073589,8.17912205415,3.47218773740531,0.00125747161518583,0.0144496574678299,-1.25966516776124

"D2HGDH",-0.838886168099567,9.196273257175,-3.45592545551873,0.00131711722194041,0.0149965400890891,-1.30284526538806

"FOXO1",0.638124766047612,10.075206876275,3.45411643802114,0.00132391720415923,0.0150659670803288,-1.30764203963188

"C1orf198",-0.622876833419921,9.132507951,-3.45350235001744,0.00132623315318189,0.0150768623358752,-1.30927004910777

"RAMP3",0.694962190822508,8.332266901,3.45289115876571,0.00132854202806674,0.015094573646218,-1.31089022760425

"ARPC3",0.624931479385278,10.58143280435,3.4524764950419,0.0013301106783847,0.0150964043185345,-1.31198935438366

"IL18",-0.746098389653683,11.43118037425,-3.44794623200232,0.0013473643140996,0.0152357996319191,-1.32399294369267

"LGMN",0.592613475590905,9.9895432938625,3.4434229435223,0.00136480497774819,0.0154086481987771,-1.3359697490335

"APCDD1L",1.36813369769264,9.242009122025,3.44106766288589,0.001373971674391,0.0154655249706926,-1.34220279119229

"COA3",0.630704447874459,10.216048377925,3.43591445800661,0.00139423371647889,0.0156502917070584,-1.35583241383484

"PID1",0.816138102060602,10.4655881852,3.43347798601915,0.00140391299214308,0.0157424660713336,-1.36227282435305

"KRT10",0.702432175610387,10.49046735945,3.4291993805725,0.00142106619281968,0.0159143480863519,-1.37357673650969

"RGCC",0.639856081298689,13.3967155785,3.4217774619682,0.0014512976156484,0.0161807615141756,-1.39316736484152

"IGF2BP2",-0.608468865770565,8.113366754025,-3.41910441472669,0.0014623355197798,0.0162699996031102,-1.40021749943086

"SH3PXD2A",0.711280856164505,10.03145923805,3.41082639665186,0.00149702966874041,0.0166129246891622,-1.42203199968276

"NDUFS8",0.589862180787877,9.92573561615,3.40133283478454,0.00153778685917954,0.0170091067604668,-1.44701483460737

"SND1-IT1",-0.618673554692644,7.90412750295,-3.39968662803943,0.00154496127644884,0.0170741701911922,-1.45134311192392

"ECM2",0.635262286640693,9.78120543505,3.39017640887996,0.00158703835754985,0.0174403482621935,-1.4763256804685

"ASCC2",0.654386661316014,9.6179570483,3.38790474528253,0.0015972498874439,0.0175345994552503,-1.482287565179

"NTN4",-0.642466813077922,8.588815164925,-3.38759025686039,0.00159866850396161,0.0175411959480719,-1.48311275646944

"HSD3B7",0.68072550407359,8.427066959575,3.38492680331101,0.00161073134263389,0.0176464749902143,-1.49009977396738

"NDUFB11",0.601261853636363,10.56982135525,3.37453859878834,0.00165861554535309,0.018051223776443,-1.51732260135643

"JMJD8",0.613591416060597,11.91424484825,3.37005277791715,0.00167971027298705,0.0182437797801748,-1.52906387305104

"SERPINH1",0.93144994382684,9.5264438238,3.36115634312379,0.00172230594896854,0.0185934498798813,-1.55232438695068

"SETBP1",-0.698834716225111,8.8794138664,-3.35461794147703,0.00175426649114872,0.0187965886621535,-1.56939819516056

"ATF5",0.605478383458872,8.225464869925,3.35233314206834,0.00176556768178739,0.0188893727391628,-1.5753602278234

"NPIPB13",-0.83548077179654,10.504712826125,-3.34585205221222,0.00179800386086167,0.0191694735682623,-1.5922600745364

"NDUFB7",0.704669986186147,10.579596210175,3.34542404090142,0.00180016583109119,0.019182989191623,-1.59337550849158

"LDLR",0.781970054787875,9.1505249632,3.33840791445267,0.00183596098812893,0.0194677207891022,-1.6116489378537

"VIT",-0.945475043489181,7.72699122155,-3.3311270558327,0.00187382368544927,0.0197467579636809,-1.63058949220761

"DSG2",0.668217557086578,7.853740492025,3.32906163862105,0.00188469926495376,0.0198277017815219,-1.63595834539977

"ENPP1",0.620421736584412,8.828817432825,3.32662360774446,0.00189761427764564,0.0199147377053702,-1.64229340123271

"ECM1",0.732021732170991,8.0876063581125,3.30821881557043,0.00199786717121128,0.0207636863806459,-1.69003383423913

"LAMA5",0.711099866692637,8.49429257645,3.30242366593952,0.00203046446086905,0.0210108505306811,-1.70503540937084

"GLRX",-0.879504343082258,8.5735250751,-3.288475905429,0.00211100792435001,0.0217081644224507,-1.74108082807868

"FAM43B",0.623535605926405,8.986183854175,3.26619497851305,0.00224601124085135,0.0228013190380986,-1.79848358169808

"SLC25A37",-0.916184073203468,9.67120541025,-3.26265353103185,0.00226821135979271,0.0229940462565753,-1.80758715229135

"SRPRB",0.651895001584412,10.62747427245,3.25645728954694,0.00230755603499409,0.0232609889598958,-1.82350159829882

"PSMB6",0.603825112359303,10.864557622125,3.23774014984043,0.00243038136848449,0.0244187872296772,-1.87147006315223

"PLAUR",0.812072413522363,8.57933799910833,3.21956382251012,0.0025555808476495,0.0253399918966111,-1.91790088073794

"ENPP2",0.660106740915583,8.2964869486125,3.21912352219724,0.00255868828716888,0.0253399918966111,-1.91902374789327

"PENK",1.7615337323593,8.763282966625,3.21796415867033,0.00256688770582444,0.0253977436243727,-1.92197996837701

"SLC2A8",0.669315186826836,9.113104576275,3.21778413288115,0.00256816314427355,0.0253986480441733,-1.92243895477256

"RNASET2",0.662276040857133,10.44396893585,3.20200355998667,0.00268233086906207,0.0262613781251714,-1.9626147782821

"NINJ2",0.833984546740252,9.121361214775,3.19999698401579,0.00269718807707211,0.0263827548751819,-1.96771513012443

"SGCA",-0.588718134549786,7.738955091425,-3.19724099551602,0.00271772134206938,0.0264990184130592,-1.97471732754698

"SERTAD4",0.618733254190472,9.46191244285,3.18916390329244,0.00277875594766678,0.0268621423314106,-1.99521875567846

"STK32B",-0.726181709580091,7.833547501025,-3.18847907768925,0.00278399014830094,0.0268751729672618,-1.99695560453473

"U82695.1",0.661845467636361,8.6697971788,3.18046729045556,0.00284592324063766,0.0272817600350049,-2.01725886446126

"ADGRG2",0.652563240880953,10.2145510250125,3.18041549305995,0.00284632786156081,0.0272817600350049,-2.01739003100543

"WBP2",-0.726560673593072,12.29541714,-3.17525607690093,0.00288690538012793,0.0275476011161585,-2.03044895959297

"UBASH3B",0.86424701612121,9.5100357903,3.15119314015097,0.00308349646363475,0.0289978442093069,-2.09118980979805

"PHGDH",0.702178815242416,10.745379613575,3.12254028129994,0.00333410838997026,0.0309075017602645,-2.16316003274909

"IL11",2.19717604055844,8.662179726175,3.11358228947548,0.00341632954899432,0.0314522253886168,-2.18558034317113

"STC2",-0.897129382562772,10.2302743751,-3.06039500339422,0.00394531712492086,0.0352042419495331,-2.31789905126621

"AKR1C3",0.585928872536791,8.3252473947,3.05816913729933,0.00396905661180481,0.0353572397756748,-2.32340638084284

"HNRNPUL1",-0.699070488108228,8.478821369025,-3.05617672962076,0.00399042028773235,0.035518052112924,-2.3283340047505

"VAMP8",0.655194732965363,9.375096901125,3.05464229507076,0.00400694711922316,0.0356034565716218,-2.33212763306581

"CTSK",0.710847253419903,10.6972877255,3.05341566669076,0.0040202050355374,0.0356647718020318,-2.33515942617398

"ACTN1",0.704811822441554,10.8067081338,3.00873403259012,0.00453227780147075,0.0391707861077151,-2.44508609191203

"SSR4",0.58535031004328,12.3224097515,3.0056945452157,0.00456925138073484,0.0393792733499972,-2.45252752198759

"ANPEP",0.948306097238092,8.25841572565,3.00151582801863,0.00462054594892893,0.0396832560714153,-2.46275044791021

"ITPRIP",-0.653421057683987,8.797317079125,-2.98158869374223,0.00487268317010591,0.0412227099097805,-2.51137877910191

"FRZB",-1.31119123574026,10.5068683248,-2.97913194850395,0.00490464771002558,0.0414182854365644,-2.5173599756277

"FOSB",1.7787584227922,11.154042012375,2.97903474761466,0.00490591642240756,0.0414182854365644,-2.51759655760171

"CHST15",0.594079070229434,8.952811545225,2.94973879908569,0.00530265431338276,0.0439009022293993,-2.58868009128076

"ARHGAP44",0.592086400489173,7.878818546975,2.94652848831868,0.00534791265173639,0.0441903213761161,-2.59644262106083

"ARID5B",-0.704886982818186,8.990933362175,-2.94002759837164,0.00544067170660011,0.0448748618163725,-2.61214536931539

"TMSB10",0.854555338168826,10.844065539275,2.93742637857885,0.00547820811436042,0.0451105728449694,-2.61842239309685

"PGAM1",0.819021971212115,9.60726994825,2.93038476094384,0.00558104139475376,0.0457577026561389,-2.63539686102431

"PLA2G2A",0.867069125259734,13.196218527625,2.92228116983577,0.00570161912124049,0.0463629385025511,-2.65489921021727

"CARHSP1",0.640596258406918,9.8965549769,2.91753376376479,0.00577338642777282,0.0467339291555301,-2.66630845642333

"BEND6",0.607944767883117,7.958191360075,2.91121088108117,0.00587028341440667,0.0472286737979688,-2.68148556495682

"MTHFD1L",0.627469326688308,9.227440461375,2.90708746002587,0.00593429124856264,0.0476408987922594,-2.69137184533724

"RPL7P23",-0.738928394151516,11.397371434825,-2.89334420348311,0.00615237089933452,0.0489657914131531,-2.72425758383155

"ITGB2",1.4079283937316,8.7532611154,2.8886431687508,0.00622867073327046,0.0493759112710217,-2.73548349661418
