# Supplementary material for: Identification and development of the novel 7-genes diagnostic signature by integrating multi cohorts based on osteoarthritis
Source: Hereditas. 2022 Jan 29;159:10. doi: 10.1186/s41065-022-00226-z (PMC8801091; doi:10.1186/s41065-022-00226-z)
Supplement: Supplementary file 3 — Additional file 3. Tableshowing the 4297 differentially expressed genes in the GSE51588 dataset, ofwhich 2664 were upregulated and 1633 were downregulated [file 41065_2022_226_MOESM3_ESM.docx]

"","logFC","AveExpr","t","P.Value","adj.P.Val","B"

"IL1R2",-4.1806759087,0.46619968304,-16.559356423375,2.9151085467282e-22,5.97247439053674e-18,39.9199568614342

"SRGN",-3.54627589255,0.48970555596,-14.2328220589924,1.78425424976318e-19,1.8277900534574e-15,33.8340462816282

"ANP32E",-1.182528374275,0.02891704458,-13.595122598581,1.16015706148771e-18,7.92309929192008e-15,32.0422908963229

"SLA",-1.9745104569,0.24360006448,-12.5602739032684,2.69995085676956e-17,1.19281276959969e-13,29.0163428811073

"SIAH2",-1.109398924875,0.0893419551,-12.5274224750456,2.9903417396999e-17,1.19281276959969e-13,28.9178599804191

"TKT",-1.8178695959,0.11438404328,-12.4612717436787,3.67484171430407e-17,1.19281276959969e-13,28.7190972439708

"C8orf89",1.86745664175,-0.2668555066,12.4281363590128,4.07540481608642e-17,1.19281276959969e-13,28.6193072970924

"IRAK3",-1.686312440425,0.24312287766,-12.3137735801764,5.83069668077817e-17,1.49324141994729e-13,28.2737239448078

"RPIA",-1.233136848425,0.13629001826,-12.0801791725755,1.21819697904046e-16,2.77315774517567e-13,27.5622058321728

"ANP32B",-1.001728386525,0.02401458578,-12.0068993280185,1.53720575079957e-16,3.14942714223816e-13,27.3374397144091

"FBXL2",1.495372791675,-0.15237418666,11.644793390545,4.90112960317141e-16,9.12857666452507e-13,26.2158744265366

"TBC1D8B",1.4716055983,0.00192012914000001,11.2724115852228,1.64351529884048e-15,2.80602845355365e-12,25.0436679875903

"CC2D2A",1.33819513026667,-0.137984301453333,11.1704429687878,2.29617048717407e-15,3.61876468778634e-12,24.7193848358887

"BAG1",-0.862049628658333,0.107649384406667,-11.1398345295755,2.53927940276412e-15,3.66272126625525e-12,24.621768049805

"P2RY8",-1.794430740425,0.21715700516,-11.1103027662364,2.79851914877707e-15,3.66272126625525e-12,24.5274649198915

"LILRB3",-1.75774965925,0.1199737026,-11.103664831643,2.86038365189789e-15,3.66272126625525e-12,24.5062519322677

"RUNX1T1",1.373971227775,-0.16412019378,10.938001741831,4.94536418736107e-15,5.66157020949632e-12,23.9749174197194

"AFAP1L2",1.0963597157,-0.07894369244,10.9362574359556,4.97404645504363e-15,5.66157020949632e-12,23.9693032764708

"ARG1",-4.8517751290125,0.76358540679,-10.8800009732666,5.99515124571881e-15,6.46466624854142e-12,23.7880207453068

"SLC7A5",-2.616335197075,0.31073461234,-10.8360047482248,6.93965577133258e-15,7.10898337215309e-12,23.645951827052

"SLC22A4",-2.91612492275,0.3803493218,-10.7587991690179,8.97612666946516e-15,8.55252450878544e-12,23.3960243349089

"ZNF287",1.279899134475,-0.17708290042,10.7519506219644,9.18369480638811e-15,8.55252450878544e-12,23.3738162933416

"GLRX",-1.41815592815,0.08310373248,-10.7268522582302,9.9867606884378e-15,8.89603273846581e-12,23.292375892764

"PGD",-2.1611452266,0.22614677372,-10.7036976570643,1.07905194455065e-14,9.21150676664738e-12,23.2171689645503

"UBALD2",-1.02632221515,0.11060382588,-10.6762986250197,1.18265946877206e-14,9.63161261048511e-12,23.1280847249045

"ZNF793",1.162589682675,-0.14984511286,10.6588652206662,1.25376348067241e-14,9.63161261048511e-12,23.0713509657323

"TPTE2P1",1.294051359575,-0.17779535234,10.6551899258993,1.26929685905456e-14,9.63161261048511e-12,23.0593853085962

"C3orf18",1.006169276675,-0.14846853366,10.5547057643507,1.77847844547632e-14,1.27094687331442e-11,22.7315535958008

"NME5",1.044871478875,-0.0843989539,10.5512980328045,1.79897790541381e-14,1.27094687331442e-11,22.7204126798941

"BAZ1A",-1.3229558729,0.09201371068,-10.530695553548,1.92810334911904e-14,1.31676604722503e-11,22.6530246851462

"PPIF",-1.6994604001,0.24477946992,-10.4660344637817,2.39750683863766e-14,1.58452000354866e-11,22.441168879245

"HMGB2",-2.159033751375,0.1622378549,-10.4407410998547,2.61117598390828e-14,1.67180542369727e-11,22.3581503921031

"MACROH2A1",-1.042305817025,0.10983476538,-10.3610793991325,3.4184445667825e-14,2.12233612982545e-11,22.0961446065034

"CRNDE",1.001564714,-0.0331526523,10.3200486178581,3.92841080557757e-14,2.29423960667307e-11,21.9608778458178

"SETBP1",0.768019611675,-0.0976487149933333,10.3144549129505,4.00365297012908e-14,2.29423960667307e-11,21.9424203567538

"C5AR1",-2.483139753275,0.19574700738,-10.3058393497895,4.12240208341395e-14,2.29423960667307e-11,21.913983876503

"KLF12",1.217863938075,-0.11138034884,10.3043528330226,4.14324802064153e-14,2.29423960667307e-11,21.9090765314965

"ASIC1",1.565904604725,-0.17154496622,10.1814913700157,6.29202766178783e-14,3.3923963877555e-11,21.5025136286145

"GLRXP3",-1.680869176075,0.11502877514,-10.1416776041629,7.20704861566622e-14,3.78610287276332e-11,21.3703573040115

"PRRT2",2.239944441075,-0.14336301714,10.1314474987781,7.46314815279012e-14,3.8226244838591e-11,21.3363678400881

"ZNF135",1.2026775457,-0.07462878144,10.0796294703517,8.90875170958365e-14,4.45176841526707e-11,21.1640023718872

"HECW2",1.855753080925,-0.17491920126,10.0046386811765,1.1517034828188e-13,5.6181192752361e-11,20.913966604445

"MAP1B",1.397833384525,0.02802370562,9.92186780982984,1.53025738065366e-13,7.17580110254631e-11,20.637188325084

"S100A9",-5.548115349,1.0061790108,-9.91470299847711,1.56842751691817e-13,7.17580110254631e-11,20.6131905614897

"AC120036.4",1.351816149225,-0.17282807262,9.91328437289786,1.57609844599075e-13,7.17580110254631e-11,20.608438288745

"UGCG",-1.212377806425,0.000809932860000003,-9.90523958291032,1.62032115364956e-13,7.21676952086353e-11,20.5814843213088

"ISG20",-1.427942514125,0.1330401427,-9.8945825567225,1.68083909115771e-13,7.32702793609345e-11,20.5457660109042

"HACD1",-1.025917766875,0.0480128465,-9.88464567440328,1.73932263326875e-13,7.42400877300212e-11,20.5124489648752

"KIAA1671",1.192983693575,0.00328557186,9.79292383644886,2.38627431468453e-13,9.90371052413863e-11,20.2043566120537

"PRR13",-0.89695134215,0.04924333628,-9.78922444668431,2.41695395454379e-13,9.90371052413863e-11,20.1919092795539

"GYPC",-1.340844684725,0.15338255222,-9.73708141696319,2.89427040133379e-13,1.16270219573582e-10,20.0162906783086

"CSRNP3",1.52038809451667,-0.168360541386667,9.73117169549862,2.95405401184079e-13,1.16389728066527e-10,19.9963663195623

"PLXDC1",1.810014465275,-0.220997711046667,9.72263274170305,3.04264421761457e-13,1.17618291944316e-10,19.9675703425896

"IQCD",1.03399487345,-0.03221457424,9.71300469560593,3.14575506689652e-13,1.19110884899845e-10,19.9350913038561

"ABHD5",-1.54584571215,0.15734961728,-9.70828968091593,3.19752961220786e-13,1.19110884899845e-10,19.919181796777

"ADAMTS10",1.543616199525,-0.01029384638,9.69751493642535,3.31909686476965e-13,1.21431529581072e-10,19.8828156057406

"PPFIBP1",0.9265085101875,-0.09834797335,9.6597755422398,3.78289892003858e-13,1.33834860986986e-10,19.7553328590364

"EFS",0.93765627475,-0.1074698422,9.65932873259798,3.78876510017825e-13,1.33834860986986e-10,19.7538225500064

"SRPK1",-1.003020337125,0.0768786053,-9.65361348310512,3.8646156390914e-13,1.34200415616448e-10,19.7345017714946

"CCDC113",1.42484808105,-0.22028426016,9.60268832539926,4.61192066111531e-13,1.57481717508217e-10,19.5621783424305

"ZNF30",1.002145143275,-0.04225169338,9.59515704209868,4.73420030459074e-13,1.59007042361402e-10,19.5366680809482

"CYTH3",0.76972493225,-0.0088201692,9.58679551537191,4.87379709011291e-13,1.61055410939086e-10,19.5083379261282

"TM4SF18",1.490851966125,-0.1354591301,9.5593777695123,5.3613586998393e-13,1.71972299956478e-10,19.415385937209

"FAM218A",1.1023675225875,-0.05687545593,9.55880589814287,5.37203592210786e-13,1.71972299956478e-10,19.4134462535304

"C1orf162",-1.923176874975,0.12832042002,-9.50534399220412,6.47117260446247e-13,2.03971360492657e-10,19.2319483407122

"SPIN3",1.159852725925,0.00429137623999999,9.43663370038098,8.22398094424611e-13,2.55292305432901e-10,18.998208406155

"SIGLEC12",-2.789647911125,0.0395944311,-9.40676914294297,9.12843984225493e-13,2.76254798155671e-10,18.8964496235412

"ECM2",1.43141589738333,-0.191129014093333,9.40352134275617,9.23266400740084e-13,2.76254798155671e-10,18.885377270507

"TBC1D19",0.955966268575,-0.02695676714,9.40132645618427,9.30377834475854e-13,2.76254798155671e-10,18.8778938320704

"CLEC1B",-3.92600088884167,0.31741790716,-9.38526773046573,9.84117136795626e-13,2.8803702712384e-10,18.8231255595017

"MIR99AHG",1.28063872676667,-0.10963415192,9.37173896743714,1.03181374391378e-12,2.97743661764866e-10,18.7769635870138

"CD34",1.1297122134125,-0.03904881827,9.35800539034353,1.08261879450677e-12,3.05923605479057e-10,18.7300821208155

"THSD1",1.083482335425,-0.03060531166,9.35605793275309,1.09002456071706e-12,3.05923605479057e-10,18.7234325248751

"ALOX5AP",-2.094242522575,0.15331872694,-9.34574124680443,1.1301175846285e-12,3.12889852349578e-10,18.6881992431887

"HNRNPAB",-1.48398332195,0.09915338644,-9.29349359080539,1.35720212114523e-12,3.64867021324822e-10,18.5095857706632

"SERPINB1",-1.57924011175,0.2149511166,-9.29231302207547,1.362833432175e-12,3.64867021324822e-10,18.5055464566441

"SNRPB",-1.1621924142,0.06050418864,-9.2873651589818,1.38669220593646e-12,3.64867021324822e-10,18.4886157094521

"S100P",-4.9933951767,1.01404669864,-9.28687314015602,1.38908764463765e-12,3.64867021324822e-10,18.4869319597705

"CMYA5",1.2462170806,-0.13601125652,9.27483304844569,1.4490244212971e-12,3.75792561310568e-10,18.4457211137438

"ANO1",1.4134799773,-0.02246792816,9.24621314239738,1.60217980876752e-12,4.10318249025361e-10,18.3476981756116

"ADIPOR1",-0.7965094240875,0.04734944223,-9.22118104404823,1.74947017239353e-12,4.30028968320525e-10,18.2618914914836

"BBS9",0.9526882067125,-0.04578946713,9.22061380683133,1.75296152805542e-12,4.30028968320525e-10,18.2599463035868

"PRR5L",1.1410059964,-0.10060791988,9.21957865971,1.75935099591201e-12,4.30028968320525e-10,18.2563964558807

"GADD45A",-2.219684824325,0.34819679054,-9.21897273347865,1.76310197866674e-12,4.30028968320525e-10,18.2543184897858

"CCDC30",1.43542509,-0.2316027734,9.20621841086258,1.84395608788563e-12,4.44458497983539e-10,18.2105697016939

"AC104083.1",1.740935351975,-0.13811028642,9.18703929076881,1.97263405804583e-12,4.64062309000377e-10,18.1447506543466

"NASP",-0.669837826925,0.06279268246,-9.18256976303132,2.00389988303855e-12,4.64062309000377e-10,18.1294065094444

"LIN7A",-2.699637522375,0.0857189321,-9.18225560806012,2.00611620254683e-12,4.64062309000377e-10,18.1283279181563

"IER5L",1.66156530815,-0.19704711748,9.18087443074349,2.01588956955455e-12,4.64062309000377e-10,18.1235857853336

"PRRT4",-2.911542910675,-0.05677480854,-9.17530338057533,2.05580071230744e-12,4.67715193507173e-10,18.1044561066982

"FGR",-1.8626032043,0.22109744956,-9.17130496998398,2.08493562048829e-12,4.67715193507173e-10,18.0907244892619

"CCDC65",1.6692367876,-0.20036774992,9.16698665348099,2.11686934511294e-12,4.67715193507173e-10,18.0758923407495

"KLK1",1.9118758549,-0.06924009608,9.16615541470036,2.12307267650171e-12,4.67715193507173e-10,18.073037054883

"SVEP1",1.1031745035375,-0.03305151942,9.14359390435934,2.29862789290564e-12,5.0100306670054e-10,17.9955111328212

"GCA",-2.1464321007,0.22262608144,-9.13240513082093,2.39104708180956e-12,5.15660764338045e-10,17.9570445085143

"YDJC",-0.789873700175,0.06217014286,-9.11786643770858,2.51675684888749e-12,5.37117857500072e-10,17.9070415131088

"RANGAP1",-0.882284504075,0.13797247074,-9.10081279293182,2.67273223488239e-12,5.64525134312066e-10,17.8483609076702

"ADGRA3",1.1029251265,-0.1218689678,9.05549614673277,3.13624054060938e-12,6.55666287714337e-10,17.6922834673016

"ISLR",1.29845691845,-0.08360681624,9.04581781412238,3.24529424554375e-12,6.71612005077781e-10,17.6589225986255

"FCAR",-2.5219488265625,0.20157059175,-9.03751216648328,3.34192368131443e-12,6.84693323827701e-10,17.6302857340582

"RARG",1.2318722696,-0.15259136032,9.03019847696474,3.42941103996007e-12,6.95661122640614e-10,17.6050632281747

"PYGO1",0.86822911145,-0.00496412334,9.02059485625918,3.54780659221327e-12,7.12622171188877e-10,17.5719352856795

"SLIT2",1.590718604275,-0.02594072058,9.01402107364134,3.63121646177677e-12,7.22294785134782e-10,17.5492534821485

"SSPN",1.125120381625,-0.1892574527,9.00584460808218,3.73772502867148e-12,7.36331830648282e-10,17.5210358005384

"SIGLEC10",-1.35865971285,0.06930710772,-8.97238966964243,4.20733981075086e-12,8.20952171834892e-10,17.4055101521268

"CXCR4",-1.894965769975,0.16959648402,-8.96609150023365,4.30218748037102e-12,8.31539783941901e-10,17.3837490042214

"CEMIP",1.6105114466375,-0.00412543869,8.95888881600044,4.41330093167073e-12,8.45044013907196e-10,17.3588577908476

"ETFBKMT",1.10667737155,-0.17097548426,8.95076312257066,4.54212905617217e-12,8.60156791987659e-10,17.3307706503802

"ALDH7A1",0.791351814958333,-0.0155419993666667,8.94661641173848,4.60932631500727e-12,8.60156791987659e-10,17.3164346812005

"CYS1",1.667382515225,-0.09371438182,8.94607467545841,4.6181787933738e-12,8.60156791987659e-10,17.3145616703077

"FXN",-0.5952285004,-0.01434989332,-8.93099253452564,4.87163391873388e-12,8.97477283469148e-10,17.2624047364086

"DHCR7",-1.121431368375,0.0885876453,-8.92806122173824,4.92249816335519e-12,8.97477283469148e-10,17.2522650985958

"MRAP2",2.021145922375,-0.1744166571,8.92649082883336,4.94996744592023e-12,8.97477283469148e-10,17.2468326410862

"MBL1P",1.54560342485,-0.06604848512,8.92049389570049,5.0562943153763e-12,9.05636238115368e-10,17.2260852360545

"MCEMP1",-2.691206548525,0.14056828118,-8.91734572379438,5.11303012341407e-12,9.05636238115368e-10,17.2151921929543

"MINDY2",0.9273535032,-0.10052681144,8.91654419504125,5.12757729506944e-12,9.05636238115368e-10,17.2124186548569

"ZNF781",1.181940638625,-0.0694056781,8.91279969115367,5.19609175889545e-12,9.0989340133547e-10,17.1994606780584

"LRG1",-2.019602907475,0.18241425002,-8.88484612430795,5.73758191705113e-12,9.96199816241894e-10,17.1026833024985

"DDX39A",-1.020406887825,0.17535186874,-8.86313137178865,6.19718266974061e-12,1.06263243678968e-09,17.0274529515603

"CDC14B",0.959113809475,0.09979161248,8.85872994216246,6.29476935467026e-12,1.06263243678968e-09,17.0121987482337

"FAM229B",1.122502576275,-0.13989606198,8.85725403696864,6.32783790755404e-12,1.06263243678968e-09,17.0070832307529

"CLIP3",0.9935749323,-0.05284398916,8.8567408450777,6.33937724130825e-12,1.06263243678968e-09,17.0053044482464

"FAM76A",0.713891163958333,0.0904384076666667,8.85496247097989,6.37952897916493e-12,1.06263243678968e-09,16.9991402023854

"NUP98",-0.9250447316,0.00682624297,-8.85134882822063,6.46190793809606e-12,1.067673950288e-09,16.986613567847

"GYS1",-0.88392699085,0.06046018632,-8.83820343499124,6.77072337844837e-12,1.1097486446212e-09,16.9410347417023

"FPR2",-1.657006380775,0.00562367337999999,-8.83591391523021,6.8260102038528e-12,1.10993092902013e-09,16.9330946381649

"RNF10",-0.7394401839,0.06540309988,-8.82348251260294,7.13423702536451e-12,1.15091533996589e-09,16.8899735654463

"DPYSL3",1.38027592435,-0.15761894352,8.79984367641586,7.75956194766619e-12,1.24201488424832e-09,16.8079366587122

"MYLK",1.337784437975,-0.09720790962,8.78855670903857,8.07733189855901e-12,1.28285562742385e-09,16.7687474762251

"GUCY1A1",1.4388697221,-0.0917245196866667,8.77579799974326,8.45235119968709e-12,1.33209054907069e-09,16.7244339323352

"GLI1",2.382373840275,-0.30632703978,8.77283407540536,8.54195161711105e-12,1.33593515062115e-09,16.7141374565232

"CX3CR1",2.585862528325,-0.30536643434,8.75518253032983,9.0957330240472e-12,1.41176801664151e-09,16.6528002476779

"ZNF436",1.3293906472875,0.15795280383,8.74616586700306,9.39242446688933e-12,1.44685708629796e-09,16.6214571801425

"EPHA4",1.075515656875,-0.1059465695,8.73981667419889,9.60717340568429e-12,1.46889379653477e-09,16.5993820781576

"TET1",1.002471570075,-0.04937306494,8.72382899237683,1.01700876596691e-11,1.54344263682445e-09,16.5437791188138

"HRH4",-2.5586557709,0.21802144728,-8.71775796859969,1.03924436822147e-11,1.56559107471482e-09,16.5226587999469

"IL17RA",-1.11375401165,0.110116787513333,-8.69878228424681,1.11195100467588e-11,1.66289431998535e-09,16.4566231561723

"RNF144B",-1.08921165025,-0.0328293412,-8.67737698975105,1.20013565451052e-11,1.78176661518924e-09,16.3820932874148

"PRDX6",-1.0443989026,0.08093519192,-8.67257168263198,1.22088126775803e-11,1.7995262887645e-09,16.3653562925371

"RNF207",1.31924843739167,-0.113593470953333,8.66234631220514,1.26623622705662e-11,1.85304627285258e-09,16.3297342051687

"ZNF503-AS2",1.0977902365,-0.1480843668,8.64647970608877,1.33999545377326e-11,1.94707991892954e-09,16.2744412874199

"MTMR9LP",1.2790539754375,0.03932041035,8.57956541342182,1.70178222191109e-11,2.45536015228974e-09,16.041010128244

"AC040160.1",1.1805693454625,-0.11108537363,8.57685540888444,1.71834888618778e-11,2.46192531330176e-09,16.0315480324094

"LRRC7",2.05701964671667,-0.0231758532933333,8.56175307448654,1.81369005864051e-11,2.58047791121019e-09,15.9788060131811

"MED17",0.66402049075,-0.0636966134,8.51030724328541,2.18028236000612e-11,3.0806637925383e-09,15.7989954003444

"PTPRD",1.82972824075,0.0111182246,8.49960878041422,2.26542983991621e-11,3.16481708884238e-09,15.7615747233486

"DCLK1",1.18953808855833,-0.08909429782,8.49895570740029,2.27073463520027e-11,3.16481708884238e-09,15.7592901200801

"TLR3",1.201807499625,-0.0581561093,8.49216017413024,2.32668087181834e-11,3.22088092579825e-09,15.7355156377723

"PRKG1",1.148647729625,-0.1550111293,8.48722126271444,2.36821051694726e-11,3.25636893095406e-09,15.7182342249312

"RAB20",-1.4941254574,0.11655951508,-8.48347727135718,2.40018888832795e-11,3.25719434617307e-09,15.7051325298648

"HOXC4",0.787147683425,0.01554788774,8.48342866148083,2.40060692245282e-11,3.25719434617307e-09,15.7049624172042

"BMP3",1.652360769425,-0.10373280096,8.46713060690046,2.5449792725975e-11,3.43036416690643e-09,15.6479155846097

"ZNF284",0.746927998575,-0.03745420414,8.46386932870484,2.57490136483528e-11,3.44801170998335e-09,15.6364977598861

"LBR",-1.225892828725,0.11197162702,-8.45631254833656,2.64560217112453e-11,3.51518234234339e-09,15.6100379175524

"GRK6",-1.093303566725,0.09498267872,-8.45485488459007,2.65946310500284e-11,3.51518234234339e-09,15.6049334143697

"UNC5B",1.663588971425,-0.13947583986,8.4530700381799,2.67653477843405e-11,3.51518234234339e-09,15.5986829342831

"LMCD1-AS1",1.532484817475,-0.14336300202,8.43880618423001,2.81698797977843e-11,3.67607960061786e-09,15.5487220243711

"DNALI1",1.30218761035,-0.17478093772,8.43477304070921,2.85803010267611e-11,3.69508473315603e-09,15.5345924499361

"PPFIBP2",1.371155661475,-0.06839176382,8.43383882593431,2.86762237686357e-11,3.69508473315603e-09,15.5313193669442

"PMEPA1",1.165858026475,-0.16383738482,8.4283402857002,2.92473999849697e-11,3.74512956807537e-09,15.5120534423288

"ZC3H12B",0.981188358,-0.0768729256,8.42234945309957,2.98827534700355e-11,3.80271958443533e-09,15.4910598404655

"FGD6",1.165607650775,-0.15934036238,8.41824779213083,3.03257432796172e-11,3.83527054514072e-09,15.4766847777006

"NT5E",1.086919001525,-0.02363107293,8.41356113521752,3.08400083768445e-11,3.87638093021343e-09,15.4602578340407

"SYNJ2BP",0.599838132775,-0.04278383178,8.39998521648458,3.23797522694906e-11,4.004619433736e-09,15.4126637341439

"DLG3",0.911244227825,-0.13100241614,8.39975203950412,3.24068634521175e-11,4.004619433736e-09,15.4118461403171

"ZNF575",1.120058145875,-0.0800917403,8.39941025808258,3.24466432057876e-11,4.004619433736e-09,15.4106477364345

"ORM1",-2.333815064175,0.19488051166,-8.39645340250532,3.27928474274657e-11,4.02311292271807e-09,15.4002795912207

"TRIM47",1.1543393684,-0.14715238828,8.38583149224991,3.40674592200956e-11,4.1546077648888e-09,15.3630284038257

"ETV1",1.2667137487,-0.06429956154,8.36472000597865,3.67507234578065e-11,4.45531847457715e-09,15.2889637792077

"APLNR",1.667910595775,-0.15485220838,8.35639199413324,3.78668005845e-11,4.56361770808962e-09,15.2597373566659

"REEP2",1.33488901845,-0.00654775224000001,8.35205783195618,3.84610686325464e-11,4.60813084294509e-09,15.2445248664339

"PA2G4",-0.959533953475,0.11220291022,-8.34119741494157,3.99917062924207e-11,4.76366324720416e-09,15.206399472912

"RAB40B",1.007827853825,-0.08194958794,8.33920426742341,4.02791993429628e-11,4.77017477536776e-09,15.1994015595813

"LRIG3",1.1011459329,-0.14345768868,8.3291494863345,4.1761517401323e-11,4.91729866964543e-09,15.1640947128496

"NBPF3",1.204690867125,-0.0359214883,8.32532112689216,4.234022696044e-11,4.94122283328767e-09,15.1506495914256

"RBFOX2",0.88352875765,-0.02875362538,8.32462019527226,4.24470528435489e-11,4.94122283328767e-09,15.1481878124853

"TCN1",-4.161114264625,0.8104756433,-8.32002540603869,4.31540693508143e-11,4.99514447943211e-09,15.132049283474

"RNASE2",-4.9352190295,0.7332581104,-8.31079679319449,4.46101006722817e-11,5.13467271108824e-09,15.0996302747286

"KLF3-AS1",1.567122537575,-0.10475696994,8.30780494305087,4.50926630514787e-11,5.16122056200388e-09,15.0891188792837

"CLIP2",0.9060319930625,-0.04612849155,8.30058574669441,4.62787946741805e-11,5.26755525158116e-09,15.0637525844379

"PRTFDC1",1.2231538998,-0.0151524174266667,8.29339142280035,4.74920607893698e-11,5.37578641686525e-09,15.0384697831426

"DISP1",0.7367452341,-0.05255897622,8.28063964418773,4.97216953095642e-11,5.59724227199095e-09,14.9936470611474

"LIMK2",-0.912264825775,0.06186954658,-8.27857530274066,5.00924175436266e-11,5.60816093242526e-09,14.9863897252496

"PFN4",0.887178500125,-0.0939932629,8.27464845826196,5.08053058676898e-11,5.63364422099326e-09,14.9725837566497

"PTCH1",1.258174862375,-0.0385529364333333,8.27429494743061,5.08699814956927e-11,5.63364422099326e-09,14.9713408299487

"AC007773.1",1.416570147525,-0.03317153798,8.27189842965152,5.13106186692277e-11,5.65189223276955e-09,14.9629145471136

"EPHB3",1.6263321477,-0.12757016184,8.26921061729925,5.18093832622982e-11,5.67631360576453e-09,14.9534635514616

"PLD2",0.936703396625,-0.0824677847,8.2547697882079,5.45737754076027e-11,5.94738037527109e-09,14.9026769987395

"SBK1",1.489986441075,-0.00221599614000001,8.2470910136307,5.6103638046239e-11,6.0817531020706e-09,14.8756654971201

"TRPC1",0.917130295625,-0.1750369085,8.24479695166097,5.65690030924272e-11,6.09992492293500e-09,14.8675948769772

"LDLRAD4",1.021507627425,-0.02150697706,8.23907546958275,5.77466258811672e-11,6.19430822541022e-09,14.8474647591622

"ZBED8",1.262724933925,-0.10631675586,8.23447287647513,5.87118224516475e-11,6.26504072077789e-09,14.8312695477757

"N6AMT1",0.864646507125,-0.0318004993,8.22379762289505,6.1013415020609e-11,6.47690594270589e-09,14.7937004815076

"RIBC1",0.77512567425,-0.0291926803333333,8.2216573074099,6.14856755629076e-11,6.49339443779819e-09,14.7861671520321

"LDOC1",0.753194150675,-0.09085040046,8.21633966965951,6.267498425056e-11,6.58505167859217e-09,14.7674490873896

"ID1",1.50404178405,-0.10287113076,8.20930568324744,6.42837723076569e-11,6.71962207673099e-09,14.7426863731375

"FAM201A",-3.7399853514,0.52277530188,-8.20543394418023,6.51869536768565e-11,6.77944318239307e-09,14.7290546493103

"NT5C3A",-0.8199838583625,0.06342419531,-8.18788906271076,6.94423541321387e-11,7.18553005787504e-09,14.6672688342227

"TEX2",-1.060828324575,0.03169547134,-8.18197045816511,7.09400006088264e-11,7.28973520651112e-09,14.6464210623753

"TUBA4A",-1.80218459345,0.21923151324,-8.18110764405104,7.11610231014362e-11,7.28973520651112e-09,14.6433816692228

"BDNF-AS",1.250869181425,-0.03067110186,8.1752099925666,7.2690446372872e-11,7.40936251386767e-09,14.622604905997

"NDST2",-1.026261299325,0.06307566654,-8.17193339795331,7.35543934857733e-11,7.46030897889368e-09,14.6110607825497

"OLFML2A",1.2307405181625,-0.10466680747,8.16809152353131,7.45805469290294e-11,7.52712436198007e-09,14.5975241092876

"NRARP",1.2443530425125,-0.06759531749,8.16330356491039,7.58795656265471e-11,7.59015400329414e-09,14.5806525164704

"BMX",-1.2695483506,0.01534801552,-8.16191107691342,7.62616131270602e-11,7.59015400329414e-09,14.5757454319798

"CEP126",1.869747044825,-0.23684295414,8.1617117303488,7.63164645001265e-11,7.59015400329414e-09,14.5750429298982

"LINGO1",1.4311103775875,-0.11205875793,8.15491029504611,7.82118714776532e-11,7.7410861006481e-09,14.551072864907

"TLR7",2.2060811425,-0.262266064,8.14856686765974,8.00222731898906e-11,7.88219390920423e-09,14.5287140716544

"MED12L",-1.7732073082,0.04703315494,-8.14580437016786,8.08237959845073e-11,7.92305230684491e-09,14.5189761840502

"CAPN2",0.738810923075,-0.05856607454,8.1364279676374,8.36049781765595e-11,8.15666091848263e-09,14.4859201927067

"JHY",1.1257839209,0.07433322127,8.13337076484993,8.45324307377713e-11,8.20805896187421e-09,14.4751408985716

"LMO7",1.42883248213333,0.0445987502066667,8.12909572611584,8.58467024456738e-11,8.29635490427813e-09,14.4600666124396

"OAZ1",-0.798998050675,0.00541693146,-8.12358594453531,8.75709128434697e-11,8.42325287482163e-09,14.440636655892

"ZNF608",1.0187100310125,-0.01704851669,8.11653807905977,8.98272868764006e-11,8.58507187036715e-09,14.4157797459592

"S100A8",-5.43885812755,0.85760538796,-8.11572509461651,9.00912950082457e-11,8.58507187036715e-09,14.4129122403922

"KMT5B",0.601575406,0.0458872381333333,8.11135606785997,9.15235063861312e-11,8.68117406869933e-09,14.3975013368304

"NOTCH2NLA",1.42820643245,-0.10410394304,8.10957528972171,9.21138081736974e-11,8.68647381235227e-09,14.3912196175399

"ODC1",-1.12156507525,0.0116131038,-8.10738490843334,9.28451384392782e-11,8.68647381235227e-09,14.3834927273055

"LRRN3",1.2102003714,-0.11385635188,8.10688823483179,9.30117791794115e-11,8.68647381235227e-09,14.3817405940792

"RAB7B",2.269400701425,-0.30300514286,8.10610464781962,9.32752947441185e-11,8.68647381235227e-09,14.3789762727444

"FZD8",2.04101070285,-0.29841639172,8.10426049233356,9.38984380583991e-11,8.70493755176688e-09,14.3724703395906

"CCDC89",1.2806798493,-0.05577243156,8.09987295776997,9.53978658763481e-11,8.80410574808387e-09,14.3569907978564

"MAMDC2",2.099356617625,-0.0136004842,8.09830623457497,9.59391006207467e-11,8.81435109200833e-09,14.3514629748637

"ZC2HC1A",1.4662972401375,-0.15483673239,8.09688263667415,9.64335676147951e-11,8.82022738076751e-09,14.3464399956722

"BCL2A1",-2.65994420695,0.06366774444,-8.09514672277751,9.70399805505967e-11,8.83624498453611e-09,14.3403148689269

"CUBN",1.4118743727375,-0.09841392631,8.08843833306013,9.94196904594891e-11,9.01287884130094e-09,14.3166426225325

"FBN1",1.1235774849,-0.05755638248,8.08220174897017,1.01684618919967e-10,9.17759679485584e-09,14.2946326072242

"CYTIP",-1.451924699975,-0.01170007858,-8.07128798637577,1.05773656408269e-10,9.50478365128339e-09,14.2561098780601

"RCOR1",-0.84209709155,-0.02510427624,-8.06970095512234,1.0638187491959e-10,9.50628686358493e-09,14.2505074270031

"IFT172",0.866091980625,-0.0914384865,8.06882684644744,1.06718370686477e-10,9.50628686358493e-09,14.2474216265209

"RGS11",1.665295449375,-0.0402456455,8.05688667522993,1.11423401174541e-10,9.88243568512551e-09,14.2052651878982

"SKIDA1",1.2868695556375,-0.08679994649,8.05246967574221,1.13216269282138e-10,9.99816778039845e-09,14.1896680303694

"BID",-1.0497767228375,0.08696933823,-8.03794433577584,1.1931911253887e-10,1.04918883162935e-08,14.1383678594604

"EML1",1.04548038765,-0.03790691338,8.03176263143033,1.22015645992044e-10,1.06831476713034e-08,14.1165314345664

"KCTD1",0.7065777940625,-0.0867295356,8.02976135539304,1.22901682294472e-10,1.07149347525495e-08,14.1094615545404

"CCDC36",1.12544838975,0.0113205763,8.02852308870898,1.2345314193053e-10,1.07174066604776e-08,14.1050870207798

"RHOU",-1.46372271895,0.04433029984,-8.0244090566869,1.25303234049146e-10,1.08321209248899e-08,14.0905523257684

"HMOX1",-1.569332881525,-0.14958375922,-8.01963185228181,1.2748654508245e-10,1.095753444589e-08,14.073673340791

"TLR2",-1.5211285496,0.11016550132,-8.01807674873622,1.28205484513413e-10,1.095753444589e-08,14.0681784870478

"HNRNPA2B1",-0.6261187458875,-0.09077979946,-8.01774697093761,1.28358466761695e-10,1.095753444589e-08,14.0670132199939

"TRDMT1",0.7351293519,-0.0586349564466667,8.01194561661222,1.31079881345889e-10,1.11434216141684e-08,14.0465130762937

"GLUL",-2.265571694075,0.22434742004,-8.00534031234926,1.3424908674095e-10,1.13258197238519e-08,14.0231694859056

"CHPT1",-0.996594240425,0.01849714266,-8.0051716051784,1.34331032452948e-10,1.13258197238519e-08,14.0225732280496

"SAP30",-1.52814049625,0.191974772,-8.00165805005187,1.36049131956798e-10,1.14236664570938e-08,14.0101549522918

"RNASE3",-4.832536591725,0.75923943662,-7.99371783475492,1.40013603559786e-10,1.17085661621751e-08,13.9820883345417

"FAM225B",1.5558973252,-0.01546002984,7.98503400587527,1.44482375785778e-10,1.20331500613781e-08,13.9513888190649

"ZFHX4",1.108392006775,-0.09051885758,7.97998565183909,1.47145886059483e-10,1.22053640226182e-08,13.9335395121248

"LINC01546",1.359885016,-0.0882857972,7.97805247442627,1.48178843347062e-10,1.22414844455427e-08,13.9267040287892

"MYOF",0.697676737241667,-0.06135110934,7.97658704663127,1.4896671900827e-10,1.22571491527768e-08,13.9215223015287

"SOD2",-1.664712967,0.2230315584,-7.97485379224424,1.49904024133868e-10,1.22849345858188e-08,13.9153933768528

"MATN2",1.1547475398,-0.06382461516,7.97011653361251,1.52496166161718e-10,1.24475755072561e-08,13.8986411328594

"CHST11",-0.945394211075,0.00992466014,-7.96216886792997,1.56946537062019e-10,1.27168333797348e-08,13.8705329879559

"XBP1",-1.26365212475,0.0889330482,-7.96201097952424,1.57036257568962e-10,1.27168333797348e-08,13.8699745530399

"CAPN5",1.333570315925,-0.02887810626,7.95083735109226,1.63518323491953e-10,1.31896197311147e-08,13.8304508263113

"AOPEP",1.2683887833,-0.08407604336,7.94943308375785,1.64351759421325e-10,1.32048582236239e-08,13.825483082722

"FLRT3",2.530199118675,0.06988082494,7.9336823562962,1.7399739163553e-10,1.38796886625938e-08,13.7697552904727

"HDAC5",0.650685858625,-0.1045192151,7.93351033381218,1.7410581737049e-10,1.38796886625938e-08,13.7691465761171

"IGIP",0.989378650125,-0.0699739859,7.93147654770081,1.75392868482025e-10,1.3928097245968e-08,13.7619497422687

"AQP9",-2.583141477325,0.28603550814,-7.9251453358322,1.79460988353636e-10,1.41961263682985e-08,13.7395443364357

"HAUS2",-0.744679231925,0.04899581996,-7.9135486762408,1.8715982980272e-10,1.47481945884543e-08,13.6984991438499

"DHFR2",0.9069808249,-0.14723521143,7.90742623564637,1.91357354350471e-10,1.5021185731542e-08,13.6768262852595

"FAM198B-AS1",1.425494868375,-0.0310777923,7.90188290925876,1.95239351562676e-10,1.52093681932172e-08,13.6572015978158

"ZBTB46",0.9626129628,-0.05148093176,7.88698252740778,2.06070721473104e-10,1.59923368997763e-08,13.6044421298275

"TRABD",-0.921890252925,0.04152723466,-7.88004451996288,2.11318052785586e-10,1.62337587322214e-08,13.5798717051779

"H2AC7",-1.59125403845,0.07138098024,-7.87978609235707,2.11516077376105e-10,1.62337587322214e-08,13.5789564518578

"CANX",-0.8378451870375,0.04573713207,-7.87973056815903,2.11558648062432e-10,1.62337587322214e-08,13.578759805562

"LRRC49",0.82259537535,-0.06678648972,7.87814464329275,2.12778224448961e-10,1.62664188899638e-08,13.5731429706664

"GLI3",1.0437089678,-0.11180025076,7.87349420110237,2.16395276180205e-10,1.6481436499554e-08,13.5566718012481

"ZEB1-AS1",0.6326065421625,-0.00442177752,7.86916060438224,2.19821454347853e-10,1.66803776173289e-08,13.5413217785633

"NME3",0.658375432275,0.01812528582,7.86810546181339,2.20663873421075e-10,1.66825145337674e-08,13.5375842044414

"SLC29A1",0.910098497975,-0.00140880362,7.86077594366434,2.26605885736583e-10,1.70687550991585e-08,13.5116195703541

"MAPKAPK3",-1.078617403425,0.06825870526,-7.85690088705653,2.2981204464193e-10,1.7246846778842e-08,13.4978910968239

"PLA2R1",1.318691344575,-0.06697647034,7.85573576357021,2.30784927489387e-10,1.72566481547539e-08,13.4937631611591

"SERTAD4-AS1",1.135352356975,-0.06546055942,7.85124533680114,2.34573296564834e-10,1.74737725674138e-08,13.4778532654289

"ANKAR",1.181239934675,-0.10375493126,7.85009384704983,2.35554775814306e-10,1.74737725674138e-08,13.4737732814593

"CCDC171",1.2534784728625,-0.17342240171,7.84928424201738,2.3624731555904e-10,1.74737725674138e-08,13.4709046282296

"SATB2-AS1",1.8054089104,0.06398597832,7.84679425741142,2.3839010005155e-10,1.75552574422364e-08,13.4620817071943

"TMEM35A",0.982810261475,-0.09002081282,7.84601729444025,2.3906271116673e-10,1.75552574422364e-08,13.4593285766418

"PCNX2",0.9920678913,-0.04269911406,7.82879283843405,2.5447316424646e-10,1.86201649610053e-08,13.398286281562

"FAT4",0.865518111475,-0.02759987012,7.82612896530255,2.56943991597233e-10,1.87340516008687e-08,13.3888442935065

"MXRA8",1.438158612975,-0.03598863262,7.79957114374186,2.8293377060131e-10,2.05558407520555e-08,13.2946909524417

"IL18RAP",-1.6828012649875,0.08309442871,-7.79474636905532,2.87931230656716e-10,2.08450001897343e-08,13.2775821577092

"ZNF711",1.1707589278125,-0.02441533775,7.78905831524858,2.9393682079076e-10,2.12048506491588e-08,13.2574106279095

"TAS2R10",1.0612461735,-0.0557887812,7.78800861499429,2.95058789560695e-10,2.12111034404194e-08,13.253687899511

"CBLN3",1.39442631105,-0.08989822016,7.7844265142495,2.98919977509683e-10,2.13616364306336e-08,13.2409836748093

"ZFP2",1.1090414907,-0.06192861544,7.78413348411487,2.99238073779376e-10,2.13616364306336e-08,13.239944389915

"CDC42",-0.7788240805,0.1164880613,-7.78151668073698,3.02093832251774e-10,2.14906195665776e-08,13.2306632273109

"ZNF684",0.7486481312625,0.04228050801,7.78025525537396,3.03480203207573e-10,2.15145411879473e-08,13.2261891367383

"DDR2",0.87359883655,-0.07469099976,7.77877871779038,3.05111109775016e-10,2.15555738519674e-08,13.2209519733944

"AMOT",0.938965935541667,0.0278964390333333,7.77171205516632,3.13039241332377e-10,2.20396837677586e-08,13.1958855661976

"S100A12",-5.815839116825,1.10393072154,-7.75009338815669,3.38600808391764e-10,2.37577169942824e-08,13.1191859186573

"SMO",1.0005010371,-0.13287906532,7.74433621351736,3.45754521291607e-10,2.41768554000766e-08,13.0987565214306

"TFDP2",-1.131018229675,0.08331817526,-7.73625730633339,3.56049725506648e-10,2.48120638645585e-08,13.0700857420255

"NAB1",0.6949930677625,-0.00690246048999999,7.73351708844916,3.59611105709603e-10,2.49752960467063e-08,13.0603604282613

"EREG",-2.351184575375,0.1890817112,-7.73217526280017,3.61368056396965e-10,2.50125295252061e-08,13.0555980213792

"NPR2",0.799173714625,0.0177426337,7.73108228215526,3.62805541632165e-10,2.50274745352182e-08,13.0517187518734

"CTSO",1.026888754725,-0.02384327022,7.71612818311969,3.83059481684802e-10,2.63359820830813e-08,12.9986371486299

"GRPEL1",-0.9883185857,0.08247211364,-7.71474365935132,3.84991150763159e-10,2.6380263200119e-08,12.9937220639486

"AL078621.1",-0.8830450744,0.01859836548,-7.712197485078,3.88569097887998e-10,2.65366789250977e-08,12.9846828661361

"TRPM4",1.056625184,0.0368476762,7.71059859169091,3.90832942081449e-10,2.66026090277898e-08,12.9790064656299

"FARSA",-0.924260134825,0.04641170314,-7.70282219026456,4.02033599912994e-10,2.7274385413965e-08,12.9513969579667

"DLX6",1.261959435075,-0.06969013654,7.69647322241503,4.1141675631313e-10,2.78188333443677e-08,12.9288533855684

"TBC1D4",0.88228118055,-0.07961816856,7.6929642657624,4.16696635235783e-10,2.80831600747063e-08,12.9163931841839

"ZNF521",0.6982837195625,-0.08335155585,7.68835785396872,4.23731212750944e-10,2.84636232355454e-08,12.9000351060893

"CD177",-3.000521654975,0.11541933602,-7.68652585522227,4.26561922466101e-10,2.85601329002793e-08,12.8935291304374

"LRRC25",-0.881023258075,0.05996573454,-7.67657530903814,4.42271782385327e-10,2.95155188192527e-08,12.8581891399989

"CRISP3",-2.37149079515,0.37223041288,-7.67189681618102,4.49857559668715e-10,2.99242911769242e-08,12.8415716573931

"SPTLC3",0.949956715708333,-0.0040371578,7.65734053168242,4.74304645001105e-10,3.14483934200085e-08,12.7898632205793

"RNF126P1",-0.785142894425,0.06945018646,-7.65589603657948,4.76802215488132e-10,3.15120122287769e-08,12.7847314233165

"KBTBD3",1.22376222585,-0.09086404172,7.64408350851939,4.97727942101876e-10,3.27892285459268e-08,12.7427621854251

"HMGA2-AS1",2.328653310775,-0.19285930138,7.64098188208902,5.03373569357473e-10,3.30548643878074e-08,12.7317412973149

"ZNF443",0.68177951675,-0.0573649796,7.63475268576364,5.14906904130592e-10,3.35986890033223e-08,12.7096061095553

"RCOR2",2.02373524755,-0.10673853296,7.63473768090533,5.14935003272315e-10,3.35986890033223e-08,12.7095527884504

"HTR2B",2.109783984,-0.1382206968,7.63255513964496,5.19038612929144e-10,3.37589304815629e-08,12.7017968325711

"PROK2",-3.571413447275,0.35762022218,-7.63125291712082,5.21502661727986e-10,3.3811856118617e-08,12.6971691136008

"ASH2L",-0.738500713175,0.00647354046,-7.62921969250216,5.25373393102704e-10,3.39553630217293e-08,12.6899434849523

"TAS1R1",1.3425243386,-0.12323657012,7.60511673559334,5.73515893801792e-10,3.69502944409155e-08,12.604273956038

"ARMC8",-1.02565118945,0.05740025544,-7.60014042709765,5.83993868451882e-10,3.74189703313211e-08,12.5865836460666

"HOXA11-AS",1.084539867325,-0.05115034114,7.59992907457339,5.84443113335746e-10,3.74189703313211e-08,12.5858322857321

"PLAC9",1.0216251442625,0.01744096741,7.59782842573825,5.8892708315272e-10,3.75885921483892e-08,12.5783643613351

"PRTN3",-5.48444949195,1.11115833144,-7.59110694204696,6.03507726724976e-10,3.83995847985755e-08,12.5544679332091

"BTBD8",1.41569916309167,-0.135394604026667,7.5886104359808,6.09015058499e-10,3.86300325651006e-08,12.545591823

"EPB41L2",0.7648256273,0.02381252284,7.58008543817171,6.28204188100869e-10,3.97242203883043e-08,12.515280097093

"AC108488.1",0.875326013175,-0.06778867846,7.57661968843956,6.36177680000424e-10,4.01046409472267e-08,12.5029563714901

"SPON2",1.743922786,-0.1844179552,7.57209847079866,6.46732243461034e-10,4.06449392761646e-08,12.4868788531831

"CCND3",-0.849072789525,0.01312601138,-7.56921439935146,6.53556549032695e-10,4.08447964255585e-08,12.4766226426769

"MYO6",0.96168417815,-0.00115341248,7.56907025496938,6.53899513255721e-10,4.08447964255585e-08,12.4761100343188

"NFIL3",-2.2716743768,0.22044527056,-7.56772552613684,6.5710775124198e-10,4.09204364967954e-08,12.4713278519056

"MPO",-6.333891109,1.2997216328,-7.55749096031562,6.82048303306721e-10,4.23298901565826e-08,12.4349290089247

"HOXC6",1.0077170601,-0.14358154352,7.55675691115035,6.83873176582821e-10,4.23298901565826e-08,12.4323182398332

"PRPS1L1",-1.030198650825,-0.00822835766,-7.55441282564798,6.8973351459965e-10,4.25640368889085e-08,12.4239809716424

"H2BC10",-1.401102746625,0.1480910697,-7.55147138061473,6.97158598055399e-10,4.2893049119997e-08,12.4135187706865

"NFE2",-4.589259749,0.7644290658,-7.52818560006601,7.58840205197209e-10,4.64484500867979e-08,12.3306841806437

"H2BC3",-1.443661037425,0.24172376506,-7.52795406911545,7.59480221548092e-10,4.64484500867979e-08,12.3298604565685

"BBC3",0.82463393495,-0.04866058404,7.5182545112732,7.86784582685985e-10,4.79751265775907e-08,12.2953503661583

"SNX30",0.606845789675,0.02740070414,7.50069956576864,8.38729776230212e-10,5.09907882949691e-08,12.2328832309754

"H3C8",-1.138866409125,0.0323585707,-7.49812312483096,8.46637644325722e-10,5.13192664406668e-08,12.2237143921155

"GYG1",-0.898155631025,0.02050772318,-7.49643618752353,8.51855819678905e-10,5.14832508365233e-08,12.2177109285496

"EPHX2",0.96667968895,-0.06929864884,7.49326443312119,8.61754439696605e-10,5.19283087073648e-08,12.2064230466626

"CPEB1",1.414421811275,-0.16308735698,7.49197433565914,8.65813584288741e-10,5.20199082548614e-08,12.201831652837

"TTC30B",0.749814907475,-0.05576488582,7.48862590057612,8.76438740060994e-10,5.24325965779163e-08,12.1899144770985

"TMEM232",1.2136790006,-0.19090491152,7.4881996374211,8.77800694368669e-10,5.24325965779163e-08,12.1883973676638

"TDP1",-0.95239361635,-0.00570743808,-7.48620984713116,8.84186406372434e-10,5.26604973655768e-08,12.1813154429973

"GABRB1",1.067365510475,-0.10215167532,7.48157970342304,8.9922653614781e-10,5.33261511946851e-08,12.1648356488763

"DST",0.83166006855,-0.07713235141,7.48117034334841,9.00568543213639e-10,5.33261511946851e-08,12.1633786041407

"MMP8",-1.863335992225,0.03181113122,-7.47560808297757,9.19003615993534e-10,5.4105017484125e-08,12.1435801812977

"SIGLEC7",-0.9392279399,0.01068730308,-7.47251543924404,9.29416801592691e-10,5.45612934986563e-08,12.1325717283779

"ZNF582",0.939194990375,-0.0067323977,7.47137076974575,9.33300925363452e-10,5.4632769596704e-08,12.1284971297612

"PPBP",-3.93038759755,0.40907666296,-7.46978857054592,9.38696497416923e-10,5.47920622195952e-08,12.1228650185502

"SDE2",-0.6438078171,0.04317732932,-7.45158181933423,1.0030817617848e-09,5.83839179984287e-08,12.0580492418198

"SNHG1",-0.994333005625,0.0786448663,-7.44967129375412,1.01008964753521e-09,5.86252597696923e-08,12.0512471981841

"AZU1",-5.705557564575,1.13157846834,-7.44573033124591,1.02470070284166e-09,5.93052768356493e-08,12.0372158366369

"RUVBL2",-0.881569529475,0.08671844542,-7.44353918870269,1.03291579176069e-09,5.96123344833607e-08,12.0294143106366

"PLAC8",-2.7713664547625,0.14336332129,-7.44071831067904,1.04358919918306e-09,6.00591446990519e-08,12.0193704076273

"EFEMP2",0.724699500475,-0.01302909862,7.43988851615345,1.0467499040627e-09,6.00723026174696e-08,12.0164158293841

"POLH",0.8276380392,-0.03792828464,7.43702991756502,1.05771202727955e-09,6.05318547902333e-08,12.0062373038395

"TIMP2",0.771596915325,-0.04743995774,7.43437491783944,1.06799645580449e-09,6.09501709930989e-08,11.9967835074336

"NOTCH3",1.0608572986,0.02734231888,7.43255398753792,1.07510792476042e-09,6.11855865624763e-08,11.9902995042573

"YBX3",-1.443860380925,0.07982181926,-7.42867827230571,1.09040260761173e-09,6.18841236142637e-08,11.9764984569955

"LAMB3",1.280259314675,-0.04997383526,7.42661037161534,1.09865218115849e-09,6.21800715126387e-08,11.9691346815751

"KANK4",1.553466316075,-0.03246865164,7.42182664941424,1.11797669569177e-09,6.3099467056014e-08,11.9520994117476

"RAB27A",-1.115461491675,0.08348356866,-7.42037822278108,1.1238947506815e-09,6.32592188240731e-08,11.9469413016845

"RBM4B",0.8672307153,-0.17966723476,7.4184778681043,1.13170697374363e-09,6.34200839654979e-08,11.9401737019856

"PDE4DIP",0.6702495651,-0.08435633117,7.41817823321371,1.13294371004355e-09,6.34200839654979e-08,11.9391066237947

"SCN4B",1.683727182825,-0.13370100874,7.41128373115404,1.16177787883459e-09,6.48569623475833e-08,11.9145527899625

"FASLG",1.68078443445,-0.02472182244,7.40168921526546,1.20313232838472e-09,6.69830846302882e-08,11.8803809891359

"DEFA8P",-3.82010845955,0.73868728236,-7.3942094658024,1.23639263519722e-09,6.85055736378053e-08,11.853739363816

"MKRN1",-0.89268132605,0.09261453106,-7.39283847994444,1.24258822296422e-09,6.85055736378053e-08,11.8488559740559

"HSPD1P6",-1.1253499506,0.18804439482,-7.39201266002922,1.24633517277248e-09,6.85055736378053e-08,11.8459144161786

"ELANE",-5.387109151375,1.1621180869,-7.39187811224604,1.24694672142236e-09,6.85055736378053e-08,11.8454351572884

"RGS9",1.620284811175,0.07586317794,7.39150065564852,1.24866394885919e-09,6.85055736378053e-08,11.8440906551542

"PRR13P1",-0.853200046025,-0.00620228782,-7.39108865790309,1.25054102599274e-09,6.85055736378053e-08,11.8426231128972

"SLC24A1",0.7039643503125,-0.063875228,7.38913744481223,1.2594693142536e-09,6.88106861611406e-08,11.8356727999151

"CXCL14",2.15313964425,-0.1027536008,7.37381872160565,1.33182070333042e-09,7.25700600261532e-08,11.7811032693451

"MXRA5",1.70144881995,-0.05533245354,7.36616533385539,1.36951417755434e-09,7.44260118560566e-08,11.7538374949811

"SP7",1.2005533432625,0.02463954786,7.36186050917307,1.391183664041e-09,7.54036267430476e-08,11.7385005753932

"CLPB",-0.967339353975,0.14412415482,-7.34980160149123,1.4537324501567e-09,7.8464328437142e-08,11.6955355072669

"FGFR3",2.0193865926125,-0.25541061141,7.34950373696365,1.45531261255925e-09,7.8464328437142e-08,11.6944741910009

"MYOM3",1.7408432346,-0.09140057632,7.34120671676967,1.50002659285983e-09,8.06628473346779e-08,11.664910353208

"CCDC80",1.01088848975,-0.0552491127,7.33130591189975,1.55518889160602e-09,8.34102356314767e-08,11.629629794545

"SLC9A3R2",1.161048018425,0.06349874274,7.33027459625562,1.56105050290247e-09,8.35060122805897e-08,11.62595466995

"SLC36A4",-1.093148336825,0.05064950804,-7.32544205509355,1.58881313704014e-09,8.4710072819259e-08,11.6087334379377

"MPP1",-1.09277806625,0.0547832809,-7.32492232034628,1.59182829145913e-09,8.4710072819259e-08,11.6068812807474

"PGAP2",0.600802195925,-0.04360054926,7.31942641121285,1.62406490802216e-09,8.62016627864197e-08,11.5872953642346

"MYOM2",1.3449071396,-0.11285945932,7.31252560093112,1.66547003464161e-09,8.81709304127577e-08,11.5627018120765

"IFIT1",2.065057705675,-0.00440205646,7.3115072287341,1.67166922874665e-09,8.82710287591787e-08,11.5590723830746

"INSC",2.017010879125,0.0792373763,7.31052204685256,1.67768838276056e-09,8.83611300411266e-08,11.5555612212605

"GPR160",-1.639350909375,0.1833629535,-7.30904741022485,1.68673855565761e-09,8.84885437925855e-08,11.5503056165146

"LRTOMT",0.658327833575,-0.09840662914,7.30863214566403,1.68929594230833e-09,8.84885437925855e-08,11.5488256051888

"MAPK14",-1.015715548875,0.0398898499,-7.30768387092257,1.69515042092090e-09,8.84885437925855e-08,11.5454459208174

"PF4V1",-4.084515337575,0.55494342494,-7.30732299792502,1.69738372269065e-09,8.84885437925855e-08,11.544159751738

"COL13A1",1.8717922693,-0.22080921156,7.30430897411433,1.71615184320255e-09,8.92398958465326e-08,11.5334175139318

"LINC01197",1.11115941785,-0.22490952672,7.30277913572796,1.72575745115224e-09,8.95121991372332e-08,11.5279649654108

"APEX1",-0.8937958643,0.05547351056,-7.30021746672571,1.74196252874831e-09,9.00915020276677e-08,11.5188347237547

"SLCO1C1",1.9252864729,0.02443942832,7.29962678834749,1.74572072945061e-09,9.00915020276677e-08,11.5167294218826

"HOXC8",1.142293189475,-0.06043494542,7.2914867591438,1.79834683004289e-09,9.2574195612861e-08,11.4877159080244

"HSF2BP",1.300117174825,0.00107849986000001,7.29001488741062,1.80803093832446e-09,9.28394432691516e-08,11.4824695686831

"FAM167A",1.2558741841,-0.06848197117,7.28915652934915,1.81370256414766e-09,9.28978453356429e-08,11.479410017025

"AL512599.1",-1.070325925775,0.00819839638,-7.28041975131954,1.87245493809657e-09,9.5667971999308e-08,11.448267598025

"RASSF9",2.3732995485,-0.2729314842,7.27933860573875,1.87985667741132e-09,9.58072229024952e-08,11.4444137277761

"FCER1G",-0.8922836468,0.07795301406,-7.27702071630242,1.89582434879361e-09,9.6381263667701e-08,11.4361512618748

"LINC00476",0.818148436925,0.02615650309,7.27268690488837,1.92604490463575e-09,9.76752673420226e-08,11.4207024589626

"TRIL",1.429067431,-0.1181727172,7.2705360670036,1.94122185234515e-09,9.82018600267837e-08,11.4130352008349

"HEY2",1.0546308842625,-0.01670731449,7.2657393918115,1.97550153070722e-09,9.93764271322316e-08,11.3959358062406

"SLC25A37",-2.0012543164125,0.21998447627,-7.26565233680778,1.97612924428897e-09,9.93764271322316e-08,11.3956254648162

"RGS7BP",1.7997578055,0.0387086474,7.2646992367692,1.98301469250398e-09,9.93764271322316e-08,11.3922277597368

"ZNF396",0.8725105821375,-0.02606992784,7.26458491644198,1.98384218552727e-09,9.93764271322316e-08,11.3918202182157

"PSIP1",0.8814308715,-0.0474250699,7.25814617312371,2.03101091464257e-09,1.01491101510237e-07,11.3688662973076

"CLEC4D",-2.2167578762875,0.24592995247,-7.25229630393965,2.07483903690825e-09,1.03428959095319e-07,11.348011041403

"DEFA4",-6.19573553865,0.99350181908,-7.2510213583894,2.08451616717007e-09,1.0365914376937e-07,11.3434656764705

"AADAT",1.1273327813,-0.08441950596,7.2445618418611,2.134244603395e-09,1.05875068848322e-07,11.3204361291852

"PLIN2",-2.6027622515125,0.35997723329,-7.24297783833971,2.14661936858349e-09,1.0623173339019e-07,11.3147887100301

"CLSTN1",0.627769422125,-0.0485565553,7.23661646700571,2.1970446579592e-09,1.08465183017514e-07,11.2921081979138

"ZFHX3",0.7725039590125,-0.06707843839,7.23579056703492,2.20367783070402e-09,1.08531133162173e-07,11.2891635259911

"CMTM7",-0.59187933775,0.00102603445,-7.22395459382486,2.30097128582208e-09,1.13051078426674e-07,11.2469622037515

"TRIM45",1.352556850875,-0.1970536163,7.22270117052609,2.31152330199886e-09,1.1329782155826e-07,11.2424929744453

"PF4",-4.200166171075,0.63338974014,-7.22080407415493,2.32758637919193e-09,1.13541880325915e-07,11.2357286056107

"SMAD6",0.779844465225,0.03924746918,7.21814912709935,2.35025420659325e-09,1.14137348321299e-07,11.2262619175685

"SERTAD4",1.1471503533,-0.18901161236,7.21806976028872,2.35093522996819e-09,1.14137348321299e-07,11.225978919349

"PLCD3",0.89807482905,-0.06333482776,7.21464744272039,2.38048990626294e-09,1.1504523168196e-07,11.2137758705792

"RUNX2",1.02201823675,-0.0132744451,7.21460419182748,2.38086578646774e-09,1.1504523168196e-07,11.2136216485786

"TSC22D1-AS1",1.733404928225,-0.19443293642,7.21311670849954,2.39382928633102e-09,1.15399469219647e-07,11.2083176332506

"LAMB2",0.9084992851625,0.02757214563,7.20372407284601,2.47733499848624e-09,1.19144693542221e-07,11.1748249465759

"H2BC8",-1.556443778025,0.24207943058,-7.19696747418761,2.53920319934371e-09,1.21834180674833e-07,11.1507311719261

"RCBTB2",0.99432765175,-0.1380385626,7.19537802332156,2.55398073549444e-09,1.22256909600024e-07,11.1450631572992

"NPHP1",0.756259155325,-0.08271616874,7.19147891697704,2.59059754359014e-09,1.23177524534371e-07,11.1311587163933

"NDUFB9",-0.84677990575,0.0363986294,-7.19145487712336,2.59082492668286e-09,1.23177524534371e-07,11.1310729882291

"PSMF1",-0.66075032898125,0.028065373865,-7.19141002965473,2.5912491738732e-09,1.23177524534371e-07,11.130913058318

"MDGA1",1.04151203085,-0.08947611152,7.18691598857459,2.63411650373928e-09,1.24670147583695e-07,11.1148867863678

"ZNF573",0.83566245875,-0.013918133,7.18684294181035,2.63481910892913e-09,1.24670147583695e-07,11.1146262909102

"PCDHB11",0.8247938171,-0.14718761532,7.18418213785193,2.66054055579442e-09,1.25597131122387e-07,11.1051374265685

"AKAP17BP",2.21156954785,-0.28851338612,7.18035290821543,2.69799887405308e-09,1.27072645819769e-07,11.09148160279

"JMJD1C-AS1",1.139897176075,0.000468737359999997,7.1745578048738,2.75569458111394e-09,1.29492363710694e-07,11.0708147132275

"AC004540.1",1.900788245725,-0.05986726342,7.17345550732425,2.76680804032759e-09,1.29717078101217e-07,11.066883577828

"COL8A1",1.71518815425,-0.2145635626,7.17198917651455,2.78166130330311e-09,1.30115700415694e-07,11.0616541637008

"HMGA1",-1.4859499325,0.112121631,-7.16980357218829,2.80394880578356e-09,1.30859460439393e-07,11.0538595370867

"SLC11A1",-1.78394092146667,0.227626873826667,-7.16791900881951,2.82330997891928e-09,1.31463579200223e-07,11.0471384813876

"PDK1",-1.264268256375,0.0498325559,-7.16594769600443,2.84370563955703e-09,1.32113018465407e-07,11.0401079997364

"TCEA3",1.08260384615,-0.11003167108,7.16118342964535,2.89360905540479e-09,1.34127290332881e-07,11.0231165533956

"MIR100HG",1.200257139825,-0.12355684314,7.15679524397124,2.94034861752575e-09,1.35986145543719e-07,11.0074661455979

"IHH",2.1116284462,0.03413034996,7.15353870705843,2.97552270046848e-09,1.36994402443142e-07,10.995851610422

"POLE3",-0.6137379418,-0.02480487844,-7.15157364393723,2.99695097153425e-09,1.37671595302228e-07,10.9888430989446

"CSNK1D",-0.6341002707,0.05039867394,-7.1469159372409,3.04836065754229e-09,1.39719939936748e-07,10.9722309541464

"CABCOCO1",1.82579148415,0.07037543132,7.14557863853012,3.06328364957149e-09,1.40018892236652e-07,10.9674613118637

"METTL25",0.8259830756,-0.01781194752,7.14510812835695,3.06855147472943e-09,1.40018892236652e-07,10.965783174399

"GLRX5",-1.2112616939,0.09100602988,-7.14382939276096,3.08291404505912e-09,1.40031057789015e-07,10.9612223814374

"TUBG1",-0.9244517461375,0.17140705659,-7.14374544158439,3.08385932328103e-09,1.40031057789015e-07,10.9609229570057

"AJUBA",1.10619043935,-0.11331119552,7.14292363415406,3.0931280948205e-09,1.40031057789015e-07,10.9579918540835

"BIVM",0.7937757821,-0.02665730032,7.14219516237384,3.10136749481783e-09,1.40031057789015e-07,10.9553936414583

"S100A13",0.9197650411,0.02827066588,7.14189593984634,3.10475822261632e-09,1.40031057789015e-07,10.9543264142935

"C2orf88",-1.61918764275,0.2538080308,-7.1414492619422,3.10982679099971e-09,1.40031057789015e-07,10.9527332612121

"IDH2",-1.292022571125,-0.0017939459,-7.13898436283816,3.13794598856914e-09,1.40987362749571e-07,10.943941741705

"TMCO1-AS1",1.09997456085,-0.08333884382,7.13820595925912,3.14687867621733e-09,1.41079322359608e-07,10.9411654086545

"TTC21A",1.02430781005,-0.04691258996,7.13742649888779,3.15584900079799e-09,1.41172564035697e-07,10.9383853004292

"ZNF846",1.629034861775,0.00831869942,7.1335625342403,3.2006963885914e-09,1.42866813963966e-07,10.9246035772313

"PIGM",0.6580435981375,-0.03734919534,7.13015498817137,3.24077547360553e-09,1.44341321528761e-07,10.9124496597752

"ZNF518A",0.84329771035,-0.08879073572,7.1250081189389,3.30226683786849e-09,1.46761047666485e-07,10.8940917839471

"H2BC14",-1.3522043456,0.12354410152,-7.12348685918031,3.32066448660564e-09,1.47084487210959e-07,10.8886657029425

"ANTXR1",1.0938826977,0.04966553766,7.12321997592914,3.32390265417192e-09,1.47084487210959e-07,10.8877137726153

"YEATS4",-0.9046370361,0.11834387612,-7.11037050597253,3.48360675606817e-09,1.53819256936044e-07,10.8418810426867

"IFT81",0.7436233815875,-0.03771290298,7.09820145371781,3.64193020988349e-09,1.601198844208e-07,10.7984741142156

"COL8A2",1.4020202186375,-0.06680818959,7.09757190448376,3.65031404926518e-09,1.60144827069261e-07,10.7962284876167

"ARMH4",1.1734389110625,-0.05771860665,7.09537533596842,3.67971779366681e-09,1.6108986785608e-07,10.788393221353

"ACOX2",1.086377910675,-0.06738334546,7.09178168141592,3.72833531630113e-09,1.62870221663918e-07,10.77557441294

"COL15A1",1.6336086543,0.12954462844,7.08959184781178,3.75827567099908e-09,1.638288339307e-07,10.7677630881095

"ARPC5L",-0.808925843,0.0715368468,-7.0884734502939,3.77365960582441e-09,1.64134507207285e-07,10.7637736564363

"LINC01004",1.326746069575,-0.12358593134,7.0879189430053,3.7813103964193e-09,1.64134507207285e-07,10.7617956726806

"PCDHB17P",1.51440570525,-0.1296800688,7.08180222736187,3.86674273552338e-09,1.67488002463854e-07,10.7399766061313

"ZKSCAN3",0.620880349625,-0.0161872103,7.07580416984722,3.95239356451367e-09,1.70836791877123e-07,10.7185806028429

"NAMPT",-1.660918352625,0.1909843429,-7.06769520917939,4.0712142421234e-09,1.75602183984472e-07,10.6896543861983

"TRIM65",0.7382688018,-0.05663713416,7.06607901841916,4.09532019443288e-09,1.76270840637691e-07,10.6838890867243

"H2AC19",-1.2278989535,0.0059979472,-7.0621546470119,4.15444954231594e-09,1.78012897830071e-07,10.6698899633067

"FRMD6-AS1",2.05956927925,-0.0671937198,7.06173360189886,4.16084405434602e-09,1.78012897830071e-07,10.6683879959347

"FKTN",0.8195007533875,0.03782289946,7.0616667977054,4.16185953048634e-09,1.78012897830071e-07,10.6681496895293

"PER3",1.2002485264125,-0.11501993222,7.05554723322337,4.25594149639474e-09,1.81657769537782e-07,10.646319675794

"IRGM",-1.854794458675,-0.06231237704,-7.05497652920786,4.26482324770792e-09,1.81658417253721e-07,10.644283824844

"BOC",1.102845917875,-0.0712119947,7.05276191910342,4.29946477687457e-09,1.82754013171382e-07,10.6363837184785

"PIM1",-1.306449804775,0.15467591718,-7.05178600691484,4.31481950748795e-09,1.83026960806238e-07,10.6329023733083

"THSD4",1.6459153810125,-0.11945864219,7.04637258294079,4.40099468347991e-09,1.86296650981687e-07,10.6135911553149

"CEACAM3",-3.192505506425,0.22275092486,-7.04303082246824,4.45504905629392e-09,1.88195969206907e-07,10.6016701036811

"CABP1",1.1279554469,-0.06088180548,7.04187800587842,4.47385006137576e-09,1.88601316990672e-07,10.5975576574138

"GLT1D1",-2.681257966475,0.43966411482,-7.0400385497106,4.50401385689163e-09,1.89483030595474e-07,10.5909957523218

"ZNF280D",0.760729321425,0.00256882314,7.03060210195917,4.66198445230999e-09,1.95726921022392e-07,10.5573329207046

"NPNT",1.1943857232,-0.07024692544,7.02887371299704,4.69151357758216e-09,1.96563865393667e-07,10.5511671842684

"SLC51A",-2.05284187625,0.266360979,-7.02479554601751,4.76193200808843e-09,1.99107067309624e-07,10.5366189913402

"TRAF5",0.821184806425,-0.01840748786,7.02194866382246,4.81171551280024e-09,2.00778874595216e-07,10.5264631913513

"ZNF248",0.7552552820125,-0.06273156794,7.02001842583368,4.84576554551865e-09,2.01788708326394e-07,10.519577368473

"MLIP",1.177548992175,-0.02550268126,7.01944370866864,4.85595024642411e-09,2.01802654459913e-07,10.5175271538926

"SMIM3",-1.93503416325,0.0702160804,-7.01768860474489,4.88718563070551e-09,2.02689593526102e-07,10.5112660902146

"AC021092.2",0.75351225425,0.0064062484,7.01649937325878,4.90846442421512e-09,2.03160846713777e-07,10.507023688042

"TOP1",-0.6641497801125,-0.00267950984,-7.012487495861,4.98093471676074e-09,2.05744738864907e-07,10.4927119194272

"CLEC3B",1.04368939185,-0.06638563052,7.01162310184621,4.99668871380836e-09,2.05980197924559e-07,10.489628322801

"GSC",0.877115702675,-0.07436468186,7.00853755471156,5.05333199532551e-09,2.07896919518532e-07,10.4786210919538

"KIF3A",0.7009424956375,-0.07711421489,7.00780314015426,5.06690847692997e-09,2.08037717185053e-07,10.4760011768707

"NTN5",1.13142710715,0.02850861472,7.00709162013731,5.08009653066807e-09,2.08162035440655e-07,10.473462934618

"LILRA6",-1.1873158844125,-0.03807340128,-6.99750441080707,5.2611812769143e-09,2.15151860282276e-07,10.4392619947345

"KRT18P59",1.197873447125,0.0181051427,6.99582952186758,5.29347310258294e-09,2.16041189095058e-07,10.433287082766

"UQCRFS1P2",-0.780003424575,0.02459821734,-6.99438278264113,5.32152577218592e-09,2.16591430876874e-07,10.4281260620877

"CCDC191",1.17565446995,-0.13986872604,6.99404491788424,5.32809845577628e-09,2.16591430876874e-07,10.4269207815695

"GPRC5C",1.1170575887125,-0.06437908803,6.99263322462124,5.35564893654305e-09,2.17280268142364e-07,10.4218847843439

"CALHM2",0.897775979625,0.0218712627,6.98763174257046,5.45440936158672e-09,2.20849681818555e-07,10.4040427897034

"ABCB4",1.81301949855,-0.30549980016,6.98091686177444,5.58987400620185e-09,2.25888241891644e-07,10.3800885750825

"PCDHA3",1.2448610864,-0.26303905388,6.97966631538809,5.61547156282529e-09,2.26475947596781e-07,10.3756274689805

"ZNF618",1.125736562625,-0.1383960791,6.97831708469077,5.64322061855678e-09,2.27147945054993e-07,10.3708143274209

"ZNF334",1.039910312225,-0.12728151922,6.97765610457894,5.65686473395012e-09,2.27250675821902e-07,10.368456399356

"ABCG4",1.472674177125,-0.1204441773,6.97629668066517,5.68503011626045e-09,2.27562601131063e-07,10.3636069012862

"H2BC13",-1.295832023925,0.25725700386,-6.97620938223558,5.68684360499338e-09,2.27562601131063e-07,10.3632954801279

"CEACAM8",-4.983390252925,0.88523902766,-6.97153376288065,5.78482254067768e-09,2.31032054996889e-07,10.3466160915967

"UQCRFS1",-0.847567558825,0.01344871394,-6.96647609959939,5.89270941803557e-09,2.3488293882629e-07,10.3285738997792

"CST7",-3.444257055975,0.28192508992,-6.95978229985635,6.03859813761192e-09,2.40230676977462e-07,10.3046952441792

"IFFO2",0.86215246,-0.008635617,6.95449273436871,6.15643315479291e-09,2.44443803246894e-07,10.2858259974603

"RHOG",-0.8238511201,-0.01184798308,-6.95283776405687,6.19377099822824e-09,2.44877215982815e-07,10.2799223132166

"MMP16",1.836116648,-0.0102470096,6.95256725660996,6.19989542614014e-09,2.44877215982815e-07,10.2789573479981

"AL049775.1",0.960652885575,-0.08875670824,6.95242116475386,6.20320553958809e-09,2.44877215982815e-07,10.2784362032853

"CDC14C",0.6188418460125,-0.01811530969,6.95089614294731,6.23786482082897e-09,2.45595445213106e-07,10.2729960898424

"IL17RD",1.4779202794,-0.13328085948,6.9505668438014,6.24537421690882e-09,2.45595445213106e-07,10.2718214032083

"IMP4",-0.9130839643,0.10768857956,-6.94448799880783,6.38563275974722e-09,2.50581495526086e-07,10.2501368361777

"HEG1",1.049261708075,-0.03566539854,6.94366978544179,6.40475060306025e-09,2.50581495526086e-07,10.2472181035893

"SAMSN1",-1.1144391871875,0.02109508445,-6.94349427049897,6.40885902263126e-09,2.50581495526086e-07,10.246592006705

"ZNF462",0.761016413975,-0.11017099382,6.93695176723214,6.56389968942553e-09,2.56154622546572e-07,10.2232537001175

"ALAS1",-0.9606560377,0.07697362884,-6.93256735245779,6.6698928432753e-09,2.59193779580496e-07,10.2076138173465

"SEMA3D",2.189770471175,-0.29843777806,6.93219065703029,6.67907891132792e-09,2.59193779580496e-07,10.2062700917648

"LINC01252",1.316419138925,0.09374824614,6.93216396258202,6.67973036013775e-09,2.59193779580496e-07,10.2061748689434

"ZNF594",0.9594778419875,-0.07357783291,6.93153260438105,6.69515650480393e-09,2.59301259868474e-07,10.2039227273492

"NKX3-2",1.53009242635,-0.16641043692,6.93011517948214,6.72991873420922e-09,2.60155801936752e-07,10.1988665860967

"HAAO",0.90299402645,0.00470535591,6.92947908124201,6.74557761525329e-09,2.60270045539189e-07,10.1965975437571

"KCNS3",1.480258168625,0.0536933364,6.92814620773406,6.77850733674201e-09,2.61048981795433e-07,10.1918430243292

"DPPA4",-2.551178039725,0.10563966522,-6.9248035382082,6.86179958013661e-09,2.63760881421837e-07,10.1799193695409

"CPS1",0.89850789715,-0.03449578428,6.92060184663637,6.9679502406826e-09,2.6733963395338e-07,10.1649315929927

"CNIH3",1.104269289,-0.00145786180000001,6.91955259486188,6.99471358542586e-09,2.67392136058024e-07,10.1611888444169

"H2BC12",-1.447446472625,0.1665744685,-6.91952491629844,6.9954209745754e-09,2.67392136058024e-07,10.1610901133005

"SLC4A3",1.287269242325,-0.04530009944,6.91759833536883,7.04483537223395e-09,2.68779491818118e-07,10.1542178949147

"MXD1",-1.374590815525,0.12255702108,-6.9167360870248,7.06706389179835e-09,2.69126403373912e-07,10.1511422166759

"VMA21",-0.8119114392625,-9.99571000000338e-06,-6.91169735212299,7.19837073794715e-09,2.73111517924187e-07,10.1331689299015

"TBXA2R",0.751626467025,0.04240720762,6.90573351193067,7.35694159813301e-09,2.78611865919684e-07,10.1118960125867

"P2RY2",-1.801601291025,0.18448561418,-6.90345994664401,7.41830827279303e-09,2.8041752747783e-07,10.103786315912

"SPAG16",0.7842660604375,-0.0028813843,6.90248774363418,7.4447053643008e-09,2.80560300170782e-07,10.1003185276845

"CYP39A1",1.114729339825,-0.20655881114,6.90231251867604,7.44947302288684e-09,2.80560300170782e-07,10.0996935118665

"TRPV1",0.9247128506375,-0.01352874674,6.90095446529038,7.48652768445435e-09,2.81438493943304e-07,10.0948494338613

"REM1",1.11192263085,-0.10309604532,6.89376949949285,7.68565920642419e-09,2.88395212126774e-07,10.0692214379822

"SNAI3-AS1",1.017895914075,-0.00346598329,6.8888138550549,7.82608275738665e-09,2.93127575015243e-07,10.0515454523534

"ZNF112",0.7666410306,-0.02793058352,6.88501917417197,7.93534093755548e-09,2.96677491110651e-07,10.0380105884212

"SERPINB10",-2.89718954065,0.39406932248,-6.88408034702295,7.9626065930537e-09,2.97154615443505e-07,10.0346620015665

"GYPA",-4.207820810025,1.05887639398,-6.8834794874284,7.98010604809645e-09,2.97266204933455e-07,10.0325188739403

"HECTD2",0.661872723883333,-0.01819215536,6.87991083330614,8.08483490802632e-09,3.00620866779752e-07,10.0197903795017

"TAS2R14",1.085053591275,-0.06972090598,6.86448875789668,8.55345683867305e-09,3.17469608171618e-07,9.96478519620374

"ARHGEF10",1.215873523075,-0.09324480454,6.86177486226671,8.63868873636822e-09,3.2005326370834e-07,9.95510593880411

"COL14A1",1.2701391957,-0.07414796394,6.84492449925816,9.18722029381261e-09,3.39149134017356e-07,9.89501012701593

"SDK2",1.12797750125,-0.03220962335,6.83580412731752,9.49850138901505e-09,3.49827797290824e-07,9.86248434486662

"HCCS",-0.73836642225,0.0339090802,-6.83513707116065,9.52167769160406e-09,3.49827797290824e-07,9.86010548025881

"H2BC9",-1.427231144475,0.20515207542,-6.83496345234283,9.52771919603083e-09,3.49827797290824e-07,9.85948631948711

"CDH11",0.9054885974,-0.03053110873,6.83264605479436,9.60872674185532e-09,3.5217100802707e-07,9.85122203756466

"CTSF",0.70318236815,-0.03260361528,6.8303778352318,9.68868185761715e-09,3.53944509988737e-07,9.84313320493584

"FLRT2",1.09456701765,-0.11754025378,6.8302935181533,9.69166683442411e-09,3.53944509988737e-07,9.84283251816013

"TTC3",0.67572205615,-0.06818328458,6.8259959629512,9.84503172816926e-09,3.58905711826925e-07,9.82750695274725

"CXCR1",-1.505810767375,0.1732821741,-6.82233491366665,9.97759387896203e-09,3.63092261797823e-07,9.81445144621457

"HMGB3P1",-1.698826649,-0.0249866522,-6.82112348091063,1.0021850056261e-08,3.64056141760061e-07,9.81013145194898

"PTX3",-2.9726677595,0.2125018724,-6.81894753074307,1.01018353610843e-08,3.66312217483002e-07,9.80237202267872

"SNHG3",-0.926195744075,0.11013965874,-6.81472330128721,1.0258939122893e-08,3.7101270891307e-07,9.78730863897518

"C2orf81",0.70327490415,-0.03913274168,6.81449020625554,1.02676789317508e-08,3.7101270891307e-07,9.78647744197023

"KNSTRN",-0.9082089029,0.17145294068,-6.81357002771807,1.03022534075563e-08,3.71606633475375e-07,9.78319617184135

"SS18L2",-0.718064656025,-0.02842559882,-6.81225862200197,1.03517289008505e-08,3.7273501181129e-07,9.77851984472349

"LIMCH1",0.9518889381375,-0.07959035999,6.8108976984663,1.04033236606239e-08,3.73935605541864e-07,9.77366697050018

"GART",-0.7666271163625,0.11198581821,-6.80256011249884,1.07250731314088e-08,3.84825391096855e-07,9.74393686961885

"R3HDM4",-0.91043470705,0.10927898526,-6.80145222090653,1.07685688805238e-08,3.85710558084216e-07,9.73998644169511

"NUP88",-0.615714862675,0.03709405336,-6.79873322324994,1.08760652467474e-08,3.88881020550369e-07,9.73029135022295

"BFSP1",1.575572420075,0.04093203606,6.79201301472502,1.11463738685774e-08,3.97851755782951e-07,9.70632972389078

"CCNJL",0.99925416225,-0.2005077822,6.79043629663345,1.12107608765591e-08,3.99254617842847e-07,9.70070787642996

"CDKL5",0.6985477218,-0.01799545256,6.78953330942766,1.12478025936121e-08,3.99384713237303e-07,9.6974882608941

"SLC36A2",2.542037971425,-0.15059459486,6.7861971994937,1.13857179850297e-08,4.03582335773856e-07,9.68559342916291

"ZFP14",1.011628314025,-0.05121432118,6.78502282087508,1.14346680905841e-08,4.0461740904989e-07,9.68140625245311

"KCTD12",0.8327276182875,-0.08976644337,6.78291174607399,1.15231906972266e-08,4.06034166897418e-07,9.67387940271411

"H2BC11",-1.6027257867,0.20330716064,-6.78282807720209,1.15267132145607e-08,4.06034166897418e-07,9.67358109040935

"RGL4",-3.930797239025,0.62291502128,-6.78265126347355,1.15341607347861e-08,4.06034166897418e-07,9.67295068061201

"FBXL22",0.8838406977,-0.07253806984,6.78090432509186,1.16080019649005e-08,4.07851549524988e-07,9.66672219498728

"ALYREF",-0.854181556725,-0.03289806238,-6.78048961492872,1.16256005916924e-08,4.07851549524988e-07,9.66524360691272

"EFHC1",1.140736022525,-0.07462202998,6.77928518998717,1.1676862830545e-08,4.08949684909752e-07,9.66094942454205

"MMAA",0.695376270375,-0.0141839127,6.77846373924042,1.17119545771072e-08,4.09478712245344e-07,9.65802068977944

"C14orf28",1.05880924325,-0.1996753004,6.77318543789524,1.19399672939053e-08,4.1673943767893e-07,9.63920215084454

"ZNF662",0.852183087475,-0.04822803402,6.77184036581481,1.19987776724742e-08,4.18054201153126e-07,9.63440669584547

"CAVIN4",1.136620477925,-0.07544688366,6.77139199125548,1.20184461381878e-08,4.18054201153126e-07,9.63280815704065

"UNC5B-AS1",1.283781231325,-0.00288395994000001,6.76890424602864,1.21281606346656e-08,4.2115551708988e-07,9.62393895069756

"TMIGD3",-1.633559153375,0.0813160016,-6.75941488006884,1.25559285071136e-08,4.35272188246606e-07,9.59010894423944

"IL2RB",1.3320410081,-0.05290448052,6.75227738586243,1.28875819038258e-08,4.46014827779701e-07,9.56466463204129

"RGN",1.2398067551,-0.12396684792,6.7457554107689,1.31982813444098e-08,4.55944075519642e-07,9.54141547757424

"FBL",-0.722067861475,0.04870775382,-6.74532606348376,1.32189955514773e-08,4.55944075519642e-07,9.53988499647807

"FANCE",-0.68341484365,0.05064781268,-6.74260098010799,1.33512288209822e-08,4.59731052242493e-07,9.53017106563734

"UACA",1.28511541365,-0.10347343298,6.73641723403861,1.36562143710639e-08,4.69443825561003e-07,9.50812884633246

"RPS6",-0.6970602266375,0.01805345469,-6.73198305774205,1.38791858245028e-08,4.76309479350775e-07,9.49232355051222

"RNF149",-0.790215718175,0.04426014106,-6.72174091291268,1.44082104144995e-08,4.93637817679376e-07,9.45581784822939

"GNAS-AS1",0.934600445275,-0.05261952478,6.71949673727589,1.4526790063843e-08,4.96869574003363e-07,9.44781933327292

"RETN",-3.9404560771625,0.32970430077,-6.71040730545287,1.50171187401981e-08,5.12784547915299e-07,9.41542469397324

"BLID",1.25351721875,-0.205882885,6.70983355442727,1.50486186383405e-08,5.13005155844125e-07,9.41337991695704

"CDA",-1.545503601175,0.19517869506,-6.70235220762605,1.54654491952282e-08,5.25466207482314e-07,9.38671805882209

"SLC25A5",-1.068386662825,-0.01279516326,-6.69992251670055,1.56032873014225e-08,5.29271771906531e-07,9.37805947448878

"SLC2A1",-1.120930454375,0.0401769455,-6.699065644414,1.56521903934747e-08,5.30053019473571e-07,9.37500591069178

"CA1",-4.48293916725,0.7152770662,-6.6941180008766,1.59375692671866e-08,5.37938911278616e-07,9.35737476790172

"RIPOR2",-1.2359441699125,0.06458865657,-6.69073278056382,1.61358164726042e-08,5.43734552451834e-07,9.34531174444526

"ATXN3",0.796303360575,-0.00289294729,6.68933457989498,1.62184156695864e-08,5.45620525843164e-07,9.34032942814179

"PDP1",0.9700835816,-0.13120176572,6.68770592183032,1.63151618041648e-08,5.479754672848e-07,9.33452596862528

"GPR84",-1.2437653806,0.06005326852,-6.68514995102596,1.64681558822462e-08,5.522088014983e-07,9.32541831861141

"HIBCH",0.6796222219,-0.02032233248,6.6836618695808,1.65578878287684e-08,5.54310467051972e-07,9.32011594009793

"LRRC6",0.721217248225,-0.03959115202,6.68253794060365,1.66259847654402e-08,5.55682179240358e-07,9.3161111595402

"RTL5",0.876763439075,-0.02030612814,6.67695875353532,1.69681809109038e-08,5.66195587137781e-07,9.29623191018426

"HTR2A",1.42694089485,0.04185216888,6.67227858859526,1.72606549550417e-08,5.75018371900642e-07,9.27955661675884

"DUBR",1.1148501429,-0.11920539568,6.66879337180588,1.74817196174783e-08,5.81437453770934e-07,9.26713927575173

"IFRD2",-0.78410556015,0.11431510888,-6.66568129462113,1.76815047226167e-08,5.86607092524563e-07,9.25605166122871

"AC015911.1",-0.8924981075,0.062178536,-6.66508767750947,1.77198711222668e-08,5.86607092524563e-07,9.2539367705864

"SNPH",1.026943100775,-0.01219896438,6.66503859433795,1.77230471628614e-08,5.86607092524563e-07,9.25376190150364

"IFT80",0.7082084435,-0.0208452242,6.6634829702579,1.78240024877407e-08,5.88459789985069e-07,9.24821969955561

"LRRC17",2.265884348025,-0.03326371858,6.66329151942027,1.78364666917575e-08,5.88459789985069e-07,9.2475376247822

"DZIP3",0.8408600301875,-0.06045805085,6.66219769618969,1.79078459989188e-08,5.89864869494932e-07,9.24364072115323

"BEX1",-2.065358967775,0.07166130778,-6.65727800733936,1.82324317896573e-08,5.99592395676563e-07,9.2261140341892

"CAVIN3",1.180327438075,-0.06494176954,6.65287263568754,1.85280669055741e-08,6.08338196733017e-07,9.21042021820662

"RASL12",1.359352204175,-0.17574293166,6.65201533705305,1.85861523694303e-08,6.0926894359182e-07,9.2073662191224

"FGFR1",0.977756592183333,0.0127173590133333,6.64792328348263,1.88659207701034e-08,6.17452052296929e-07,9.19278918012689

"SRSF2",-0.65414739755,0.03847249996,-6.64675566332308,1.89465177882833e-08,6.18560388661605e-07,9.18862988040405

"PLCD1",0.657635738675,-0.01213747106,6.64655840056371,1.89601681022788e-08,6.18560388661605e-07,9.18792719442037

"ZNF549",0.600858601225,-0.07041861472,6.64483419751676,1.90798994030534e-08,6.21476914101365e-07,9.18178531703762

"CLIC3",1.4160067793,-0.08262388146,6.64391559370224,1.91439966050146e-08,6.22574924513553e-07,9.17851314435249

"ST20",-0.664165611775,0.02836809158,-6.64238367265023,1.92513676989927e-08,6.25074518885836e-07,9.17305632068747

"TRPS1",0.9806672464,0.01112357262,6.63952583720603,1.9453280762146e-08,6.30631038377923e-07,9.16287667252919

"CMAS",-0.82732548335,0.06995113312,-6.63203552032429,1.99925800423562e-08,6.47090015652122e-07,9.13619723348432

"GJC2",0.9395848203,0.08553573624,6.62654562829606,2.03973058172596e-08,6.59148267482672e-07,9.11664411438612

"SSRP1",-0.59919400355,0.02828296576,-6.62505888566343,2.05083121450086e-08,6.61691809806198e-07,9.11134900645992

"FMNL3",0.8742518725625,0.00874252345,6.62354714327628,2.06218035030227e-08,6.64307405927562e-07,9.10596493167707

"HOXC9",0.92654012385,0.02223461708,6.61936860708904,2.09387719671381e-08,6.73459277963464e-07,9.09108343825469

"HERC6",1.8213038731625,0.16924994953,6.61812736502155,2.1033861692885e-08,6.74499627076836e-07,9.08666296931446

"TUBB4B",-0.969795922325,0.02629815544,-6.61808699992002,2.10369612310669e-08,6.74499627076836e-07,9.08651921681223

"KCTD11",0.62976242655,0.00591355224,6.616490485154,2.11599200906108e-08,6.77381941900678e-07,9.08083358005606

"CAPS",1.0781794308,-0.01100383136,6.6127810047599,2.14483906064263e-08,6.85545439538943e-07,9.06762339663729

"PPIA",-0.6809195303375,-0.03403775877,-6.61212358571951,2.14999233164778e-08,6.86122163408094e-07,9.06528224557703

"CCDC42",1.46580500385,-0.17286180492,6.61168183407231,2.15346200498252e-08,6.86160646315426e-07,9.06370912084103

"KANK2",0.66410579825,0.0104737466,6.60922640985189,2.17284990733174e-08,6.91263181698954e-07,9.05496520915532

"CCDC146",0.875237328675,-0.03610768806,6.60739955244678,2.18738764094825e-08,6.94809271127873e-07,9.048459789261

"ZCWPW2",1.0048096153,-0.10711727146,6.6068122073995,2.1920822008184e-08,6.95222602637265e-07,9.04636828305975

"TUBA4B",-1.262740946825,0.21238624624,-6.60463287821438,2.20958931436195e-08,6.99691899113564e-07,9.03860790081843

"ST6GALNAC4",-0.669591715325,0.05015694434,-6.60397268179057,2.21492033715561e-08,7.00297652278458e-07,9.03625703568872

"PRTG",1.5644310387,0.18156139196,6.59640091878019,2.2769873830374e-08,7.18812288192145e-07,9.00929611077409

"MARCHF3",-0.937173864425,0.07880071121,-6.59509889073964,2.28783393216115e-08,7.20894401303179e-07,9.00466014866697

"PAPLN",0.7851297399125,-0.03473049142,6.59447483144958,2.29305092888016e-08,7.20894401303179e-07,9.00243816223985

"TBCK",0.638332351775,-0.07801415558,6.59434488293349,2.29413876244471e-08,7.20894401303179e-07,9.00197547735894

"ULK2",1.01582481915833,-0.0668659557733333,6.59314980075671,2.3041672972148e-08,7.22713063784716e-07,8.99772038493349

"ZNF469",1.426751989825,-0.10629441814,6.59281538524958,2.30698137307304e-08,7.22713063784716e-07,8.99652970659961

"SYNGR2",-0.653969788325,0.02926757674,-6.59156537551889,2.31753050119673e-08,7.24909387916313e-07,8.99207911104899

"PNMA8A",2.5405956579,0.01852190432,6.59114406246601,2.3210969001223e-08,7.24918190391855e-07,8.99057905959978

"CDH6",1.128549921125,0.0779460219,6.58619080837736,2.36343906931692e-08,7.37018868373897e-07,8.97294385337925

"AC244197.2",1.087439201275,-0.09513519298,6.58539391421646,2.37032283426088e-08,7.38042161524876e-07,8.97010672870504

"SPATA18",1.05820543425,-0.0642671586,6.58265309371659,2.39415184565399e-08,7.4433054649103e-07,8.96034895293465

"ENTPD1-AS1",1.73411433495,-0.18017406444,6.58120922781189,2.40680098786037e-08,7.47129373322473e-07,8.95520865669314

"ELOVL5",-0.9577452539375,0.0628448908,-6.57924962637033,2.42407504914022e-08,7.51353246698712e-07,8.94823241179129

"CR1",-1.381380512925,0.05403262566,-6.57568755047813,2.45579258172058e-08,7.58888060547378e-07,8.935551654501

"ENO1",-0.986339100625,0.0460603355,-6.57307746409433,2.47929602132281e-08,7.64997242241893e-07,8.92626020528031

"ANKMY2",0.671146070125,-0.0689833819,6.56961873179971,2.51078760588864e-08,7.73549119841301e-07,8.91394810347787

"CEP164",1.0215849695,0.05588439235,6.56917541498069,2.51485274079568e-08,7.73638182483811e-07,8.91237005318239

"KLK3",1.4330357447625,-0.11016773379,6.56786721443745,2.52688703526141e-08,7.76174836258408e-07,8.90771336590844

"PRSS57",-4.75673784875,1.041180039,-6.56477584561708,2.55555377783306e-08,7.83805176650355e-07,8.89670953839829

"ADM",-2.578656439,0.1822861768,-6.56386000464393,2.56410866855919e-08,7.84989766447678e-07,8.89344964004351

"KRTAP19-5",-0.90934901895,0.00924802784,-6.56354273091214,2.56707899023791e-08,7.84989766447678e-07,8.89232032441424

"IL7",1.1711900845375,-0.05408496937,6.56300110532406,2.57215763458506e-08,7.85370575519801e-07,8.89039245122007

"LTBP3",1.4494355165875,-0.02419089973,6.55870420371214,2.61280520460885e-08,7.96594539167054e-07,8.8750983563895

"POMT1",0.658443562625,0.005010415,6.55009435121182,2.69619136431447e-08,8.19818518425797e-07,8.84445512050712

"AC241952.2",1.170810131375,0.0408332521,6.55001398942732,2.69698204519224e-08,8.19818518425797e-07,8.8441691189207

"NAB2",0.696403570825,-0.08135560884,6.54846327023827,2.71228498971551e-08,8.2324881287839e-07,8.83865027339508

"AC092747.2",0.93403368505,-0.02924857996,6.54647320072652,2.73205058586224e-08,8.28021485253632e-07,8.83156796121699

"ARHGAP21",0.718231619258333,-0.00719452119333333,6.54535556680626,2.74321401415197e-08,8.30176790575265e-07,8.82759056259159

"FUT10",0.6824449693625,0.04630177549,6.53837274399991,2.81399953928932e-08,8.50342515648371e-07,8.80274141743357

"C2orf42",0.61332778535,-0.09699283572,6.53611227779203,2.83730234191155e-08,8.5612150782156e-07,8.79469770638684

"ANKRD50",1.162500164825,-0.10586542314,6.53541225237925,2.84455777109345e-08,8.57048523737686e-07,8.79220675558193

"MPP6",0.868236459275,-0.11147620158,6.53419962934352,2.85716986718144e-08,8.5958437942457e-07,8.7878918374741

"CERS6",-1.051873829725,0.11151750532,-6.53133404298097,2.88719605981641e-08,8.66074273404372e-07,8.77769535447647

"C2orf27A",0.9156972752,-0.01521773484,6.52980019051541,2.90339723316788e-08,8.6839127756414e-07,8.77223765247628

"AL009178.2",1.360373206575,-0.17811888774,6.52918129079747,2.90995995938841e-08,8.69085417608598e-07,8.77003553098924

"MAP2",1.1435915466,-0.15665855122,6.5257307073001,2.94682170101642e-08,8.78813435377356e-07,8.75775821080669

"ZNF658B",0.86060619245,0.00623550355999999,6.52350074524473,2.97089130379576e-08,8.83939026337949e-07,8.74982416762478

"PADI4",-4.03940864125,0.695377347,-6.52333966027638,2.97263758857305e-08,8.83939026337949e-07,8.74925104678207

"ZNF441",0.6890009554,-0.05479681868,6.52256041394755,2.98109968841735e-08,8.85170585743402e-07,8.74647859740055

"ARHGAP12",0.620269263575,0.02419545346,6.51992811560745,3.00986282650476e-08,8.91729675049555e-07,8.73711343076157

"CAPS2",1.3262432092,-0.25420765464,6.51974313874849,3.01189445106546e-08,8.91729675049555e-07,8.73645533242427

"AC073111.4",1.0499310715,-0.0608418638,6.51736513699829,3.03813452741333e-08,8.98200580052587e-07,8.72799516098438

"SLC2A5",-0.991826285408333,0.0676355707066667,-6.51533154408357,3.06075506841774e-08,9.03584291667761e-07,8.72076047041147

"SYP",0.9044646239,0.01270610912,6.51352627859421,3.08097657591113e-08,9.08245296219672e-07,8.71433821818348

"DDX58",0.923541849775,-0.07996091458,6.51129904057969,3.10610852163694e-08,9.14338382058873e-07,8.70641497999098

"PCDHB8",0.743246939825,-0.10903265924,6.50904333309667,3.13177018271879e-08,9.20569691585976e-07,8.69839067415189

"PCDHB14",0.7949727811,-0.11722536142,6.5072647550655,3.15215290953488e-08,9.25233650580956e-07,8.69206382733361

"ZNF404",0.961642057425,-0.18266860906,6.50385671323303,3.19157998335234e-08,9.35466247481013e-07,8.67994094186606

"C11orf54",0.62067167105,-0.02633357976,6.50189588811762,3.21448701020756e-08,9.40834426644751e-07,8.67296623283019

"IFIT2",1.572235652425,0.02443268794,6.50010755899837,3.23552185761989e-08,9.45640111539463e-07,8.6666052392302

"PROM1",-1.494441508225,0.20215568442,-6.49854247989815,3.25404341448185e-08,9.49698596522852e-07,8.66103844624233

"NECTIN3",0.62151475365,-0.09217884948,6.49644493945219,3.27903223105277e-08,9.55630332145223e-07,8.65357791850191

"SMURF2",1.042015498,0.0828551774,6.49284688057924,3.32234394241583e-08,9.66877595059879e-07,8.6407807907857

"KLKP1",1.611543320425,0.00962587273999999,6.49112693663582,3.34324894357173e-08,9.71581338381527e-07,8.63466370625137

"HLTF",0.7777999615,-0.0558924678,6.48931962648173,3.36535723045346e-08,9.76620948123661e-07,8.62823603713735

"STXBP2",-1.19737717675,0.1679310727,-6.48882867188668,3.37138808050988e-08,9.76987255919185e-07,8.62648998972701

"ZFP3",0.645505574975,-0.01532418582,6.48728270072448,3.39044918889231e-08,9.81123205960814e-07,8.62099191482753

"PLXNA3",0.95333931905,0.03080232624,6.48310694899009,3.44247330371313e-08,9.94772821529966e-07,8.60614184190556

"CADM1",0.873758105275,-0.0873471918466667,6.47823024259053,3.50423915363656e-08,1.00970592477198e-06,8.5887999665118

"PANK1",1.1746316846,0.01211082868,6.47786299162492,3.5089350763259e-08,1.00970592477198e-06,8.58749404182618

"H2BC5",-1.511031015475,0.19875633462,-6.47312105917341,3.57013542376477e-08,1.02443885941306e-06,8.5706325284786

"MAMSTR",0.871941502425,-0.04068671406,6.47118847939654,3.59538201421878e-08,1.03024037352887e-06,8.56376089096733

"HMBS",-2.079741694375,0.3136268095,-6.46965813964762,3.61550014327615e-08,1.03455819742237e-06,8.55831961088489

"UCK2",-1.254713996525,0.13140631428,-6.46844534385291,3.63152349916954e-08,1.03769391145029e-06,8.55400746659372

"AC022101.1",-1.625671010925,0.27967049826,-6.46690605531142,3.65196248485138e-08,1.042080882864e-06,8.54853456082166

"CSF3R",-1.8862681565,0.2891704358,-6.46526572232264,3.67386945181164e-08,1.04511470904735e-06,8.54270251311852

"PURG",1.1702592968625,-0.09754877151,6.46518479024681,3.67495370193645e-08,1.04511470904735e-06,8.54241477002579

"PABPC5",0.93225327755,-0.02392048551,6.46496515149564,3.67789781932419e-08,1.04511470904735e-06,8.54163387555072

"BTF3",-0.8469413742,-0.03717405436,-6.46303722460829,3.7038416079454e-08,1.05102918093609e-06,8.53477949973948

"TMEM169",1.157963515575,-0.02855253454,6.46143712614557,3.72551241476704e-08,1.05571643642804e-06,8.52909078542037

"EMID1",1.417911190025,-0.10542268128,6.45837567447971,3.76732799719307e-08,1.06462091043437e-06,8.51820695824877

"PCDHB6",1.512183987425,-0.25734051006,6.45735951746243,3.78131063578508e-08,1.06619722769148e-06,8.51459449472558

"GSR",-0.70034533755,0.01580708521,-6.45721423013326,3.78331405960418e-08,1.06619722769148e-06,8.51407799858312

"CEP112",0.615696659325,-0.01206055454,6.45607484223044,3.79906227368088e-08,1.06916466845019e-06,8.51002751121817

"BTN3A1",1.4258759419125,0.10408262353,6.45091262195481,3.87123573338782e-08,1.08798186153154e-06,8.49167674694501

"CA2",-2.52749301405,0.24939136876,-6.44858683732485,3.90419799726186e-08,1.09574258312193e-06,8.48340941181551

"RPL22L1",-1.257695308725,0.08562030502,-6.44600188348101,3.94116185970165e-08,1.10460361397493e-06,8.47422112395377

"PGLYRP1",-3.94103511325,0.8801081424,-6.4393753436265,4.03752072779461e-08,1.1285228468084e-06,8.45066837058867

"SLC7A1",-1.252734066525,-0.00011850722,-6.4378463958358,4.06008519237413e-08,1.13328372508667e-06,8.44523432206053

"HMGB3P27",-1.30657490585,0.02038500732,-6.43521134394272,4.09926923676427e-08,1.14266432820172e-06,8.43586932422844

"STAR",-2.056422563125,0.1491145932,-6.43419125448726,4.11453933470927e-08,1.14536252567287e-06,8.43224400803285

"CTSL",-0.8179780089625,-0.09073905027,-6.43196777305613,4.14802029983637e-08,1.1531158738541e-06,8.42434210945298

"CCT2",-0.987445873,-0.000419085399999997,-6.42969987188346,4.18245000592834e-08,1.16111159514173e-06,8.41628260005767

"RHOBTB2",0.605976299175,-0.00995849556,6.42883432340418,4.19566516629787e-08,1.16320416680799e-06,8.41320674085403

"MPP3",0.77072843975,-0.0662508322,6.42838268854931,4.20257723193418e-08,1.16354597740362e-06,8.41160180184194

"OR51E1",1.52274607975,-0.03934081805,6.42745574450069,4.21679927567989e-08,1.16590801025816e-06,8.40830782576812

"VWCE",-0.77600100155,0.08613665611,-6.42524063892377,4.25098017168844e-08,1.17377468675947e-06,8.40043642834037

"ZYX",-1.028638662625,-0.0242380721,-6.42097632395874,4.31756181251793e-08,1.1905545950857e-06,8.38528383459045

"C1orf220",1.1768929592875,-0.07852032607,6.41632959096262,4.39129876453317e-08,1.20925979956661e-06,8.36877341427575

"VSTM1",-3.536393518725,0.45743284102,-6.41578831104813,4.39996933682875e-08,1.21002109762346e-06,8.36685025020868

"NOP16",-1.368816099325,0.16319830954,-6.41373536278648,4.43301021803196e-08,1.21747336926325e-06,8.35955627307531

"SIGLEC9",-0.836524578825,0.04273488044,-6.40955612545177,4.50103869942953e-08,1.23450175199347e-06,8.3447084066535

"SEMA3B",1.1229953102,-0.03066392784,6.40883294200701,4.51291567432465e-08,1.23610449646475e-06,8.34213919322512

"IQCG",0.708209688675,-0.01752744856,6.40733233339272,4.5376602244628e-08,1.24122273269418e-06,8.33680815041127

"LYNX1",1.230126174,-0.0790936278,6.40541556457801,4.56946395821698e-08,1.24825570101266e-06,8.32999883067278

"AC021231.2",-0.8277132166,-0.02888590828,-6.40134085212952,4.63781337153342e-08,1.26523995147772e-06,8.31552404854656

"MIS18BP1",-0.8544095069,0.02197214148,-6.40018765941451,4.65734146806526e-08,1.26887781911863e-06,8.31142766725364

"CLGN",-1.42906078645,0.15578674684,-6.39976763728197,4.66447444559601e-08,1.26913349855739e-06,8.30993567815115

"ZKSCAN4",0.6490425732875,-0.00471516182,6.39602001255606,4.72860210260925e-08,1.28487532995038e-06,8.29662389424408

"LINC01936",1.385225467475,-0.09963898802,6.39314190478365,4.77844701851942e-08,1.2939483628813e-06,8.28640118615824

"LCN2",-5.4595672335,0.3658945982,-6.39299876534611,4.78093962661627e-08,1.2939483628813e-06,8.2858927827183

"DIAPH1",-0.5897387575375,-0.04028396553,-6.39230418151594,4.7930534202346e-08,1.29531788389414e-06,8.28342577142311

"MGAM",-1.880594843675,0.07167264246,-6.39198419194056,4.79864444492217e-08,1.29531788389414e-06,8.28228924635078

"PPM1G",-0.9291107527,-0.02251214116,-6.38948224405839,4.84258452597373e-08,1.3054588390546e-06,8.27340312353056

"OPA3",-0.825421603325,0.03815253334,-6.38833083998439,4.86294042828909e-08,1.30820970221159e-06,8.26931381427457

"ZCCHC14",0.767302581975,-0.01231305057,6.3881830549809,4.86555931806535e-08,1.30820970221159e-06,8.268788948405

"GARNL3",0.84153026215,-0.10609359728,6.38700567338199,4.88647391327618e-08,1.31160539184818e-06,8.2646074600417

"FANK1",0.614323182741667,-0.0329017143066667,6.38648580536162,4.89573716592232e-08,1.31160539184818e-06,8.2627611645283

"CNTN1",1.3868298788875,-0.03779282839,6.38639291316267,4.8973942052121e-08,1.31160539184818e-06,8.26243126222009

"DPT",1.51964501655,-0.13132310976,6.38531480516587,4.91666677105534e-08,1.31504789563162e-06,8.25860244574748

"RAMP2",0.871951730425,0.03260357934,6.38184820823407,4.97915050780644e-08,1.33002393225474e-06,8.24629151945001

"NFATC1",0.835032713125,-0.0371029215,6.38057771760624,5.00224816799713e-08,1.33445391231673e-06,8.24177978776844

"LRR1",-1.069120878175,-0.00547800053999999,-6.37980195914051,5.01640402897971e-08,1.33649006171309e-06,8.23902497829977

"CCDC102A",0.91679420825,0.00899864919999999,6.37179621393531,5.16484389057058e-08,1.37425093025987e-06,8.21059754535577

"AC080013.1",1.5484290266,-0.18802856412,6.36449613875977,5.30401220445986e-08,1.40672511970119e-06,8.18467892451043

"XYLT2",0.5880656484,0.01100923472,6.36431617435017,5.30748983565511e-08,1.40672511970119e-06,8.18404000541576

"CROCC",1.0691333376125,0.03268951779,6.36319866682506,5.3291354738262e-08,1.41063730733529e-06,8.18007261185084

"SH3YL1",0.8047693043875,0.01177160901,6.36201381894709,5.35218157198875e-08,1.41490962641169e-06,8.17586622142344

"GLIS2",1.245115437975,-0.09858003262,6.36128966906775,5.3663156605711e-08,1.41681798007449e-06,8.17329541762205

"ZNF837",0.668254288675,-0.03104894806,6.35925428970988,5.40624210946827e-08,1.42552237244255e-06,8.16606977633332

"PDCD1LG2",1.0839945717875,0.00766913873,6.35888786204226,5.41346142015873e-08,1.42559122848602e-06,8.16476897474193

"NOLC1",-0.894185018,0.1116235166,-6.35422136897706,5.50624476888808e-08,1.44650166202794e-06,8.14820379670726

"AL358852.1",0.9929083063,-0.14942206276,6.35418440438956,5.50698602294901e-08,1.44650166202794e-06,8.14807258423621

"SGCD",1.67080396385,0.03298225308,6.35373803597447,5.51594494273171e-08,1.4469997437476e-06,8.1464881251345

"LINC01137",1.236641861925,-0.05064370346,6.35197705737351,5.55143090480769e-08,1.45368764496487e-06,8.1402373481453

"H3C3",-0.64514545635,-0.08565124208,-6.35176943312639,5.55562976380072e-08,1.45368764496487e-06,8.13950037584686

"ARHGEF17",0.685716198675,0.01056989694,6.34839202922927,5.62437841300898e-08,1.46792694172902e-06,8.12751246206033

"MS4A3",-3.522746899,0.3357064748,-6.34616195955459,5.6702366091389e-08,1.47625700440895e-06,8.11959730653454

"RYR1",1.35510491095,-0.00101527994,6.34613922221394,5.67070608390199e-08,1.47625700440895e-06,8.11951660667232

"AC117500.1",-0.74120185335,-0.01971614938,-6.34136098983227,5.77023019974273e-08,1.50025985193311e-06,8.10255826048962

"AC121247.2",1.2188068759,-0.06890334228,6.33613815025471,5.88100701597324e-08,1.52712384972446e-06,8.08402347700121

"MALL",0.7863844318,-0.02873603656,6.33569079411311,5.89059343750355e-08,1.52767694110852e-06,8.08243597586465

"TSTA3",-0.720956739475,0.00741152762,-6.33138207845368,5.98372614940441e-08,1.54829599021946e-06,8.06714654058979

"AL645568.1",0.995322782925,-0.11998108966,6.33131383711362,5.98521292587766e-08,1.54829599021946e-06,8.0669043956268

"EFCAB6",0.9048760798,-0.12749069396,6.32958590102061,6.02298244723416e-08,1.5561016945641e-06,8.06077314460385

"DMPK",0.96806534705,0.09902292064,6.32913082473945,6.03296903248288e-08,1.55671875991825e-06,8.05915842266673

"AC010182.1",0.79646269115,0.04260466592,6.32290120028888,6.17134780028744e-08,1.59042231109798e-06,8.03705542489423

"PLEKHH2",1.101541787925,-0.0761188648266667,6.32252206610751,6.17987088594897e-08,1.59061802401159e-06,8.03571031365188

"C14orf132",1.25025846475,-0.1459822942,6.31989815886987,6.23917970257511e-08,1.60386842843612e-06,8.02640131958197

"LRRC75B",0.853179864975,-0.01046615502,6.31749344099664,6.29403204361203e-08,1.61594146001909e-06,8.01787031930354

"ZFP37",1.10423409075,-0.1148117544,6.31485181245928,6.35484237078909e-08,1.62951202118557e-06,8.00849925373071

"H1-2",-1.348750505475,0.07612710862,-6.31303306163278,6.3970499605249e-08,1.63828449489043e-06,8.0020475571618

"CFAP44",1.113882727125,-0.0153114803,6.31111871763754,6.44177772256542e-08,1.64767967515506e-06,7.99525697743011

"ZNF528",1.210894344725,-0.11678404722,6.30442704261876,6.6005879414134e-08,1.68409521474069e-06,7.97152195844859

"TRAF1",1.24068357415,-0.07013700068,6.30011906705128,6.70488481427595e-08,1.70645565310417e-06,7.95624325104076

"IL1RAP",-0.853459894925,0.08994286156,-6.29609379359119,6.80382061810189e-08,1.72734419855851e-06,7.94196821606873

"GMNN",-0.85464248555,0.06838676306,-6.29072599378279,6.9380203636852e-08,1.75923466845523e-06,7.92293368304805

"CCDC170",1.091272709225,-0.09643602312,6.28808285423843,7.00506685013595e-08,1.77403967398746e-06,7.91356161975929

"AC097263.1",-0.9299865209,-0.03374952372,-6.28749436480874,7.02008224011781e-08,1.77564746833992e-06,7.91147500965369

"AC026410.2",-0.83928067775,0.0467665108,-6.28599640087476,7.05844783527212e-08,1.78315017569735e-06,7.90616376974911

"SMAD3",0.80693016445,-0.06948234544,6.28550073019644,7.07118880357112e-08,1.78416891881238e-06,7.90440633155521

"PTPRD-AS1",1.318898859425,-0.17536739346,6.28426047112631,7.10316951832649e-08,1.79003366656179e-06,7.90000896669614

"MBNL2",0.608456777175,-0.02205115346,6.28250443158535,7.14869646782001e-08,1.79901633550123e-06,7.89378305837089

"NDRG4",0.93951965865,-0.03560751048,6.28187227297643,7.16515682237898e-08,1.79901633550123e-06,7.89154183482376

"NADK",-0.86950516145,0.06720040214,-6.28115411935562,7.1839021562734e-08,1.80151514538225e-06,7.88899575977219

"AL449106.1",-1.65542122495,-0.10817415796,-6.28014536343751,7.21031535887788e-08,1.80592837497176e-06,7.88541946596795

"IRX5",0.933069777025,-0.06393999038,6.27908061867002,7.23829951756338e-08,1.8107238158222e-06,7.88164474816248

"RALGPS1",0.9176825709875,-0.05656656911,6.27578678978326,7.32555645028508e-08,1.82703384103375e-06,7.86996797366369

"C3orf52",-0.87520751965,-0.00768603572,-6.27561068801513,7.33025096314789e-08,1.82703384103375e-06,7.86934370445195

"RIPPLY2",1.493228304725,-0.16753874722,6.27341899363565,7.38892839298797e-08,1.83942120188988e-06,7.86157445894291

"ZNF547",0.7590819086125,-0.06979812541,6.27130629660438,7.44593367541464e-08,1.85136273230455e-06,7.85408554157434

"CARF",0.93780592155,-0.09626052876,6.26898448608659,7.50908667200196e-08,1.86480203316335e-06,7.84585570938663

"MARVELD1",0.7404147405375,0.01061241493,6.26833883411375,7.5267429523618e-08,1.86692384513303e-06,7.84356720962541

"PAICS",-0.9156815966,-0.00420532328,-6.26668495704709,7.57215945248257e-08,1.8759178097033e-06,7.83770520124063

"ITGA3",1.0435269709,0.04631787342,6.26251898699944,7.68777296764022e-08,1.90225957199291e-06,7.82294011309704

"AL355916.1",1.052418995225,-0.07838077182,6.26144359695954,7.71790149261049e-08,1.90615886069516e-06,7.8191288858707

"BPI",-3.78329948205,0.57617818236,-6.26102134094783,7.72976370188384e-08,1.90615886069516e-06,7.81763241372986

"SHOX2",1.281467462575,0.05304806206,6.26096162052727,7.73144286039475e-08,1.90615886069516e-06,7.8174207659547

"IL11RA",0.861608926925,-0.10890125146,6.26009316991859,7.75590217389106e-08,1.90989091032067e-06,7.81434302384573

"CCDC142",0.658993485825,0.04554061916,6.25814241752383,7.81112512679893e-08,1.92118045135482e-06,7.80742984405614

"UBN2",0.770365873125,-0.0512972435,6.2563132131504,7.86326294465434e-08,1.93168502649974e-06,7.80094764128041

"LOH12CR2",0.8343401509625,-0.08299721303,6.25441296664659,7.91779276018488e-08,1.94275135413974e-06,7.79421391959602

"ERI1",-0.6541114338,-0.03038219404,-6.25214166401302,7.98346479190771e-08,1.95652184995939e-06,7.78616563706735

"BNIP3L",-1.0034329537375,0.05780113031,-6.2490012888334,8.07515949542869e-08,1.97662924423349e-06,7.77503839351705

"GPR137C",1.275614900475,-0.07541296262,6.24715654600175,8.12951173913037e-08,1.98755890824944e-06,7.76850225259043

"ANKRD26",0.7998644978625,-0.07520094871,6.24651861153676,8.14839206042934e-08,1.98841928467239e-06,7.7662420289533

"TNFRSF10C",-1.180747034825,0.22642354914,-6.24638198201932,8.1524414248575e-08,1.98841928467239e-06,7.76575794946022

"SCARA3",0.849979623275,-0.01159354438,6.24419035880846,8.21767034990138e-08,2.00147477895785e-06,7.75799318258496

"PTPRK",0.8719104789625,0.01434974242,6.24392824016637,8.22550646174595e-08,2.00147477895785e-06,7.75706453638337

"HAUS8",-0.631091864175,-0.02389817089,-6.24055086756846,8.32714213964581e-08,2.02380175749779e-06,7.74509943868509

"AMD1",-0.601772780925,-0.00245017494,-6.23920742654325,8.36791728108601e-08,2.03130200538969e-06,7.74034021366406

"PRKD1",0.726056287375,-0.0723548301,6.23850559399604,8.38929779695678e-08,2.03408205046214e-06,7.73785397621015

"PCDHB2",1.373848747275,-0.25208963518,6.23727169521553,8.42701907558392e-08,2.04081284657876e-06,7.7334829788334

"ASAP3",0.670378197525,0.00393329622,6.23596946200982,8.46701259646259e-08,2.04807737988578e-06,7.72887002444408

"OLFML1",0.78620587025,-0.0027013488,6.23347854336287,8.54403971206288e-08,2.06427223609368e-06,7.72004666398783

"PWWP3A",0.781634608,-0.0418736406,6.2286431615467,8.6955626737418e-08,2.09490215133298e-06,7.7029199370724

"AREG",-3.06467296115,0.24978621763,-6.22845564805044,8.70149224318804e-08,2.09490215133298e-06,7.70225580434605

"ENAH",1.04866683880833,-0.0285706577533333,6.22804233818273,8.71457618853522e-08,2.09558963557171e-06,7.70079195772271

"GSPT1",-0.6896254459,0.08137583768,-6.22370213205902,8.85316053124208e-08,2.12641914377594e-06,7.68542068098191

"ORM2",-1.319789411225,-0.05030747098,-6.22323468373696,8.86821660888576e-08,2.1270733968238e-06,7.68376524384918

"CACNA1E",1.593198423475,0.05595122378,6.22109385592139,8.93749711101001e-08,2.13915234591557e-06,7.67618383938627

"DEPDC1",-1.5354108885,0.00585364320000001,-6.22043930914441,8.95878663752217e-08,2.14174586498896e-06,7.67386592902514

"MEGF10",1.8458239534,-0.22084692828,6.21773818463821,9.04717779245896e-08,2.1578414273795e-06,7.66430090354764

"CORO2A",-1.427225216575,0.11641361974,-6.21602498814249,9.10368963728126e-08,2.16879527079789e-06,7.65823452246979

"SLC19A2",-1.21230956885,0.10085585492,-6.20437446322336,9.49743031602007e-08,2.25536396780432e-06,7.61698585011074

"NDUFAF4P2",1.109821396675,-0.08089274466,6.20429731736647,9.50009324587626e-08,2.25536396780432e-06,7.61671274751709

"GOPC",0.667620321875,-0.0197716525,6.20364892202823,9.52250403288903e-08,2.25806785446563e-06,7.61441739226719

"SLC35B4",0.6936298236,0.03199555388,6.20322315439278,9.53724865682135e-08,2.25894971654284e-06,7.61291016776421

"ATP1A1-AS1",0.93131800455,0.00136982964,6.19798833433677,9.72040253840629e-08,2.29967213864744e-06,7.59437988383101

"RTKN",1.17485890005,-0.06180704096,6.19297354450527,9.89914092465341e-08,2.33925720028027e-06,7.57663032435453

"POGZ",0.709249018575,-0.04909146614,6.18910334317041,1.00393172927112e-07,2.36692212535175e-06,7.56293322169743

"AL356653.1",-0.9155533506,0.06722648752,-6.18754008109626,1.00964963953943e-07,2.377666875274e-06,7.55740096211326

"INPP5D",-0.941316962725,-0.02361640918,-6.18705207234455,1.01144124987366e-07,2.37915135791177e-06,7.55567397485656

"DIS3",0.7466638995,-0.0211425598,6.18570551124571,1.01640126602604e-07,2.38586311841388e-06,7.5509087948283

"C19orf38",-1.223490041275,0.06495605408,-6.18564529524768,1.01662363450572e-07,2.38586311841388e-06,7.55069570692905

"MCTP2",-1.452561889525,0.10837502623,-6.18349163294246,1.02460870118298e-07,2.40185160982115e-06,7.54307466245962

"KLHL3",1.0968051441,-0.11566854442,6.18116706089975,1.03329751826341e-07,2.41945137762067e-06,7.53484921359601

"SLC27A2",-1.554952576075,-0.05661760386,-6.18008864749102,1.03735329451666e-07,2.42617514818007e-06,7.53103340647231

"RFC2",-0.752029493625,-0.0212382879,-6.17563507740783,1.05427113516162e-07,2.46293124483366e-06,7.51527601958836

"SREBF1",-0.6869616506375,0.09800028719,-6.175314750146,1.05549850087816e-07,2.46299012368926e-06,7.51414271153001

"PTH2R",-1.951775739725,0.13326726262,-6.17285154869559,1.06498414279602e-07,2.48229751053526e-06,7.50542823641797

"HOXD3",0.7601771039125,-0.04643318537,6.16997318818294,1.07617609578333e-07,2.50553361936464e-06,7.49524556070443

"EFCAB7",0.734742163525,-0.05375504618,6.16890193431794,1.08037128806158e-07,2.51244573777589e-06,7.49145598228217

"GFOD2",-0.5886692977,0.06385412184,-6.16685494081386,1.08843294614138e-07,2.52832360550393e-06,7.48421494923122

"DEPDC7",1.1506379545,-0.1090779607,6.16199984148537,1.10779396363156e-07,2.57006054464296e-06,7.46704179300036

"JMJD6",-1.1385642638,0.05383698396,-6.16172271292773,1.10890937205407e-07,2.57006054464296e-06,7.46606160504775

"CACNA1A",0.90683919345,-0.02615866854,6.15935454245305,1.11848667000172e-07,2.58932823672264e-06,7.45768575935901

"RAB22A",0.703233945725,0.05669707258,6.15644475860889,1.13036713693518e-07,2.61093144323877e-06,7.44739489908723

"CCDC102B",1.5873722542625,0.02961309941,6.15416651650993,1.13975665113812e-07,2.62965475996822e-06,7.43933802809447

"ZNRD1",-0.771213091925,0.04865756736,-6.15380107364733,1.14126998951707e-07,2.63018442578467e-06,7.43804569773459

"KIAA1522",0.9090144183,-0.08262447436,6.15111709895397,1.15244606767033e-07,2.65295674544154e-06,7.42855456481598

"HAL",-2.16874850225,-0.0580491658,-6.14994296545188,1.15736934552297e-07,2.66130001695563e-06,7.42440274178216

"FAM66C",0.990151975025,-0.10284183398,6.14291379041404,1.18728432026364e-07,2.72440749367502e-06,7.39954929438839

"TDRD3",0.726434705325,0.00286441326,6.14222980944763,1.19023599027988e-07,2.72769071239981e-06,7.39713111004409

"STOM",-1.31427985285,-0.05956359528,-6.13748057521005,1.21093333215071e-07,2.7664747580117e-06,7.38034141361766

"FBXO44",0.7459129696125,0.00572540919,6.13741754940052,1.21121039532238e-07,2.7664747580117e-06,7.38011861398024

"CGNL1",1.059852005675,-0.03867239946,6.13670423722667,1.21435053561194e-07,2.77055832668345e-06,7.37759703874901

"CYP2U1",0.873179330725,0.05039239828,6.1342264900199,1.22532114127453e-07,2.7881223498031e-06,7.36883845597176

"SLC25A5P7",-1.3036233216,-0.00289451528,-6.13404434211723,1.22613150974843e-07,2.7881223498031e-06,7.36819460090128

"SYT12",1.09500318605,-0.000297294160000004,6.13003009347787,1.2441267453087e-07,2.82590562725994e-06,7.35400573111242

"PLK2",1.0171991333,-0.14778224936,6.12636902849788,1.26076773625903e-07,2.85758397655896e-06,7.34106634996279

"EDA2R",1.0829021214625,-0.02216282803,6.1263482302137,1.26086290258166e-07,2.85758397655896e-06,7.34099284518967

"EIF3K",-0.597245000975,-0.01337310978,-6.12483406767384,1.26781046457697e-07,2.86721224036858e-06,7.33564162286746

"DUS1L",-0.6361637519,0.00875664873000001,-6.12481240492003,1.2679101375312e-07,2.86721224036858e-06,7.33556506556632

"AL158801.6",-0.7028628302,0.00489942484,-6.12014150696387,1.28958461010591e-07,2.91152537859901e-06,7.31905874748418

"PLCB3",0.62408222135,0.03376031908,6.11997841583089,1.2903480299531e-07,2.91152537859901e-06,7.31848243727879

"AGFG1",-0.7782289850625,0.0605895131,-6.11912074714995,1.29437013214659e-07,2.91603722501446e-06,7.31545175465761

"PBX4",0.864884706675,-0.02580425266,6.11865128656325,1.296576983259e-07,2.91603722501446e-06,7.31379288039197

"ARL6",0.75103659875,-0.003008168,6.11864267720695,1.29661748925623e-07,2.91603722501446e-06,7.31376245875044

"VASH1",1.05791591205,-0.06412517736,6.11822079927384,1.29860391801531e-07,2.91730231055895e-06,7.31227173682889

"BBS2",0.736919546125,-0.0947776611,6.1177507376205,1.30082078828941e-07,2.91908174265864e-06,7.31061077279917

"VAPB",0.7638595619,-0.04227272048,6.10613984275498,1.35679050049741e-07,3.04134833415656e-06,7.26958933960718

"AGRN",1.120686657,0.00905246690000001,6.10403070508187,1.36721164219887e-07,3.06135870222628e-06,7.26213891223306

"RETREG1",-1.6549788705,0.1457469922,-6.10284155954381,1.37312221429491e-07,3.07123667319587e-06,7.25793847403887

"DLX6-AS1",0.779593668875,0.00864965510000001,6.10139959556302,1.38032351559701e-07,3.08065978846556e-06,7.25284515740325

"PCDHB10",0.913858415325,-0.24456372374,6.10114106406593,1.38161861212633e-07,3.08065978846556e-06,7.25193198852486

"FKBP1A",-0.7608683011375,0.13031616559,-6.10001124578545,1.38729256214119e-07,3.08944021882051e-06,7.2479413794167

"HPCA",1.1131031399,-0.09310027208,6.09164478076658,1.43003664258997e-07,3.17772133767715e-06,7.21839364605117

"ZNF624",0.953660457625,-0.0587186983,6.08911771572414,1.44320313855075e-07,3.20198543262535e-06,7.20946999146077

"RARB",0.9432092669125,0.05205526903,6.08894991365698,1.44408167695521e-07,3.20198543262535e-06,7.20887746226079

"CCDC121",0.9321739559125,-0.00910886177,6.08800047044622,1.44906257740644e-07,3.20955611739492e-06,7.20552490904574

"FBXO9",-0.625444936975,0.02656656152,-6.0874418903464,1.45200094086649e-07,3.21259128255643e-06,7.20355255733777

"LINC00892",1.75095208225,-0.0572410862,6.0870764866999,1.45392632190505e-07,3.21338106614786e-06,7.2022623277292

"AVIL",0.974452948875,0.0508341121,6.08623317834128,1.45837957297305e-07,3.21975007446895e-06,7.19928467338746

"AC006116.3",0.613759884825,-0.05399183214,6.08475280769066,1.46622979185188e-07,3.23359698336504e-06,7.19405774784973

"FRMD4B",0.81717205695,0.00245532896,6.08387700964271,1.47089381298071e-07,3.24039488605901e-06,7.19096554764399

"IGSF9B",1.1685403618625,-0.12940965301,6.08042288226614,1.4894329255166e-07,3.27771232846231e-06,7.1787706130178

"CLC",-4.481398762625,0.6719035649,-6.08005187707542,1.49143796865971e-07,3.27860312252148e-06,7.17746082446017

"AC093525.6",0.8781954898,-0.02093327456,6.07957880339498,1.49399852076024e-07,3.28071186423748e-06,7.17579071249005

"TMEM183A",-0.684514306075,0.00913627414,-6.07886339240911,1.49787905302892e-07,3.28571156728657e-06,7.17326510299495

"ZNF610",0.7963956541,-0.03442464962,6.07737576131232,1.50598038963692e-07,3.2999493286504e-06,7.16801347047605

"GCDH",-0.773110399275,0.01570320158,-6.07626480177065,1.51205886266411e-07,3.30972884383143e-06,7.16409168601844

"TMEM30B",0.980935765325,-0.04385980674,6.07260752377178,1.53224211046251e-07,3.34966757548897e-06,7.15118191845471

"TEX41",0.694082515716667,-0.04432654616,6.0723677128474,1.53357486616978e-07,3.34966757548897e-06,7.15033545363536

"TIMM23",-0.6842786315625,-0.00812317825,-6.06990366538483,1.54733584178755e-07,3.37612531699077e-06,7.1416383477698

"MRPL37",-0.659015381875,-9.69850000000196e-06,-6.06917654542306,1.55142000946922e-07,3.38143544191546e-06,7.13907200360572

"GPER1",0.76371429723125,-0.06478318549,6.06639539485546,1.56714065867372e-07,3.41206990594126e-06,7.12925645107056

"ALOX12",-2.50223624175,0.1236262689,-6.06407857239718,1.58035755227594e-07,3.43719379310292e-06,7.12108016763775

"FBXO7",-0.817786570875,0.0465494142,-6.06223114880003,1.59097603983555e-07,3.45483467521783e-06,7.11456076963446

"CBR3",1.119440187425,-0.15644311776,6.06208119418767,1.59184104519994e-07,3.45483467521783e-06,7.11403160576785

"AL139128.1",-0.78684082175,0.0463924216,-6.06157066876421,1.59478949290688e-07,3.45603628588988e-06,7.11223006456386

"KIAA1841",1.0137580618,-0.05623573846,6.06140137557305,1.59576841392612e-07,3.45603628588988e-06,7.11163266804873

"RGS19",-0.934926846375,0.1649588779,-6.06091750419026,1.59856964825203e-07,3.45844719676744e-06,7.10992521158146

"GLT8D2",1.4504762552375,-0.00955526346,6.05559743782375,1.6296929863532e-07,3.51835088560636e-06,7.09115342316502

"RRS1",-0.983171084625,0.1325067703,-6.05384559529842,1.64007289157107e-07,3.53703298973769e-06,7.08497260957123

"TOMM22",-0.695661245825,-0.00236955806,-6.05234879398254,1.64899371155308e-07,3.55253240402728e-06,7.07969184009479

"PRR13P5",-0.7199117004,0.04214239228,-6.05188279266024,1.65178088111784e-07,3.55479902230486e-06,7.07804781063672

"COL22A1",2.3124342562125,-0.16609879453,6.05013422284475,1.66228096963844e-07,3.5736424455354e-06,7.07187911366421

"PTRH2",-0.768578914375,-0.0170562934,-6.04956607730196,1.66570692663377e-07,3.57725403698875e-06,7.0698748377414

"LYN",-0.69634744985,-0.00671194288,-6.04831343498955,1.67328527381281e-07,3.58976635496092e-06,7.06545592830145

"SMIM24",-2.48785558635,0.46497784242,-6.04744740629337,1.67854468826413e-07,3.59728280053928e-06,7.0624009453189

"TRDC",1.39038350695,-0.04211061514,6.0469667625475,1.68147074031865e-07,3.59978814290999e-06,7.06070546605176

"APMAP",-1.356519893125,-0.0441041695,-6.04617506037088,1.68630150926785e-07,3.60636172462211e-06,7.05791276724167

"MTMR11",1.1431536697,-0.16379723624,6.04440071449754,1.69717830863911e-07,3.62583828857124e-06,7.05165403237758

"PYURF",-0.771410535875,-0.0048355679,-6.04224227749966,1.71050369035825e-07,3.65049995917291e-06,7.04404085112524

"SIRT4",0.915135715575,-0.09937674494,6.03866702541578,1.7328052224338e-07,3.69424697161536e-06,7.031431229772

"AATF",-0.645083131525,0.03112052678,-6.03790555437592,1.73759230867676e-07,3.70060199793861e-06,7.02874573110161

"LIMS2",1.00333910466667,0.1463934692,6.03754729423355,1.73984910411815e-07,3.70156058620692e-06,7.02748226416662

"SENP7",0.6354841157875,0.01869727553,6.03460899761103,1.75846869257729e-07,3.7372932130211e-06,7.0171202818966

"PPIAP72",-0.66756103235,0.01555063212,-6.0335949227815,1.76494062970187e-07,3.74716099702921e-06,7.01354429944769

"SYT7",1.021237215075,-0.03103006094,6.03082538828528,1.78273702555435e-07,3.77818045020904e-06,7.00377842369449

"CDH23",0.913621663758333,0.0613272311733333,6.03074763592251,1.7832392109294e-07,3.77818045020904e-06,7.00350426488043

"GGCT",-0.704722785216667,0.0787410073266667,-6.02973927896208,1.78976472101395e-07,3.78808880207994e-06,6.99994879599895

"SLC4A1",-2.26415118311875,0.18951978723,-6.02604654378138,1.81386532445652e-07,3.83095099503156e-06,6.98692898836132

"MDFI",1.068620595175,-0.10475424046,6.02602161083898,1.81402914088983e-07,3.83095099503156e-06,6.9868410842485

"HCK",-0.9475245382,-0.03566492956,-6.02542809067224,1.81793308087386e-07,3.83187376141395e-06,6.98474857356137

"PTAFR",-0.788268709575,0.04335268249,-6.02236918205889,1.83818622298874e-07,3.87058163788215e-06,6.97396461373115

"H2AC14",-1.3998724332,0.16772651604,-6.02170150683038,1.84263666442321e-07,3.87596919719741e-06,6.97161088653922

"ANKRD9",-0.957288095875,0.071908703,-6.02043067344188,1.85113712515915e-07,3.88866374089327e-06,6.96713098381244

"NFKBIB",-0.6768156019375,0.0141770132,-6.02023227647713,1.85246769382655e-07,3.88866374089327e-06,6.96643161424907

"HSPB2",0.669820498125,-0.0059152028,6.01822313360985,1.86599589463145e-07,3.9130525986908e-06,6.95934938321001

"TSR1",-0.74018796845,0.06358424924,-6.01763398901393,1.86998138467802e-07,3.91740067579583e-06,6.95727271785663

"HSPA9",-0.70814604825,0.0230667509,-6.01630444047503,1.8790067426685e-07,3.93228704226683e-06,6.95258633269499

"MAP1LC3B2",-0.72449114615,-0.00398818792,-6.0144956654451,1.89135480089858e-07,3.95408950620511e-06,6.94621103331609

"AC092376.1",1.003239672,-0.1154211234,6.01325728006468,1.89985544187485e-07,3.96781226229684e-06,6.94184633150936

"DTX3",0.81356625175,-0.0506963926,6.01029344407541,1.92035463025164e-07,4.00654029171034e-06,6.93140083574199

"UBE2D1",-0.772839450225,0.04459503182,-6.00812879155678,1.93546511858401e-07,4.03395822477611e-06,6.92377242847269

"LINC00607",1.69605965105,-0.27480806916,6.00393372781456,1.96508631735557e-07,4.09153338109562e-06,6.90898992859436

"C18orf54",1.308167540575,-0.13030912354,6.00333449748424,1.96935408494258e-07,4.09625649668057e-06,6.90687850379209

"NAP1L3",0.835733974,-0.0837695403,6.00224692100065,1.97712344434072e-07,4.10824595615139e-06,6.90304644692433

"HERPUD1",-0.6974988488,0.04637990856,-6.00096356902148,1.98633057104381e-07,4.11902234205928e-06,6.89852472054111

"GCH1",-0.9013350505375,0.00921567472,-6.00021895119958,1.99169219395337e-07,4.12596457732221e-06,6.89590122556602

"PDE11A",2.0804360387125,0.01494058247,5.99797045082109,2.00796999510794e-07,4.15548376361327e-06,6.88797945061176

"HSPG2",1.072599345325,0.10986399026,5.99566388796345,2.02480554700457e-07,4.18609647295959e-06,6.87985360654346

"ZNF541",1.05440853695,0.08221100156,5.99435627970637,2.03441199296185e-07,4.19748569101735e-06,6.87524722517786

"VSTM4",0.7859453644125,-0.04997195657,5.99396082584381,2.03732613793899e-07,4.19926940785653e-06,6.87385417048211

"LYPD1",1.602305199175,-0.08816084066,5.99202257143445,2.05166947559327e-07,4.22458333828692e-06,6.86702654712041

"PTPN11",-0.90286124785,0.25527702762,-5.98665327546656,2.09192925791116e-07,4.2988411871699e-06,6.84811471994807

"PNMA8B",0.6151156119625,-0.04814915623,5.98607842904242,2.09628580614105e-07,4.30347731425029e-06,6.84609014809468

"MMP15",0.804124905975,-0.06864856782,5.98560018455583,2.09991711520341e-07,4.30661680243118e-06,6.84440582596788

"FERMT3",-1.0442977603,0.05277236676,-5.9840207451099,2.11195427357075e-07,4.32264926642532e-06,6.83884337652497

"SLC44A3",1.624072490325,-0.00731743874,5.98345402409292,2.11629004211306e-07,4.32720063700723e-06,6.83684756375533

"C20orf27",-0.909411673675,0.08776393496,-5.98160397007029,2.13050578254369e-07,4.35192447385396e-06,6.83033247020313

"HEMK1",1.396213246,-0.0826276692,5.97984768566289,2.14408880402327e-07,4.37140720853604e-06,6.824147895798

"BDH2",0.604867813575,-0.08394322214,5.97960890413421,2.14594216342632e-07,4.37140720853604e-06,6.82330707409129

"UPF3AP2",0.642075153,0.0751137544,5.97954420976483,2.14644457818589e-07,4.37140720853604e-06,6.82307926666571

"SLC26A8",-2.70671689315,0.27507924248,-5.97784383546762,2.15969163869862e-07,4.3896589577041e-06,6.81709190517982

"ARSL",1.193947255775,-0.18356470038,5.97615907054608,2.17289722769892e-07,4.40963270175599e-06,6.81115978145188

"SERP2",0.608553565325,-0.04039289474,5.97604131385489,2.17382322763254e-07,4.40963270175599e-06,6.8107451657097

"SFRP4",1.332511906125,-0.2503067029,5.97557106328348,2.17752503568644e-07,4.41277279239801e-06,6.80908944899512

"TRH",-1.633470946575,0.04073725974,-5.97111283639776,2.2129328897537e-07,4.48009575546185e-06,6.7933934291797

"NDUFV3",-0.628481225075,0.05031723194,-5.96936976028737,2.22693171707171e-07,4.50398588542598e-06,6.78725712725426

"PPP1R13L",0.8483513366,-0.07376196132,5.96900744968073,2.22985250074295e-07,4.50544556560371e-06,6.78598169080992

"ISM1",1.47585218745,-0.04778028704,5.96858434048361,2.2332682343929e-07,4.50790143706816e-06,6.7844922421552

"CD163",-1.737765890175,0.22366716586,-5.9664018268697,2.25097029059874e-07,4.53916134978218e-06,6.77680953267749

"AHNAK2",1.4624446659875,0.02403602629,5.9653220591774,2.25977962169846e-07,4.55244492520729e-06,6.77300879242009

"A2M",0.7715673679,-0.07698019068,5.96485612606269,2.26359153102902e-07,4.55564472374486e-06,6.77136876108645

"TRIM7",0.766991411225,-0.01164700602,5.96288233811557,2.27981055661682e-07,4.57901923860572e-06,6.76442148800493

"SLC16A8",1.2120013208,-0.11088911336,5.96262778041138,2.28191070022279e-07,4.57901923860572e-06,6.76352553198594

"DDX21",-1.646420752075,0.02877823554,-5.95908685904988,2.31132404418394e-07,4.6335036220392e-06,6.75106335834982

"CYC1",-0.929025742375,-0.0107257699,-5.95476350085029,2.34774811763878e-07,4.69350086571333e-06,6.73584908997834

"THAP10",0.952209883225,-0.11124200712,5.95468483374477,2.34841614063266e-07,4.69350086571333e-06,6.73557227074481

"FAM161A",0.70387776895,-0.00518520369,5.95451970255121,2.34981901123693e-07,4.69350086571333e-06,6.73499119770493

"VRK1",-0.781440414825,0.15708748784,-5.95419784196338,2.35255576887274e-07,4.69350086571333e-06,6.73385862409111

"FAM49A",-0.823912215175,0.02268546986,-5.95418010791779,2.3527066522294e-07,4.69350086571333e-06,6.73379622124599

"EMX2OS",2.56118336985,-0.17918759042,5.95161442031099,2.37463746565551e-07,4.73264322921693e-06,6.72476836597781

"GPAT3",-1.1465723449,-0.06309842392,-5.94910606552946,2.39627456574749e-07,4.77112471360881e-06,6.71594287730554

"PYROXD2",1.017238306475,0.02226790288,5.94293204318355,2.45036988080056e-07,4.87409496289728e-06,6.69422263349246

"FGFR2",0.836197940783333,-0.0813705967066667,5.9413810608194,2.46414883739563e-07,4.89674892148998e-06,6.6887668673541

"TSPY26P",1.231644405025,-0.01784794698,5.93718522126233,2.50181180115228e-07,4.96196710377617e-06,6.67400870866272

"CALCR",1.3642984202,-0.03773046984,5.93585359185585,2.51388400278907e-07,4.98108853473331e-06,6.6693252967051

"STARD9",0.8179542840625,-0.007097171575,5.93543338214789,2.51770551352421e-07,4.98384063392117e-06,6.6678474337505

"SIVA1",-0.591765813825,0.03164497494,-5.93359198059365,2.53451993919712e-07,5.01228228902227e-06,6.6613714985731

"TRO",1.070708853475,-0.09453711172,5.93071937210276,2.56097369002044e-07,5.05507399562655e-06,6.65126964677802

"SLF2",0.78832539711875,-0.042863753905,5.9305035556088,2.56297217953829e-07,5.05507399562655e-06,6.65051073749602

"SNX5",-0.60988738715,0.01306436628,-5.93044010072764,2.56356007490042e-07,5.05507399562655e-06,6.65028760207456

"PLAAT4",0.8484286093,0.05035247524,5.92899234041859,2.57700971088785e-07,5.07670913044907e-06,6.6451967476693

"ARPIN",0.66532263915,0.06788604802,5.92788368331787,2.5873564673797e-07,5.09219589852789e-06,6.6412984480585

"TMEM98",1.062474391425,-0.08359315886,5.92539868560551,2.6106983604094e-07,5.133204223423e-06,6.63256106502888

"PRR29",0.768567951625,-0.0534066897,5.92442727242446,2.61987973394294e-07,5.1396189643355e-06,6.62914569621932

"CYP4F3",-3.711840129,0.6042257968,-5.92435835314284,2.62053234624482e-07,5.1396189643355e-06,6.62890338819431

"DOK4",0.6464366307,-0.08027997044,5.9242576059385,2.62148663497198e-07,5.1396189643355e-06,6.62854917968584

"WNT5A",1.70350437895,0.37444801316,5.9220045088903,2.64291864557587e-07,5.17668424575128e-06,6.62062797890722

"EXTL3-AS1",0.966650973725,-0.06109303402,5.91891510455313,2.67258905618085e-07,5.22979986466412e-06,6.60976742261942

"UPP1",-1.098896878525,0.17252060688,-5.91794469932937,2.68197687144481e-07,5.24316241814517e-06,6.60635624216525

"ATP10D",0.814011002925,-0.05370348036,5.91744021385652,2.68687025807439e-07,5.24772143445454e-06,6.60458290685116

"HK1",-0.88598169275,-0.000249974499999998,-5.91442597167191,2.71629303381574e-07,5.30013444541112e-06,6.5939879803947

"MPC2",-0.795522431025,0.03818036918,-5.91154609094022,2.74470318258473e-07,5.35047372072274e-06,6.58386620489803

"MACORIS",1.258979237925,-0.15559907566,5.908089295093,2.77919465203745e-07,5.41256083944328e-06,6.57171790909947

"TMBIM6",-0.5908375186375,0.01894895664,-5.90556806862328,2.80462232911962e-07,5.45363957120537e-06,6.56285828939509

"CORO1A",-1.366208567075,0.16132953234,-5.90533388112763,2.80699590970236e-07,5.45363957120537e-06,6.5620353853793

"NEIL1",0.72637162885,-0.02264139092,5.90520795816101,2.80827301231046e-07,5.45363957120537e-06,6.56159291102453

"OSER1",-0.664130595375,0.0306420143,-5.90233694715689,2.83754771980024e-07,5.50527250788516e-06,6.55150505971057

"MYO1H",1.486442293475,0.10580493678,5.90106167410094,2.85064827247535e-07,5.52545712454824e-06,6.54702441822707

"COL2A1",4.1885828275,-0.501782049,5.90021464316101,2.85938278840688e-07,5.53714882503593e-06,6.54404848940083

"PCMTD2",0.677520910375,-0.0051689337,5.89637120189949,2.89935119946728e-07,5.60395352591373e-06,6.53054602418003

"COQ2",-0.67710199605,0.01809900316,-5.89577138276867,2.90563865047468e-07,5.61081288133132e-06,6.52843892861805

"PLCB4",0.961890579625,-0.0996363882,5.89525950025275,2.91101503686481e-07,5.61590170200436e-06,6.52664077425571

"SMIM17",0.9777982245,-0.0625429144,5.89215422233952,2.94384270951577e-07,5.67388988076756e-06,6.51573306528413

"BMERB1",0.76512169815,-0.06873617198,5.890074909827,2.96602957560656e-07,5.70591680234997e-06,6.50842976891137

"SALL1",1.31779742005,0.31339588194,5.88743178681035,2.99447233696241e-07,5.74983591749633e-06,6.49914682861534

"PSMD8",-0.835141092275,0.12613914618,-5.88632748962955,3.00643580501495e-07,5.76740231958299e-06,6.49526863480477

"THAP2",1.076952930925,-0.17206808226,5.88490323742941,3.02193563985827e-07,5.78630069059966e-06,6.49026697913622

"DBP",1.30581185745,-0.00597205003999999,5.88292441190977,3.0436025900219e-07,5.81845251070134e-06,6.48331814506444

"RFESD",-1.561576151575,0.21253890374,-5.88285121406674,3.04440701458016e-07,5.81845251070134e-06,6.48306111189796

"DCAF12",-0.77338407845,0.09255610324,-5.88186340167259,3.05528348010629e-07,5.83379757133436e-06,6.47959247901583

"JAG2",0.9032392798,0.07871916784,5.88117450541395,3.06289151348279e-07,5.84287908084129e-06,6.47717353066369

"OPHN1",0.761376175775,0.04294689262,5.88039778483134,3.07149202804537e-07,5.8538352251715e-06,6.47444626229639

"HNRNPC",-0.5893108530375,0.03249128377,-5.88007894005809,3.07502948942609e-07,5.85513049994068e-06,6.47332673393092

"MSRB1",-0.675924394925,0.04613256546,-5.87950027682817,3.08145987545483e-07,5.86192664144091e-06,6.47129495836201

"SNHG6",-0.689123484175,0.01574317766,-5.87866735805689,3.09073912319789e-07,5.87412459703881e-06,6.46837051557997

"B3GALT2",1.93984705355,0.02972980284,5.87676449149392,3.11204251680434e-07,5.90913133311281e-06,6.46168968228771

"PTDSS1",-0.72708105965,0.03637485728,-5.87629310567486,3.11734237605865e-07,5.91371394450831e-06,6.46003473937223

"FRMD3",0.74196550170625,0.00623458949,5.87443733961565,3.13829426614259e-07,5.94780564738825e-06,6.45351974227929

"SNRPD1",-0.859556153575,-0.01968799436,-5.87418803941196,3.1411195384977e-07,5.94780564738825e-06,6.45264455782981

"CS",-0.60655223745,0.04780616604,-5.87163114609913,3.1702425248143e-07,5.99740801924242e-06,6.44366880636878

"SEC14L1",-0.8019624629375,0.03498689505,-5.86532809946994,3.24318435652243e-07,6.12408858031628e-06,6.42154553534395

"IRAK1BP1",0.594767520625,0.0020138841,5.86385814626031,3.26043357642099e-07,6.15099107861079e-06,6.416386719488

"ZSWIM5",0.7240063582,-0.11503009844,5.86280530267184,3.27284416762576e-07,6.16872413121588e-06,6.41269189901748

"ZNF599",0.763481532933333,-0.0600541224866667,5.86061087364481,3.29886238166493e-07,6.21204894076757e-06,6.40499121923875

"AKNAD1",0.757651734575,-0.04443694184,5.85477527627813,3.3690555735375e-07,6.33258812758132e-06,6.38451553790549

"ZNF213-AS1",0.656045733275,-0.09123583538,5.84991598847248,3.42863532766734e-07,6.438669165284e-06,6.36746834961311

"DCAF11",-0.870308519475,0.10643224852,-5.84851107030525,3.44605515818766e-07,6.46545586821875e-06,6.3625401501958

"AC073343.2",0.88203200055,-0.06211736756,5.84807352456417,3.45149828893241e-07,6.46974354470698e-06,6.36100536338163

"CPLANE1",0.64477798626875,0.024913802565,5.84630292640813,3.47361207602177e-07,6.50524352957348e-06,6.35479482242352

"TRANK1",1.0252645104,-0.06575182908,5.84449821637954,3.49629665531952e-07,6.54174665517683e-06,6.34846498950367

"EIF4A3",-0.87518696455,0.00807100396,-5.84404310731719,3.50204040127946e-07,6.54651493990999e-06,6.34686879900212

"AHRR",1.323505098975,-0.12014865582,5.84350210728315,3.50888032980878e-07,6.548915465748e-06,6.3449713956731

"SLCO4A1",-2.01951628335,0.48994024632,-5.84306554569933,3.51440949852148e-07,6.548915465748e-06,6.34344030421433

"DUSP16",-0.796302877225,-0.00558038478,-5.84301872002371,3.515003071572e-07,6.548915465748e-06,6.34327608032574

"NRXN3",1.1722466218875,-0.09946113699,5.84274379610236,3.51849007867818e-07,6.548915465748e-06,6.34231189040672

"LINC01140",0.8091811317375,0.07734876069,5.84267943695423,3.51930687611702e-07,6.548915465748e-06,6.34208617666529

"SLC22A16",-2.6856416535,0.3332725612,-5.8417133317403,3.5315905958967e-07,6.56581017502101e-06,6.33869800804363

"CD38",-1.8957292795,-0.0296242356,-5.83862093996082,3.57119625902738e-07,6.63342420262493e-06,6.32785357113446

"LDHB",-0.8077215889,0.05705100988,-5.83668638530534,3.59619685672567e-07,6.6738117029525e-06,6.32106999619418

"AC010226.1",0.62363064315,-0.03158597908,5.83263011897212,3.64918217940671e-07,6.76601307617055e-06,6.30684793999671

"SOX13",0.92053759025,0.0563049922,5.83214128572904,3.65561970287183e-07,6.77182065754412e-06,6.30513412100126

"SMCR5",1.049307795825,-0.12405392734,5.83059442821333,3.67606488839224e-07,6.80354267690879e-06,6.29971111232762

"ITGB4",1.003937339575,-0.06436340334,5.83018709452913,3.68146756932106e-07,6.80739237908392e-06,6.29828311748844

"TMEM154",-1.619952851025,0.17130022743,-5.82980394132972,3.68655672555498e-07,6.81065592364026e-06,6.29693990960262

"LNX1",1.08451187955,-0.01928061076,5.81954392765807,3.82546113383694e-07,7.06090519910372e-06,6.2609779057275

"CEACAM6",-2.58874977255,0.33145631426,-5.81790546495442,3.84811933389656e-07,7.0963338355421e-06,6.25523610027248

"PRAG1",0.857865214825,-0.00849170664,5.81757802900946,3.85266337016568e-07,7.09832438201029e-06,6.25408867523334

"CEBPE",-3.61392175075,0.9073025924,-5.81730727392706,3.85642483261944e-07,7.09887079700873e-06,6.25313988470426

"SEMA6D",1.08892869695,0.03554859056,5.81670109906704,3.86485933194189e-07,7.10801059181558e-06,6.25101573355645

"TFCP2",0.6682322807625,-0.05441275629,5.8161145687502,3.87303791996049e-07,7.1137521507792e-06,6.24896046070221

"EIF4EBP1",-1.131092422475,0.05015936672,-5.81597937363814,3.87492551750761e-07,7.1137521507792e-06,6.24848672634719

"STIP1",-0.825553948175,0.06796297936,-5.81407310604474,3.90163828209021e-07,7.1563800468634e-06,6.2418072374846

"BTN3A2",1.00238832395,-0.05644078854,5.8128654159213,3.91865628831026e-07,7.17506268004031e-06,6.23757575353926

"TMEM42",0.77411260565,-0.11008499148,5.81285324537916,3.9188281623219e-07,7.17506268004031e-06,6.23753311145658

"AC027237.5",1.304657546475,-0.01000886382,5.81214440981773,3.92885135074365e-07,7.1869916494675e-06,6.23504958474352

"GLI2",0.94782485788125,-0.043001911695,5.81129790754181,3.94085453726431e-07,7.20251808737477e-06,6.23208379497336

"ALAS2",-3.53013095915,0.31458324298,-5.80964034489737,3.96446386349366e-07,7.2392099496665e-06,6.2262766284001

"SLC16A2",0.6813405375,-0.080518087,5.80890219248316,3.97502278848237e-07,7.25202732773169e-06,6.22369065988929

"RPS19",-0.645112097575,-0.02871212991,-5.8078681403191,3.98986137312501e-07,7.26883518087533e-06,6.22006817204128

"DAB2",0.934709598125,-0.0743653875,5.80776591342836,3.99133130539084e-07,7.26883518087533e-06,6.2197100578808

"GLIPR2",-1.1355713778625,-0.08347793079,-5.80624767632531,4.01322558428358e-07,7.30221720877459e-06,6.21439161727808

"DYRK2",0.71564947055,-0.04167767406,5.80540556377373,4.02542091693399e-07,7.31790805200919e-06,6.21144178109192

"ARHGAP5-AS1",0.649569900291667,0.0178661289333333,5.80376937515412,4.04922117501818e-07,7.35464924058266e-06,6.20571061097914

"C1QTNF8",2.1267253555,-0.0235818936,5.80274209424376,4.06423547766451e-07,7.37362548622144e-06,6.20211245515491

"SFXN3",0.69156159915,0.03433811232,5.80234288678086,4.07008502061598e-07,7.37362548622144e-06,6.2007142235441

"KLK4",0.9017410053,0.07986119224,5.80231691675807,4.07046584582021e-07,7.37362548622144e-06,6.20062326369557

"PAEP",1.0406596679125,-0.02877337417,5.80055776560872,4.09634458736086e-07,7.41394946164747e-06,6.19446203020477

"CCL28",0.70868256645,0.03724849716,5.79998314782582,4.10483307370053e-07,7.42275551756191e-06,6.19244957273184

"TTLL1",0.794890896575,-0.14621412974,5.79901175371414,4.11922261691341e-07,7.44212871952258e-06,6.18904759134448

"RAB26",1.110976538225,-0.02498915342,5.79877000730673,4.1228114489741e-07,7.44212871952258e-06,6.18820097292155

"MAB21L1",1.069655007325,-0.07103008164,5.79625159666346,4.16038339955104e-07,7.50333935651424e-06,6.17938167064295

"AUNIP",-1.5129600853,0.10117363796,-5.79537573272607,4.17352981404304e-07,7.51382063533513e-06,6.17631462883568

"RRP12",-0.967618106475,0.02050279642,-5.79504898377562,4.17844475378476e-07,7.51606462823021e-06,6.17517046421748

"ZFHX4-AS1",1.114383821375,-0.1467950709,5.79432434084123,4.18936529672793e-07,7.52909791222472e-06,6.17263305373519

"ABCB10",-1.4719153628,0.18088253696,-5.79014381242897,4.25292214491548e-07,7.63117885438864e-06,6.15799571524114

"AGTPBP1",-0.77132085865,0.06478740608,-5.79009858658655,4.25361492176485e-07,7.63117885438864e-06,6.1578373766537

"PPIAP76",-0.686585736425,-0.01489635514,-5.78953436831795,4.26226712823273e-07,7.64001127937289e-06,6.15586203241377

"SMOC1",0.990025967325,-0.00656571314000001,5.78827315055796,4.28167092384405e-07,7.66808338179344e-06,6.15144660883107

"ZNF251",0.67630400975,-0.0567950542,5.7878343093951,4.28844301670102e-07,7.67350397608477e-06,6.14991030466497

"PLA2G10",1.07032942905,-0.03518466906,5.78758756104344,4.29225544589189e-07,7.67362387220184e-06,6.14904649295043

"ALOX15B",-1.595162552275,0.09887395718,-5.78638856318464,4.31082870458342e-07,7.69635992045705e-06,6.1448491675441

"FAM171A1",0.6116088400625,0.0218160926,5.7862818296278,4.31248593746813e-07,7.69635992045705e-06,6.14447553418949

"ZNF787",-0.591928824175,0.04809218396,-5.78586292606594,4.31899629011501e-07,7.70127032131213e-06,6.14300912608272

"PODNL1",1.191217593925,0.02376575514,5.77417305955094,4.50465947636817e-07,8.01837214177507e-06,6.10209614526681

"H2AC8",-1.4790127419,0.14636934348,-5.77073957231809,4.56068083795911e-07,8.11104418473144e-06,6.09008249585233

"MRI1",0.834997557325,-0.01370661624,5.76829521882722,4.60098400762106e-07,8.1756253554328e-06,6.08153065295319

"SPAG8",0.599174567575,-0.01612352394,5.76357528262524,4.67981015703183e-07,8.30848791137505e-06,6.06501946841246

"SLC16A10",-1.629357732675,0.04954444986,-5.76327062512417,4.68494393023347e-07,8.31040097338731e-06,6.06395381424015

"LIPC",1.503640414925,-0.08015165696,5.76131382931072,4.71805127904168e-07,8.36188880666141e-06,6.05710945302887

"ZNF345",0.6535032840875,-0.04783936473,5.75785135523449,4.77720313617378e-07,8.45940690180886e-06,6.04499976053612

"TMEM97",-0.75659833245,-0.12935884396,-5.75584644181993,4.81179019431879e-07,8.51316556412e-06,6.0379884224045

"NYNRIN",0.628307258841667,0.00154701567333334,5.75561076547161,4.81587216361532e-07,8.51316556412e-06,6.03716427606315

"KCNJ12",1.177147851925,-0.21294041546,5.75515154091477,4.82383591073729e-07,8.51989225337807e-06,6.0355584142354

"FAR2",-1.28807901485,0.08298539952,-5.74849905260762,4.9406776751045e-07,8.71874282580027e-06,6.0122982279448

"HPRT1",-1.1098601391,0.01516984872,-5.7479193587186,4.95099119437155e-07,8.72942406112602e-06,6.01027160606235

"AC104115.1",0.97187729395,-0.14797137384,5.74606502541034,4.98412586167943e-07,8.77882708124958e-06,6.00378909575565

"MIRLET7BHG",0.869760928775,-0.0268343583133333,5.74587240160857,4.98758039953851e-07,8.77882708124958e-06,6.00311573202569

"PPIAP74",-0.750361994975,-0.01263490498,-5.74336869079167,5.03269889379291e-07,8.85063819193384e-06,5.99436381295761

"LINC01550",1.547460081075,0.10434990986,5.74222909631243,5.05336897313122e-07,8.87936736891187e-06,5.99038052654261

"DSG2",0.809568204375,-0.0602544525,5.74179352060255,5.061291713935e-07,8.88566792074552e-06,5.98885807751918

"CNIH4",-0.655929671775,0.00220066058,-5.74138886665247,5.06866305423179e-07,8.89099046704632e-06,5.98744372901077

"C3orf67",1.32540121985,-0.14243014412,5.73913809804521,5.10985872786126e-07,8.95558474049798e-06,5.97957720119244

"ZNF532",0.654915567375,-0.0018073145,5.7382731275458,5.12577841319983e-07,8.97580753244771e-06,5.9765542610908

"WDR4",-0.844708290425,0.07159946436,-5.73776244025795,5.1352006418598e-07,8.98462773274326e-06,5.97476953040929

"PHC2",-1.160400249325,0.08654362699,-5.73721086525667,5.14539655050603e-07,8.99478536917813e-06,5.9728419432549

"AC005099.1",-0.651074881675,-0.10195512774,-5.7360070625497,5.1677187251368e-07,9.02610581761319e-06,5.96863514955985

"ERO1A",-1.12429195275,-0.0127906246,-5.73537028362046,5.1795653637173e-07,9.03909158192845e-06,5.96640994283468

"DMAC1",0.92221367695,-0.09542341444,5.73271183045216,5.2293148928494e-07,9.11814498082541e-06,5.95712059557957

"RTP4",1.4424808082,-0.11099675844,5.73232132550057,5.23666250205689e-07,9.12319229099843e-06,5.9557561409188

"TESC",-1.828813546425,0.23726045906,-5.73001278550899,5.28030887055389e-07,9.19016200655316e-06,5.94769031174594

"RCL1",-0.781715108475,0.03679135912,-5.72981452730395,5.28407401587252e-07,9.19016200655316e-06,5.94699764663445

"HS3ST1",1.073140328,-0.2059293166,5.72837860336679,5.31142341556487e-07,9.22989337897312e-06,5.94198103054069

"NARF",-0.8969358999,0.02657550788,-5.72774809716167,5.32347669726428e-07,9.24299920114836e-06,5.93977834393808

"KIF6",0.961743792933333,-0.09777340732,5.72543828380168,5.36786519228101e-07,9.30762464753137e-06,5.93170938860623

"TSPAN11",1.0313889737625,0.03826659046,5.72520669022137,5.37233600379881e-07,9.30762464753137e-06,5.93090039124565

"ZBTB20",0.7394446894,-0.10991052748,5.72510364596255,5.37432641450098e-07,9.30762464753137e-06,5.93054044158058

"PIN4",1.01233057885,-0.05888132592,5.72397680278056,5.39614038810902e-07,9.33636440326275e-06,5.92660428973867

"CLEC18B",1.5968799334,0.01974041972,5.72377609165689,5.40003505362474e-07,9.33636440326275e-06,5.92590320675799

"PRDM6",0.6990990194,-0.02515172148,5.72258739931925,5.42315804674537e-07,9.36843693606401e-06,5.92175121414174

"SIGIRR",0.6344816450125,0.02846618061,5.72150569147451,5.4442852489374e-07,9.3970106301794e-06,5.91797306318118

"ZG16B",-1.230671099675,0.17318216526,-5.72046132980201,5.4647604700329e-07,9.42441182744394e-06,5.91432549362341

"SLC44A5",0.984834845525,-0.00942454337999999,5.71955556150369,5.48258032511523e-07,9.44719139621201e-06,5.91116209101947

"PPIAP70",-0.77696470865,-0.01572069192,-5.71790367493097,5.51522762702733e-07,9.48748812951604e-06,5.90539313170754

"MINPP1",-0.826099045375,0.0394911485,-5.71233316948002,5.62674886985464e-07,9.6659975443276e-06,5.88594154884281

"ZFAND5",-1.3596803962125,0.16041729183,-5.71076014851522,5.65864358282391e-07,9.70973950794776e-06,5.88044944929269

"CNTNAP2",2.54087188375,-0.291772193,5.70982120735618,5.67776701245206e-07,9.7262617517657e-06,5.87717134805416

"BCL9L",1.0582835247,0.04828997676,5.70809619932994,5.71306744871186e-07,9.77855688297482e-06,5.87114916481374

"RPS16",-0.598910857075,0.000166282440000001,-5.70565219685707,5.76345422586976e-07,9.84834446869222e-06,5.86261754786345

"GOLIM4",0.7078974744,0.00463212952,5.70520566669863,5.77270760798185e-07,9.85593612269435e-06,5.86105886596386

"HHIPL1",1.2325603442375,0.01401887589,5.70226380072683,5.83404079778281e-07,9.95235868983965e-06,5.85079047131337

"ZNF780A",0.669278027725,-0.00831239067,5.70101575147954,5.86025541926496e-07,9.98876148335279e-06,5.84643457112593

"ZNF366",0.83902265175,-0.0351722646,5.69971187489334,5.8877674163487e-07,1.00273132856319e-05,5.84188403821079

"BGN",1.32693428,-0.08946606,5.69903036703548,5.90219825924849e-07,1.00435413567677e-05,5.83950566100215

"PSMA7",-0.808052466675,-0.02522243494,-5.69661056365507,5.95372118822629e-07,1.01228082742224e-05,5.83106133361155

"HSPA4L",-1.249722640425,-0.02580707234,-5.69166388606789,6.06043843063952e-07,1.02957099972589e-05,5.81380139181093

"LETM1P2",-0.744247629225,0.04928860242,-5.68950724577391,6.10755636272008e-07,1.03671594663968e-05,5.80627743815581

"JAM2",0.640922662683333,-0.00964352312,5.6857646511725,6.19018752944442e-07,1.04900382219402e-05,5.79322194126685

"BOP1",-0.987934183575,0.12697280924,-5.68354347219528,6.23975107995372e-07,1.05652909195117e-05,5.78547454547953

"ARRB2",-0.66685254705,0.05244783336,-5.68312253454606,6.24918816019023e-07,1.05725323720873e-05,5.78400640213986

"EXOSC5",-0.834635331275,0.02386287498,-5.6790369031235,6.34152301962082e-07,1.07158364367737e-05,5.76975777180778

"GCLC",-1.145544668575,0.11359899314,-5.67891263340824,6.34435259557131e-07,1.07158364367737e-05,5.76932441577142

"ZNF683",1.41762071125,0.197972955,5.67610906626055,6.40852247156684e-07,1.07989982666602e-05,5.75954829540821

"MAPRE1",-0.621719489575,-0.02197562316,-5.67607085313421,6.40940154834969e-07,1.07989982666602e-05,5.75941505226781

"CRYGS",1.019207905225,-0.11311420442,5.67421550282602,6.45222720053507e-07,1.08572962395886e-05,5.75294597006861

"ARHGAP22",0.99053853105,0.06132511084,5.67411301316011,6.4546011420436e-07,1.08572962395886e-05,5.75258863079765

"SUV39H2",-0.80817115625,0.012118719,-5.67255667980167,6.49075666831536e-07,1.09091569007748e-05,5.74716250751383

"CASC15",0.8807320347,0.05172794576,5.6648892263489,6.6718328465995e-07,1.11951278756045e-05,5.72043477535235

"OXTR",1.613424991,0.0087395551,5.65958631547917,6.79998764171932e-07,1.13915083240838e-05,5.70195410099209

"H2BW2",0.76499936385,0.00806329708,5.65911342526137,6.81153371950024e-07,1.14015280102223e-05,5.70030625830809

"RHAG",-3.93340577225,0.6776855992,-5.65524633618504,6.90668453789032e-07,1.15513594132487e-05,5.68683204673384

"CDK2AP2",-0.723574218875,-0.0003630227,-5.65462547089412,6.92208335100146e-07,1.15638397689883e-05,5.68466893456524

"HSPE1",-0.931716717975,-0.03949379008,-5.65189885596356,6.99011318239275e-07,1.1662332156422e-05,5.67516994498098

"METAP2",-0.60810981555,-0.07022469994,-5.6490840826353,7.06103771284203e-07,1.17710773523765e-05,5.66536487960034

"MRGPRF",0.928954210375,-0.0282070847,5.64801385514596,7.08819123153751e-07,1.18067367440439e-05,5.6616370978927

"NR2E3",1.0990595232125,0.00448595807,5.64684143445867,7.11805635667479e-07,1.18468512295332e-05,5.65755353790768

"PEX2",1.218165967075,-0.09813572744,5.64511203728549,7.16233714879437e-07,1.19108736610795e-05,5.65153035961258

"SLC2A14",-1.75739552125,0.417678243,-5.64425335145009,7.1844249050377e-07,1.19379154464244e-05,5.64853986239499

"DMGDH",1.0694665311875,0.03316457245,5.64376328469347,7.19706100919549e-07,1.19492209040841e-05,5.64683317909787

"ADA",-0.730888719975,0.01518087302,-5.64013590968016,7.29127824696893e-07,1.20958468602348e-05,5.63420166917161

"LARP4",-0.796348838025,0.09155228588,-5.63946906190562,7.30873144035659e-07,1.21149910800992e-05,5.63187971802362

"NOC4L",-0.68247706965,0.08563465228,-5.6366955417779,7.38176715971061e-07,1.22162880103514e-05,5.62222302478932

"CNNM4",0.6612795306,-0.04772302542,5.63598602992967,7.40056677218316e-07,1.22375150951161e-05,5.61975285330322

"CCDC148",1.2032962916625,-0.09985691367,5.63500261851333,7.42670225201585e-07,1.22708286886533e-05,5.61632921197447

"PCDHGC3",1.1086507725,-0.023041383,5.63124683885498,7.52736151814146e-07,1.24271219003773e-05,5.60325508694147

"THY1",2.381845694875,0.2324995949,5.63078227548346,7.53990593381723e-07,1.24378094019362e-05,5.60163804464746

"ENPP1",1.0364211633875,-0.04057518944,5.62913414746724,7.58457699686259e-07,1.25008626761201e-05,5.59590151498141

"ESR1",0.649182494175,-0.00364982752666667,5.6289225003998,7.59033247222445e-07,1.25008626761201e-05,5.59516487616257

"HES5",1.421300053525,-0.00616675518000001,5.62508906106418,7.69533024623844e-07,1.26432980019994e-05,5.58182363687841

"PRMT1",-0.89217703205,0.05938840896,-5.62226105473836,7.77371048107834e-07,1.27618413731036e-05,5.57198283304974

"SNRPG",-0.913046947375,-0.00518512490000001,-5.62154457088575,7.79369361177271e-07,1.27844030999199e-05,5.56948981167807

"PCBP4",0.783299314675,-0.02112840326,5.62103320622778,7.80798697189588e-07,1.27940970243517e-05,5.56771055055822

"EEF1AKMT4",-1.242642367125,0.0467617427,-5.62088649216295,7.81209262859431e-07,1.27940970243517e-05,5.56720007490033

"HDAC9",0.680356525575,-0.06355452784,5.62006272212716,7.83518482007916e-07,1.28216666608452e-05,5.56433391192939

"XAF1",1.24628027285,0.13192477828,5.61904339829366,7.86385247611818e-07,1.28466322026957e-05,5.56078748422665

"CMC2",-0.787054040875,0.0385945503,-5.61885182321303,7.8692519593826e-07,1.28466322026957e-05,5.56012097301748

"UBE2J1",-0.687469868875,0.0895874795,-5.61649736029652,7.93591249079405e-07,1.29367504131634e-05,5.55192994877093

"RPL36A",-0.7081780457875,0.04696195057,-5.61645619950712,7.93708281401134e-07,1.29367504131634e-05,5.55178675990289

"SRGAP1",0.9926065891,0.08067003358,5.61432066390034,7.998037570884e-07,1.30128140835345e-05,5.54435804904558

"PPIAP51",-0.770509411175,-0.01403421394,-5.61415433802157,8.00280444418852e-07,1.30128140835345e-05,5.54377949166427

"NOD2",-0.78619832085,0.05276731232,-5.6120758639111,8.06261107326329e-07,1.30996650015082e-05,5.53654993484842

"GAS2",0.8955460773,-0.11304364516,5.60704827240573,8.2091136119027e-07,1.33165732130374e-05,5.51906496084046

"LRRC66",1.08787550725,-0.0425599472,5.60653375961018,8.22425430423808e-07,1.33305792868061e-05,5.51727578643392

"RGS2",-1.329306866575,0.13238409294,-5.60568701652635,8.24923186600544e-07,1.33537978227536e-05,5.51433139045123

"FGF9",0.870887496575,-0.00154780898999999,5.60560637338549,8.25161462495417e-07,1.33537978227536e-05,5.51405097376337

"SPATA17",0.80190286455,-0.14525564336,5.60533801493254,8.25954870968031e-07,1.33560879213836e-05,5.51311782982929

"RAPGEF4",0.99958872855,-0.00479354875999999,5.60370427925637,8.30801382325099e-07,1.34238633446976e-05,5.50743717443847

"FCN2",-1.7799582472,0.13570356224,-5.60346014990762,8.31528012495876e-07,1.34250164854338e-05,5.50658834557478

"AP005901.3",0.99561408575,0.0418540766,5.60194200177117,8.36060802241356e-07,1.34875698553708e-05,5.50130998819113

"VSIG10L",0.6685550046125,-0.06851910651,5.6015209107411,8.37322399320193e-07,1.3497294506115e-05,5.49984597982974

"ZNF260",0.62945696825,0.0394148343,5.60113107977312,8.38492021582307e-07,1.35055224356748e-05,5.49849067571125

"SCUBE2",0.91775790405,-0.02043039276,5.60035718163419,8.4081877056643e-07,1.35216208422883e-05,5.49580016970851

"EVC",0.592061466825,-0.03402460954,5.60014921647619,8.41445113858168e-07,1.35216208422883e-05,5.49507718005183

"RAB44",-2.64794667185,0.39951601802,-5.60014048342769,8.41471425903825e-07,1.35216208422883e-05,5.4950468197934

"G6PD",-0.899191703725,0.03426058902,-5.59934561379132,8.43869727090195e-07,1.35454053274996e-05,5.49228351743263

"DUS3L",-0.6287062713,-0.02282403844,-5.59921189613737,8.44273848263226e-07,1.35454053274996e-05,5.49181866720066

"RHOH",-0.934380942675,-0.02015666114,-5.59352276132794,8.61646282327504e-07,1.38133091019764e-05,5.47204355197357

"GRAMD1C",0.904866791875,-0.00699730610000001,5.59253956963909,8.64684251826657e-07,1.38511735351248e-05,5.4686264979199

"MGAT3",-1.23209949415,0.11465875208,-5.59025686702267,8.7177855968796e-07,1.39539055710054e-05,5.46069356183327

"METTL15",0.7110224542,-0.08981212084,5.58922376055299,8.75008225771642e-07,1.3994667080101e-05,5.45710351441437

"HDX",0.59814966095,-0.01399240594,5.58673922230733,8.82823916036246e-07,1.41086555317867e-05,5.44847036172392

"PJVK",0.849272213425,-0.08219326186,5.57894567069209,9.07791725250018e-07,1.44850754415283e-05,5.42139544208223

"PZP",1.0653906488,-0.10583700296,5.57741628397364,9.12772806330117e-07,1.45532212109661e-05,5.41608335315716

"STK36",0.67729340705,-0.05498557936,5.57264537205257,9.28485830583361e-07,1.47922377115022e-05,5.39951449476786

"RNF5",-0.731715422125,-0.0717475687,-5.57172425598573,9.3155023965084e-07,1.4829527047371e-05,5.3963159378666

"PTPN21",0.8185085117,0.13385219476,5.57143667372949,9.32509030950361e-07,1.48332647718253e-05,5.39531733947637

"S100A2",0.8808415458,0.07921209604,5.57116200254749,9.33425689505875e-07,1.48363270183059e-05,5.3943635845896

"PCDHGA3",0.996856546325,-0.11657134894,5.57049651030935,9.35650334767746e-07,1.48486476055163e-05,5.39205280697813

"UBQLNL",1.2053775415,-0.0931604128,5.56718450352584,9.46800229200557e-07,1.50023535157471e-05,5.3805535370437

"PIWIL4",-1.0162263202,0.06449392354,-5.56651428782183,9.49072471000355e-07,1.50267363105528e-05,5.37822674564384

"PPIAP81",-0.75496504325,0.0376482194,-5.56608926970316,9.50516213153101e-07,1.50379738803712e-05,5.37675124186875

"SUSD2",1.32994457855,-0.01835800716,5.56092972250535,9.68217069828497e-07,1.53061970113011e-05,5.35884132636183

"DPY19L4",0.6188774968,-0.04428863586,5.55959336792877,9.72854672823262e-07,1.53405456173552e-05,5.35420318180725

"GNG5",-0.733987128475,0.00953682022,-5.5595339630597,9.7306133767356e-07,1.53405456173552e-05,5.35399700878618

"ANGPTL5",1.4486099044,-0.06422399048,5.55840831248705,9.76985613656752e-07,1.5370844177957e-05,5.35009037657312

"SHISA2",1.3956502185,-0.1237153402,5.55803455170057,9.78292091843838e-07,1.5370844177957e-05,5.34879326027637

"LETM1",-0.730580364675,0.10089670626,-5.55802991294154,9.78308317456847e-07,1.5370844177957e-05,5.34877716184644

"ZNF519",0.848668413525,-0.06255624538,5.55557446434776,9.86934580394501e-07,1.54944947763391e-05,5.34025617358042

"ABCB1",1.0984573766625,0.17252603333,5.55531799581593,9.87839915296207e-07,1.549683321944e-05,5.33936621809421

"SYCP2",0.89642019205,-0.06195922536,5.54896359739454,1.01053542400857e-06,1.5840741979409e-05,5.31731930851262

"TMEM182",0.7995797844,-0.09223700588,5.54232624132974,1.03479286563138e-06,1.62085903907153e-05,5.29429703618488

"BTG3",-0.911975004425,0.00285436976,-5.54084791745402,1.04027368071813e-06,1.62819917269313e-05,5.28917022602904

"ANKRD65",1.00463801805,-0.14624586096,5.53949020466163,1.04533264911652e-06,1.63486834466407e-05,5.28446198029528

"SNRNP70",-0.7759629702,0.07902315024,-5.53807831619083,1.05061931526978e-06,1.6418831831615e-05,5.27956615675681

"ARHGAP28",0.9867346396,0.10967064768,5.5361101386253,1.05803314021852e-06,1.65220906835344e-05,5.27274185768679

"MYOZ3",0.889752657225,0.03584754978,5.53555650727263,1.0601279024333e-06,1.65421938043058e-05,5.27082234558919

"CDC7",-1.10751292515,0.07841024988,-5.53032680963666,1.08011906126304e-06,1.6828501389473e-05,5.25269256639637

"AC013701.1",0.77724917925,-0.0779658116,5.52644356187024,1.09520425242334e-06,1.70505658994296e-05,5.23923317842244

"KPNB1",-0.628658578925,-0.000427093539999998,-5.52573342342076,1.09798538576503e-06,1.70728573322804e-05,5.23677207479503

"LRRC3",0.795504605525,-0.0318813226133333,5.52558444138135,1.09856973447933e-06,1.70728573322804e-05,5.2362557622579

"FAM210B",-1.078472687875,0.19854763945,-5.52544013890873,1.09913602212406e-06,1.70728573322804e-05,5.23575567043125

"STOML2",-0.95126953185,-0.05411712748,-5.5252213123958,1.09999531657727e-06,1.70732606409357e-05,5.23499731554197

"SNHG8",-0.801041963425,0.06462182976,-5.52487921707352,1.10133999921155e-06,1.7081191448786e-05,5.23381178053152

"ZNF859P",0.87094032315,-0.05154175948,5.5246239449582,1.10234446366631e-06,1.70838376487105e-05,5.23292714378389

"RBBP8",-0.821677670875,-0.0106612227,-5.52391983081863,1.10511976906894e-06,1.71009772119974e-05,5.23048711141336

"UBAP1",-0.66902944185,-0.01786553148,-5.52289546145366,1.10916973305665e-06,1.71506939553695e-05,5.22693740249455

"SATB2",1.1598863527,0.18700132256,5.5210217550359,1.11661569898029e-06,1.72528072705191e-05,5.2204449281788

"AC034207.1",-0.6873434811,-0.04144658888,-5.52069755432278,1.11790905942497e-06,1.7259774536171e-05,5.21932161252094

"GPR20",1.241295927125,-0.0239062893,5.52032740299312,1.11938754747664e-06,1.72695874041427e-05,5.21803910308547

"AL590617.2",1.1182827372,-0.10574506424,5.51909530978737,1.1243228298561e-06,1.73326757999186e-05,5.21377026582555

"ARMCX2",0.857500333425,-0.01444080326,5.5187975188341,1.12551889182207e-06,1.73380684628951e-05,5.21273854310291

"SPCS3",-0.907693780425,-0.05183967634,-5.51395798823588,1.14513409327381e-06,1.76237608673953e-05,5.19597345589906

"ADIPOR2",-1.03185742065,0.02922798148,-5.51379872803094,1.14578531215202e-06,1.76237608673953e-05,5.1954218077444

"SLC8A3",2.4070208179,-0.07779684368,5.51247150414399,1.15122663793082e-06,1.76941720614604e-05,5.19082469786362

"SLC22A15",-1.1720367834375,0.0611007054,-5.51198345854251,1.15323394232695e-06,1.77117368893513e-05,5.18913432021502

"MYH6",1.115124583875,0.0678381448,5.51090531378247,1.15768057866879e-06,1.77667113825964e-05,5.18540022466246

"ZNF132",0.624630498675,0.04340080194,5.5078595688008,1.17033415532209e-06,1.79474597112567e-05,5.17485241294155

"GATA1",-2.7105168676,0.45173376762,-5.50681766675956,1.17469409160505e-06,1.80008470821274e-05,5.17124449635927

"NCKAP5",1.3262898661,0.01540508958,5.504948930607,1.18255430103718e-06,1.81077522568384e-05,5.1647738211566

"LINC01600",1.137533161775,-0.07331144358,5.50311355172478,1.19032486460432e-06,1.82131260836544e-05,5.15841917053321

"AC023824.6",0.65424355635,-0.07033559692,5.50264490422692,1.19231709343527e-06,1.82299944852999e-05,5.1567966506112

"SLC25A53",0.71126374645,-0.12418791684,5.50114802902344,1.19870246448271e-06,1.83076955519768e-05,5.15161449608457

"DYNLRB2",0.6479126878375,-0.01787018273,5.50092967950238,1.19963672391099e-06,1.83076955519768e-05,5.15085860287662

"MYL3",0.864528708475,-0.08999458422,5.50082618313726,1.20007980897622e-06,1.83076955519768e-05,5.15050031658942

"FKBP4",-0.6288259765625,0.06310263545,-5.49949793857621,1.20578064072085e-06,1.83809775052743e-05,5.14590231313042

"LINC00900",0.775020796475,-0.15309789682,5.49713311295958,1.2159968928338e-06,1.85229325950773e-05,5.13771663574962

"TMEM200B",1.1329335105,0.0653590764,5.49412853293827,1.22910057242787e-06,1.86831030675503e-05,5.12731774048113

"ZNF382",1.16949127715,-0.02790046653,5.49409502178396,1.22924750756823e-06,1.86831030675503e-05,5.12720176576858

"AL139095.2",-0.779644758875,-0.0356942365,-5.49045751624079,1.24530052322085e-06,1.88710925441929e-05,5.11461419715423

"MAP3K12",0.84580257075,0.0964581256,5.48581936378412,1.26607078642888e-06,1.91441001376112e-05,5.09856687912902

"NHSL1",0.86369340225,-0.000505314199999999,5.48580858123338,1.2661194692729e-06,1.91441001376112e-05,5.09852957701231

"ATG3",-0.758763190675,-0.04145593754,-5.48552537562283,1.26739879624504e-06,1.91493116058027e-05,5.09754983678677

"SCARA5",1.3440781453125,0.0750901226,5.48069240243415,1.28942902639071e-06,1.9410596541288e-05,5.08083226175971

"RPA3",-0.89172298545,-0.05087867936,-5.47998022589493,1.29270722395024e-06,1.94456575655599e-05,5.0783691049408

"NBPF12",1.08891176055,0.12050046804,5.479048064993,1.2970104877218e-06,1.94960754750141e-05,5.07514522327763

"CTPS1",-1.20988426205,-0.00784318964,-5.47788840653282,1.30238376842359e-06,1.95474809741127e-05,5.07113473089439

"DLX3",1.225448827625,-0.0626508409,5.47751785858411,1.30410534201032e-06,1.95474809741127e-05,5.06985329490164

"DKC1",-0.807202123975,0.03737085452,-5.47748745341531,1.30424670497911e-06,1.95474809741127e-05,5.069748148124

"CD5L",-2.48187074665,0.24060084668,-5.47622174563321,1.31014484015079e-06,1.96215259393344e-05,5.06537122211224

"DCLK2",0.7341867854125,0.02858700163,5.47494656135304,1.31611382085507e-06,1.9689912305003e-05,5.06096178041154

"SIX4",1.03736566025,0.0438423992,5.47483588632397,1.31663314417484e-06,1.9689912305003e-05,5.06057909086585

"PNO1",-0.88788051255,-0.00566601704,-5.47456898296677,1.31788637589616e-06,1.96942786793293e-05,5.05965620657101

"SNRPF",-0.77699420985,-0.02815664288,-5.47394673748614,1.32081268409041e-06,1.97198284474648e-05,5.05750468268126

"TPCN1",0.72858525545,-0.01709412744,5.4737963044528,1.32152110788604e-06,1.97198284474648e-05,5.05698454300005

"PIP5K1B",-0.9240618988125,-0.0102599673,-5.47247210961449,1.32777328686835e-06,1.97987038583398e-05,5.05240613953405

"SDF2L1",-1.0256324812,0.04384071484,-5.47165558275359,1.3316430905835e-06,1.98419662835453e-05,5.04958313592881

"C1QBP",-0.90264712435,-0.03615167348,-5.47133471250659,1.33316685797867e-06,1.9850234437694e-05,5.0484738100982

"FDFT1",-0.6231328813,0.03191448396,-5.47071226133524,1.33612771101736e-06,1.98654459675789e-05,5.0463218926557

"PXYLP1",0.73293703605,0.00971377089,5.46953950307341,1.34172393096019e-06,1.99341841171229e-05,5.04226763827281

"AC133561.1",-0.9627272732,0.15459258594,-5.46894227276956,1.3445827259101e-06,1.99621818032218e-05,5.04020308190769

"NR3C1",0.6416486065125,0.00653010216,5.46687157849724,1.35454137677588e-06,2.00893165345831e-05,5.03304536770345

"ABCF1",-0.664160845825,-0.02473421036,-5.46675438864999,1.35510715788724e-06,2.00893165345831e-05,5.03264030096821

"GSTM2P1",0.944907069425,0.02445783454,5.46503988072856,1.36341141415262e-06,2.01978113182639e-05,5.02671435301862

"PPIAP19",-0.712230733625,-0.0367528329,-5.46455927838018,1.36574824233749e-06,2.02178106857013e-05,5.02505330444245

"TMEM121",0.8895899259,-0.05734985328,5.4639444147208,1.36874366590646e-06,2.02375549052504e-05,5.02292827801178

"FAM225A",1.192096756,-0.0576557752,5.46294189801031,1.37364155684305e-06,2.02906764359051e-05,5.01946361517476

"PTGER2",-0.968916253275,0.11245239148,-5.4626466269195,1.37508743434129e-06,2.02974001115162e-05,5.01844319904008

"THNSL1",0.76816295645,-0.10893488984,5.4616154340085,1.38014877891979e-06,2.03574428959745e-05,5.01487964751184

"CTSG",-4.35162602525,0.6268530238,-5.46068977507202,1.3847078308745e-06,2.03953228173665e-05,5.01168093991756

"TAF4B",-0.7908005583,0.01308797881,-5.45824158021053,1.3968376003483e-06,2.05592016924827e-05,5.00322161469958

"ROR1",1.0235241566625,0.02075756888,5.45682757816293,1.40389120398302e-06,2.06481859204625e-05,4.99833620458858

"HSPA12B",0.8882156014,0.04283243312,5.45435669465479,1.41630162761976e-06,2.08157731324775e-05,4.98980001192746

"TDRP",0.626749341275,-0.07601718998,5.4516244804619,1.43015099525684e-06,2.10042534701234e-05,4.98036213568984

"MYO1B",1.0923589805875,0.14363642752,5.45060683754078,1.43534350905202e-06,2.10654139064884e-05,4.97684720217006

"FAM171B",0.9704497226125,0.07050202109,5.44936107295062,1.44172540344276e-06,2.11439298967324e-05,4.97254456414505

"PGF",1.1721889483,0.06193612679,5.447683119304,1.45036572828044e-06,2.12402380564758e-05,4.96674962057352

"H1-9",1.155513316725,-0.11624429822,5.44596821804564,1.4592492276281e-06,2.13491066694249e-05,4.96082754422595

"PRAM1",-2.35824771865,0.39809173808,-5.44412416207151,1.46886182548235e-06,2.1465079230016e-05,4.95445998672101

"ZSWIM3",0.961769512075,-0.18284049034,5.44369686268075,1.4710981512566e-06,2.14824368659623e-05,4.95298459283489

"CCN3",1.13403219515,-0.02124052088,5.44255893345924,1.47707008685266e-06,2.15542820081462e-05,4.94905565604292

"PTGIS",0.894756008475,-0.09870601622,5.44153585519364,1.48245974187762e-06,2.16175339441912e-05,4.94552344484792

"CRIP2",0.7913058089,-0.05295455688,5.44026674835917,1.48917252966381e-06,2.16845535094187e-05,4.94114204749842

"AHCY",-1.155820812,-0.0517647446,-5.43943443358365,1.49359127222993e-06,2.17334502737549e-05,4.93826874969844

"EPX",-3.432929812725,0.43636803882,-5.43804354281475,1.50100444247347e-06,2.18258190329286e-05,4.93346739960593

"LRRC3B",1.4727967415,-0.00278277730000001,5.43746390623693,1.50410452113567e-06,2.18553854106579e-05,4.93146658887489

"GK",-0.8794508876125,-0.03989596994,-5.43600909842067,1.51191320906881e-06,2.19377321723808e-05,4.92644507054971

"EIF3J",-0.73192594985,0.01178238142,-5.43532555469286,1.5155959641558e-06,2.19756051759547e-05,4.92408582116183

"TMEM54",0.77090294685,-0.04770979052,5.43268551089701,1.5299033184155e-06,2.21517026061461e-05,4.91497443131376

"SLC25A21",-1.558122356725,0.18614351962,-5.43113454802734,1.53837074595874e-06,2.22585733355951e-05,4.90962223666242

"VSIR",-0.81254802925,-0.067323113,-5.42966647064529,1.54642835177527e-06,2.23593677284204e-05,4.90455643221152

"SHC4",1.329046896575,-0.11634517674,5.42781247997898,1.55666371583751e-06,2.24914853385605e-05,4.89815948407961

"COLEC12",1.22914465405,-0.07120773276,5.42603756273445,1.56652529376536e-06,2.26180198863035e-05,4.89203589564007

"AC092835.1",0.909984308275,0.01162339062,5.42287940426272,1.58422525711382e-06,2.28413842841294e-05,4.8811413063348

"TEX21P",1.018588433725,-0.06278671102,5.42179952902823,1.59032269484036e-06,2.29131725540712e-05,4.87741647436689

"DPH3",-0.8282110996,0.06050698192,-5.42068827233502,1.59662155246778e-06,2.29877599205621e-05,4.87358359815305

"VANGL2",0.7653455086,-0.04711231952,5.42032034395796,1.5987124858658e-06,2.30017004286647e-05,4.87231460770712

"SMAP2",-0.804661016225,-0.04389132498,-5.41910994188091,1.6056103129183e-06,2.30847326954878e-05,4.86814007035664

"SLC23A3",1.0717211875,0.038005188,5.41817748200947,1.61094426575546e-06,2.31451796050477e-05,4.86492428842018

"MXRA5Y",1.25893256595,-0.000915981239999998,5.41561270521427,1.62570610653029e-06,2.3340901689273e-05,4.85607986239947

"VNN2",-1.75415728325,0.3300010955,-5.41476741096618,1.63060052875438e-06,2.3391449171123e-05,4.85316517159625

"NSDHL",-0.611551167375,0.0566319471,-5.41441519716941,1.63264421439951e-06,2.3391449171123e-05,4.85195072489219

"ADD3-AS1",0.6503383637,-0.11986724349,5.41441387186082,1.63265190915199e-06,2.3391449171123e-05,4.85194615521601

"PVT1",-0.9151013981625,-0.05532285078,-5.41180685966751,1.64785786237408e-06,2.35545721902899e-05,4.84295771658967

"ANKRD36B",1.0327505818,0.01817958944,5.41170275128583,1.64846799545553e-06,2.35545721902899e-05,4.84259879582886

"CA8",-1.079772152375,-0.0001494199,-5.40911758145885,1.66369036974364e-06,2.37530928887161e-05,4.83368682333438

"CLLU1",0.98162394345,-0.09087606324,5.40857033165564,1.6669305457966e-06,2.37827806561844e-05,4.83180040685057

"SRM",-0.788417312875,0.0332668477,-5.4063731047928,1.68000289642414e-06,2.39526091453986e-05,4.82422688128596

"LINC00922",1.91302190065,0.15324545052,5.40603507887691,1.68202294921111e-06,2.39529634665367e-05,4.82306182584966

"BST1",-1.450551579,0.2939678674,-5.40594677344576,1.68255106054356e-06,2.39529634665367e-05,4.82275747148738

"UBQLN1",-0.6552057274125,0.05031942902,-5.40578230325874,1.68353511283741e-06,2.39529634665367e-05,4.82219061039819

"XK",-2.7435043239,0.43251065018,-5.40497657170547,1.68836416545425e-06,2.40050000151469e-05,4.81941365030271

"MRPL20",-0.58773438485,-0.05708402628,-5.40285289249117,1.70115788673981e-06,2.41701267569523e-05,4.81209489241833

"AC107956.2",-0.652053073875,-0.0022040001,-5.40237596143704,1.70404422428409e-06,2.41943576348805e-05,4.81045136599037

"SHF",0.614897337425,0.02873670794,5.40213281971044,1.70551755563849e-06,2.41985067035467e-05,4.8096135030112

"PCMT1",-0.73662084235,0.02424566012,-5.40187874267148,1.70705849573027e-06,2.42036086231985e-05,4.80873796766142

"RCAN2",0.89684728725,0.1117561748,5.40110149484262,1.71178094064797e-06,2.42537814052529e-05,4.8060596819004

"MEDAG",-1.5818320936,0.25092378232,-5.4004708210496,1.71562231270102e-06,2.42914097737516e-05,4.80388654377227

"SLC28A3",-1.8613877365,-0.0754808432,-5.3988118809388,1.7257674917366e-06,2.44013280681155e-05,4.79817058500148

"GAREM2",0.664718374525,-0.01094342038,5.39833490537232,1.72869537749356e-06,2.44258695821297e-05,4.7965272278875

"ZBTB7B",-0.67591376565,0.07418526678,-5.39687046206295,1.73771549218014e-06,2.45351329523785e-05,4.79148191793605

"LY6G6F",-1.767327591175,0.10149505006,-5.3966911143452,1.7388233623025e-06,2.45351329523785e-05,4.79086405271599

"GOLGA8IP",0.848202927633333,-0.00279004679333333,5.39511142934322,1.74861163029643e-06,2.46562664015921e-05,4.78542216269447

"TBX18",0.6257452877,-0.00631657584,5.39418813219062,1.75435789598418e-06,2.47202782482282e-05,4.78224167123463

"PABPC4L",0.660020174375,-0.0489436635,5.39340314287071,1.75925806284802e-06,2.47722881042132e-05,4.77953772306186

"NCALD",0.75539790945,-0.03883362844,5.39099338892456,1.77438522792233e-06,2.49681349929071e-05,4.77123781304349

"DLX1",1.0493972286625,0.01978268773,5.38943342950063,1.78424631441422e-06,2.50896626559495e-05,4.7658653706611

"IPP",0.6262029634875,0.02592725384,5.38918780004465,1.78580395859389e-06,2.50943425951109e-05,4.76501946937635

"ELOC",-0.66156224815,-0.01555875812,-5.38838213551304,1.79092247179301e-06,2.51490196039035e-05,4.76224498502008

"AC025171.2",0.913739048025,-0.11332399298,5.38803264528188,1.79314734529035e-06,2.51630156234991e-05,4.76104147208498

"TTLL7",0.7767996654625,-0.03210102413,5.38732381480583,1.79766819117366e-06,2.51965339859183e-05,4.7586005899143

"UTP4",-0.774715262975,0.13086475592,-5.38727255721713,1.79799554311854e-06,2.51965339859183e-05,4.75842408593716

"AL121895.1",1.1305571137,-0.14141673844,5.38445980644953,1.81604953974947e-06,2.54321414698477e-05,4.74873914474153

"DYNC2H1",0.727169210925,-0.01438765406,5.38391681384221,1.81955538441863e-06,2.54638324562629e-05,4.74686965202093

"LRRC15",2.04720170535,0.09305758828,5.38326717040628,1.82375861305014e-06,2.55052330813455e-05,4.74463303228915

"AC105760.1",1.140873562225,0.05904675378,5.37794040556486,1.85858698487923e-06,2.59568712653072e-05,4.72629652810423

"EXD2",0.643612321875,0.0515536673,5.37611884189088,1.87064723799675e-06,2.6104475299895e-05,4.72002720638654

"DNM3",0.6939360136375,0.01926995786,5.37577280328928,1.87294702542672e-06,2.6104475299895e-05,4.71883630060017

"KLHDC9",1.1100669868,-0.09722639956,5.37576810572178,1.87297826487923e-06,2.6104475299895e-05,4.71882013387309

"DEPP1",-1.9918814539,0.22665266688,-5.37554723722568,1.87444765139989e-06,2.61071947531482e-05,4.71806001691645

"TMEM175",0.6084255388,-0.03228956356,5.37534116587855,1.87581962328744e-06,2.61085546480388e-05,4.71735083173037

"GPRASP2",0.69635196645,-0.13735316204,5.37081235422015,1.9062231574746e-06,2.65137135440188e-05,4.7017669659431

"AP000894.1",-0.745357506125,0.0997759801,-5.36874072603528,1.92029258680407e-06,2.6691285290666e-05,4.69463956204675

"PLEKHH3",0.8248998593,0.01543975894,5.36682442827945,1.93339843794077e-06,2.68552319976478e-05,4.68804722627414

"AC079250.1",-0.66658503635,-0.01080947908,-5.36656138705792,1.93520430254452e-06,2.68621041670272e-05,4.68714237648835

"CPED1",0.7483557319,0.0509667748533333,5.36605676747558,1.93867334959427e-06,2.6892037634724e-05,4.68540654150366

"AHSP",-3.894490696,0.7090733322,-5.36481322259314,1.94724845082946e-06,2.6992710595801e-05,4.68112907316725

"LRP1",0.63388732685,0.08167193258,5.36417403662512,1.95167065317371e-06,2.7035718960259e-05,4.67893054468441

"AC106795.1",-0.602146112175,-0.06812302734,-5.36180363261436,1.96815708864188e-06,2.72324663706352e-05,4.6707779759476

"MNDA",-1.8291326732,0.23229160144,-5.36167556375861,1.96905173047701e-06,2.72324663706352e-05,4.67033753428082

"AOX2P",-1.62147900655,0.19187092076,-5.36088577957854,1.97457775898738e-06,2.72659528684557e-05,4.66762144972006

"SAMD9L",1.3410809811375,-0.14029844664,5.3608333643942,1.97494504377139e-06,2.72659528684557e-05,4.66744119660236

"SLC25A39",-1.274528558175,0.17403608496,-5.36003911483181,1.98051879512547e-06,2.73244909592799e-05,4.66470987141319

"OAS3",1.2132718469875,0.07835637759,5.35936969990333,1.98522857228104e-06,2.73710383505342e-05,4.66240792168318

"MRC2",1.2558663289,-0.12043894088,5.35619288333634,2.00773089970833e-06,2.76440797535109e-05,4.65148470560981

"ZNF891",0.644882735091667,-0.0963671556933333,5.35455717649042,2.01941519730562e-06,2.77495670197411e-05,4.64586114794334

"B4GALT5",-0.72675163575,-0.0225317586,-5.35455154966939,2.01945550695207e-06,2.77495670197411e-05,4.64584180374285

"ZNF827",1.1380329428,-0.04604276896,5.35390394648769,2.02410014800374e-06,2.77947478768771e-05,4.64361547400101

"OLMALINC",0.591502209725,-0.04368719122,5.35294990355591,2.03096182483372e-06,2.78482896812891e-05,4.64033579869695

"PPIAP33",-0.7302054134,-0.03789291372,-5.35279515468355,2.03207697547477e-06,2.78482896812891e-05,4.63980383959296

"PTPN13",0.79305773305,0.00714963764,5.35243102024454,2.03470338676385e-06,2.78656437085681e-05,4.63855212070175

"RPL7AP8",-0.605210892675,-0.02868251114,-5.35032297247594,2.04997425840591e-06,2.80560271250636e-05,4.6313061209498

"AL133346.1",0.8154345008,-0.04565835736,5.35011850925666,2.05146141751074e-06,2.80576378651268e-05,4.63060336001223

"NNMT",-1.59159192085,0.16010924932,-5.34867578721758,2.06198536801227e-06,2.81639708132236e-05,4.62564478440069

"LCP1",-1.067880401525,0.07241876278,-5.3457584488969,2.08342917580338e-06,2.83623235573818e-05,4.61561912423566

"CDNF",0.91137927735,-0.05903925742,5.34541224696728,2.08598850254296e-06,2.83783083931608e-05,4.61442947310321

"LINC01711",2.224553649025,0.46840938622,5.34492269398473,2.08961286650759e-06,2.84087514326527e-05,4.61274726159848

"SALL4",1.274853503875,-0.0411967914,5.34354846862524,2.09982010586076e-06,2.85285904037634e-05,4.60802534580568

"TIGD6",0.78615114635,-0.11458704252,5.34264718134833,2.10654126772306e-06,2.86009393592511e-05,4.60492865156458

"TBC1D22B",-0.817025517875,-0.0379084383,-5.34212402149021,2.11045236798747e-06,2.86350649770379e-05,4.60313121440278

"NOP56",-0.87946460665,-0.06212985832,-5.34030810901423,2.124083731256e-06,2.87478038436484e-05,4.59689259810889

"AC093270.2",-0.673553136025,-0.01318961982,-5.3402695626722,2.12437402476004e-06,2.87478038436484e-05,4.59676017738625

"DANCR",-0.930239051775,-0.04081675642,-5.33911724484902,2.13307028328184e-06,2.88464316593256e-05,4.5928016665727

"GULP1",0.82723757725,0.0094440693,5.33891581081676,2.13459406340635e-06,2.88479968146895e-05,4.59210971235449

"RNF148",0.96070576045,-0.02909301764,5.33694802884125,2.14953634656294e-06,2.90116605193555e-05,4.58535048011383

"OSGEPL1",0.8079709656,-0.15277506852,5.33278539949737,2.18148645381853e-06,2.93562904992426e-05,4.57105430582343

"KAZALD1",2.17855815935,-0.23842130602,5.33272570576916,2.18194802615045e-06,2.93562904992426e-05,4.57084931535189

"MXD4",0.7223837690625,-0.00224363255,5.33272278172678,2.18197063832511e-06,2.93562904992426e-05,4.57083927409836

"RPGR",-0.920129460308333,0.0642398117533333,-5.33268860018905,2.18223498781464e-06,2.93562904992426e-05,4.57072189373414

"CCT3",-0.9014445695875,0.07572954803,-5.33185028318674,2.18872818554497e-06,2.94108282941451e-05,4.56784315415081

"INTU",0.9004315978625,-0.07609072341,5.33179460078843,2.18916014977408e-06,2.94108282941451e-05,4.56765194791862

"CUL7",0.78631267555,-0.11852995656,5.33055629860519,2.19878824838393e-06,2.95208215156552e-05,4.56339991835353

"SAXO2",1.368743924125,-0.1801991627,5.32890520922725,2.2116909652112e-06,2.96551861879889e-05,4.55773089985399

"PAG1",-0.7739690560625,0.0829792177,-5.32767703326677,2.22133723650478e-06,2.97650472867953e-05,4.55351426869617

"NAPEPLD",0.785794308175,-0.07522521846,5.32724946435726,2.22470516747559e-06,2.97906924648627e-05,4.552046381896

"RMND5A",-0.6764616008,0.01528857851,-5.32513118432221,2.24146525474476e-06,2.99759400386493e-05,4.54477459352455

"GPX8",1.3155064678,0.07318597724,5.32210346807757,2.26563765371026e-06,3.02597029004014e-05,4.53438221031269

"BCL2L1",-0.8447524751,0.05552450092,-5.32187982312495,2.26743334741118e-06,3.02639572780198e-05,4.53361463241618

"RPS6KA6",1.034114283725,-0.00116464802,5.32102516303306,2.27430855853787e-06,3.0335959470914e-05,4.53068141250298

"TP63",1.73560025075,-0.0797432784,5.31897374962803,2.29089504913972e-06,3.05373180005039e-05,4.52364142983073

"QRICH2",0.942402626025,0.04892250932,5.31510195301929,2.32252626851387e-06,3.0898648174878e-05,4.51035635635188

"GMPR",-1.599423798325,0.31889283234,-5.31363437463686,2.33462827794214e-06,3.10394965337304e-05,4.50532144030419

"WDR66",0.6626648292,-0.10817012774,5.3105601064306,2.36018168812191e-06,3.13588861389375e-05,4.49477560217331

"HEMGN",-3.157686068175,0.34419081186,-5.30634789781267,2.39564274413518e-06,3.18094157756589e-05,4.48032898149452

"PDE4B",-1.3228141358,0.07053612066,-5.30551844235149,2.40268738424138e-06,3.18822921815656e-05,4.47748457314865

"PDE5A",0.921299094925,-0.05531006506,5.30469007400259,2.40974320841186e-06,3.19424870677672e-05,4.47464401669887

"STK38L",0.75723168245,-0.06311233654,5.30461989574098,2.41034190778837e-06,3.19424870677672e-05,4.47440337423788

"TMEM38B",-0.7870112294375,0.03517558985,-5.30224698571412,2.43067217938288e-06,3.2191087014348e-05,4.46626714890093

"TJP1",0.623665717975,-0.01619184732,5.30156685166807,2.43653049290349e-06,3.22478273505212e-05,4.46393529550067

"CENPN",-1.0037829790125,0.02211487284,-5.3004083723858,2.44654113653164e-06,3.23594156263785e-05,4.45996361854571

"CD300A",-0.806667940575,-0.01592484811,-5.29828045122624,2.46503484647498e-06,3.25619819049512e-05,4.45266898543627

"VASP",-0.711470341975,-0.08980691798,-5.2974553251309,2.47224308088193e-06,3.26361573718485e-05,4.44984062791723

"TTF2",-0.815066450875,-0.0548861507,-5.29670923308999,2.47877879724031e-06,3.26866911078369e-05,4.44728328883631

"HMGN2P20",-0.7795805502,0.04364446524,-5.29665416010722,2.47926190851125e-06,3.26866911078369e-05,4.44709452217852

"RPL7AP60",-0.7366969987,0.00302129704,-5.29390476042659,2.503498745567e-06,3.29850046940043e-05,4.43767145562767

"NOX5",2.4361746648,0.30115011484,5.29357839470602,2.50639125298891e-06,3.30018920252165e-05,4.43655298791015

"GABRB2",1.8685331197,-0.21478806924,5.29319418138759,2.50980068031749e-06,3.30255596264257e-05,4.43523629914114

"FAM66B",0.82539796535,-0.0697280454533333,5.29088840711785,2.53035805025234e-06,3.3274695592792e-05,4.4273350376607

"TENM4",0.89428603605,-0.11618189916,5.28735258568117,2.56220541762607e-06,3.36718823581289e-05,4.41522062444464

"FARSB",-0.6919132195,0.0043727004,-5.28681210262101,2.56710832991779e-06,3.37146893995869e-05,4.41336902560646

"ZNF391",0.712482870025,0.00419760002,5.28653657470797,2.56961130435928e-06,3.37259426032754e-05,4.41242513655949

"DHX58",0.71581091575,-0.0078167694,5.28592530598411,2.57517285041785e-06,3.37556886496231e-05,4.4103311339203

"CCT6P3",-0.73307322705,-0.000238610040000003,-5.28342130349001,2.59807950552635e-06,3.4012429973945e-05,4.40175397197943

"SEMA5A",1.11441307145,0.00544895316,5.28255921061173,2.60601240625282e-06,3.40944969216524e-05,4.39880124184679

"THEMIS2",-0.875243921025,-0.00145731982,-5.28161495193199,2.61472883316744e-06,3.4186703467731e-05,4.39556724458068

"AC016582.3",0.8096378897,0.04316834296,5.28120287982251,2.6185416755191e-06,3.42040341679249e-05,4.39415598767832

"OR2G6",-1.64974331155,-0.07701678324,-5.2809553666769,2.62083451637868e-06,3.42040341679249e-05,4.39330832426513

"E2F8",-2.958458595475,0.32898775362,-5.27977976123747,2.63175184455455e-06,3.42998293837365e-05,4.38928235775907

"TPPP3",1.6825723154,0.04220134832,5.27706689226903,2.65711670508174e-06,3.45644489229934e-05,4.3799928653352

"RAN",-1.230759165,-0.036539098,-5.27642939819941,2.66311209899564e-06,3.46204572869433e-05,4.37781013468492

"CDKN2D",-1.02185555065,0.12967542748,-5.27577191253161,2.66930949632224e-06,3.46587737245486e-05,4.37555903290055

"FOXL1",0.73577337985,-0.01400971312,5.27550339694649,2.6718445914962e-06,3.46587737245486e-05,4.37463971122969

"COL6A1",1.2953615791,0.15261949328,5.2754254291022,2.67258114257717e-06,3.46587737245486e-05,4.37437277380365

"SLC22A17",0.62138261965,-0.05152147328,5.27518330936154,2.67486969116651e-06,3.46633334804677e-05,4.37354383896537

"ITPRIP",-1.02995702005,0.22731632296,-5.27407401733111,2.685379631274e-06,3.4777533429546e-05,4.36974614251047

"NUDT12",0.6091711575,-0.1002222557,5.2734340180295,2.69146181169405e-06,3.48219914384087e-05,4.36755518807038

"LINC01116",1.321753608825,0.08432597306,5.27335520390165,2.69221175509759e-06,3.48219914384087e-05,4.36728538338413

"NTN4",0.932475974425,-0.07281042046,5.27063406626767,2.71823120894672e-06,3.5136353948833e-05,4.35797080828171

"QSOX2",-0.787344205125,-0.0131500111,-5.2691563075852,2.73246535546046e-06,3.52785540515529e-05,4.35291294947419

"TNFAIP2",0.936744753775,-0.04387201398,5.26913449910131,2.73267597031504e-06,3.52785540515529e-05,4.352838309581

"AC046143.1",0.711286592175,-0.01319237826,5.26695538674237,2.75380169057823e-06,3.54619038570501e-05,4.34538070707774

"MAGI3",0.668558487125,-0.0253481293,5.26533056310565,2.76965862277407e-06,3.56436971503738e-05,4.33982062787022

"AL355802.1",-0.905678316025,-0.10209850382,-5.26448101576251,2.77798534908463e-06,3.57284142071851e-05,4.33691370759611

"GRM3-AS1",0.8473792808,0.01214705334,5.26183782383515,2.80405060918523e-06,3.60184256307129e-05,4.32787028266218

"TMEM71",-1.3229699906,0.20087404452,-5.26106215623766,2.81174539835191e-06,3.60946364169386e-05,4.32521665846527

"KCNT2",0.843090329575,-0.10653893234,5.26001190480632,2.8221973436038e-06,3.61778686714163e-05,4.32162383886565

"ANP32BP1",-0.6542891398,0.06370753116,-5.25990610331379,2.82325238809761e-06,3.61778686714163e-05,4.32126191250537

"PPIAL4A",-0.702003196275,0.02384733198,-5.25987861608202,2.82352655240115e-06,3.61778686714163e-05,4.32116788436557

"CALHM5",0.7175253959,-0.01499989748,5.2541078700868,2.88167111211102e-06,3.68997985905817e-05,4.30143048558239

"ATP11A",-0.606741603875,0.0266564949,-5.2532384416412,2.89053308698265e-06,3.69901573304813e-05,4.2984573633043

"PPWD1",0.670770641925,-0.02531223326,5.2528758655816,2.89423672194794e-06,3.70144331830645e-05,4.29721752982282

"TSPAN10",0.7956310268375,-0.09664452138,5.25258217965951,2.8972400922896e-06,3.70297286405673e-05,4.29621328515188

"E2F4",-1.223548887375,-0.0285917299,-5.24901778837379,2.93393736663898e-06,3.74287103161266e-05,4.28402631618421

"AL159141.1",-0.83443732005,0.03203996496,-5.24877767124624,2.9364259255686e-06,3.74371464611385e-05,4.28320542047637

"DOK7",1.20601022255,-0.14385903001,5.24849304982643,2.93937842247117e-06,3.74514832833267e-05,4.28223239066606

"CDV3",-0.709349888325,0.03191188784,-5.24363321154075,2.9902460307169e-06,3.8052273712626e-05,4.26562051309733

"FES",-0.85038852575,-0.0193677896,-5.24240776799071,3.00320921520373e-06,3.81698203480732e-05,4.26143241208118

"GYPB",-2.659724059125,0.3172300581,-5.2399924827225,3.02892155951175e-06,3.84637216264661e-05,4.25317871629735

"PDGFB",0.803707527175,-0.02016268526,5.23988337647525,3.03008818357655e-06,3.84637216264661e-05,4.25280589627029

"BCL11A",-1.18955132064375,-0.067546511165,-5.2388717416949,3.04092627754067e-06,3.85773978787945e-05,4.2493492108807

"HSP90AA2P",-0.5903150233,0.06861825826,-5.23605305317284,3.07132633811345e-06,3.88908121231572e-05,4.23971897431161

"TRIM22",0.745151354825,0.00570522286,5.23547649360224,3.07758148215634e-06,3.89459477494867e-05,4.23774930633688

"SRP14-AS1",0.75504466805,0.01606621329,5.23361087973483,3.09790795993608e-06,3.91789742488707e-05,4.23137634849985

"ZNF214",1.01070266475,-0.0105990502,5.23214348846719,3.11398869621468e-06,3.93580508377831e-05,4.22636418952779

"CASC1",1.1589082481875,-0.20806066365,5.2308046361894,3.12873265312112e-06,3.95200213299294e-05,4.22179143876802

"MARCKS",0.608503410725,0.03435045278,5.22667364350145,3.17466024912585e-06,4.00754400394888e-05,4.20768449851824

"UNC5C",0.63750540525,-0.02446845105,5.22578657705527,3.18460890600245e-06,4.0126855637256e-05,4.20465567848336

"CFI",1.018147490575,0.16729871746,5.22532444524609,3.189804002684e-06,4.01676118051566e-05,4.2030778247828

"SNHG15",-1.0904595947,0.15638980954,-5.22489369592551,3.19465383919252e-06,4.02039728853664e-05,4.20160715699021

"TMEM74B",0.81063290195,0.00631123156,5.22459912091438,3.19797466792819e-06,4.02210589297193e-05,4.20060143711167

"RTL3",1.3347316133,-0.16589134536,5.22060791279487,3.24330581667008e-06,4.07411707982444e-05,4.18697654240363

"CCNA1",-1.165790323925,0.07914385986,-5.21799751002545,3.27329642842495e-06,4.10675426978385e-05,4.178066997671

"LINC00310",1.48691563705,-0.12427912636,5.21620289543102,3.29407308248977e-06,4.13029187968484e-05,4.17194257495922

"H1-3",-1.498388488125,0.1445371772,-5.21427300055593,3.31656110105803e-06,4.15594518889767e-05,4.16535717953316

"SLC30A4",0.664055166825,-0.07313928554,5.21159781180825,3.3479842979435e-06,4.18763750282457e-05,4.15622980312119

"ENPEP",0.97548153955,-0.03534396236,5.21139166923981,3.35041783390866e-06,4.18812450159369e-05,4.15552653050084

"PRDX2",-1.1758013024,0.12067066988,-5.21044118998123,3.36166093776864e-06,4.19961642030511e-05,4.1522839975028

"SIM2",0.7892514058125,-0.07701674635,5.20739149681561,3.39798727653683e-06,4.23880929421109e-05,4.14188123913778

"AC007690.1",-0.625753625575,0.00128013154,-5.20728687101293,3.39924037016245e-06,4.23880929421109e-05,4.14152438382003

"PON3",0.87273720875,-0.154779109,5.20690531011646,3.40381415379052e-06,4.23991829462858e-05,4.14022298258475

"UBE2O",-0.808396312325,0.09504215174,-5.20686742272479,3.40426864245608e-06,4.23991829462858e-05,4.14009376046199

"AP005900.2",0.84664220365,-0.07415450808,5.2051181051749,3.4253183810302e-06,4.26354331655813e-05,4.13412768576185

"SSUH2",1.0223154513,0.09433074504,5.19722600801312,3.52189225305358e-06,4.37108257990597e-05,4.10721898244226

"LTF",-3.516464342575,0.85016653644,-5.19718648879723,3.52238253583793e-06,4.37108257990597e-05,4.10708426930629

"TPT1-AS1",0.79494588075,-0.0414505604,5.1962026726311,3.53460966499126e-06,4.38094874871995e-05,4.10373073452204

"ELK3",0.6251401801,-0.04296632882,5.19535922069057,3.54512563355416e-06,4.39132611730698e-05,4.10085581034153

"GAPDHP61",-1.11129501425,-0.0613633314,-5.19385063623903,3.56401133453839e-06,4.40850215493995e-05,4.0957141154877

"AP001024.1",-0.5893027402,0.03838323684,-5.19373626836358,3.56544712550541e-06,4.40850215493995e-05,4.09532433469248

"PFDN2",-0.79466826125,-0.0134008973,-5.18938716297254,3.62047181078554e-06,4.47114083540531e-05,4.08050391735067

"ARHGAP44",0.618731648675,-0.02589804556,5.18875317499093,3.62856262965269e-06,4.47843320218822e-05,4.07834379150738

"BICD1",0.67001938255,-0.09322461856,5.18774137852576,3.64151191256265e-06,4.4917095764349e-05,4.07489655944994

"ARHGDIB",-0.92292189545,0.18750353004,-5.18597183497872,3.66426872070989e-06,4.51328397957664e-05,4.06886813724329

"TFRC",-1.402268590425,-0.01566940734,-5.18589217513591,3.66529646018349e-06,4.51328397957664e-05,4.06859676926892

"APCDD1L",3.29717805925,0.4960644024,5.18586777220177,3.66561135397087e-06,4.51328397957664e-05,4.06851363886588

"HOXB3",0.95227652945,-0.00186934504,5.18454782793583,3.68268363307692e-06,4.52886088082113e-05,4.06401732614573

"AL450326.2",0.778336585275,0.03477063042,5.18096609418408,3.72940618577304e-06,4.58356772250258e-05,4.05181809580857

"HK3",-1.78573886085,0.23338586632,-5.18075380948703,3.73219364703476e-06,4.58424361153765e-05,4.05109514302687

"ADCY10P1",0.9647287249,-0.00729904308,5.1799602803759,3.74263150031882e-06,4.59431001668855e-05,4.04839279426196

"SIX1",1.02626708705,0.01041826264,5.17900272607435,3.75526522687487e-06,4.60705832144984e-05,4.04513202696481

"ISYNA1",0.8821289554375,0.04326299825,5.17771662710808,3.77229988407249e-06,4.625187314475e-05,4.04075275018232

"ANKH",1.041096756875,0.0193201155,5.17646305686831,3.78897701697639e-06,4.64008135826732e-05,4.03648455261387

"PTPN4",0.78617200125,0.0550414827,5.17409633624132,3.8206616242527e-06,4.67329643926504e-05,4.02842711545184

"CACNA1G",0.835877600375,-0.0822746517,5.17374237038993,3.82542276767366e-06,4.67632826158102e-05,4.02722214399557

"MROH7",0.89402460275,0.0132759122,5.17272912877642,3.83908410090639e-06,4.68866851100235e-05,4.02377300228063

"ABCF2",-0.91343589305,0.01948473926,-5.17265432476335,3.8400945731462e-06,4.68866851100235e-05,4.02351837256364

"BHLHE22",1.800806218675,0.12887643794,5.17237964954712,3.84380720803448e-06,4.69040631793988e-05,4.02258339923769

"ZNF674",1.165633815125,-0.0134580418,5.17085053081365,3.86454025872055e-06,4.71041096560622e-05,4.01737867161736

"HDGF",-0.8138286034375,0.11238316585,-5.17083147604356,3.86479931334638e-06,4.71041096560622e-05,4.01731381701444

"SARDH",0.861639754316667,-0.00654468934666666,5.17024719520207,3.87275108309394e-06,4.71729632523357e-05,4.01532520044439

"HSP90B3P",-0.764983579825,0.07624717734,-5.16995825146277,3.87668942204182e-06,4.71928775275062e-05,4.01434179734438

"SOX8",0.943308866075,0.02691642686,5.16722717697925,3.91410990985196e-06,4.7591859841571e-05,4.00504756933454

"SLC1A3",-1.298146650225,0.09006696982,-5.16574909406169,3.93451052855036e-06,4.78115371939144e-05,4.00001806897505

"H4C6",-1.40494325605,0.14114282016,-5.16508909532771,3.94365370765169e-06,4.78908092750965e-05,3.99777242026595

"IFIT1B",-3.5523607297,0.69476762084,-5.16494094189152,3.94570900314149e-06,4.78908092750965e-05,3.99726833951886

"TAS2R64P",1.06312647145,-0.11414405884,5.16394678143129,3.95952808920542e-06,4.80016636045211e-05,3.99388589834924

"RPL8",-0.622232105925,0.01508815866,-5.15978943042124,4.01783518213193e-06,4.86433021926723e-05,3.97974345479641

"ZNF577",0.84088864695,-0.01541717029,5.15966609531486,4.0195778315206e-06,4.86433021926723e-05,3.97932394747214

"FO393415.1",1.6918471384,-0.14328704528,5.15510823437787,4.08450285360119e-06,4.93998196367068e-05,3.96382316200084

"RBM38",-1.8050275519,0.17763277348,-5.15483235775623,4.08846562563878e-06,4.94185744767477e-05,3.96288507030819

"ELOVL6",-0.66839718695,-0.02123241336,-5.15348276763012,4.10790622095199e-06,4.95750756117364e-05,3.958296141513

"CHMP4C",1.194334612975,-0.02862388162,5.15234738259897,4.12433179106454e-06,4.97137910537296e-05,3.954435846302

"SNX21",0.705245198166666,0.0260321465666667,5.1522997909322,4.12502170984675e-06,4.97137910537296e-05,3.95427404090982

"PICART1",1.065807754775,-0.02309112318,5.15025831881005,4.15472353045225e-06,5.00423137518552e-05,3.94733373639657

"HINT1",-0.666846879225,-0.01687040338,-5.14922662918324,4.16981390017148e-06,5.01150576271932e-05,3.94382666683467

"IFIT5",0.765310407025,0.02595796712,5.14919901712805,4.17021851906374e-06,5.01150576271932e-05,3.94373280688131

"SSR4P1",0.93868218925,-0.0746355635,5.14846975255576,4.18091896753116e-06,5.02102390426603e-05,3.9412539188321

"MYB",-1.909161982975,0.26169966822,-5.14829478444392,4.18349028566711e-06,5.02116865686864e-05,3.9406591900079

"SYNCRIP",-0.659774783625,0.0106454861,-5.14800292604756,4.18778288391572e-06,5.02337796988672e-05,3.93966715676812

"PCDHB5",0.681383425125,-0.1061543949,5.14746313251934,4.19573350946888e-06,5.02702854631569e-05,3.93783243199679

"TNFSF10",0.87541102045,-0.09067417084,5.14561530944907,4.22306293131165e-06,5.05681550769802e-05,3.93155224372899

"MSRA",-0.608327057275,0.08983266948,-5.14382326633664,4.2497349457276e-06,5.0857809327142e-05,3.92546229743555

"PPIAP66",-0.68507742995,0.00132379504,-5.14176480648552,4.28057714944264e-06,5.11970021236316e-05,3.91846778685363

"HAT1",-0.68527002115,-0.02871356992,-5.14134577234194,4.28688257402402e-06,5.12425030201891e-05,3.91704404227027

"FAM107B",-0.790574383975,-0.03857179573,-5.13698694754434,4.35301741835902e-06,5.19723897828319e-05,3.90223626854098

"AC093789.1",-0.761561386325,0.02762155394,-5.1367686498669,4.35635588141856e-06,5.19819564930131e-05,3.9014947710369

"COL6A2",1.0244045785125,-0.04647661799,5.13356008806251,4.40571707584153e-06,5.25097914193375e-05,3.89059728945547

"FAM43A",0.74082954055,0.07920490384,5.13300488290907,4.41431423808289e-06,5.25816686685129e-05,3.88871181818964

"IFIT3",1.3768670625,0.024165352,5.13057136088878,4.45219196676487e-06,5.30020389396157e-05,3.88044834739667

"C10orf142",0.609070275225,0.01767900518,5.12982071917918,4.46394020232492e-06,5.31110376685441e-05,3.87789965109027

"CDKN3",-1.9584286455875,-0.06352575567,-5.12773545333028,4.49673710016032e-06,5.34701971608152e-05,3.87082003785159

"LINC00575",0.666898325775,-0.03056214088,5.12745454137699,4.5011733722908e-06,5.34919025820731e-05,3.86986639188036

"PCMTD1",1.1148218767,-0.16224514864,5.12699283425447,4.50847420518882e-06,5.35476055164687e-05,3.8682990138616

"CEP19",0.637552990925,-0.06813968656,5.12654297839315,4.5155988620449e-06,5.36011526567647e-05,3.86677191008602

"TRA2B",-0.6289538356,0.0353084435533333,-5.12529326788526,4.53544961052973e-06,5.37744743174382e-05,3.86252979756123

"GAPDHP35",-1.02618781145,-0.09974969916,-5.12233698646277,4.58275098348103e-06,5.42411335352741e-05,3.85249605336066

"GABPB1",-0.66532151,0.0220498162,-5.12113404513146,4.60213725590201e-06,5.44077253888751e-05,3.84841373636132

"HSD17B11",-0.6562469576,-1.32070799999969e-05,-5.12007716571751,4.61923631103795e-06,5.45783815112719e-05,3.84482734392763

"SLC35A5",0.61265089285,0.02808029128,5.11956517901436,4.62754214319683e-06,5.46450048586839e-05,3.84309006210801

"TMTC2",1.053620338975,0.05525793818,5.11451639614731,4.71023944220343e-06,5.54936087934812e-05,3.82596135764624

"LRFN4",0.655739392125,0.0921666851,5.11361574161303,4.72514414838083e-06,5.56372145471416e-05,3.822906316936

"ST8SIA1",0.8550076729,0.03766364957,5.11329601367021,4.7304464150382e-06,5.56676543086172e-05,3.82182183302773

"ENO3",0.87150626165,0.07112564232,5.11269788506028,4.74038133710442e-06,5.57525446811684e-05,3.81979310012425

"NOP58",-0.682622144575,0.06306213164,-5.10770334515824,4.82414728729077e-06,5.66726660676682e-05,3.80285552632958

"WDR43",-0.731962084,0.1202966688,-5.10640617680417,4.84614026626887e-06,5.68658200316819e-05,3.79845739694897

"HRH1",0.6900900967,-0.10425135514,5.10081370546477,4.94209488820118e-06,5.79585804633462e-05,3.77949980806018

"ATP1B3",-1.1293327533,0.12195856436,-5.09990601704816,4.9578444113821e-06,5.81100207668172e-05,3.77642350691887

"RPS18P12",-0.70508914045,-0.04408859196,-5.0953518231107,5.03761482132465e-06,5.89101897598741e-05,3.76099122917134

"DENND3",-0.868124514133333,0.01178096686,-5.09463694809456,5.05025072624579e-06,5.90242651907152e-05,3.75856921004624

"AL596275.1",-0.875349706825,-0.09907690246,-5.0919866734371,5.0973691751318e-06,5.95409918244585e-05,3.74959093598397

"TPI1P2",-0.836470774325,-0.02546037716,-5.09160337115276,5.1042195410062e-06,5.95870370120427e-05,3.74829255439656

"GPX3",-2.265894070625,0.0472673797,-5.08468260424414,5.22947892592785e-06,6.09451446157052e-05,3.72485479356125

"KLRA1P",0.658714825175,-0.04091277086,5.08446688997009,5.23343142316451e-06,6.09565338247837e-05,3.72412442206537

"PKD1L1",1.8732614831,-0.06530773552,5.08388515543066,5.24410512301118e-06,6.10114853834486e-05,3.72215481802695

"CCDC3",0.6589641808,0.05131856864,5.08316699964976,5.25731146598154e-06,6.11304184534789e-05,3.71972342574224

"VAV2",0.586774670075,-0.02842600794,5.0815994126116,5.28625210504506e-06,6.14157217653878e-05,3.71441657483644

"LINC02139",0.638813003875,-0.0206816765,5.0815134708039,5.28784328358766e-06,6.14157217653878e-05,3.71412564573986

"AP5B1",-1.329264028375,0.0168788533,-5.08032980209182,5.309806492112e-06,6.16358727537624e-05,3.71011886556275

"AC116351.1",1.350164627825,0.12614177676,5.07993663432468,5.31712165834143e-06,6.16858372231592e-05,3.70878803818304

"ENPP6",1.39616086835,0.05645415508,5.07858166817281,5.34240789819423e-06,6.19441160261479e-05,3.7042018868222

"MYL4",-0.989679480625,-0.00874201559999999,-5.07707163648879,5.37072751445723e-06,6.21742081331424e-05,3.69909134645735

"H3C10",-1.744399314575,0.27422245034,-5.07675637148526,5.37665872505618e-06,6.22004426645686e-05,3.69802442721419

"ATP5F1B",-0.84624501215,-0.03593202572,-5.0756949650951,5.39667486178234e-06,6.23967689436775e-05,3.69443257359453

"HSP90B1",-0.6993411355,-0.0176115607,-5.07486625368841,5.4123538093648e-06,6.25342061392871e-05,3.69162833895817

"ACVR2B",0.670451312375,-0.0651226695,5.07474423078859,5.41466622857747e-06,6.25342061392871e-05,3.69121544422775

"NCOA7",-1.02877283395,0.09220085384,-5.07446083677955,5.4200404978105e-06,6.25610082924741e-05,3.69025652262204

"TREML1",-1.61284665185,0.15507724152,-5.07368488980835,5.43478241466037e-06,6.26605639344748e-05,3.6876310351544

"SCAI",0.592292072925,-0.02648977756,5.07345942691331,5.43907329629414e-06,6.26747658574097e-05,3.68686818498835

"S1PR5",0.878949189925,0.01524636294,5.07314294706891,5.44510198987534e-06,6.27089654685587e-05,3.68579739874462

"SLFN12",0.71968930235,-0.01420379312,5.072266447147,5.46183300126298e-06,6.28310132116092e-05,3.68283193767432

"DCXR",-1.0390295667,0.06463882564,-5.07201236710775,5.46669244980929e-06,6.28516245295695e-05,3.68197233974066

"ENTPD3",1.9776230991,0.07167081528,5.07053684308101,5.49499709889721e-06,6.31416155705025e-05,3.67698065295947

"ASPRV1",0.768781355475,-0.10459762662,5.07009288520066,5.50354165452027e-06,6.31850129010445e-05,3.67547883805291

"CELSR2",0.745451113075,-0.12952415104,5.07002020011052,5.50494182098616e-06,6.31850129010445e-05,3.67523296390621

"RPGRIP1L",0.810219620325,-0.03213518174,5.06900175504671,5.52459753244767e-06,6.33396498292042e-05,3.67178795681541

"APBB2",0.62206069335,0.01333177668,5.06802551121433,5.54350364330652e-06,6.34427507598811e-05,3.66848590910818

"ABCC9",0.9193184139,0.16119711412,5.06797424611188,5.54449821120859e-06,6.34427507598811e-05,3.66831251564887

"NMNAT2",1.342105061525,0.01201214022,5.06793358697128,5.5452871432454e-06,6.34427507598811e-05,3.66817499503279

"RSPO2",2.499893200525,-0.34824055358,5.06789806410035,5.54597650385333e-06,6.34427507598811e-05,3.66805484701167

"NRK",1.5370975234,-0.07216273228,5.06454355362754,5.61145618913495e-06,6.41559790195295e-05,3.65671020100636

"ANGPTL2",0.972253243275,0.05966266262,5.06377794992606,5.62650706512766e-06,6.42921788903154e-05,3.65412133889058

"MTX3",0.8030626743,-0.01807680156,5.0626795392852,5.64816998970742e-06,6.45037384331804e-05,3.65040732220274

"CRTC1",0.6021989338125,-0.0337116548,5.06032100768663,5.69496298972975e-06,6.49883508650652e-05,3.64243338220363

"AP2S1",-0.8095210178,0.00632556786,-5.06022136016299,5.69694836751548e-06,6.49883508650652e-05,3.64209651113467

"NR1D2",0.6982973417,-0.0662414063066667,5.05819396761152,5.7374904621944e-06,6.54144154643511e-05,3.63524311956055

"MAFG",-0.588020282075,-0.03656884786,-5.05747875450198,5.75186037361425e-06,6.55417771605166e-05,3.6328256272857

"TTC28",0.5992658940125,-0.02845643604,5.05583674925494,5.78498540596333e-06,6.58825908823662e-05,3.62727590294146

"RPSAP45",-0.614939068525,-0.01323993782,-5.05520870136118,5.79770494652406e-06,6.5954124899714e-05,3.62515335227796

"ENHO",1.3364933384875,-0.13198875171,5.05476815958358,5.80664344736587e-06,6.6019151470384e-05,3.62366454826536

"CCDC18-AS1",0.901367717,-0.0904583072,5.05456728454439,5.81072366276579e-06,6.60288998351334e-05,3.62298570818509

"LSMEM2",1.02406638915,-0.01720070768,5.05251620639866,5.85254749449141e-06,6.6467291057173e-05,3.61605476500224

"AVPR2",1.15170189915,0.13423158832,5.05076904479559,5.88840778651377e-06,6.68375062216588e-05,3.61015152766619

"ILF3",-0.614599135483333,-0.01982611842,-5.04876529407785,5.92980091793775e-06,6.72700781875463e-05,3.603382154149

"GCSAML",-1.140624751875,-0.1423593585,-5.04430978245464,6.02287155673023e-06,6.82458679447084e-05,3.5883330037764

"LINC00174",0.803261140425,-0.00355360066,5.04417026533991,6.0258090156178e-06,6.82458679447084e-05,3.5878618339412

"DICER1-AS1",0.8017981715,-0.0939501758,5.04400631089008,6.0292627922996e-06,6.82472575075328e-05,3.58730814105746

"DHX9",-0.592483015425,-0.00543047864,-5.04372014030189,6.03529577875173e-06,6.82617507056697e-05,3.58634172438864

"PDZD2",0.8985537996,0.15788119193,5.04362955252505,6.03720676877555e-06,6.82617507056697e-05,3.58603580730614

"DLGAP3",-0.64312939065,0.06004855328,-5.04212533339672,6.0690261591962e-06,6.85458698729944e-05,3.58095628540647

"ZNF622",-0.58599844295,0.00405977244,-5.03936316806391,6.12788593237117e-06,6.91725217533998e-05,3.57163015640787

"RPSAP18",-0.606444386125,-0.0003221689,-5.03913633837645,6.13274440498884e-06,6.91814383010414e-05,3.57086436661848

"AP000866.6",1.15256364305,-0.03679298656,5.03840114849507,6.14851753900328e-06,6.92559145306763e-05,3.56838240166355

"ALOX5",-0.7164153259875,0.000418414710000001,-5.03838824037962,6.14879483264839e-06,6.92559145306763e-05,3.56833882555709

"ZC3H6",0.79573596565,-0.01488471248,5.03781498275767,6.16112204138115e-06,6.93566309801192e-05,3.56640361975738

"IL21R",1.1376216325,0.196078671,5.03664113314115,6.18644021304976e-06,6.95651959851611e-05,3.56244115745943

"ENSAP2",-0.7122309453,-0.08480207524,-5.03236178104949,6.27960964022363e-06,7.05741318205715e-05,3.54799828146814

"MYCN",0.869288757575,-0.13453496794,5.03145653554722,6.29949478795674e-06,7.0758798912093e-05,3.54494358056171

"PMF1-BGLAP",2.252459570075,-0.24828103594,5.03078691473251,6.314243936197e-06,7.08856053505777e-05,3.54268409821796

"RUNDC3B",1.019437226375,0.1975993931,5.0289848915297,6.35410454253154e-06,7.1294027309631e-05,3.5366040735574

"TMEM86A",0.707312059525,-0.08396429238,5.02769986608646,6.3826803822266e-06,7.1575454663962e-05,3.53226883623596

"AC087071.1",0.599743592325,-0.05161515214,5.02701060531019,6.39805994603169e-06,7.17086718677775e-05,3.52994365575233

"ADAMTS6",1.17997897121667,0.01341214364,5.02444135803202,6.4557099413513e-06,7.22757296603308e-05,3.52127737671153

"UBXN2B",-0.659258783375,0.0473680026,-5.02190696747856,6.51307862452104e-06,7.27986660442919e-05,3.51273010288174

"SPOCK1",1.382136162825,0.13461227526,5.02015415622226,6.55304862149401e-06,7.31687843700209e-05,3.50681955055527

"CCDC136",0.7165613153125,-0.0638854564,5.02014165303342,6.5533346016687e-06,7.31687843700209e-05,3.50677739173095

"AC041040.1",0.87499611385,-0.00426738012,5.0196823879089,6.56384769562017e-06,7.32462481415393e-05,3.50522884460337

"HMGA1P8",-1.3737745314,0.26250843688,-5.01862554343152,6.58810313771613e-06,7.34115606134711e-05,3.50166556116758

"PPIAP26",-0.7503622018,-0.07084669344,-5.01856865114656,6.5894113611955e-06,7.34115606134711e-05,3.50147374874251

"C11orf58",-0.596321237525,-0.01676475852,-5.01733386387704,6.61786805147442e-06,7.36692387509931e-05,3.4973108387579

"FAT1",1.238632548,0.0061714944,5.01375006990239,6.70114620235313e-06,7.44135953353989e-05,3.48523050648453

"SEMA5B",0.9060350016375,-0.01168071999,5.01357205228688,6.7053096516006e-06,7.44194930346658e-05,3.48463051530852

"PGK1",-0.796453582425,0.02300888156,-5.01295136012137,6.71984622789808e-06,7.45404491159588e-05,3.48253858757192

"LMNB2",-0.8783617372,-0.07532990476,-5.01194639173048,6.7434483147794e-06,7.47617798015153e-05,3.47915171028039

"CYB561D1",0.759047551075,-0.05389200134,5.01163001777075,6.7508953535222e-06,7.4803863711716e-05,3.47808553453547

"DDIT4",-1.59295704915,0.07994039818,-5.01023355804482,6.78386304588114e-06,7.50879449400393e-05,3.4733797520609

"MYC",-0.85883795315,-0.05571884092,-5.00646104324741,6.87371892657228e-06,7.59594138983889e-05,3.46066933093052

"ADAM22",0.726533645425,0.02444189734,5.005023083935,6.90827645733151e-06,7.62734344679112e-05,3.4558253723759

"COX8A",-0.683914830525,-0.03138040442,-5.00439602730839,6.92339957297433e-06,7.63848198444254e-05,3.45371319323426

"SUGT1P3",0.977867021525,-0.14292662208,5.00291936756551,6.95914189804044e-06,7.6737835956433e-05,3.44873955716015

"PYGL",-1.059790446775,-0.25408378942,-5.00272042759512,6.96397106673165e-06,7.67497790291544e-05,3.4480695315919

"KIF17",0.646317380675,0.02958737374,5.0016790533683,6.9893037633873e-06,7.69734727048089e-05,3.44456235046641

"GAPDHP76",-0.913214903875,-0.0536652371,-5.00146102093588,6.99461913603314e-06,7.69734727048089e-05,3.44382808309423

"PTH1R",1.015426592625,0.0327174767,5.00031391019487,7.02264991648297e-06,7.71888688245188e-05,3.43996513658779

"FAM135B",1.10348064185,-0.05196473602,5.00015862373918,7.02645297778486e-06,7.71892593076977e-05,3.43944222526641

"CRIP3",0.9219419277625,0.03891167176,4.99955414554651,7.04127632893377e-06,7.73106481389042e-05,3.4374067581737

"CMSS1",-0.872714420825,0.08845317834,-4.9983207051258,7.0716189545205e-06,7.76022116444649e-05,3.43325363363168

"AP001462.1",0.77618760265,-0.04717358688,4.99669662101925,7.11176770163523e-06,7.8001015348556e-05,3.42778569295286

"CREM",-0.678916822575,0.03520735349,-4.99645041518624,7.11787366434181e-06,7.80262148930097e-05,3.42695682293175

"ZMYND10",0.732297036825,-0.03788516174,4.99586479844107,7.13241782996329e-06,7.81438377006887e-05,3.42498535606053

"OPCML",2.45195263575,-0.0323252914,4.99341750267352,7.19351504481244e-06,7.87711043496084e-05,3.41674742197291

"PDE3A",1.3215251441,0.08793501828,4.99321660602577,7.19855327702546e-06,7.87841664207787e-05,3.41607123629455

"WLS",0.65761631885,-0.0593573909533333,4.9915597612211,7.24023743209932e-06,7.91980696790448e-05,3.41049491204357

"MT1CP",-1.574443000325,0.23160531874,-4.99137829191899,7.24481737794703e-06,7.92058796368083e-05,3.40988419148787

"PCDHGA5",0.8717661951375,-0.07046917389,4.99016964368733,7.27539419204278e-06,7.94130400674333e-05,3.40581677173233

"KCTD15",0.74768006925,0.04593379635,4.98844453982314,7.31925651958364e-06,7.98492692083225e-05,3.40001191568645

"ANXA3",-2.61774655675,0.4819973896,-4.98770182924655,7.33822062341202e-06,8.00135519598007e-05,3.39751295327705

"PLCD4",1.27927363395,0.18024139016,4.98701471063337,7.35580830370833e-06,8.01626598544555e-05,3.39520114986296

"TRGC1",-0.9889174458875,-0.09693807056,-4.9857197183697,7.38906808367843e-06,8.04823109507728e-05,3.3908444531133

"RGS17",1.12330943385,-0.03417690832,4.98478129417441,7.41326239290122e-06,8.06677407412756e-05,3.38768758367865

"CKS1BP3",-0.774586519575,-0.12303282766,-4.98475400991547,7.41396699608659e-06,8.06677407412756e-05,3.38759580212091

"PFKFB3",-1.143016101725,0.14137701617,-4.98301903590652,7.45890743284266e-06,8.11136387919748e-05,3.38175986807569

"PPIAP9",-0.674511458625,-0.0206737719,-4.98273042920824,7.46640906398757e-06,8.11521426540994e-05,3.38078914780598

"LINC00685",1.1505101903,-0.04184112536,4.982432885374,7.47415076413592e-06,8.11932136031903e-05,3.37978838758039

"TNFRSF11A",1.0810224255,-0.0582296476,4.98200598755503,7.4852718786246e-06,8.1270932829497e-05,3.37835259279285

"RAI2",0.714017070225,0.14938287188,4.98167280759751,7.49396285745688e-06,8.1322198635369e-05,3.37723203016669

"LGALS12",-1.84369049755,-0.19112060204,-4.98128552240617,7.50407764352831e-06,8.13888527054568e-05,3.37592953067052

"PTPN7",-1.360173284975,0.20426393552,-4.98078546244996,7.51715765178511e-06,8.14799850873324e-05,3.37424780287647

"CYB561",0.705106013775,0.07696971852,4.98066033887491,7.52043400039758e-06,8.14799850873324e-05,3.37382701465805

"P2RX5",-1.52637767875,-0.064621039,-4.97901299557516,7.56370073381989e-06,8.18189549284593e-05,3.36828736303602

"COL10A1",2.0484485019,0.14344106552,4.97673478548796,7.6239406084586e-06,8.23401661497627e-05,3.36062726591092

"PRSS21",-1.0377322305,-0.0226218604,-4.97629131442863,7.63572151340322e-06,8.2380548902899e-05,3.35913630709347

"SLPI",-1.400363834175,0.02486923466,-4.97430126644546,7.68880841102977e-06,8.28660214230289e-05,3.35244627840119

"ZNF514",0.845325143675,-0.06378585706,4.97392940910013,7.69876832558981e-06,8.29297399866898e-05,3.35119629024646

"CHAF1B",-0.616153165925,0.04745594046,-4.97159874805219,7.76148294649408e-06,8.34298334773194e-05,3.34336255890228

"AL451081.2",-0.82924276,-0.026318397,-4.97124700380297,7.77099139761356e-06,8.34882389901975e-05,3.34218039729502

"TRIM66",1.091536181925,-0.09701465661,4.97090746100689,7.78018088291065e-06,8.35431582437491e-05,3.34103926991897

"ADRA2C",1.11113155075,-0.0368610764,4.97051552203566,7.79080172337585e-06,8.36133817226425e-05,3.33972208359658

"LILRA5",-1.2551459459,0.04906704228,-4.96983901732228,7.80916734159495e-06,8.37666075887944e-05,3.33744864225962

"TMEM130",1.344227579775,-0.10076062918,4.96904425357879,7.83079783484345e-06,8.39543227930204e-05,3.33477791737905

"ELOCP29",-0.611355494675,0.00788406336,-4.96889514355086,7.8348626112971e-06,8.39543227930204e-05,3.3342768640485

"ANO5",2.09102003875,-0.054944847,4.9685485304182,7.84431939163517e-06,8.40117175618513e-05,3.33311216221796

"C2CD6",1.419838305225,-0.06798964482,4.96732312201639,7.87784277256891e-06,8.43266680900689e-05,3.32899472242195

"PROCA1",0.61606367045,-0.04078787704,4.9654430365561,7.92955019584809e-06,8.48358352023685e-05,3.32267820283465

"DPP3",-0.617459489525,0.07572626238,-4.96468965108934,7.95036387513722e-06,8.49697731214457e-05,3.32014728338213

"C1QTNF7",0.902589021,-0.0867637634,4.96367608595677,7.97845028926411e-06,8.52254898469464e-05,3.31674252386539

"CSF2RA",-0.827247267225,0.09217795952,-4.96326547643981,7.98985624355444e-06,8.53028529014817e-05,3.31536327518256

"ARHGEF4",0.58869090305,-0.0192853292266667,4.96256615310796,8.00931904457106e-06,8.54661086381103e-05,3.31301431830784

"CRYGD",-0.8781271812,-0.15006376896,-4.95935750187612,8.09921809504173e-06,8.63354736374688e-05,3.30223822762135

"ARL10",0.865847255,-0.087231527,4.95905627929071,8.10770843631298e-06,8.63810350718567e-05,3.30122670910305

"FZD7",0.7647489832,-0.06793690694,4.95873109468068,8.11688400232913e-06,8.64338458626399e-05,3.30013474860933

"ZSCAN16-AS1",0.7752888489,0.00541898652000001,4.9562905289322,8.18607486317547e-06,8.7034925685905e-05,3.29194018027192

"TIPIN",-0.64360089755,-0.01696859434,-4.95538061699947,8.21201932433901e-06,8.72309437258004e-05,3.28888536633369

"UBA52",-0.62697563285,-0.06654928228,-4.95506401629808,8.22106555748822e-06,8.72309437258004e-05,3.28782249940305

"NREP",1.25234722695,-0.06412658944,4.95450586002043,8.23703763087997e-06,8.73501174852323e-05,3.28594875770789

"S100A10",0.859990430325,0.03382278426,4.95418054481118,8.24636084792532e-06,8.74037460177413e-05,3.28485670141485

"SLC6A8",-1.067471003875,0.1363807769,-4.95319495414098,8.27467030311366e-06,8.76333987433055e-05,3.28154830476106

"EXOSC4",-0.689996759275,0.09704006378,-4.95312849349756,8.27658271028388e-06,8.76333987433055e-05,3.28132522008437

"COL3A1",2.77237427885,0.32928009308,4.95259981147029,8.2918110401464e-06,8.77159999421639e-05,3.27955065958695

"ZNF117",0.943791288075,0.15599198846,4.95256042319513,8.29294669503961e-06,8.77159999421639e-05,3.27941845251238

"TBX3",1.1194770912,0.24961933966,4.95099151126844,8.33830663399971e-06,8.81048098594049e-05,3.27415267952351

"TUBB6",-0.839590923575,0.04549787414,-4.95012659536222,8.36341706269823e-06,8.83245818456502e-05,3.27124998815256

"ARL4D",0.850052124725,0.02974272678,4.94691736032913,8.45723972498452e-06,8.92149177527545e-05,3.26048119169613

"OLAH",-1.0195632200375,0.20720317597,-4.94522951701516,8.50699865671947e-06,8.96560640323398e-05,3.2548184866142

"OLFM4",-3.536886867875,-0.2088668433,-4.9440373576758,8.5423177827181e-06,8.99820086027395e-05,3.25081919899347

"MTSS2",0.850195602475,0.09697686498,4.94281873500243,8.57856991756689e-06,9.02710531438677e-05,3.24673147886846

"RASAL2",0.8503541625625,0.08570845095,4.94219533584206,8.59717351358174e-06,9.04203752290876e-05,3.2446404970691

"GUCY1B1",0.9835169788,0.01308594604,4.94118676213893,8.62735559103532e-06,9.06447494098111e-05,3.24125776982997

"B4GALT3",-0.660055975,-0.08909195455,-4.93700803852937,8.7535198188785e-06,9.18760830169994e-05,3.22724498772569

"RHOBTB1",0.7332751325,0.0952399845,4.93408136557665,8.84296032141579e-06,9.2672414867093e-05,3.21743322556302

"GXYLT2",1.7500430947,-0.07537953724,4.93281762794564,8.88185788913921e-06,9.30324664788773e-05,3.21319712783756

"RASL10B",-1.1455307471,0.21683102732,-4.93227561491201,8.89859242592748e-06,9.31137459715878e-05,3.21138039413426

"YOD1",-1.168110939275,0.20498210858,-4.93227189860109,8.89870727315349e-06,9.31137459715878e-05,3.21136793794303

"COL16A1",1.23367727855,0.04258018529,4.93185302839185,8.91166119423902e-06,9.32016919589428e-05,3.20996400534806

"ITGA2B",-1.0148276546,-0.06373271028,-4.92704995743064,9.06153170789374e-06,9.4595512088852e-05,3.19386844572503

"PLXNA1",0.695439386275,-0.03856006698,4.92689053148808,9.06654853503079e-06,9.4595512088852e-05,3.19333428712686

"U82695.1",1.76017317635,0.13450521508,4.92491536062799,9.12893025682226e-06,9.51824545047198e-05,3.18671695061091

"HLA-DOA",1.043066226225,-0.14263707722,4.9224818433293,9.20636823543136e-06,9.57462296484861e-05,3.17856530258279

"PHKG1",0.724536629875,0.0505550669,4.92193666442906,9.22380492818817e-06,9.58789017598778e-05,3.17673928769995

"DNTT",-2.904713955975,0.75575701422,-4.91847294827041,9.33534714091227e-06,9.69208598905203e-05,3.16513960902497

"SLC6A1",1.5892231511,-0.23743609562,4.9183837846404,9.33823591487149e-06,9.69208598905203e-05,3.16484104550242

"ZNF474",1.112669889125,0.1172540573,4.9166155603319,9.39570566361824e-06,9.73693564168996e-05,3.15892055414649

"NBEAL1",0.789147843075,0.08665784196,4.91533661597947,9.43748962842049e-06,9.7752925938867e-05,3.15463876704174

"C1S",0.6697811552375,0.07334673789,4.91240581861189,9.53393070099009e-06,9.87019566457226e-05,3.14482819736499

"PCK2",-0.7813545056,0.04263173102,-4.91055044685101,9.59548368627985e-06,9.92890251335866e-05,3.1386185722689

"KRT8P44",0.760893540125,-0.00198506590000001,4.90841489107787,9.66681548322567e-06,9.98254615021812e-05,3.13147223426834

"BMF",0.636558371875,-0.1340553628,4.90760205357697,9.69410256648314e-06,0.000100056812786955,3.12875247381241

"LINC00342",1.097060226175,-0.09461154136,4.90717558461329,9.70844945707591e-06,0.000100154437299381,3.12732556860061

"TMEM17",0.685490002475,-0.09945169372,4.90635154604409,9.73623016530767e-06,0.000100390479932976,3.12456857460865

"FZD1",1.123588449325,0.07515184346,4.90398490979354,9.81645134640559e-06,0.000101166727960341,3.11665140010362

"MATN4",2.714390880425,0.70277952834,4.90374386574975,9.82465830040436e-06,0.000101200401839459,3.11584510400541

"MXI1",-1.0177119888125,-0.11397177335,-4.90301254200975,9.84959934772282e-06,0.000101364755052275,3.11339889989088

"RAMP3",0.78280918805,0.07197517354,4.90298590505479,9.85050894714367e-06,0.000101364755052275,3.11330980438498

"STMN3",0.7169608799,0.02112329442,4.90276292478408,9.85812652138286e-06,0.000101392216952857,3.11256398481748

"SNAI1",0.75724837655,-0.03862106396,4.90136090366349,9.90615592794724e-06,0.000101835084120313,3.10787480832346

"LYAR",-0.6690902525,0.052818785,-4.90082329501136,9.92463385378481e-06,0.000101893406815303,3.1060768565269

"HSP90B2P",-0.758153539825,0.04085323354,-4.90076180912579,9.92674931683647e-06,0.000101893406815303,3.10587123059393

"DUSP21",-1.3373973135,-0.0443359008,-4.9004319105603,9.9381072662397e-06,0.000101939701072819,3.10476797337849

"RPL4",-0.5996429416,-0.01099344228,-4.89939397222644,9.97392539362794e-06,0.000102224003734192,3.10129703773125

"EBF3",0.894617772725,-0.13245927822,4.89759772838252,1.00362117600914e-05,0.000102759573483634,3.09529088940834

"ARID5B",-0.9287168713125,0.0791202795,-4.89740274791864,1.00429958339803e-05,0.000102777671651643,3.09463897474762

"ARMCX4",0.7377374229,0.05296018732,4.89453429458337,1.01433219497256e-05,0.000103531203567725,3.08504939624559

"ITPKC",-0.965513919925,0.26945258966,-4.89332902964773,1.01857701214142e-05,0.000103823909575888,3.08102064490835

"ACVR2A",0.5998188295,0.0026766881,4.89220792849409,1.02254107812147e-05,0.000104176139276741,3.07727353641859

"ZFP28",1.095604925375,-0.0930426777,4.89194539376977,1.02347155438126e-05,0.000104219111362641,3.07639609826385

"CASZ1",0.772271378475,0.00460032618,4.89062023393517,1.02818090070518e-05,0.000104646648254584,3.07196743048621

"CFL1P2",-0.736827136025,-0.02771720882,-4.88693597841531,1.04138614891416e-05,0.000105938030878616,3.05965692614814

"H1-4",-1.408025077925,0.02634082446,-4.8867366340418,1.04210537859282e-05,0.000105958585591115,3.05899093421935

"AP001094.3",0.865568137375,0.0381770909,4.88586309595403,1.04526284169234e-05,0.000106226910221193,3.05607263428124

"PDGFA",0.760322985075,0.04427623906,4.88296876270874,1.0557919131804e-05,0.000107190608113181,3.04640462199264

"MREG",0.7752506464625,0.02497856497,4.8825286691457,1.05740199478149e-05,0.00010730090177852,3.04493474484248

"STXBP6",1.1513670670625,0.08027349325,4.87942983894222,1.06880754650938e-05,0.000108404599073685,3.0345862341791

"COX19",0.773777913425,-0.01693162196,4.87626288763085,1.08058867299429e-05,0.000109491101544545,3.02401264982241

"GASK1B",0.75180098865,-0.03639919178,4.87604103478298,1.08141873460905e-05,0.000109494818736054,3.02327203549818

"NES",0.59114994055,0.04161373169,4.87596744028162,1.08169422648269e-05,0.000109494818736054,3.02302635664467

"FAM189A2",0.9294374368125,0.08738022825,4.87424073487669,1.08817775294377e-05,0.000110096720011417,3.01726251463878

"SLIRP",-0.67660100295,-0.01833425576,-4.87363501294418,1.09046118186843e-05,0.000110273290691611,3.01524075251716

"HBQ1",-2.5107753784,0.35346463728,-4.87295995149715,1.09301155001652e-05,0.000110476668163486,3.01298765652054

"THBS3",1.0923468811,-0.01965972912,4.87185226781529,1.09720905540658e-05,0.000110846248161588,3.00929087501156

"FOXO3",-0.703319697475,-0.01222249853,-4.87128855050073,1.09935130569982e-05,0.000111007932731286,3.0074096411308

"C10orf105",2.2326579301,-0.04083766092,4.87091624269301,1.10076840965363e-05,0.000111096271807801,3.00616722050403

"RAB21",-0.633387138075,0.05719600794,-4.87047562844734,1.10244782458511e-05,0.000111210984884785,3.00469689973633

"LCAT",1.06412942515,-0.00610440987999999,4.87024585690691,1.10332460407559e-05,0.000111244657914865,3.00393017564522

"SHC3",1.11512835984167,0.01482504554,4.86774578990561,1.11290892544829e-05,0.0001121558193044,2.99558854444142

"EPB42",-2.87877974125,0.549409615,-4.86705144375902,1.11558526625966e-05,0.000112370260251366,2.99327208671701

"HTRA2",-0.6257183467,-0.01329889436,-4.86494041505774,1.12376105974359e-05,0.000113027081944166,2.98623006025351

"PELI3",0.651597205575,0.000876676460000004,4.8628034477927,1.13209723950845e-05,0.000113724655848509,2.97910262280826

"AP006621.5",1.043443219875,-0.0256345086,4.86268504107445,1.13256090846417e-05,0.000113724655848509,2.97870773314628

"KIF9-AS1",0.709712683,-0.0552364356,4.86259415403383,1.1329169396076e-05,0.000113724655848509,2.97840462470708

"FLVCR1",-0.86440033615,0.14437703008,-4.86226232780504,1.1342177335424e-05,0.000113799475635733,2.9772980009187

"LDHA",-1.222596216725,0.18614574162,-4.86199075127169,1.13528343366693e-05,0.000113850646054665,2.97639232700739

"SORBS2",0.590405947166667,-0.0740059945,4.86108094206083,1.13886080865992e-05,0.00011415352371734,2.97335835896822

"DNAH6",0.938722880605,-0.106456903196,4.86043768472134,1.14139677827264e-05,0.000114314092522508,2.97121339250793

"CEROX1",1.201926304125,0.0475799633,4.85969481370782,1.14433236435097e-05,0.000114533861655216,2.96873638731046

"FCGR2A",-0.9421305222125,0.21125195373,-4.85898768919041,1.14713357914032e-05,0.000114702160904963,2.96637870050353

"KLHL13",0.761571771925,-0.05284561096,4.85751016403998,1.15300842433721e-05,0.000115192254954087,2.96145275060948

"CCDC9B",1.090119293625,0.0775365259,4.85747227027052,1.15315948316494e-05,0.000115192254954087,2.9613264229213

"UBTD2",0.710536796875,0.0318528353,4.85727016401245,1.15396548308412e-05,0.000115216592677521,2.96065266072397

"AC096631.2",-0.796228702025,-0.13772472302,-4.85677410220203,1.15594612402306e-05,0.000115301967813946,2.95899898085298

"ISCA2",-0.60357632445,0.06823261204,-4.85604109409294,1.15887894000435e-05,0.000115538256558682,2.95655552417288

"POGLUT3",0.619051825375,0.05051854625,4.85514253776528,1.16248409591358e-05,0.000115841313993568,2.95356039954096

"AP002414.2",1.2265889623875,-0.19905879129,4.85358866935667,1.16874447874871e-05,0.000116274075953267,2.94838141865076

"LAYN",0.6875762499,-0.06231178208,4.85336779943584,1.16963702500006e-05,0.000116274075953267,2.94764531659851

"DHODH",-0.60943900575,0.021605883,-4.85336099369247,1.16966453797191e-05,0.000116274075953267,2.94762263501619

"NKAPP1",0.66634565135,-0.02081290492,4.85101933316231,1.1791687836885e-05,0.000117105235289433,2.9398192361196

"ARL4C",-1.1583305012,0.11421571704,-4.85053283590127,1.18115285379322e-05,0.000117245444130405,2.9381981929195

"GSTO1",-0.7859405957,-0.02770372456,-4.8490144815992,1.18736622530119e-05,0.000117805129413903,2.93313930687593

"HCFC1R1",0.8126781608,0.07182391964,4.84854930912104,1.18927620663179e-05,0.000117937516560852,2.9315895495684

"HMGN2P30",-0.6920254503,0.07531345466,-4.84818284872583,1.19078300382017e-05,0.000118029812202553,2.93036869687885

"AL355864.1",-0.65100589165,-0.05931474732,-4.84741640239384,1.19394050975837e-05,0.000118285556885539,2.92781540994604

"SLC25A34",0.718214892875,0.0619932853,4.84593214922112,1.20007853478504e-05,0.000118836196329995,2.92287128556062

"NF1",0.593051188775,-0.01515748973,4.84466782175375,1.20533150076911e-05,0.000119298704288684,2.91866017736035

"TRIM2",0.807766958225,0.06758588938,4.84429495118969,1.20688499124897e-05,0.000119335072460925,2.91741832960676

"HOXA2",0.9332664303,-0.01052463676,4.8438502548089,1.20874030004521e-05,0.000119405358087398,2.91593731079114

"ITGAM",-0.899417171525,0.12220829978,-4.84313940524418,1.2117118384463e-05,0.000119583584518727,2.91356999653697

"CYP27A1",0.604427165225,-0.08831256482,4.84240698036448,1.21478106927131e-05,0.000119828765273137,2.91113096258598

"RHBDL3",1.0196263794,0.13180050952,4.8397560157586,1.22595389359967e-05,0.000120814542434199,2.90230415515371

"GMPPB",-0.805909695275,-0.03926520422,-4.83948056705399,1.22712057661968e-05,0.000120871376797038,2.90138710558027

"EIF4A1P2",-0.9244570223125,0.08251766205,-4.83881380658472,1.22994920368608e-05,0.000121091779361463,2.89916734277528

"CBLN4",1.1780784639,-0.15856948888,4.83786043318293,1.23400486442606e-05,0.000121432716918161,2.89599358814721

"ZNF555",0.597033877975,0.05512546528,4.83705426644151,1.2374445488e-05,0.000121712740834442,2.89331005790787

"SHROOM3",0.9770877290875,0.07717616137,4.83606531474253,1.24167698229945e-05,0.000122011884956121,2.8900183037086

"TACSTD2",-0.95023648105,0.03766761816,-4.8354201116964,1.24444592571573e-05,0.000122225350556395,2.88787085875931

"SLC6A10P",-0.593333425525,0.11388511748,-4.83208565146088,1.25885294264031e-05,0.000123581116860636,2.87677435648429

"HAGH",-0.898048948075,0.20150789154,-4.83149554587549,1.26141957550515e-05,0.000123773775205697,2.87481087842703

"PDE6H",-1.5615742434,-0.12611100692,-4.83064645563711,1.26512166400719e-05,0.000124077609632261,2.87198582547692

"LYPD3",0.81885793115,-0.05154481883,4.82640165625606,1.28378979480429e-05,0.000125753516648582,2.857865447093

"GGPS1",0.5942794031,0.06203479998,4.8263302055286,1.2841063294408e-05,0.000125753516648582,2.8576278041561

"DLX2",0.8359623378,-0.03660036176,4.82620606443973,1.28465647040047e-05,0.000125753516648582,2.85721491774466

"RPL7P32",-0.764940738975,-0.08286533498,-4.82620420052205,1.28466473226026e-05,0.000125753516648582,2.85720871848692

"MYO15A",0.739826565675,-0.03826674366,4.82521184636596,1.28907077943343e-05,0.000126124556490124,2.85390834336187

"MAPKAPK2",-0.683109281675,-0.07961139934,-4.82221732056012,1.30245656381481e-05,0.000127373413267006,2.84395064362986

"IFI44L",1.557962309,0.2210201232,4.82127004887221,1.30671928359451e-05,0.000127729316232273,2.84080115138918

"AL159987.1",0.955785209075,-0.08516177974,4.81996007736834,1.31263672022013e-05,0.000128246548039437,2.83644612713867

"ABCC13",-1.12095709105,-0.02618635009,-4.81576185937748,1.33177877743492e-05,0.000129992775569731,2.82249200036109

"COL5A1",1.0747369215625,-0.1161373174,4.81519400464858,1.33438889354155e-05,0.000130185522147045,2.82060489552821

"RHBDL2",1.97116976579167,0.0544849623,4.81453896432748,1.33740599004973e-05,0.00013041777212822,2.81842815559576

"ZNF510",0.868519770225,-0.10889130582,4.81354216378012,1.34201007398342e-05,0.000130742284335579,2.8151159339959

"PPP1R15B",-0.637997434725,-0.02311920278,-4.81311230565914,1.34400032614275e-05,0.000130873948108426,2.81368765653658

"ZNF365",1.828730415,-0.346508734,4.81141560654051,1.35188439884795e-05,0.000131579133318749,2.80805054053612

"CAPZA1",-0.622573061025,0.01674638528,-4.81119689774401,1.35290397226874e-05,0.000131612934522583,2.80732395530268

"F2RL3",-1.8008268336,0.39540172612,-4.81106558994854,1.35351646348635e-05,0.000131612934522583,2.80688773586967

"LINC01128",-0.705604396375,-0.0454730721,-4.80932265451076,1.36167228938896e-05,0.00013229851764297,2.80109792159923

"AL133163.1",-0.7736177629,-0.08036167472,-4.80928297582763,1.36185852064146e-05,0.00013229851764297,2.80096612296027

"S1PR4",-1.865373637325,0.20537317114,-4.80880299157441,1.36411329979448e-05,0.000132394287070242,2.79937181573247

"LRRC56",0.600311606475,0.02766291518,4.80753079264294,1.37010733297396e-05,0.00013284788943668,2.79514638609928

"TCTEX1D1",-1.28453997085,0.03864399782,-4.80685418253352,1.37330573813156e-05,0.000133095023476062,2.79289928889894

"PDE1A",0.656301970125,0.0404556651,4.80660391685017,1.37449062485351e-05,0.000133146874335691,2.79206815838428

"GPAT2",-1.525606312075,0.01428200734,-4.80636225945992,1.37563570747727e-05,0.000133157775806943,2.7912656311065

"C1QTNF3",1.39621672545,0.09658277536,4.80630587377687,1.37590302315159e-05,0.000133157775806943,2.791078380343

"AC024619.4",-0.624471780675,-0.01160812554,-4.80606725871065,1.3770348241215e-05,0.000133204388463651,2.7902859744574

"TNFAIP8",-0.975241892025,-0.02920598507,-4.8032605384423,1.39041644933639e-05,0.000134371944405679,2.78096635602486

"PRKG2",1.07507781625,-0.17609827,4.80259712935103,1.3935979876923e-05,0.000134571523457693,2.77876382290819

"IL6R",-0.9275795844,-0.05312443552,-4.80126914699539,1.3999881235651e-05,0.000135042168905846,2.77435522969388

"ATP2C2",-1.63793505675,0.043228136,-4.80007030007115,1.4057815580736e-05,0.000135533620407255,2.77037572368817

"NAALAD2",1.231916809825,-0.15857313414,4.79994135676955,1.40640607665865e-05,0.000135533620407255,2.76994772545146

"PLEKHG5",0.632062983225,0.12274128058,4.79889865881049,1.41146624744836e-05,0.000135806034852138,2.7664868809684

"SSC5D",1.615724854025,0.20446914472,4.79845966013324,1.41360202996318e-05,0.000135848436725584,2.765029872975

"SRD5A3",0.625706850025,-0.09024114598,4.79844955189477,1.41365124511394e-05,0.000135848436725584,2.76499632497219

"PPA1",-1.190307968125,0.1155023099,-4.79456127566914,1.43270778176528e-05,0.00013755068900097,2.75209355767468

"ABCG2",0.761136716625,0.0737534123,4.79410670307727,1.43495204184312e-05,0.000137672127976693,2.75058536777965

"SPATA20",0.62147862945,0.02800975776,4.79403292770991,1.43531660170937e-05,0.000137672127976693,2.75034059942211

"NIPBL-DT",0.83347878375,-0.102114176,4.79347781462598,1.43806260037317e-05,0.0001378709712515,2.74849891686101

"CENPV",-1.045430796625,-0.1354824653,-4.79266698475448,1.44208282774583e-05,0.000138191735149002,2.74580899253258

"DAB2IP",0.6439269355625,-0.02133537755,4.79238204533035,1.44349822071095e-05,0.000138262700074455,2.74486374747397

"KBTBD11",-1.32452417675,0.1554363986,-4.79112675773468,1.44974991457369e-05,0.000138731790050377,2.74069976239947

"PDE1C",0.99939179385,0.0136499231466667,4.79071150051201,1.45182385632421e-05,0.000138835995051808,2.73932237861233

"AZIN2",0.808020433875,-0.0655568704,4.7906374193504,1.45219414972679e-05,0.000138835995051808,2.73907666042395

"MFSD2A",-1.272528353875,0.3204000949,-4.79041864630914,1.45328822390974e-05,0.000138875788859434,2.73835102514882

"LINC01089",1.0089229248,-0.09826662176,4.7893438675921,1.45867489987082e-05,0.00013932555407251,2.7347863347206

"MTHFD2",-1.294832693275,0.07208758338,-4.78595350320427,1.47579571007233e-05,0.000140895165461146,2.72354355408598

"ITPK1-AS1",1.111959959625,-0.1661679093,4.78532595089781,1.47898630198949e-05,0.000141134007243413,2.72146285461786

"LRRC4C",0.949271116825,-0.09981452724,4.78492800327371,1.48101304620348e-05,0.000141199812231685,2.72014348042216

"KIAA0895",0.95603983095,-0.04592872824,4.78396439219536,1.48593200299439e-05,0.000141598952917902,2.71694884870217

"IL17D",0.9368339205,-0.1055055042,4.78311145410762,1.4902993747725e-05,0.000141949110136397,2.71412132829086

"KRT8P41",0.936936572675,-0.05988629286,4.78159597621728,1.49809028863564e-05,0.000142492450480813,2.70909792911133

"ENGASE",0.6679269204,0.08470829567,4.78131191992905,1.49955503200113e-05,0.000142565584666539,2.70815642555619

"COX7B",-0.65252261205,-0.01039327664,-4.78012712469884,1.50567962261817e-05,0.00014305871393694,2.70422965145935

"ABCB8",0.6555006023125,0.0082237587,4.77991029795604,1.50680312341626e-05,0.00014305871393694,2.70351106064422

"STRA6",1.29356717975,-0.000475942199999991,4.77990381889479,1.50683670771142e-05,0.00014305871393694,2.70348958841634

"TUB",0.879252711525,-0.08235890578,4.77975855947497,1.5075898545284e-05,0.00014306392283269,2.70300818768092

"BYSL",-0.923509167725,0.07762628422,-4.77859682463256,1.51362656380258e-05,0.000143503845623264,2.6991583072095

"FGL1",0.726818090075,-0.02448759194,4.77839943160286,1.51465462881578e-05,0.00014353489377973,2.69850419999869

"EPM2AIP1",0.941902475725,-0.11233203642,4.77482222394354,1.53340460459927e-05,0.000145043368139565,2.68665205298875

"SLC39A13",0.679356721275,0.05961343942,4.77419289523564,1.53672670514738e-05,0.000145290524850297,2.68456728011902

"TFDP1",-0.8426899434,0.16413945223,-4.77125904111849,1.55230733140343e-05,0.000146493194867773,2.6748496826005

"TMED5",-0.677569647275,-0.00180656432,-4.76790814106606,1.57029231930698e-05,0.000148122233139786,2.66375347855531

"RACK1",-0.713871548975,-0.00283313818,-4.76571882617845,1.58215304436224e-05,0.000149035179645487,2.65650534315361

"RPL23AP64",0.600226601575,-0.08299773274,4.76540637484291,1.58385291366033e-05,0.000149126739407504,2.65547101690946

"PSMG3-AS1",0.727713722975,0.06479505508,4.76431601423905,1.58979898576975e-05,0.000149617830135281,2.65186173145982

"HFM1",0.948452762225,-0.05336775902,4.76260838199083,1.59915524546613e-05,0.000150408282116657,2.64620979378747

"PRIMPOL",0.610673020875,-0.059408653,4.76251537719794,1.59966637413215e-05,0.000150408282116657,2.6459019875656

"EPHA3",0.792694336025,0.000503407419999999,4.76232582006891,1.60070862384773e-05,0.000150437239841249,2.64527464128519

"FAM122C",0.8111844084625,-0.09472173458,4.76082689186141,1.60897372824846e-05,0.000151025373195017,2.64031421513339

"PLEC",0.7372797748125,0.056138855,4.76066717505172,1.60985687155567e-05,0.000151025373195017,2.63978569658387

"CENPH",-0.819062018975,0.01307005122,-4.760656639166,1.60991514573368e-05,0.000151025373195017,2.63975083254255

"C12orf75",1.1520158635,-0.0800812252,4.75686784003723,1.63100574321098e-05,0.000152933847445797,2.62721530023039

"CYYR1",0.5865056702125,-0.01784702483,4.75576373778627,1.63720261664447e-05,0.000153444680740219,2.62356300334309

"SMAD7",0.68009231455,-0.06862251316,4.75484995302666,1.64234877131752e-05,0.000153716042150541,2.62054050671124

"ZNF204P",0.933630355625,-0.0441701808,4.75463051248063,1.64358695281842e-05,0.000153761687165953,2.61981470297157

"RPL29P7",-0.61344564275,-0.0228377322,-4.75422728689041,1.64586451819481e-05,0.000153904483107144,2.61848105980051

"LINC00028",0.6125882868,0.03047990744,4.75356208948052,1.64962856138685e-05,0.000154186085609917,2.61628105500154

"CCT8",-0.72512940915,0.00727487368,-4.75314007951855,1.6520208943581e-05,0.000154339279907017,2.61488540355624

"RELN",1.31574177615,-0.05120210578,4.75156111075171,1.66100212556071e-05,0.000154895819519745,2.60966392904939

"DBH-AS1",1.039658166125,0.2205165224,4.7502057605698,1.66874957658596e-05,0.00015547676819051,2.60518246107195

"COLCA2",0.839377182675,-0.00165237366,4.74700148963137,1.68720699712657e-05,0.000157009723668853,2.59458946298885

"PLAT",1.186002536325,-0.14507280274,4.74695055417398,1.6875020085846e-05,0.000157009723668853,2.59442109740205

"PLN",1.22783975665,-0.00968681168000001,4.74504508152801,1.69857466271361e-05,0.000157819913719873,2.58812310962622

"FGFBP3",0.7427332265,-0.1242500407,4.74218461472848,1.71533061035986e-05,0.00015923739712303,2.57867047153621

"SELENOK",-0.613131902025,-0.06555492492,-4.74085913549139,1.72314979663504e-05,0.000159890819897911,2.57429105688718

"LINC00924",0.640199922175,0.02365643634,4.74033908732211,1.72622716789743e-05,0.000160103857926132,2.57257293291193

"FASN",-1.721384981225,0.18690215602,-4.73957918151736,1.73073358071995e-05,0.000160414903533232,2.57006250164652

"ZSCAN23",1.234273718275,0.10172066062,4.73950960227196,1.73114677719629e-05,0.000160414903533232,2.56983264673048

"ADAMTS17",0.79535281435,0.09512958498,4.73836909559392,1.7379334781034e-05,0.000160970981461946,2.56606516837659

"MT1B",-1.349499821275,0.31756954298,-4.7368203552526,1.74719118660602e-05,0.00016175532323174,2.56094971049597

"GATA2",1.0692678322,0.04869195946,4.734777015679,1.75947939390257e-05,0.000162745886330817,2.55420157864398

"EBNA1BP2",-0.681983599075,0.04376649904,-4.73365667510327,1.76625279955449e-05,0.000163225021909212,2.55050212465898

"LINC01146",1.022459278,0.2518719264,4.73218804189213,1.77517065400509e-05,0.00016390129048786,2.54565308780113

"RBM43",0.6180350041625,0.00385473723,4.73014914374239,1.78762448538847e-05,0.000164976803858734,2.53892214242776

"PLCE1",0.7836256195375,0.05281914313,4.72993359535182,1.78894607511436e-05,0.000165024435780923,2.53821062476233

"GATA6",1.302402713175,0.06072327254,4.72905948062314,1.79431534957275e-05,0.000165445242493459,2.53532533010331

"MAP3K8",-1.0620970203625,-0.03311608359,-4.72757194468028,1.80348891479571e-05,0.000166008468815928,2.53041571289922

"NTNG1",1.09316556656,-0.174300907532,4.72743003483671,1.80436646431226e-05,0.000166008468815928,2.52994736994964

"PSD3",1.0828194201625,-0.02340470437,4.72674782678099,1.80859098359669e-05,0.000166312441974547,2.52769596411849

"EIF4E",-0.62421171005,0.00195666841,-4.72559286732258,1.81576508715511e-05,0.000166783859092444,2.52388468026854

"ZNF318",0.6538313255,0.0301984974,4.72392231164295,1.82619121999462e-05,0.000167404947271811,2.51837259842527

"TMEM131L",-0.9364930268,0.03083239396,-4.72342947508921,1.82927826280573e-05,0.000167612938498943,2.51674660264486

"LAMP3",1.15146269855,-0.12385478516,4.72201462689372,1.83816907131554e-05,0.000168352292950884,2.51207901334563

"PARVG",-0.954652682225,0.07929839222,-4.71994646278005,1.85124151625025e-05,0.0001694737988603,2.50525710026738

"ZNF789",0.7405440149,-0.14854853788,4.7178121006597,1.8648279084858e-05,0.00017056515262972,2.49821803716461

"GRID1",0.657661055,-0.07968079565,4.71754019924966,1.86656571265694e-05,0.000170647917540898,2.49732140244265

"AC138409.2",0.743790104875,-0.0965083495,4.7173777630301,1.86760464881923e-05,0.000170666744179341,2.49678575472149

"TBC1D32",0.81094839545,-0.00229509564,4.71355214216851,1.89223767480919e-05,0.000172686705930916,2.48417249023299

"SLC26A4",1.4321772324,-0.21323124308,4.71305974013444,1.89543126898149e-05,0.000172901139086788,2.48254930284393

"CCDC8",0.71411398415,-0.17504236768,4.71254031137094,1.89880587606699e-05,0.000173131886020741,2.4808370937015

"TARS1",-0.711509400575,0.05795513154,-4.7116509308425,1.90459764852673e-05,0.000173582725191351,2.47790557037945

"KCNT1",0.865734457525,0.12062346477,4.71032136080894,1.91328829408231e-05,0.000174065055813315,2.47352351937919

"EIF5A",-0.93477710905,0.14347516876,-4.70992217943235,1.91590509241014e-05,0.000174137456371113,2.47220797450318

"AMOTL2",0.9249158546,0.04181848518,4.70729837970303,1.93319267119302e-05,0.000175486271366427,2.46356203596382

"JPT1",-1.099162305,0.1170169107,-4.706104492099,1.94110941415296e-05,0.000176126880766899,2.45962855798656

"SNTB1",-0.825577148425,-0.00348926694000001,-4.70468536420169,1.95056106660849e-05,0.000176906131618746,2.45495348822694

"COL12A1",1.31184054365,0.03732660827,4.70356710449564,1.95804063332369e-05,0.000177505913697061,2.45126996149455

"FANCD2",-0.8626428627,-0.07923379716,-4.7027619073771,1.96344365080764e-05,0.000177916999193928,2.44861786749037

"SNHG17",-0.592272469666667,-0.0136239873666667,-4.70171169204196,1.97051274013962e-05,0.000178478625198853,2.44515901687746

"CR1L",-2.2147009746,0.28970481832,-4.70074461679428,1.97704425545477e-05,0.000178991085752352,2.44197425078219

"AC091951.1",1.0067636813,0.00434416504,4.69858759593223,1.9916890363475e-05,0.000179998786840263,2.43487168047736

"FKBP5",-1.740981687725,0.31976167622,-4.69772884137602,1.99754895773388e-05,0.000180448778862662,2.43204435330801

"IFI44",1.07925127195,0.14941995356,4.69481021440901,2.01759145210064e-05,0.000182018554252038,2.42243669286232

"ZNF440",0.646454194375,0.0173202325,4.69378837088498,2.0246550051643e-05,0.000182575403810767,2.41907349451544

"CD2",1.06543556975,-0.08464960195,4.69268871112441,2.03228353068645e-05,0.00018318268797494,2.41545449800798

"GAREM1",0.61588738295,0.000348263859999995,4.69248492966872,2.03370028616652e-05,0.000183195029080371,2.41478388654054

"KIAA0895L",0.745268872475,0.01525710798,4.68687085072149,2.07311393129272e-05,0.000186289290457567,2.39631334152789

"FAM199X",-0.690209207475,0.07781624572,-4.68537166583507,2.08376491922832e-05,0.000187111341177326,2.39138242511176

"AL589843.2",-0.764497166725,0.15664337962,-4.68532621798259,2.08408864001688e-05,0.000187111341177326,2.39123295381257

"MAPK1",-0.6046153089,0.10050012558,-4.68395131616947,2.09390525661039e-05,0.000187910341206455,2.38671137233347

"CLEC18C",0.75260114845,0.03242273276,4.68322500278364,2.09910930496769e-05,0.000188294883713564,2.38432298594183

"EIF2AK1",-0.71028770435,0.12002399132,-4.67911644099223,2.12878660152089e-05,0.000190706514612856,2.37081525227419

"NXT1",-0.6261308202,0.01379928654,-4.67518594866034,2.15756206880339e-05,0.000193031142644733,2.35789731370847

"ATG4C",0.633732003766666,0.00844955594666666,4.67484996955072,2.16003939896632e-05,0.000193168429533051,2.35679328418549

"LZTFL1",0.616231743925,-0.01072796786,4.67354118966347,2.16971623007928e-05,0.00019384084637323,2.35249292257723

"HSD17B10",-0.663937137175,-0.01360429594,-4.6734493552414,2.17039682536211e-05,0.00019384084637323,2.35219119273737

"ADAM32",0.7138531577625,0.11741167926,4.6732433105624,2.17192460662874e-05,0.000193892772725968,2.35151422382995

"COL24A1",1.6618239654,0.10082749732,4.67286839090477,2.17470726519615e-05,0.000194056630876911,2.35028243884166

"HPX",0.802635776175,0.07049874194,4.67224780659599,2.17932092415564e-05,0.000194383661707012,2.34824361700244

"FRK",0.726732857425,-0.12132053406,4.67115278585335,2.1874851124464e-05,0.000194942126941287,2.34464637680706

"QPCT",-0.932952400225,0.12307305737,-4.67029154745444,2.19392731551085e-05,0.00019534629656752,2.34181736567385

"LINC01704",0.843216591075,0.04279866286,4.66934070657407,2.20106133932317e-05,0.000195896371503271,2.33869426549346

"METRNL",-0.819632631125,0.1252817353,-4.66912528623953,2.20268076097005e-05,0.000195955377467453,2.33798673789138

"CCDC74B",0.9626609732375,0.01771572409,4.66695929409245,2.21902857942833e-05,0.000197324034441526,2.33087345823444

"CBLN2",1.8936050989,-0.08914122188,4.66672919689974,2.22077220238617e-05,0.000197393409468494,2.33011787842643

"C8orf48",0.93149879405,0.03192562954,4.66523490849035,2.23212826240826e-05,0.000198169543760581,2.32521137878566

"AFDN",0.68790609646,0.015390746268,4.66505873126025,2.23347088804238e-05,0.000198169543760581,2.32463294131511

"LUM",1.655391864675,0.09565212174,4.66498635005852,2.23402272568894e-05,0.000198169543760581,2.32439529676463

"ANKRD29",0.92529550605,0.03768184944,4.66494470022483,2.23434032646888e-05,0.000198169543760581,2.32425855120113

"ZNF772",0.7989012021875,-0.03564319085,4.66461930217106,2.23682317158838e-05,0.000198303908003041,2.32319021443474

"MEOX1",1.261545609,0.1004236308,4.66431689339619,2.23913302626302e-05,0.000198422826306561,2.32219738175154

"RNF180",0.709815157275,-0.05637124418,4.66370850803702,2.2437870651667e-05,0.000198663394084423,2.32020008004027

"EHF",0.946963728875,-0.1241766819,4.66356396645246,2.24489417409025e-05,0.000198675558698752,2.31972557167215

"TP53I11",0.8311111801,0.11988636168,4.66317049152179,2.24791068589188e-05,0.000198856624061109,2.31843388187686

"STIP1P3",-0.68327635325,0.0144998171,-4.66215743427897,2.25569540025328e-05,0.000199373111994776,2.31510844087058

"ESPNL",0.75391164995,0.01169312296,4.6605214807535,2.26832245037414e-05,0.000200230031724539,2.30973889677865

"DHRS3",-1.2385665194,0.02332334388,-4.66029615945431,2.27006699844152e-05,0.000200297728958096,2.30899940324835

"TWNK",-0.78859244315,0.07080193548,-4.65951821040384,2.2761003653577e-05,0.000200657247355631,2.30644632116613

"FNDC1",2.2486894553,-0.07096690806,4.65710295133664,2.29493206407625e-05,0.00020205658843487,2.29852097233028

"CMIP",-0.8922286399875,-0.10276993359,-4.65603238024547,2.30332798645356e-05,0.000202621656446804,2.29500855788615

"PDGFD",1.186519329825,0.03318187886,4.65568354661843,2.30607019410141e-05,0.000202775820329398,2.29386414607621

"C21orf62-AS1",0.655244724925,-0.04421828006,4.65553637544475,2.30722807634665e-05,0.000202790599863536,2.29338133462427

"HAPLN1",2.0691069284,0.17045280872,4.65387402399006,2.32034636238371e-05,0.000203681475032208,2.28792822588351

"FOXF2",0.76500513925,-0.1058102884,4.65151790386599,2.33906462820249e-05,0.000205148784685842,2.28020063225048

"RIOK1",-0.70816110575,0.0267018419,-4.65066815469457,2.34585169097352e-05,0.000205656009604901,2.27741400983958

"WNK2",0.752086241675,-0.04942300651,4.64935994685158,2.35633822916478e-05,0.000206310502731316,2.27312433817566

"DNAH10",-0.636389534916667,0.0611714227333333,-4.64919871523713,2.35763382704588e-05,0.000206335761847569,2.27259568577957

"NDUFS2",-0.8056759395,-0.0018958646,-4.6468631625066,2.37647986808808e-05,0.000207896325949567,2.26493860459599

"VASN",0.6559669053,-0.07699211176,4.64379073770669,2.40149661636781e-05,0.000209726609872735,2.25486803237399

"KDM5A",0.869127071675,-0.05269385066,4.64305830067153,2.40749831808693e-05,0.000210071659033071,2.25246769525042

"POLR1E",-0.68743061885,0.09011068292,-4.64223593499128,2.414254405971e-05,0.000210571495400315,2.24977282472075

"VARS1",-0.5856148592,0.05489202674,-4.64184325939174,2.41748694777553e-05,0.000210763713132022,2.24848610441195

"DESI1",-0.741616392825,0.09170845034,-4.64043846517751,2.42908606872807e-05,0.000211684880374737,2.24388322742811

"ALDH1A2",1.356448185675,-0.08505134746,4.63836243007051,2.44632729435279e-05,0.000213096741525084,2.23708201620432

"CCNO",0.8691493254,-0.05511449718,4.63641228038435,2.46263212672332e-05,0.000214425869155578,2.23069432169581

"RIN2",0.6858708752,-0.03918075384,4.63408438442643,2.48223452032584e-05,0.000215857473906774,2.22307073141203

"SPTAN1",0.6746671110625,0.10075978035,4.6328371964063,2.49279939206348e-05,0.000216592340731962,2.21898696588013

"AC092573.1",-0.83038006505,-0.07817943564,-4.63270580685905,2.49391494573036e-05,0.000216597411649528,2.21855677243971

"METTL21A",-0.662795303,0.02850287555,-4.63205421986776,2.49945442239569e-05,0.000216986534771368,2.21642342959334

"AMDHD1",0.8545930678,-0.09404018576,4.63145746759278,2.50453828402016e-05,0.000217335791457031,2.21446972515852

"HIRA",-0.690262729575,-0.10447248426,-4.630934738698,2.50899983809904e-05,0.00021761125176688,2.21275844849116

"MKRN9P",1.605564223875,-0.0368489355,4.63083676519859,2.50983691880681e-05,0.00021761125176688,2.21243771769398

"USP40",0.85075475135,-0.01734904152,4.6291339303104,2.52442958751627e-05,0.000218691388537139,2.20686366996184

"SLC10A5",0.926528759825,0.14198007586,4.6279021964647,2.53503681199005e-05,0.000219517473389908,2.20283223425039

"LIG4",0.925856450425,-0.14950258216,4.62719347868329,2.54115977526643e-05,0.000219954716838439,2.20051281312162

"ANO3",1.257543573575,0.12360206886,4.62409459031899,2.56810302278695e-05,0.000222192967613425,2.19037275728788

"GPR68",1.209496901875,-0.0622147175,4.62138164431768,2.59192003678517e-05,0.000224064378538627,2.18149781149522

"RRAGD",-0.800178710625,-0.0672448625,-4.61945238191265,2.60898843606227e-05,0.000225159878172047,2.17518783181098

"SNAP91",0.813866188833333,0.0277293086,4.61794204571031,2.62242725241024e-05,0.000226129164761705,2.17024876303488

"C1QTNF1-AS1",1.234068425025,0.19622833502,4.61724461338732,2.62865577010528e-05,0.000226570885224724,2.16796825497229

"XCL1",1.1041905921,0.00540753468,4.61620755985121,2.63794409412193e-05,0.00022711015136742,2.1645774893167

"PRR34",0.8047786241,-0.00676041272,4.61617482562217,2.63823779897725e-05,0.00022711015136742,2.16447046600396

"CIITA",0.7226502561375,-0.13828025409,4.61550939833999,2.64421522453271e-05,0.000227503862138377,2.16229494337172

"GBP3",0.943336416675,-0.00846409486,4.61541856286061,2.645032212093e-05,0.000227503862138377,2.16199797914336

"RERGL",1.6003149009,-0.14201211878,4.61350610171967,2.6622906175693e-05,0.00022879618361057,2.15574620702519

"LEF1-AS1",0.7382594991,0.06321922408,4.61258623271954,2.67063087881728e-05,0.000229416710462089,2.15273955899526

"CCDC158",0.90021660165,0.07889708967,4.61165137014506,2.67913327289013e-05,0.000230050639123944,2.14968415214452

"TNNT3",2.0847940796,0.25542283818,4.61151011303913,2.68042027982128e-05,0.000230064728500119,2.14922250402751

"FAM49B",-0.734864617275,-0.00861398782,-4.6108068689537,2.6868366103434e-05,0.000230518879701489,2.14692428851962

"C9",-1.317271029125,-0.0752234628,-4.61001582857769,2.69407194376057e-05,0.000230967725083742,2.14433932265475

"ZSWIM9",0.65544437835,-0.02228259872,4.60998843561539,2.69432283751534e-05,0.000230967725083742,2.14424981100833

"CPXM2",1.0226016466125,-0.05015741666,4.60607376924701,2.73041353296699e-05,0.000233572912164625,2.13146012129964

"FOLH1B",1.534779024,0.1091583287,4.60578976950208,2.73305014898634e-05,0.000233700882522671,2.13053243118254

"FN1",1.71118829375,0.088168507,4.60473759144533,2.74284015518811e-05,0.000234440171462219,2.12709567561401

"RPS13",-0.64050519745,-0.05117731006,-4.60423515125063,2.74752721122211e-05,0.000234703459017011,2.1254546550963

"EXD3",0.8009855408,0.00394649044,4.60416185380478,2.74821162720523e-05,0.000234703459017011,2.12521526430045

"DIMT1",-0.732373076925,0.08187269206,-4.6020171451391,2.76831187464647e-05,0.00023622313072785,2.11821129229618

"EIF3I",-0.706846571525,-0.05914466882,-4.60187087654315,2.76968793252623e-05,0.000236242158041621,2.1177336714738

"ARVCF",1.002310627075,-0.05736124959,4.59999736088096,2.78737277332587e-05,0.000237437419416638,2.11161649910756

"AC073107.1",0.99428573235,-0.07743756442,4.59945366385167,2.79252558562339e-05,0.000237695322801214,2.10984147635531

"AC138761.1",0.8855227334,-0.05921373308,4.59800936198899,2.80625904655415e-05,0.00023856695164233,2.10512663894242

"AP001830.1",0.671007319025,-0.08264601178,4.59541327361605,2.83111080304072e-05,0.000240480091760772,2.09665338005424

"HMGB3",-1.00667747775,0.1168738543,-4.59448829024864,2.84001736350162e-05,0.000241136658696317,2.09363483892547

"EPG5",0.589250902025,-0.10747218838,4.59302796547416,2.85413448016062e-05,0.000242134605505303,2.0888697980929

"CPT1C",0.791385428425,-0.00283886476,4.59218475733307,2.86231709521229e-05,0.000242728280822472,2.08611869092505

"OLIG1",-1.11571324565,0.12960275908,-4.59153137907793,2.86867334610725e-05,0.000243066085670163,2.08398707727514

"INSIG1",-0.95630386515,0.02072942588,-4.59003650491863,2.88326787769571e-05,0.000244201704333318,2.07911059178024

"P2RX7",0.951292580425,-0.14755085666,4.5896233660349,2.88731416472091e-05,0.000244443357879347,2.07776299029481

"ZNF699",0.74656076925,0.0983931654,4.58835160379838,2.89980470912747e-05,0.000245399417102865,2.07361499043679

"IGLV3-9",-2.674740565525,0.10917864458,-4.58810210447485,2.90226134632045e-05,0.000245505906124746,2.07280127466804

"SCNN1D",0.784786019825,0.07197019886,4.58686943192005,2.91442846991386e-05,0.000246433390390405,2.06878130868944

"MMRN1",-1.828475770975,-0.16709884278,-4.58507416710237,2.93223794720556e-05,0.000247734808504526,2.06292741974734

"BATF",-1.004943236775,0.04623214958,-4.58482257321212,2.93474230894674e-05,0.00024775208502383,2.06210711288551

"RPL7AP26",-0.70854068205,0.00295362436,-4.58377956090397,2.94514676548423e-05,0.000248517985713513,2.05870663027852

"GABBR1",0.6638106350125,0.03445586821,4.58290973519327,2.95385119460235e-05,0.0002490473385803,2.05587102212242

"RHEBL1",0.602942649975,-0.05928696612,4.58218381911509,2.96113475995564e-05,0.000249558736988775,2.05350472441117

"PTPN14",0.938957353275,-0.02217910688,4.58070876752225,2.97598900588587e-05,0.000250707494870845,2.04869691347353

"HSPB11",0.611092701591667,0.04992826854,4.58032731180221,2.97984223075015e-05,0.000250928925703284,2.04745369292164

"FRAS1",1.00081111045,0.00156612471000001,4.57986021923894,2.98456714954493e-05,0.000251223548725869,2.04593142714422

"RRP1",-0.714985853375,0.0885579123,-4.57927244367181,2.99052325908543e-05,0.000251621521692576,2.04401594318501

"DNMT1",-0.642885993125,0.029851858,-4.57902676953364,2.99301619300006e-05,0.000251727897217509,2.04321535311824

"UQCRHL",-0.69108054665,-0.00538890932,-4.57630400669984,3.02078135291788e-05,0.000253542680698818,2.03434373772864

"ORC1",-1.354343082825,0.03531968924,-4.57606416609398,3.02323914296551e-05,0.000253645059627672,2.03356236578972

"SRSF3",-0.6057551911,-0.03424623598,-4.5752719217248,3.03137164894094e-05,0.000254223259695056,2.03098144836881

"TRIB3",-0.785962759325,-0.00152450746,-4.57508072775226,3.03333748504664e-05,0.000254284035980505,2.03035861777229

"BTN2A2",0.77374990735,-0.11357138662,4.57287408396425,3.05611636618552e-05,0.000256088802087562,2.02317106387544

"EXTL2",0.831887994025,0.06551669422,4.56631563947007,3.12480998526777e-05,0.000261204026838702,2.00181711985874

"DLEU2L",0.9819795569,-0.02153449448,4.5638771879352,3.15073354957892e-05,0.000263156253419376,1.99388088041618

"APCDD1",1.118696220275,0.12621841122,4.5636565313388,3.15308973116526e-05,0.000263245731100708,1.99316281296374

"THORLNC",1.425213397325,-0.14614977814,4.56352011890847,3.15454721103324e-05,0.000263260135477186,1.99271890263981

"PCDHB18P",0.690450925775,-0.16055492478,4.56238645205678,3.16668522509355e-05,0.000264165500373439,1.98902996137525

"NOSTRIN",0.941574860825,0.13021644466,4.56105852241244,3.18096125172911e-05,0.000265140496848763,1.98470937328984

"CDK6-AS1",-0.74825268195,-0.02714992971,-4.5607562873375,3.18421923521067e-05,0.000265304122370867,1.98372608577193

"LINC00886",1.088955322525,-0.17136404298,4.56060085144613,3.18589604711744e-05,0.000265335927696513,1.98322040324775

"PAMR1",1.63504098965,0.17450325672,4.56036680581225,3.18842251394593e-05,0.000265438441551094,1.98245899163019

"DDX60",0.664382841525,-0.01239498228,4.56022195582317,3.18998711605504e-05,0.000265460828731664,1.98198776486715

"PTPRZ1",1.510649547325,0.00906499786,4.55894234473387,3.20384151779775e-05,0.000266397341788313,1.97782519661411

"ADGRB2",0.6093957098,0.08816411004,4.55670483056436,3.22820864598009e-05,0.000268314558778256,1.97054773861483

"BNC2",0.736898043241667,0.00741288019333333,4.55535056518823,3.24304485948416e-05,0.000269275739121658,1.96614374503008

"PELI1",-0.620182039275,0.01747951658,-4.55528945043534,3.2437159515436e-05,0.000269275739121658,1.9659450161628

"GNA15",-0.8023289032,0.00542691343999999,-4.5498300792923,3.30421531129999e-05,0.000274075964768883,1.94819706905969

"ANKFN1",0.799928965075,0.04126987406,4.54962852428847,3.30646987359433e-05,0.000274151982072847,1.94754200048595

"ERICH2",0.757993577225,-0.18642702122,4.54903839721561,3.31307959005583e-05,0.00027458889417906,1.945624113599

"NDNF",0.94454360215,-0.06287007178,4.54875881511865,3.31621555491521e-05,0.000274737663926821,1.94471552031033

"AC124804.1",1.1999355936,-0.11187411312,4.54844908923811,3.31969301803171e-05,0.000274914593991244,1.9437089921075

"PRDM5",0.8417175536375,0.06276951906,4.5476364136188,3.3288343268273e-05,0.000275448940581736,1.9410681443564

"AC023813.3",-0.96898091825,0.1217535777,-4.54660710951855,3.34044767842901e-05,0.000276298312618706,1.93772362902363

"BTBD16",0.975282825875,0.0573641567,4.54638435178715,3.34296619869962e-05,0.000276365379921954,1.93699986439547

"CPE",1.103855401,0.0760402858,4.54629682000336,3.34395634921185e-05,0.000276365379921954,1.93671546794647

"NYX",-0.765553448225,0.11928283542,-4.5460861084966,3.34634107158641e-05,0.00027645095110751,1.93603086194075

"SYTL2",1.197776855475,0.17148314708,4.54578050687179,3.34980266400933e-05,0.000276625380815087,1.9350379795027

"EMB",-1.270447971925,0.26227491906,-4.54549681079108,3.35301926124328e-05,0.000276779446512298,1.93411629185193

"HIP1",0.629633098625,-0.0453313161,4.54362223210329,3.37434956725893e-05,0.000278315917608699,1.92802665917381

"C2CD5",0.6464958089,-0.04498863308,4.54258789966393,3.38617565550102e-05,0.000279178940965412,1.92466704383114

"CD82",-0.84067763,0.109030725,-4.54163343561397,3.39712449063888e-05,0.000279856399534416,1.92156713236735

"AC007346.1",-1.221557195075,0.24595648194,-4.54008224757632,3.41499229732838e-05,0.000281102298865664,1.91652975935264

"PCDHGB6",1.253834909225,0.02894989368,4.53953576548758,3.42130895798597e-05,0.000281396443923908,1.91475526890056

"ATL3",-0.6433081427,-0.00960586476,-4.53953544379748,3.42131267968789e-05,0.000281396443923908,1.91475422436197

"BTF3P11",-0.73703362805,-0.06266843944,-4.53769745860121,3.44264143500137e-05,0.000283037069503644,1.90878672985542

"GSTM5",0.7274136313,0.03501518304,4.53741623043537,3.44591636514943e-05,0.000283192677453596,1.90787373903508

"GP9",-1.325840885275,0.10476241278,-4.53683257132134,3.45272285166757e-05,0.000283547734621669,1.90597899937904

"SENP8",0.614525532875,0.0017741963,4.53629263512505,3.45903114879303e-05,0.000283928806796761,1.90422628881682

"ARHGEF25",0.956368790125,0.0308964161,4.53598756984788,3.46260032966311e-05,0.000284107951758661,1.9032360416601

"H3C11",-0.90708134565,-0.07420318652,-4.53359587605698,3.49070746932022e-05,0.000286070458525731,1.89547352911333

"RERG",0.651965470725,0.03735550188,4.53344290223186,3.49251278026973e-05,0.000286103965782352,1.89497709365275

"DENND2C",0.704159279058333,0.0762343836133333,4.53191998850957,3.51053516976627e-05,0.000287348367322836,1.89003526928911

"GAPDHP72",-0.8019832216,-0.12086990388,-4.53180404893446,3.51191093213775e-05,0.000287348367322836,1.88965907623051

"HTRA1",1.33467377395,0.17289924486,4.53093956178279,3.52218573148379e-05,0.000288074017032495,1.88685417286153

"LINC01554",-0.936501187375,0.0434581371,-4.52821827329173,3.55472151777175e-05,0.000290387298469328,1.87802618366651

"AL109918.1",1.18682416385,-0.14101665382,4.52763771008932,3.56170065250019e-05,0.000290841462608306,1.87614309742288

"SLC45A1",0.733776731425,-0.12298909186,4.52720853117197,3.56686855270157e-05,0.000291147421943226,1.87475109903822

"KCNJ15",-0.968819206625,-0.019333367,-4.52549099328199,3.58762347204555e-05,0.000292724929093067,1.86918099405618

"ICE2",0.838965924125,-0.100284365,4.52486458325725,3.59522240400878e-05,0.000293228171231417,1.86714972034414

"COL5A2",1.728675831725,-0.06675520162,4.51939722875285,3.66221742337462e-05,0.00029809896928923,1.8494256365582

"SYCE2",-0.6616571912,-0.06191270796,-4.51908838951813,3.66603800204969e-05,0.00029829144791896,1.84842471029441

"FGF5",0.687305569525,0.12122158962,4.51760000885441,3.68450507640492e-05,0.000299675029795094,1.8436013778688

"KLF1",-1.99404814565,0.34009562558,-4.51630305686011,3.70067106408351e-05,0.000300525134780689,1.83939894717427

"MKNK2",-0.7136778292875,-0.07505775143,-4.51629077698074,3.70082445847168e-05,0.000300525134780689,1.83935915990095

"C1GALT1C1L",0.59550350405,-0.07584296276,4.51599815115714,3.70448163838848e-05,0.000300702931090742,1.83841105469144

"SYAP1",-0.59836707845,-0.00781085876,-4.51581752937601,3.7067407755098e-05,0.000300767148549089,1.83782585459159

"PHACTR2-AS1",0.8688448239,0.01542917212,4.51560373678654,3.70941653750989e-05,0.000300865106969528,1.83713319651003

"ARHGEF33",0.877126489675,-0.00129129436,4.51306197270358,3.74137337888084e-05,0.00030321700073778,1.82889929854838

"TEKT2",0.71929530085,-0.01680624032,4.51293041286367,3.74303474040909e-05,0.000303231695379603,1.82847317140587

"PDZK1IP1",-1.611481479375,0.1290245535,-4.51127451571612,3.76400742032271e-05,0.000304329060882287,1.82311010930693

"CHAD",1.744289950975,-0.07764484322,4.50927904372872,3.78943349357022e-05,0.000306110411083124,1.81664835119819

"GABARAPL1",-0.98132384545,-0.00050974936,-4.50919392161646,3.79052183189128e-05,0.000306110411083124,1.8163727348212

"B3GNT5",-0.862731011875,0.1037512495,-4.50682200971005,3.82097124522612e-05,0.000308326344514347,1.80869362241297

"PITHD1",-0.9786830451,-0.20733026558,-4.5061676453392,3.82941360303909e-05,0.000308885928736476,1.80657540618017

"GVQW3",1.09923769035,-0.13756137072,4.50552957304225,3.83766330754481e-05,0.000309429538941275,1.80451005402032

"FCN1",-1.958349910025,0.17390210498,-4.50497915404445,3.84479367481431e-05,0.000309882505151831,1.80272852323743

"WTIP",0.675316161525,0.04382491932,4.50159470035877,3.88892256762991e-05,0.000312946761844468,1.79177615763594

"IL25",1.044396102025,-0.04012675538,4.50009704985353,3.90860748758301e-05,0.000314407342778173,1.78693075623151

"CHSY1",-0.7091223424,-0.00836681392,-4.49915921098727,3.92098382370498e-05,0.000315279107457094,1.78389688233976

"VIT",0.78291539745,0.0356070936266667,4.49827123020181,3.93273746492112e-05,0.000315890292728443,1.78102454551611

"NOG",1.281730459375,-0.2962378005,4.49716475993736,3.94743126406738e-05,0.00031690819646635,1.77744580215917

"COL1A1",2.212411781575,-0.37896821974,4.49652929779954,3.9558943489919e-05,0.000317463233145891,1.7753906467594

"LINC02731",0.5972516993,-0.09622257621,4.4962332865427,3.95984267309135e-05,0.000317655664394266,1.77443335559448

"LTC4S",0.853797129225,-0.08788457862,4.49550840988923,3.96952764199924e-05,0.000318307954322037,1.7720892405291

"CRISPLD1",1.435660501175,0.09025954994,4.49533042861933,3.97190915757145e-05,0.000318374314633505,1.77151370713367

"NIPAL2",0.5947319809,-0.00602936418,4.49464140493437,3.98114194455389e-05,0.00031898958216668,1.76928572079072

"SLC46A1",0.785209992,-0.1344487864,4.4936712001147,3.99417804178607e-05,0.000319908990305368,1.76614877062647

"ATM",0.592331821941667,0.0209406488866667,4.49333894307083,3.99865196636837e-05,0.00031993055994493,1.76507455480567

"GSTA4",0.729491890575,-0.10979652404,4.49292154723161,4.00427924088981e-05,0.000320178361600198,1.76372512590633

"DNAJC6",-1.00274751865,0.12644827048,-4.49248707411692,4.01014496191053e-05,0.000320436232369825,1.76232054348008

"NCAM1",1.088938560075,-0.07731206339,4.49172651828585,4.02043325341689e-05,0.000320910249189391,1.75986192710733

"LINC00487",0.825693479625,0.0897220574,4.49087951273267,4.0319213114855e-05,0.000321549255857201,1.75712405758518

"PHLDB3",0.7807789516875,-0.0265986344,4.48956611506532,4.04979851942958e-05,0.000322723734212654,1.75287905350575

"SGCB",0.617611332825,0.08423198726,4.48840862327618,4.0656177637475e-05,0.000323858385473013,1.74913838931302

"GBP1P1",1.161100211775,0.10552531042,4.48692651504938,4.08596158688725e-05,0.000325352432927112,1.74434926489528

"USP9X",0.6675038364,-0.00543227188,4.48373782798194,4.13006786623111e-05,0.00032860905026541,1.73404798085011

"IRS2",-1.60393040405,-0.34634660524,-4.48299387437673,4.14042499488958e-05,0.000329230326693889,1.73164503621542

"TRMT9B",0.9538357885,0.1611882708,4.48294615763402,4.14109015955756e-05,0.000329230326693889,1.73149091860354

"SUGCT",0.696918202325,0.01956854926,4.48261665997583,4.1456861585343e-05,0.000329403192035611,1.7304267119186

"FNDC11",0.858073132775,0.00804126102,4.48151023298661,4.16115562104062e-05,0.00033044091613907,1.72685343566751

"PARD6G",1.104920793475,0.05249794968,4.48128916732475,4.16425318833306e-05,0.000330558773043657,1.72613953540169

"MPHOSPH6",-0.6720905235,-0.0120500952,-4.47739171832565,4.2192352612154e-05,0.000334165004498976,1.71355575751021

"SRGAP3",1.470877183475,-0.04627740722,4.4731124626593,4.28041975515051e-05,0.000338599382021327,1.69974465102654

"SDHB",-0.755278292625,-0.0914491271,-4.47286313112081,4.28401125475141e-05,0.000338752692347923,1.69894011976296

"PREPL",0.6354970488,0.04836090594,4.46973350705883,4.32934288892536e-05,0.000341677877921043,1.6888432388588

"UBA7",0.936880072575,0.04779048986,4.46915494228473,4.33777434143348e-05,0.000342211477502076,1.68697699045612

"L1TD1",1.25867195365,0.17388770012,4.46859131150804,4.34600356960252e-05,0.000342728718760648,1.68515901407703

"ENY2",-0.6289674,0.0012213698,-4.46802546282803,4.35428050570764e-05,0.000343249322820077,1.68333398336541

"CENPL",-0.7294885263,0.17330567056,-4.46680384273443,4.37220221301063e-05,0.000344529534385238,1.67939423426771

"PPIAP80",-0.648478671025,-0.02886814182,-4.46651807773889,4.37640488992661e-05,0.000344728117588682,1.67847270356171

"BNC2-AS1",0.698303697925,-0.01943650166,4.46563489628691,4.38941856262159e-05,0.000345620320949236,1.67562479401539

"MT2A",-1.2717195723,0.19268808416,-4.46458504647117,4.40493722510093e-05,0.000346709004486622,1.67223976150121

"BASP1",-1.608339582275,-0.03347553082,-4.46299910533308,4.42848186182021e-05,0.000348428327131231,1.66712686086326

"MYO1D",0.871259643325,-0.03198628034,4.45832157704711,4.49864123114164e-05,0.000353541087624203,1.65205159761888

"HCST",-1.16404244435,-0.01140268548,-4.45763465838218,4.50903540178862e-05,0.000354086306293006,1.64983829532127

"TANC1",0.711207628875,0.0273612991,4.45555807309503,4.54060013694702e-05,0.000356291901975375,1.64314828398776

"PSMC4",-0.660093377925,-0.00105443784,-4.45401994687871,4.56411901574721e-05,0.000358000269504705,1.63819386561722

"CYBA",-0.778557427525,0.11279745268,-4.45372162880883,4.56869422130635e-05,0.000358221994665612,1.63723304696233

"SLC2A10",0.8055013862,0.06639789676,4.45324596979348,4.57599850355383e-05,0.000358657449658802,1.63570110884378

"MSANTD2",0.681756907575,-0.05267845224,4.45283129194693,4.58237564643469e-05,0.000358882692064809,1.63436562855638

"RCSD1",-0.75727779185,-0.000171998680000009,-4.45174238507685,4.59916276031829e-05,0.000359922256048132,1.63085903403117

"GIPC3",0.81404220725,0.0452625003,4.44890972536113,4.64311396620656e-05,0.000362807471165675,1.62173879728914

"SAT1",-0.772774938958333,0.075137335,-4.44580830396456,4.69170520090118e-05,0.000365906570826279,1.61175613143394

"DNM1P46",0.6058854095,-0.0354594054,4.4434088028883,4.72963920215738e-05,0.000368584435046788,1.60403484369586

"BSN",1.058481572175,0.03995232774,4.4414420144283,4.76095538079938e-05,0.000370872807106458,1.59770732484425

"SYNPO2",0.9695979941875,-0.19173263735,4.43839127690576,4.80993132596014e-05,0.000373988132851125,1.58789496179426

"FOLH1",1.10749152655,0.18723991194,4.43692107476901,4.83370880772775e-05,0.000375551862164301,1.58316726910941

"CCNE2",-1.6482186825,0.040512174,-4.43670209122387,4.83726021015614e-05,0.00037568531912691,1.58246314769969

"CRYZL2P",0.9347340459,0.15780048472,4.43516218103165,4.86230593705105e-05,0.000377454370857142,1.57751213746924

"NCF4",-1.042752797375,0.1801981411,-4.43507529454878,4.8637228575891e-05,0.000377454370857142,1.57723280860049

"MAPKBP1",0.62121280205,0.16068961094,4.431922024938,4.91541890857863e-05,0.00038117752686964,1.56709707287358

"E2F2",-2.564082895625,0.5684205025,-4.43085966163744,4.93295613874936e-05,0.000382248129238642,1.56368296837425

"AC078925.2",1.19967869985,0.21566856518,4.43013656495793,4.94492772721809e-05,0.000382959102860392,1.56135936616719

"CCN5",1.3843351213,-0.07762445086,4.43007974067732,4.94586970992091e-05,0.000382959102860392,1.56117677380537

"SMAD9",0.6840586051375,-0.07174728439,4.42976454807609,4.95109787271267e-05,0.000383219090351859,1.56016399015593

"P3H2",0.698219973825,-0.03601640634,4.42916274885164,4.96109501087997e-05,0.000383847864739082,1.55823036367713

"SCN2B",0.9259829654625,-0.07738083963,4.42852690501929,4.97167908918822e-05,0.000384521559755713,1.55618747445105

"N4BP2",0.6723958281,-0.00532703452,4.42790555436589,4.98204319079065e-05,0.000385177739218561,1.55419127433099

"P2RX1",-0.96349622935,0.09666984602,-4.42607276930899,5.0127368533193e-05,0.000387404574314621,1.54830384048237

"TMEM14A",0.871269768225,-0.07705591842,4.4239366312376,5.04874353021304e-05,0.000389893167911816,1.54144330003388

"YY2",0.621161253,0.0183281164,4.42255274763897,5.07220472331901e-05,0.00039140990723676,1.53699951894268

"CEACAM1",-0.99187859505,0.15239661196,-4.4211476088412,5.09613502342051e-05,0.000392743304996567,1.53248811092569

"TCF7",0.91359765305,0.09413102644,4.42086001666533,5.1010464338085e-05,0.000392760778537056,1.53156483103021

"MEIS2",1.175692140825,0.05999983366,4.41863017985837,5.13928384498683e-05,0.000395543378723103,1.52440710680682

"HSPD1",-0.774667310425,0.10242238966,-4.41417560641551,5.21650987210632e-05,0.000400884674642589,1.5101127946521

"STAMBPL1",0.8303755598,-0.06901811646,4.41361484163986,5.22631130496625e-05,0.000401487311646601,1.50831380325136

"PDE12",-0.5991652956,-0.01561801838,-4.41319299217705,5.23369651241336e-05,0.000401903951073182,1.50696053302729

"MGAT5B",0.7445052726375,0.15805710776,4.41159880668411,5.2616974871949e-05,0.000403902802988569,1.5018469880577

"MCFD2",-0.5875504963375,-0.04793021437,-4.41135501585536,5.26599239204724e-05,0.000404081094113348,1.50106507108588

"IL5RA",-1.30957143035,0.13518664512,-4.40994682067918,5.29086777930443e-05,0.00040568599948499,1.49654890197523

"EMC3",-0.612070189525,0.03333426638,-4.40977131992135,5.29397596966114e-05,0.000405772464146717,1.49598610483934

"FAM161B",0.635447242475,-0.03229638202,4.40789805626308,5.32726334041643e-05,0.000408018584368044,1.48997952606448

"FGGY",0.6346408297,-0.06256029994,4.40628180556524,5.35614756610247e-05,0.000409771289523179,1.48479796110377

"AP000688.1",-1.109370293225,0.20821872032,-4.40411399033118,5.39512869133557e-05,0.000412427120564025,1.47784944738479

"FAM114A1",0.586972595775,0.01871428602,4.40205266524684,5.43245136887569e-05,0.000414959433789609,1.47124367280405

"ZNF585A",0.59020749015,-0.06856477313,4.401962533866,5.43408903190903e-05,0.000414959433789609,1.47095486678607

"APOBEC3B",-1.00065791565,0.04714111048,-4.40053509334542,5.46008949608552e-05,0.000416789543948585,1.46638130000781

"ADAMTS4",-1.8085277677125,0.35502895783,-4.40015546180562,5.4670247761372e-05,0.000417163514389196,1.46516505876129

"GCNT4",0.67659961115,-0.00803218907999999,4.39960523000518,5.47709190236634e-05,0.00041777609417603,1.46340234108308

"ACSF3",1.0994325752,-0.13963601354,4.39821984050635,5.50251929292133e-05,0.000419559416722636,1.45896455313864

"SLC47A1",-0.79766678475,-0.0226648026,-4.39789373669843,5.50852129978045e-05,0.000419644887058495,1.4579200421845

"TRIM58",-2.24321807925,0.4500947386,-4.39782512211394,5.50978497748609e-05,0.000419644887058495,1.45770027395534

"PCDHGB2",0.8196787981,-0.02007501592,4.39756187360906,5.51463585848054e-05,0.000419858266326827,1.45685711956784

"BSG",-0.702809933025,0.05806303158,-4.39720821716995,5.52115923981253e-05,0.000420198776022582,1.45572443436835

"CD1C",1.117342595,-0.123106002,4.39470259299125,5.56759270767495e-05,0.000423418112081827,1.4477006211048

"TENM2",1.0996657845875,-0.06044397533,4.39355650060887,5.58895839790787e-05,0.000424885267741508,1.44403114330584

"GAS2L1P2",0.62005709105,-0.04512711426,4.39263208630483,5.60624972102806e-05,0.000426041707286435,1.44107172910265

"SULF1",1.179452178175,0.09522371454,4.39098963996329,5.63710065248449e-05,0.000427910033968515,1.43581429566145

"AURKA",-1.497629581875,0.2453702545,-4.39061560812858,5.64414938426232e-05,0.000428286416980617,1.43461715069531

"IRF1-AS1",0.8125464184125,0.01022054093,4.38988933917882,5.65786066108357e-05,0.000429038871901952,1.43229274782525

"LINC02542",1.13680762815,0.05752945252,4.38807645984076,5.69222791946283e-05,0.000431295730820837,1.42649142773422

"PDK4",-1.23806415455,0.08456936436,-4.38661120315623,5.72015378368021e-05,0.000432931328851275,1.42180330164014

"PRUNE2",1.07865210889167,-0.03434643322,4.3856364426646,5.73880533951068e-05,0.000434182584179819,1.41868491770191

"SRPK2",0.7256706114,0.05160921112,4.3836067260423,5.77783333321976e-05,0.000436812728158695,1.41219258696892

"RABL2B",0.75197482885,0.02037992368,4.38215474276791,5.80591106054668e-05,0.000438773536733605,1.40754904073474

"LINC01951",0.753430708625,-0.0261409361,4.38008093394279,5.84624401671614e-05,0.000441658729404425,1.40091804507316

"AC005323.2",0.931822282775,-0.01682655138,4.37935932689938,5.86034228379301e-05,0.000442524499134696,1.39861103876147

"CX3CL1",0.820119253125,-0.0436340425,4.37843326786115,5.87848353674303e-05,0.000443603575325198,1.39565064065563

"OSBP2",-1.7043929216375,0.11757610599,-4.37802641218573,5.88647102214695e-05,0.000444042777252381,1.39435010513241

"IGHV5-51",-1.367114498375,0.2253652021,-4.37676908511952,5.9112220522354e-05,0.000445581741744661,1.39033133537057

"EDNRA",0.7860982029375,0.06556218985,4.37453132614928,5.95552436974596e-05,0.000448591115027041,1.38318011041387

"ARSI",1.480874690275,0.18309473122,4.37433152411472,5.95949567023176e-05,0.000448725274868462,1.3825416811499

"HYAL3",-0.8771338439375,0.0262427509,-4.37393153363289,5.9674537056781e-05,0.000448860792151556,1.38126362722372

"DNAH14",0.9123415532125,0.00495968066999999,4.37391072392754,5.96786800966828e-05,0.000448860792151556,1.38119713726247

"GORAB",0.7326649395,-0.0392662804,4.37257555490675,5.99450877966277e-05,0.000450533733960861,1.3769313795669

"PDGFRL",1.498860815,0.118725576,4.3693270821935,6.05981068759698e-05,0.000454941009041726,1.36655521315222

"C5orf17",0.649651890975,-0.04750890922,4.36787778725008,6.08916798611113e-05,0.000456726565159437,1.36192704032686

"CLEC5A",-1.93454441115,0.26044239508,-4.36579075022557,6.13168709843686e-05,0.000459495264348114,1.3552635180222

"GPATCH4",-0.715372836466667,0.12208087876,-4.36397144823089,6.16898748955776e-05,0.000461952542712206,1.34945599375409

"COPZ2",1.1992569403,0.14754423124,4.36274572247062,6.19424255677221e-05,0.00046367424736262,1.3455438816002

"CLDN1",1.60653181455,0.30420489864,4.36237130846799,6.20197710798558e-05,0.000463914227778053,1.34434897441551

"CDK18",0.589825784025,-0.05412008278,4.36059945856612,6.23870732140026e-05,0.000466331691775193,1.33869490883481

"COLCA1",0.7275542464,0.01547221512,4.36059269444458,6.23884794589908e-05,0.000466331691775193,1.33867332615482

"ACLY",-0.721345408575,0.01846792114,-4.36046557995501,6.24149120054445e-05,0.000466359123693489,1.33826773729432

"SLC23A2",0.6488150631,0.000732488480000002,4.3577678762757,6.29784592395911e-05,0.00046988444024062,1.32966133379438

"IGLV3-12",-2.12162967575,0.1197076504,-4.35762273767111,6.30089186070135e-05,0.000469940562220784,1.32919837061407

"HBB",-1.4074336795875,-0.23764788067,-4.35415660570459,6.37406125682383e-05,0.000474706532278469,1.31814417202941

"DOCK2",-0.6516188902125,-0.05123111702,-4.35279860290393,6.40295349156728e-05,0.000476511845750928,1.31381431003262

"PRG3",-2.503493940925,0.54290957776,-4.35246603548858,6.41004845927362e-05,0.000476866640644873,1.3127540438203

"SNHG18",1.169657942825,0.05989142776,4.35232053953013,6.41315486584469e-05,0.000476924562219332,1.31229019617022

"CBX3",-0.8104044743,-0.000479547439999993,-4.35104564958469,6.4404371389085e-05,0.000478277090287475,1.30822609004704

"LMNB1",-0.8601313325875,-0.05198744007,-4.35100857446326,6.44123222456472e-05,0.000478277090287475,1.30810790969527

"PLPPR4",0.7226769545875,-0.07174473433,4.35092549198516,6.44301429711749e-05,0.000478277090287475,1.30784307832851

"AC012020.1",0.90618163635,-0.07591243902,4.35005282834201,6.46176147394309e-05,0.000479494998472097,1.3050615386823

"LINC01127",-1.46746056925,0.0587446216,-4.34974447998307,6.46839832364232e-05,0.000479813703312035,1.30407876548399

"PSMD14",-0.660561389225,-0.03237538838,-4.34822224865507,6.5012600284385e-05,0.000481902371427815,1.29922754631137

"HOXA13",0.79458038495,-0.07339173144,4.34705743396266,6.5265156165934e-05,0.000483599464566964,1.29551590296147

"DRAXIN",0.854481712,0.00946785269999999,4.34605576075076,6.54831034277605e-05,0.00048503898157193,1.29232446531062

"GRINA",-0.80728733325,-0.0496016509,-4.3455127583587,6.56015476844206e-05,0.000485740697129891,1.29059454130867

"LRRC55",0.831625628425,0.07066787674,4.34408777552931,6.59133698743468e-05,0.000487697046582021,1.28605522690529

"IGLV3-25",-2.000211632125,-0.0644111837,-4.34362337782106,6.60153032661488e-05,0.000488255092067793,1.2845760237917

"GVINP1",1.1647056775875,0.08198612537,4.34301970188729,6.61480371271404e-05,0.000488727365546647,1.28265329801149

"IRF8",-0.99689368285,-0.04642805328,-4.34235846252676,6.6293726017512e-05,0.000489627202107709,1.28054737036802

"AL121672.1",0.836883487375,-0.1031590361,4.34211104235288,6.63483196480509e-05,0.000489853828089826,1.27975941937962

"TRIM46",0.648814154133333,0.00544742547333334,4.33883062476886,6.70762994636901e-05,0.000494693744928755,1.26931431026859

"ASTE1",0.693756144625,-0.1033445433,4.33851442394632,6.71468792787499e-05,0.000495036078684069,1.26830769164156

"PTPN6",-0.865466960025,0.06051949658,-4.33675207123692,6.75415840661771e-05,0.000497766897247423,1.26269789104599

"AGPAT2",-1.22783233725,0.1114226152,-4.33632025249774,6.76386402301325e-05,0.000498302934568484,1.26132351352864

"SMOC2",1.48656462145,-0.08265093284,4.33607821160221,6.76931010386648e-05,0.000498524893630541,1.26055318153835

"KCNK6",-0.824688016425,0.10430351386,-4.33518912432048,6.78935182449943e-05,0.000499821200791751,1.2577236941391

"ZFHX2",0.79079464385,0.05127468093,4.33417061024484,6.81238206887196e-05,0.00050115649489066,1.25448263583342

"TNFSF15",0.96253825055,-0.09464621736,4.33198898747831,6.86196828043217e-05,0.000504623137578946,1.24754156731043

"KYNU",-0.843116244875,0.0691510941,-4.33162915296751,6.87018066192471e-05,0.000505045789025882,1.24639686815727

"SPDYE5",0.64677948025,0.0483291662,4.33074023650634,6.8905091359266e-05,0.000506358504938537,1.24356924899304

"TMEM8B",0.669540924025,-0.0137243704466667,4.33050286120481,6.89594752829535e-05,0.000506576453781696,1.24281420942608

"LINC02692",1.13223871825,-0.0195103824,4.33011553322973,6.90483038744559e-05,0.000507047186301022,1.24158224337603

"SH2D4A",0.62001315215,0.11474407162,4.32943266267688,6.92051826484194e-05,0.000507653341246265,1.23941037379218

"WDR12",-0.6467320323,0.08791030686,-4.32883986679594,6.93416494706932e-05,0.000508472338709937,1.23752511358226

"YARS1",-0.8567371842,0.00482093023999999,-4.32757820827838,6.96329669523429e-05,0.000510425841473919,1.23351307197773

"ACTA2",0.816678360675,-0.05552915596,4.32656873983575,6.98669112472469e-05,0.00051177450040529,1.23030337343681

"SFRP2",1.3515196356,0.20480001848,4.32621998154278,6.99479136551353e-05,0.000512184723004436,1.22919454370531

"TGM1",0.720318084025,0.00558204582,4.32563305761525,7.00844386911319e-05,0.000512976754970956,1.22732859093823

"TIMM10",-0.718215723325,0.03528926834,-4.32553979920662,7.01061555016925e-05,0.000512976754970956,1.22703211380152

"POU6F1",0.6575486667,0.00766444236,4.32474239425215,7.02921124790004e-05,0.000513970307091278,1.22449720889325

"ROBO4",0.8954494298,-0.01748083096,4.32230704567802,7.08630194107449e-05,0.000517590567446467,1.21675669948315

"KIT",1.14886775,0.0129724675333333,4.32171231634444,7.10031230892988e-05,0.000518244383987729,1.21486671585455

"P4HA3",1.05212934645,0.07648950716,4.31947864139055,7.15317340096871e-05,0.000521762143148655,1.20776941037416

"CCR5",0.695455039725,0.04973008478,4.31946062061382,7.15360142573493e-05,0.000521762143148655,1.20771215778602

"SEC11C",-0.623466610525,0.06170549358,-4.31831864614433,7.18077620262965e-05,0.000523371550478393,1.20408429164725

"PDZD8",-0.65295097705,0.01333991836,-4.31528566801567,7.25343855076768e-05,0.000527728867287387,1.19445115444373

"PFN1P2",-0.731610491075,0.01159355764,-4.31248753850105,7.32110878254964e-05,0.000532085408786368,1.18556668600171

"PCDHA4",0.8181239926,0.08960166208,4.31199800807216,7.3330106075753e-05,0.000532761423148946,1.18401262806177

"TOX3",1.645439124975,0.12089131498,4.31159630440207,7.3427911860508e-05,0.000533282899042215,1.18273744462699

"AC009831.1",0.660325466225,0.07938194298,4.31053321737553,7.36873624776186e-05,0.000534788056125204,1.17936300450883

"FGG",-1.791932037375,-0.0778992929,-4.3083205833299,7.4230228289026e-05,0.000538346519357722,1.17234091267615

"PHEX",0.97572016305,0.15696257644,4.30796462404431,7.43179248758714e-05,0.00053874860612605,1.1712113831066

"PRDM16",0.639361699875,0.0427651101,4.30788213477135,7.43382618859011e-05,0.00053874860612605,1.17094963438523

"SPIRE2",0.629850598175,0.07589614054,4.3059594244756,7.48138263448759e-05,0.00054200342084647,1.16484929016107

"CDC37L1",-0.9155279635125,0.10511614554,-4.30467571917616,7.51329854431292e-05,0.000544123225789618,1.16077707000218

"EMX2",1.1397128875,-0.064147663,4.30431040695859,7.52240524635413e-05,0.000544397875970694,1.1596183147094

"PCOLCE",1.325854869,-0.1139673415,4.3024822025712,7.56814132110746e-05,0.000547128014773641,1.15382000793476

"ZNRD2-AS1",0.64510069645,-0.04871550334,4.30132267071896,7.597289348548e-05,0.000549041496201239,1.15014304303301

"SPTBN5",0.5983988417,-0.000703640640000003,4.30040864694051,7.62034280625226e-05,0.000550319292966149,1.14724492597433

"AC068620.3",1.00883831725,0.01334463,4.30026760373424,7.62390624616583e-05,0.000550382632739414,1.14679774226218

"AMER3",0.787691785025,0.12871914902,4.29965252079403,7.63946520334103e-05,0.000551311599457735,1.14484767391449

"SNHG5",-0.912319585475,-0.20528987888,-4.29907170331041,7.65418574703723e-05,0.000552179428117249,1.14300635970939

"LHX6",0.964456675975,0.09583715518,4.29527862185926,7.75100038861355e-05,0.000558181005138539,1.13098432050591

"CC2D2B",0.765331338983333,-0.06353819668,4.29409986256805,7.78132892948393e-05,0.000559774814281134,1.1272492850579

"TECRL",1.012565738425,-0.18463743986,4.29283180408545,7.81408395415289e-05,0.000561517490791888,1.12323182527681

"TAF1D",-0.706705019625,0.0974423981,-4.29273784148101,7.81651641809091e-05,0.000561517490791888,1.12293415507518

"AC093107.1",-0.624794485975,-0.04458990218,-4.29207430586324,7.83371472418793e-05,0.000562555721237863,1.12083218375654

"IL4",-1.07543000835,0.03857162642,-4.29139895106837,7.85125716979972e-05,0.000563617928853738,1.1186929260037

"HOXC10",0.721547033025,-0.06145327538,4.29096521589804,7.86254365277193e-05,0.000563755782770901,1.11731910633314

"ADORA3",-1.26081431865,0.00391554007999999,-4.29092214402857,7.86366531275563e-05,0.000563755782770901,1.11718268332629

"TMEM240",0.70321914185,0.02790588998,4.29090222666928,7.86418404509584e-05,0.000563755782770901,1.11711959860713

"VSIG10",0.807027364975,-0.04060552632,4.29069561292201,7.86956710550316e-05,0.00056394435417121,1.11646519405659

"LPAR4",1.813040464725,-0.02807015122,4.29023348402203,7.88162026740099e-05,0.000564610615519271,1.11500155322146

"CLK4",0.60680236775,-0.0100044998,4.28916560805956,7.90954114258424e-05,0.000566412719081671,1.11161968835167

"IMPA2",-1.1071697469625,0.05352737893,-4.28866021481686,7.92278876195943e-05,0.000567163159171994,1.11001929076312

"CHCHD3",-0.854915630825,-0.08409758346,-4.28803573997941,7.93918761813485e-05,0.000568138581628874,1.10804192582322

"IPO9",0.77536576625,-0.1054119115,4.28783718271605,7.94440869261229e-05,0.000568313705636315,1.10741323328861

"ADGRF5",0.73349719065,0.05347455822,4.28770089062474,7.94799443363602e-05,0.000568371762500296,1.10698169900746

"C1QTNF6",1.98012549625,0.101913727,4.28747144131215,7.95403462624304e-05,0.000568604414813638,1.10625522044916

"CEBPB",-0.875691836575,0.08625768574,-4.28736650991014,7.95679840526503e-05,0.000568604414813638,1.10592299442705

"INCENP",-0.809750122825,0.15562565044,-4.28652170603816,7.979083678271e-05,0.000569998139471465,1.10324837675357

"SH3PXD2A",0.7690796005375,0.21072168663,4.2859190434409,7.99501854008236e-05,0.000570937399265275,1.10134051909743

"AC098590.1",-0.76243021055,-0.10574342704,-4.2837794166793,8.0518423074355e-05,0.000574594723771294,1.09456807966012

"DKK3",1.45139175565,-0.01898602823,4.28307186422996,8.07071961716024e-05,0.000575741307508283,1.09232885017653

"FTHL17",-0.608353451,-0.0071010213,-4.28221280953045,8.09369682536994e-05,0.000577179465917784,1.08961038477366

"SHROOM2",1.15307069195,-0.02052664944,4.28194320305932,8.10092111951241e-05,0.000577493639166911,1.08875727158361

"AC093849.1",0.791640027275,0.06817434112,4.28147811984348,8.11339809227103e-05,0.000577927035210928,1.08728567198802

"ARMH1",-1.614547896975,0.37117514442,-4.28140121303383,8.11546310182468e-05,0.000577927035210928,1.0870423333987

"KIAA1109",0.593606139375,-0.00906074549999999,4.2805415972543,8.13857928912054e-05,0.000578820118653269,1.08432258681002

"LPP-AS2",0.822526349725,-0.10443219122,4.28051465732536,8.13930477274536e-05,0.000578820118653269,1.08423735542319

"AC138028.6",-0.99048673455,0.11610812966,-4.27929016281891,8.17234649392536e-05,0.000580766683897131,1.08036361637232

"BSPRY",-1.9997440178,0.33757029076,-4.2789989732706,8.1802231209366e-05,0.000581124865817438,1.07944250222054

"RNH1",-0.6415060275,-0.061260662,-4.27797763194845,8.20790862925641e-05,0.00058228404430819,1.0762119444223

"PTMA",-0.590447670175,-0.00460538713999999,-4.2758664262509,8.26542624518508e-05,0.00058616148463604,1.06953522725368

"ANOS1",1.3260568698,-0.02910364316,4.27573688550853,8.26896818236066e-05,0.000586209758201402,1.06912560295502

"WEE1",-0.682603077325,0.00495299314,-4.27323977858054,8.33753388791849e-05,0.000590661806001639,1.06123056607218

"CDC42BPA",0.697494805275,-0.05047459878,4.27305261199478,8.34269533151652e-05,0.000590823166097859,1.06063889323909

"GPR158",1.42057327565,0.25754697952,4.27125477497519,8.39243232467559e-05,0.000593609113070843,1.05495617044302

"ACKR1",-0.86413554605,0.02390133816,-4.26929937727984,8.44685553885722e-05,0.000597167620014171,1.04877668930783

"ST3GAL1",-0.5933634502,0.05548177484,-4.26805683342122,8.48161652976263e-05,0.000599418280309682,1.04485066918936

"OTOR",-1.108582584925,0.01046778506,-4.26749836396305,8.49728535085239e-05,0.000600111624502805,1.04308626809337

"PUS1",-0.613525539275,0.04207113258,-4.26710586879467,8.50831431401118e-05,0.000600683472313788,1.0418463027602

"CARNS1",0.810496429025,0.02643335292,4.26539437477865,8.55656951421951e-05,0.000603882177772406,1.03643999899129

"GNL3",-0.68632155065,0.08454060778,-4.26516699274497,8.56300049015571e-05,0.000604127940917046,1.03572181627446

"CCDC153",0.59460833745,0.04520060596,4.26377846129187,8.60237398035406e-05,0.000606279456861005,1.03133655021233

"SDC4",-1.02652783105,0.08527768956,-4.26319212126873,8.61905316437964e-05,0.000607246084015853,1.02948496971908

"PSMA5",-0.729706811025,0.00330263018,-4.26259105847836,8.63618378809248e-05,0.000608243841355925,1.02758702078007

"OR10AA1P",-1.739770383125,0.3603191105,-4.2623154785112,8.64404903283506e-05,0.000608588579328951,1.0267168763006

"RAD51C",-0.5917055403,0.06140825376,-4.25810524237299,8.76508150018762e-05,0.000616474389893044,1.01342632986711

"EEF2",-0.75527467455,-0.07757437034,-4.25623228766557,8.81945161995141e-05,0.000620085534624449,1.00751591606105

"TNFRSF25",0.6603134055,0.009272746,4.25595581260064,8.82750514896337e-05,0.000620438852459559,1.00664355754033

"NAV3",0.6835951152625,-0.06550601599,4.25524606386627,8.84821238653752e-05,0.000621042053358618,1.00440421779774

"SLC5A4",0.74784253035,0.06832016868,4.25447282426718,8.87082573746154e-05,0.00062198999900449,1.00196475707143

"ROBO1",0.72069673455,0.08207801664,4.25383733761594,8.8894525562483e-05,0.000623082805242611,0.999960043515117

"CXCL9",1.346580545,0.149144551,4.25140927484784,8.9609725145591e-05,0.000627666341464228,0.992301747738514

"ZNF221",0.702357002975,0.12409177738,4.25093269713853,8.97507589143847e-05,0.000628439353601475,0.990798827095092

"TAS2R30",0.8804681572,0.06209781276,4.24971962607865,9.01107174920244e-05,0.000630744236411546,0.986973682383828

"UQCRH",-0.6704347341,-0.05102541028,-4.2493227563027,9.022878628883e-05,0.000631274299212486,0.985722355255869

"H2AC6",-0.73762970455,0.0735020820266667,-4.2484629959052,9.04850809859888e-05,0.000632716156737522,0.983011726914746

"IQCN",0.75669205045,-0.05618741899,4.24721599372782,9.08580702244911e-05,0.000635098861713772,0.979080673618236

"TRIM8",0.5984363598,0.06464090184,4.24711671305785,9.08878300734468e-05,0.000635098861713772,0.978767724357661

"ZNF286A",0.5850027073,-0.03339253541,4.24405658107949,9.18097792765766e-05,0.000640335033543528,0.969123371871818

"CD93",-0.843501913625,-0.0064151673,-4.24400732236203,9.18246938964704e-05,0.000640335033543528,0.968968154309677

"NTMT1",-0.644780085675,0.01539741546,-4.24133926439272,9.26360591186875e-05,0.000645334096981866,0.960562195428049

"SHTN1",0.71275006005,0.0150674579733333,4.24078425044807,9.28057138986259e-05,0.000646296215620342,0.958813887847232

"SLC9B1P3",0.786713346875,-0.0149258246,4.23998780359407,9.30496966683367e-05,0.00064733520724648,0.95630524987645

"SASH3",-0.8061788231,0.01916112052,-4.23917191709495,9.33002808037531e-05,0.000648198085149981,0.953735612498116

"PTGDR",0.695459470325,0.04135565026,4.23784878798053,9.37080495034291e-05,0.000650589806244072,0.949568910687353

"ADAMTSL2",1.250124481025,-0.04006146998,4.2364439284377,9.41429016343384e-05,0.000652945080800381,0.945145504071556

"FCRL6",1.13779933705,0.07064798764,4.23577996570365,9.43491025331799e-05,0.000654153777563381,0.943055161849122

"OGN",2.7775989037625,-0.13811733649,4.23142443661051,9.57126817334462e-05,0.000662710856152364,0.929346581459791

"ZNRD1ASP",0.968129854625,-0.0533413613,4.23106683992248,9.58254806622648e-05,0.000663031173123832,0.928221380109329

"GJC1",0.759070376275,0.12920911872,4.2309875530402,9.58505080992308e-05,0.000663031173123832,0.927971904871444

"H4C4",-1.48847849165,0.25614924318,-4.2307417074365,9.59281512955569e-05,0.000663306096437182,0.927198368707544

"VWC2",2.07861146145,-0.15188548484,4.22795395610662,9.68128675795107e-05,0.000668130541226073,0.918428397166908

"RPL18AP15",-0.63299367455,-0.00915477684,-4.22792677249534,9.68215334293348e-05,0.000668130541226073,0.918342893927594

"DOK3",-0.727430589166667,0.172085242866667,-4.22777401302085,9.68702455632035e-05,0.000668241613164617,0.917862409655359

"AVPI1",-0.6781946316,0.01113663787,-4.2274734578249,9.69661564961273e-05,0.000668462006771219,0.916917078035945

"COLGALT2",0.8965074187875,-0.08239231047,4.22736723539071,9.7000075465191e-05,0.000668462006771219,0.916582985900258

"MAGOHB",0.6196775532,-0.15256194134,4.22700137290263,9.71169909904082e-05,0.000669042673642059,0.915432301174786

"H2BC7",-1.8184084552,0.44131668384,-4.22598340124531,9.74430162399933e-05,0.000670837539222105,0.912230898396938

"HEPH",0.64580130985,0.06998882188,4.22135941746133,9.89373688521903e-05,0.000679982828931122,0.897693629522845

"GOLGA7B",0.860749174125,0.0951675333,4.22009994746236,9.93482394142802e-05,0.000682577709295698,0.893735316741175

"PCDHGB3",0.871535480975,-0.00855091822,4.21824606707264,9.99560381376705e-05,0.000686523402401808,0.887909892682151

"MTMR1",-0.742585657025,-0.04241145822,-4.21585409224851,0.000100745590465087,0.000691482632311123,0.880395427720095

"SINHCAF",-0.7353460599,0.09888374308,-4.21517671379655,0.000100970280527932,0.000692792735249924,0.878267791846246

"H2BC6",-1.1228051861,-0.00792718688,-4.21502851007877,0.000101019505346548,0.000692898435065308,0.877802307912723

"MYBL2",-0.9577879544,0.11779188418,-4.21465787564986,0.00010114271100468,0.000693511333020042,0.876638239305651

"PTCRA",-1.599548491475,0.26064860282,-4.21352834929076,0.000101519086952174,0.000695859168108443,0.873090985807539

"H3C7",-1.589813158875,0.3039754479,-4.21326612399321,0.000101606658896906,0.000696226497484883,0.872267537885929

"COL1A2",1.8304809393,-0.32462044256,4.21271604368917,0.000101790600434889,0.000697253701675025,0.870540238510734

"HBD",-2.63391296875,-0.107548665,-4.2124144184472,0.00010189159823766,0.000697712254242375,0.869593155169332

"MILR1",0.664255337175,0.00720964274,4.21025817542537,0.000102616446080654,0.000702440944637632,0.862823640032009

"MCOLN1",-0.64786393755,-0.00945374564,-4.20972821307763,0.000102795364687425,0.000703246217374554,0.86116007977656

"CEACAM4",-1.08361476015,0.15410625788,-4.20970640317327,0.000102802734334088,0.000703246217374554,0.861091620288339

"FAM66D",0.799624216825,-0.02438879254,4.20820002471303,0.000103312988558661,0.000705769080216407,0.8563636339936

"SLC39A5",0.8411790592,0.04702303736,4.20810937097394,0.000103343773948127,0.000705769080216407,0.856079130016182

"HES4",0.70184729325,-0.0637927254,4.20781834039378,0.000103442666157509,0.000706084998867751,0.855165791434021

"SCIN",0.982224090625,-0.2108658995,4.20711362327346,0.000103682509895525,0.000707139568155632,0.852954309664126

"SYK",-0.99118101435,0.15381670052,-4.20629444409678,0.000103961988425685,0.000708573925104936,0.850383856318457

"ARHGEF28",0.663375311525,0.04171509512,4.20532123697167,0.000104294967356337,0.000710370774998877,0.847330399989574

"SOX5",0.840361657225,0.03424816378,4.20319448048326,0.000105026236791841,0.000714164467106285,0.840658840435084

"OSBPL3",0.6528146134375,0.03893584395,4.20243033777789,0.000105290195377999,0.000715484418873777,0.838262147684319

"EFHB",1.10755420691667,0.171078198866667,4.20132824174777,0.000105672028266564,0.000717841019603901,0.834805851132293

"KCNS1",1.37976855145,0.18095616666,4.19952224828391,0.000106300642406558,0.000721632724196673,0.829142995574089

"AC002558.1",-0.74080273035,-0.06242758758,-4.19658727181712,0.000107329974960228,0.000728137922842765,0.819942610519185

"VWA5A",0.6959256526625,-0.02110840722,4.19594027081608,0.000107558184492268,0.000729444582548025,0.817914847818067

"ZBED2",0.716376949375,-0.1391823883,4.19532900276492,0.000107774223055458,0.000730667862991473,0.815999213919943

"RGCC",-0.766478062925,-0.07852954864,-4.19495586487448,0.000107906307018065,0.000731115800966936,0.814829914796815

"MYO10",0.68534669945,0.08996198756,4.19494073933971,0.000107911664492582,0.000731115800966936,0.814782517090256

"TUSC3",0.9012238656,0.18464086448,4.19456631306936,0.000108044368873485,0.000731772902307425,0.81360923298999

"KPNA5",0.601318732425,-0.03114410896,4.19202930526995,0.000108947719226768,0.000737647346833451,0.805660717655181

"AC092474.2",-0.7368488731,0.04954009912,-4.19174711995965,0.000109048648675564,0.000738086790242802,0.804776766619295

"TRIM25",0.612927355675,-0.00924107406,4.19109408768384,0.00010928256745188,0.00073942577343267,0.802731240255752

"GADD45G",-0.870698057175,0.11679337576,-4.18942253821238,0.000109883544401049,0.000742756205110094,0.797496061162414

"ASPN",2.6018880221,0.09831542068,4.18882090298007,0.000110100636179763,0.000743978177457446,0.795612027347121

"NT5M",-0.970135411325,0.18810780484,-4.18802668940694,0.000110387854825399,0.000745427280706256,0.793125130392179

"GP6",-1.695137315425,0.17716937666,-4.1878425215512,0.000110454560918824,0.000745631974993369,0.792548483687959

"HOXD8",0.680858868875,0.0133227436,4.18745837716904,0.000110593824993283,0.000746326181311719,0.791345731289005

"DZIP1",0.816749517025,-0.01658228838,4.18713743083907,0.000110710308511276,0.00074686624984492,0.790340892266131

"ZSCAN31",0.79066663055,0.02275623094,4.18698337820704,0.000110766262306703,0.000746997755806362,0.789858587784344

"DUSP18",0.695997130825,-0.01400784494,4.18649194056313,0.000110944941965146,0.000747956555110862,0.78832006328783

"ZMIZ1-AS1",0.61874640635,-0.04463706092,4.18617996595972,0.000111058516582944,0.000748475949918212,0.787343421967816

"CPNE4",0.715401707975,0.00806759698,4.18468253104084,0.00011160523168679,0.000751913182110801,0.782656168646285

"SPATS2L",0.601491095225,0.05521297398,4.17981693479952,0.000113399759284516,0.000762498939357123,0.767431539594764

"ZNF83",0.635724709475,0.00230344708,4.17849969238618,0.000113890381249355,0.000765303672154033,0.763311315761572

"SPINK8",-1.05060517805,-0.15815433444,-4.17844009189618,0.00011391262877477,0.000765303672154033,0.76312490531302

"TNFRSF1B",-0.6395133672625,-0.05324368531,-4.17839639165111,0.000113928943775371,0.000765303672154033,0.762988226345083

"NANS",-0.623385449875,-0.0612384029,-4.17774537012349,0.000114172264116892,0.000766686773919002,0.760952142488881

"FRY",0.7125113822,-0.02544411032,4.17716000402939,0.000114391476160751,0.000767907130924466,0.759121528792406

"ABCC5",0.680706806608333,-0.117167006046667,4.17600585909945,0.000114824885728358,0.000770059659182522,0.755512539268604

"C12orf60",0.752271624125,-0.0811965681,4.17555205386024,0.000114995736767782,0.000770700901177077,0.754093631367077

"TMEM185A",0.62109558415,-0.03886591068,4.17192422490189,0.000116370458502972,0.000778895117219497,0.742753230419701

"IGLL1",-1.562829220625,0.3809635525,-4.17043682997906,0.000116938686327537,0.000781730602029081,0.738105097448438

"DAAM2",-0.8604079426,0.14281540892,-4.17041586460149,0.000116946714916982,0.000781730602029081,0.738039586067777

"TAS2R20",1.6287704414,0.46273014712,4.1694809649185,0.000117305274401436,0.000783871644467263,0.735118429285133

"KRTAP10-6",-0.99064183945,0.12593716644,-4.1686221409503,0.000117635596622359,0.000785664347249229,0.732435256982154

"TWSG1",0.726941492025,0.00247003661999999,4.1681476778618,0.000117818472194842,0.000786531397304637,0.730953035717548

"RHBDF1",0.669825194375,-0.027494812,4.16795033439104,0.000117894616844396,0.000786783358276216,0.730336559488944

"AL135791.1",0.807086493825,-0.18208698394,4.16661470668114,0.000118411223140004,0.000789716516826956,0.726164599778136

"TBC1D2",-0.726666927725,-0.05325956278,-4.16546251443789,0.000118858642591235,0.000792442521773258,0.722566141377087

"DGKI",1.185951067875,-0.0473083267,4.16276228503203,0.000119913631605414,0.000797918312546842,0.714134845762175

"TF",-0.791424228575,-0.19715534386,-4.16122721313441,0.000120517433877451,0.000801415509666085,0.70934286971063

"LINC00239",-0.70472757755,-0.13511985624,-4.15936920772427,0.000121252204325442,0.00080577851515396,0.703543958322711

"KLHDC10",0.6081429333,0.03573738964,4.15892453491284,0.000121428698931616,0.000806689748285004,0.702156303724354

"GATA3",0.905626490375,-0.0056252849,4.15539375320152,0.000122838970547811,0.000815001563660475,0.691140651425591

"AL158835.1",0.698495382475,-0.00500327302,4.15493781609765,0.000123022236031373,0.000815953244354406,0.689718511109255

"FNDC5",1.2329010268,-0.15260257656,4.15398996954013,0.000123404075351566,0.000818089544476679,0.686762271340219

"BCAS1",0.8618428385625,-0.00500750985,4.15394012024901,0.000123424188889956,0.000818089544476679,0.686606805461428

"OBP2A",0.770970555975,0.00820611078,4.15367377691081,0.000123531708844462,0.000818537403235877,0.685776171185069

"CDH2",1.469392441325,0.04294824306,4.15245937398325,0.000124023102865097,0.000821527750242519,0.681989191981885

"RGL3",0.7186946739125,0.07233117793,4.14823602367484,0.000125746837762549,0.000832138634392476,0.668823372481408

"SGSM2",0.622139950575,-0.00838016258999999,4.14735195003718,0.000126110596766874,0.000834276366341529,0.666068203803667

"ITGAX",-0.9228255604,0.03417791968,-4.14623844815449,0.000126570208159089,0.000837046618709945,0.662598444149384

"COL7A1",1.18268745615,0.11704811092,4.14567053733865,0.00012680524534634,0.000838059956985744,0.660828965361606

"MRVI1",0.630525031425,-0.01643672866,4.14549913697642,0.000126876264700264,0.000838258920083522,0.66029494483957

"C1orf115",0.7268013902,0.11228480336,4.14513528196995,0.000127027155081867,0.000838985284757348,0.659161342324036

"C17orf99",-2.847296611775,0.50562362758,-4.14369770664449,0.000127625020269768,0.000842397123720921,0.654683005974207

"FBXO16",0.7219807442,-0.05164915414,4.14188756334031,0.000128381712778083,0.000846839836251569,0.649045131054977

"CD276",0.7721087061,0.07500055283,4.14163698074946,0.000128486805790602,0.000847260275840954,0.648264761574982

"HBM",-3.395839349925,0.71736905916,-4.14149795712502,0.000128545147538066,0.000847372259575255,0.647831821344289

"BCLAF3",0.5874731921625,0.02648175718,4.13860508437993,0.000129764996573244,0.000854039591966795,0.638824607757758

"ADAM8",-0.9312987584,0.09325903128,-4.13767662648647,0.00013015887694477,0.000855807147254318,0.635934429098183

"ADAM20",1.144589906975,-0.00230169442,4.13532197452977,0.000131162997598507,0.000861856156125147,0.628606110604382

"TMEM273",0.657741327066667,-0.0794107961133333,4.13452698475548,0.000131503706872521,0.000863541008462889,0.626132349990052

"NUCB2",-0.883258664375,0.1223316595,-4.13418356638221,0.000131651151155708,0.0008637778457827,0.625063811471757

"PIGZ",0.691554502525,0.10403200502,4.13414841075228,0.00013166625402086,0.0008637778457827,0.624954427991776

"REEP1",1.0044673931,0.09564195548,4.13201550791155,0.000132585698696436,0.000869253054365624,0.61831895702089

"CAMP",-4.22563345505,0.63387051596,-4.13105370022875,0.000133002345075075,0.000871080031310994,0.615327321443765

"ARHGAP9",-0.900621740425,0.12077094966,-4.1310297716735,0.000133012726874016,0.000871080031310994,0.615252897727424

"TAS2R50",0.945657631675,0.17279760534,4.13097975311961,0.000133034430787393,0.000871080031310994,0.615097328359346

"MSC-AS1",0.7505143299375,0.02344503095,4.13051209954971,0.000133237519921011,0.000872131088863156,0.613642861680582

"HMSD",0.768799347525,0.06279080202,4.13004794469125,0.000133439386845664,0.00087317347738549,0.612199356865284

"TMEM119",1.9616739757,-0.10231225144,4.12861720764156,0.000134063500147941,0.000876138115161411,0.607750320530208

"GHR",-1.22750550805,-0.00522613044,-4.12806102368848,0.000134306880673672,0.000877199735679704,0.60602101010535

"CYB5A",-1.1296856330125,0.29264960259,-4.12805030313626,0.00013431157608489,0.000877199735679704,0.605987678446627

"MYH7B",1.0005931985,0.1185072078,4.12770946816968,0.000134460938718787,0.000877639783761629,0.604927998359529

"CIRBP",0.8077669712375,-0.06602940526,4.12770105605508,0.000134464627158715,0.000877639783761629,0.60490184504537

"MT1X",-1.6712452344,0.10685501648,-4.12742693715689,0.00013458487310905,0.000878144866324272,0.604049622182021

"ZNF490",0.634806704875,-0.0502489001,4.12650532054593,0.000134989917522808,0.000880507300288853,0.601184564316047

"NEK7",0.6174983807625,0.09110718611,4.12628687547582,0.000135086095785811,0.000880854210840136,0.600505523794388

"ENKUR",0.833934846008333,-0.109616264526667,4.12499142259236,0.000135657831798309,0.000884096361781411,0.596478950025307

"NMUR1",1.1476190452,0.21235111866,4.12435931970998,0.000135937655298041,0.000885561425038556,0.594514451925999

"EEF1A1P11",-0.6904484815,-0.0346393202,-4.12341137461568,0.000136358346871368,0.000888007301596248,0.591568634306209

"ATAD5",-0.657600111025,0.02569947518,-4.12222806062609,0.000136885264691013,0.000890601874560008,0.587891858860019

"GEMIN2",-0.746685474975,0.05732662242,-4.12144945111327,0.000137233047637697,0.000892297899080018,0.585472860171467

"TRBV12-5",1.046703902025,0.16312915762,4.1209421403372,0.000137460109724239,0.000893207335245863,0.58389685930354

"PPP1R14C",1.4521225175,-0.120337654,4.12047656686898,0.000137668811437268,0.000894171317783477,0.582450603260925

"TRNP1",0.946928307825,-0.18076368524,4.12041652396182,0.000137695749102249,0.000894171317783477,0.582264092047026

"AL121827.2",0.958668211475,0.05284379258,4.11985801270598,0.000137946564633291,0.000895516228202429,0.580529253713307

"LINC01483",1.00552351825,0.0607722406,4.11806996003871,0.000138752522683345,0.000900462998015956,0.57497601857162

"RNF151",0.641105510075,0.01621215706,4.11750163598618,0.000139009647088526,0.000901275205553709,0.573211199555472

"GALNT15",-1.52763434655,-0.05037885644,-4.11697516669022,0.000139248247319995,0.000902536567887392,0.571576460113974

"UTRN",0.62571500497,0.054730374636,4.11668933517071,0.000139377954719802,0.000903091567457084,0.570688968309145

"TRAV39",0.8480414466,0.03374880128,4.1147881006731,0.000140243699923905,0.000907859989602572,0.564786513808074

"CLEC11A",1.26859594635,-0.07094161662,4.11478126734529,0.00014024682092406,0.000907859989602572,0.564765301924836

"ANKRD30BL",0.605812536275,-0.09115749398,4.11456289104732,0.000140346595843746,0.000908218905763316,0.564087431682787

"MLPH",0.8829551619,-0.12679056948,4.11445371232319,0.000140396504787356,0.0009082550016051,0.563748532517782

"STMN2",4.30514482125,1.384124221,4.1132952211616,0.000140927147299553,0.00091140006119736,0.560152761967992

"CXCR3",0.6606634147125,0.05677033697,4.11301899463172,0.000141053958496306,0.000911895520031269,0.559295473456725

"MCC",0.678585983975,0.01285746818,4.11293448809382,0.000141092776186017,0.000911895520031269,0.559033207173552

"CYP27C1",1.3750945653625,-0.11052266801,4.10669881087369,0.000143985849263178,0.000928543304911549,0.539688162097071

"MYO1F",-0.93546011855,0.11378152976,-4.10375759486254,0.00014537031161665,0.00093711304391631,0.530568652575675

"ITGA2",1.4279679675,-0.1552722087,4.10367291770523,0.000145410360475257,0.00093711304391631,0.530306151535193

"FURIN",-0.6839151088,-0.00601469654,-4.10314831865862,0.000145658712898483,0.000938150176002555,0.528679943221818

"NPRL3",-0.608000989975,0.02957439522,-4.10258810998368,0.000145924377162485,0.000939565882873979,0.526943462870052

"CCDC69",-0.6608260954625,-0.000756319370000012,-4.101482614093,0.000146450007521991,0.000942596018864325,0.523517101326743

"ATP8B1",0.675063976225,-0.12041735802,4.10138933697298,0.000146494441835661,0.000942596018864325,0.523228020350474

"ZNF663P",0.998798074775,-0.24510249668,4.10110486105616,0.000146630037975848,0.000942924111126546,0.522346403449532

"P2RY6",1.01347235945,-0.08308083444,4.09975156513553,0.000147276756649267,0.000946785751562655,0.518152833626885

"WEE2-AS1",0.61364830065,-0.17693752148,4.0994695306377,0.000147411884005728,0.000947331283031565,0.517278957067544

"NKG7",-1.719636349975,-0.00458728358,-4.09938148396963,0.000147454093205177,0.000947331283031565,0.51700615290576

"ZNF785",0.618926501125,0.0510167414,4.09800673409737,0.000148114663096824,0.000950383093494437,0.512747001870123

"LAMA4",0.64533451224375,0.084701823045,4.09751603187261,0.000148351139979941,0.000951602428274588,0.511226916461364

"AC040168.1",0.585530104425,-0.01890253896,4.09682003868589,0.000148687177097735,0.00095345943173033,0.509071041759488

"PODXL",0.704595329625,0.0120935432,4.09600914142263,0.000149079621890751,0.000955484223458394,0.506559476718095

"JAK2",-0.625572389025,0.04578900378,-4.09597492033396,0.000149096205700727,0.000955484223458394,0.506453490349299

"AC092070.2",0.943072147325,0.07439570286,4.0954559694182,0.000149347912557773,0.00095649891606241,0.504846298186099

"SOX11",2.194028131425,-0.18795449586,4.09495216970696,0.000149592664382818,0.000957767033710993,0.503286126786844

"NDUFA6",-0.66925518645,-0.02175735316,-4.09396902270195,0.000150071408187275,0.00096053202466132,0.500241785022332

"PENK",1.69271536775,0.5466217642,4.09318208558923,0.00015045567659297,0.000962089857065162,0.497805276217033

"SOX18",1.02030896265,-0.00120826488,4.09121276877151,0.000151421493210309,0.000967661744508051,0.491708920121111

"FAT3",0.8186538256375,0.14482942131,4.08969448657154,0.000152170204857115,0.000971840136257036,0.487009822083199

"UBE2M",-0.668075183875,-0.1049091511,-4.08900602813159,0.00015251088531845,0.000973409040001375,0.484879324683483

"BAG4",-0.5856408139,0.07012913628,-4.08822019895152,0.000152900652120138,0.00097528909110753,0.482447724461519

"XAGE3",0.877999751675,0.00591867664000001,4.08777326455958,0.000153122759249181,0.000976401833643705,0.481064874826624

"NBEAP1",0.766187401825,0.05164592896,4.08473228222161,0.000154642314242964,0.000984559271040972,0.471657854316894

"CLEC3A",2.83055055835,-0.21549040132,4.08449171737956,0.000154763144006002,0.000985022458650189,0.470913837436242

"SPDYE21P",0.773171523825,0.00501950226,4.08430837605636,0.000154855293203526,0.00098508809270943,0.470346815742017

"ANKRD2",0.7530097549,-0.02654055958,4.08427988346674,0.000154869618636132,0.00098508809270943,0.470258697549518

"PLEKHJ1",-0.603833517225,-0.08136555478,-4.08330764938965,0.000155359205838966,0.000987895533590542,0.467252082911918

"RPL5",-0.7495382691,-0.10486228628,-4.08143984303735,0.000156303984924993,0.000992589848017523,0.461476938138129

"PCDHAC2",1.208830949625,-0.1582491973,4.07991604090479,0.00015707887776328,0.000996665236176553,0.456766417820706

"ALB",-1.8327547113,0.24395765896,-4.07928207022926,0.000157402362938319,0.000998408548569747,0.454806888765739

"B3GNTL1",-0.673063097325,0.05587955384,-4.07875546938565,0.00015767155276149,0.000999806491172211,0.453179343782051

"HESX1",0.647831860125,-0.1341912759,4.07624660354718,0.000158960177551674,0.00100642030830615,0.445426741205605

"SGMS2",1.653906128225,0.09281978258,4.07160618286832,0.000161370554680879,0.00102041972972279,0.431093794169812

"ETV7",0.694255549175,0.11940772064,4.0709003353129,0.000161740280528093,0.00102208499597648,0.428914352328905

"LMOD1",0.871855470041667,0.0357812523333333,4.06928205783207,0.000162591039147385,0.00102686967017621,0.423918325369239

"CYP7B1",0.585489501325,0.05095638306,4.06849876759429,0.000163004383858316,0.00102916296347894,0.421500472988118

"AL353763.1",0.868138462125,-0.0386204053,4.06792658483664,0.000163306969295077,0.00103043830825918,0.419734413667499

"ADAMTS13",0.6080191850375,-0.02249251747,4.06572436235044,0.00016447664503824,0.00103622309457056,0.412938359143953

"PRELID2",0.67936223435,-0.16230692972,4.06481919739838,0.000164959757309394,0.00103887025561348,0.410145561415613

"ISG15",1.351457242675,0.23362893914,4.06457329453946,0.000165091239758101,0.00103913650389062,0.409386906585123

"FDXR",0.651505140925,-0.04056413926,4.06408292516584,0.000165353739585617,0.00104046910830163,0.407874097632089

"KCNAB2",-0.7955192044875,0.11152089666,-4.06328315568207,0.000165782731583136,0.00104252811684324,0.405406975249111

"TENT5B",0.835892975225,-0.03672962182,4.06318274251378,0.000165836668724322,0.00104254730556119,0.405097238890746

"CFAP45",-0.78293968535,-0.00884697128000001,-4.06247508812584,0.000166217268723521,0.00104461944834586,0.402914504660424

"ADAP2",0.76151567045,0.00643609836,4.0605888279487,0.000167235897157234,0.0010500548761745,0.397097344317505

"DPP4",1.41462747875,-0.25363809,4.06004828846194,0.000167528914360829,0.00105125035143175,0.395430591771304

"FNDC4",-0.886232925525,0.07504295248,-4.05972443490557,0.000167704707819378,0.00105193264137018,0.394432043772611

"SGCG",1.075492674825,-0.04825778844,4.05965884513641,0.000167740332846367,0.00105193264137018,0.394229813661489

"AC114296.1",0.814080658225,0.10244479358,4.05617743431217,0.000169641806996978,0.00106223146141629,0.383498107574042

"NANOS3",0.6282855283,0.06314906964,4.05363436820322,0.000171043944719255,0.00107035685382043,0.375661871662784

"CSF1R",0.823335591925,-0.18572639346,4.04978600277101,0.000173187119210755,0.00108270147813389,0.363808218896331

"ST6GALNAC3",-0.913020771725,0.15616504667,-4.04971302870177,0.0001732280088502,0.00108270147813389,0.363583501037986

"EGFL6",2.21774254675,0.1560427434,4.04889948721073,0.000173684494532476,0.00108522352057986,0.361078404040788

"ABCA13",-2.486003013,0.4044331696,-4.0477568324476,0.000174327614859113,0.00108890980891265,0.357560317748474

"NXPE2",1.085153491075,0.00965490031000001,4.04745968706483,0.000174495234349743,0.00108937974234366,0.356645528339055

"COL11A1",2.1546860835,0.3253841848,4.04743495725062,0.000174509191447281,0.00108937974234366,0.356569396871001

"PGR",0.6343067207125,0.01107241822,4.04644765373718,0.000175067292512057,0.00109253082210996,0.353530147144688

"TIMM17A",-0.60744741195,0.03912799854,-4.04394239894444,0.000176491220583183,0.00109974213117647,0.345819832050878

"SLC41A2",0.756769452925,0.08901894194,4.04326946537938,0.000176875605526505,0.00110180219094771,0.343749187974104

"CRTAC1",1.895597622625,-0.1157485519,4.04272424395511,0.00017718763417313,0.00110341041001188,0.342071648220441

"SQOR",-0.6277706168,0.02415279416,-4.0416129755088,0.000177825259745365,0.00110704464347099,0.338652849668319

"AUP1",-0.615963554825,-0.04528520586,-4.04103752808448,0.000178156312858122,0.00110876869314617,0.336882683851913

"WNT1",1.1647849418875,0.22721176351,4.03982358769899,0.000178856645876811,0.00111245141491321,0.333148836252384

"FCER1A",1.589700153,-0.3385590311,4.03679181176143,0.00018061735658752,0.00112238046762666,0.32382617756349

"TRIM50",0.9808947679,-0.04184468323,4.03573825859214,0.000181233126860113,0.00112552419009094,0.320587357112728

"NRBP2",1.060376380025,0.13370612302,4.03422145466396,0.000182123221210001,0.00113070925943955,0.315925175944841

"GABRD",0.921830114725,-0.01519641972,4.03367690957271,0.000182443802848681,0.00113201351688788,0.314251633230932

"HILPDA",-1.360525781175,0.28937003306,-4.03254000804345,0.00018311487384399,0.00113514600160837,0.310757982491024

"KIRREL1",0.676486199475,0.00530739408,4.03051449070577,0.000184316382850562,0.00114224865451976,0.304534901953824

"PHBP10",-0.661944881625,-0.0727382745,-4.02847908839387,0.000185531435488114,0.00114873618926578,0.298283064866848

"MSS51",0.872422465825,-0.10403700094,4.02831497693159,0.000185629740183707,0.00114899761839389,0.297779059046523

"LINC01999",0.891965622375,-0.1637854561,4.02768192933206,0.000186009415338257,0.00115065244609004,0.29583499319119

"H4C9",-1.19885312455,0.20308877796,-4.02593322010021,0.000187062124281846,0.00115576863760147,0.290465585838368

"AC133552.2",0.62671309465,-0.00428915128,4.02498471829671,0.000187635522688589,0.00115799945329836,0.287553713832385

"FLOT2",-0.677679609875,-0.0385740895,-4.02496201699409,0.000187649267129567,0.00115799945329836,0.287484025830763

"CRELD1",0.781836752075,0.10969805546,4.02367520232712,0.000188429959453843,0.00116211710093026,0.283534117962793

"GAPDHP33",-1.02165891925,-0.098621292,-4.0223491403105,0.000189237748601245,0.00116579114135121,0.279464418010196

"IL13RA2",0.967166959175,0.27619653584,4.02232368689756,0.000189253286613341,0.00116579114135121,0.279386307926094

"ME2",-0.6847813087,-0.01598337966,-4.0212396686089,0.000189916170958752,0.00116917142746482,0.276059965810942

"MOSPD1",-0.7585060819125,0.08503259267,-4.02111908318416,0.000189990048371404,0.00116927489066787,0.275689974352892

"PARPBP",-0.66863928035,0.0984026507866667,-4.01975739032742,0.000190826229223093,0.00117367018153371,0.271512297063293

"SAMD13",0.728173107325,-0.08556432714,4.01967639886114,0.000190876075989375,0.00117367018153371,0.271263837952367

"B4GALT1",-0.70288444805,0.12074593616,-4.01806537833145,0.000191870210035157,0.00117872169811103,0.26632221365699

"B4GALNT4",1.159530428,-0.1865136426,4.01791241539071,0.000191964860765824,0.00117894966048267,0.265853070013025

"VANGL1",0.605420030325,-0.15188594524,4.01732553225974,0.00019232843265793,0.00118082856706493,0.264053160539155

"ELOVL4",0.9088902216,-0.08473689472,4.01640310704639,0.000192901217671049,0.00118363586332568,0.261224451934126

"GPT",-0.96009904755,-0.06392898604,-4.01562269701105,0.000193387105358643,0.00118626198041553,0.258831508154702

"CABYR",0.7067742960875,-0.05294685313,4.01455750241849,0.000194052211858564,0.00118962947832383,0.255565726055189

"STK39",-0.8507625032,0.01308202044,-4.01389601206383,0.00019446635847625,0.00119145536855903,0.253537886195143

"MKRN7P",-0.673365645775,0.12045103238,-4.01379041372379,0.000194532550656166,0.00119150460324171,0.253214183847651

"RNASEL",0.6365474243375,-0.12669481753,4.0135482159617,0.00019468444970673,0.0011920786029861,0.252471764660735

"CU633906.4",1.081751364625,0.078014775,4.01311758725218,0.000194954810169514,0.00119337739789453,0.251151797032082

"MAMDC2-AS1",0.832210140475,-0.06630577362,4.01194859147526,0.00019569056919015,0.00119716583504563,0.247568947065396

"GASAL1",0.766733826325,0.06701598106,4.01162720038986,0.000195893320893863,0.00119769094553072,0.246584011280924

"MERTK",-0.652901966775,0.04774320268,-4.01129894362575,0.000196100613506944,0.00119824895610212,0.245578076929902

"AC084357.1",0.71235430835,0.01507893758,4.01129744646888,0.000196101559440181,0.00119824895610212,0.245573489028732

"HOMER2",1.5080875671,0.19230394068,4.00986692447154,0.000197007410234792,0.00120326979961377,0.241190188031095

"OR1J1",0.88953816745,0.01927385196,4.00876339696491,0.000197708960737301,0.00120655065002343,0.237809390937106

"EGFLAM",-1.1143230506,0.26443975652,-4.00821758322985,0.000198056845483128,0.00120767519352926,0.236137398585875

"TNFSF12",0.58640075585,-0.02091664232,4.00712535229306,0.000198754777457715,0.00121157033042358,0.232791918313642

"AC023908.3",0.818353351025,0.00748767922,4.00652974310215,0.000199136370951828,0.00121353538609787,0.230967778387352

"EFR3B",0.621246247291667,-0.0456516988666667,4.00595658362552,0.000199504250067613,0.00121505442193378,0.229212526260004

"ELL2",-0.801452251625,0.1736149987,-4.00446323643422,0.000200465834846485,0.00122018539047379,0.224639887183888

"TMOD4",0.8398848787,-0.00757594374,4.00172379860337,0.000202241453107685,0.00123026214111349,0.216254002634225

"DIRC3",1.488147865,0.398367747,4.00124214806695,0.000202555211414319,0.00123180503753534,0.214779895469756

"MEOX2",0.678805262725,0.08648331558,4.00111404224892,0.000202638741690515,0.00123194734117367,0.214387838901139

"GPAT2P1",-0.722504051675,-0.02801583144,-4.00093909135189,0.000202752870676975,0.00123227553083058,0.213852427592468

"C5orf30",-0.716306381675,0.16831251306,-4.00035835238742,0.000203132160570365,0.00123421462211318,0.212075248899748

"TROAP",-1.034714457325,0.10190534414,-3.9993699702246,0.000203779266183914,0.00123741244978542,0.209050906737126

"MAGED2",0.677961612325,-0.05399339944,3.99919658303531,0.000203892990087266,0.00123773617212086,0.208520400660479

"MUC20-OT1",0.7303252948,-0.01040965531,3.99901500887495,0.000204012149545455,0.00123809268954007,0.207964857996616

"AL731563.4",0.6080726262,0.01244153196,3.996913657356,0.000205396084126841,0.0012457533959712,0.201536532808145

"ESYT3",0.779323665741667,-0.11143554674,3.99627553265443,0.000205818141392625,0.00124794379427408,0.199584767708376

"LINC01833",1.02013990275,0.0823867512,3.995813475105,0.000206124270476271,0.00124943019334847,0.198171621611072

"BLVRB",-0.85101506845,0.11760112524,-3.99471550524956,0.000206853479744791,0.00125347947146148,0.194813956055732

"MROCKI",0.856018021375,-0.2429401329,3.9941561756934,0.000207225912613723,0.00125536501999703,0.193103672772589

"OVCH1",0.919015570225,-0.03208165982,3.99391146850022,0.000207389056040307,0.00125552825282461,0.192355461349322

"PRSS35",2.29355776825,-0.2273553174,3.99384003806135,0.000207436701279349,0.00125552825282461,0.192137061669654

"COL28A1",0.69999515035,-0.17871564522,3.99135564297871,0.0002091004303737,0.00126335877838288,0.184542246927521

"FAM111A",0.72341509865,-0.07312043558,3.98620205874518,0.000212592856884392,0.00128105954466101,0.168795533091343

"CYP8B1",-1.31217961865,-0.18424387302,-3.9859904363186,0.00021273746509132,0.0012815540090535,0.168149149550519

"C1QTNF2",0.64135073355,0.01543964184,3.98454137279554,0.00021373021205298,0.00128708486880548,0.163723582481416

"AC018445.4",0.8653075467,-0.03023831734,3.98446708248753,0.000213781228452999,0.00128708486880548,0.163496715943664

"CBX3P3",-0.5850062222,0.02786856624,-3.98339364705474,0.000214519689098094,0.0012907722144616,0.160218922115051

"DLGAP4",0.66647644355,0.02368665684,3.98247530260061,0.000215153411907437,0.00129420525635924,0.157415072258668

"SHC2",0.9646356757,0.17067494956,3.98161434973095,0.000215749172940791,0.00129740799976839,0.154786753899807

"CASC4P1",0.72098399385,-0.12128752832,3.97977788991089,0.000217025294369221,0.00130431628953846,0.14918139629668

"IFI27",0.95409571425,0.1294834134,3.97960374726625,0.000217146680650177,0.00130466310649878,0.148649937223681

"SPARC",1.274464355125,-0.0144375279,3.97931710974362,0.000217346624432163,0.00130509895702408,0.147775185884152

"MMP11",1.44774331145,0.14163547616,3.97906842488003,0.000217520238451764,0.00130575875927329,0.147016283901239

"LINC02265",0.740267038375,0.0452525607,3.97844524480422,0.00021795588702578,0.00130722488682207,0.145114658065018

"DACH1",-0.8546466568375,0.04430482003,-3.97703180404386,0.000218947112022464,0.00131278561051105,0.140802138254605

"AC073544.1",0.77251330615,0.05173138692,3.97544721173979,0.000220063540863865,0.00131909357086567,0.135968372888629

"PDGFC",0.733004637525,-0.07639611998,3.97328183561318,0.000221598058061432,0.00132790319203353,0.1293645661999

"LINC01152",0.663004948275,-0.14419478538,3.97181260887182,0.000222645121748985,0.00133261853765504,0.124884896456275

"ULBP2",0.79772287765,-0.02218437388,3.9704176455122,0.000223643682224472,0.00133781365296788,0.12063245771271

"ANK1",-1.8654291717,0.39307387849,-3.97001657184766,0.000223931583638699,0.00133798963674434,0.119409960109819

"LINC02232",-0.792052668175,0.06887476086,-3.97001275433112,0.000223934325673387,0.00133798963674434,0.119398324391291

"ACPP",-1.339658659225,0.16428967402,-3.96984935622552,0.000224051721195345,0.00133830077663272,0.118900295532193

"CLIC5",0.627518243608333,0.0495802937533333,3.96664729311006,0.000226364317952273,0.00135093275450223,0.109142741463299

"WDR78",0.663154271925,0.03586632404,3.96616187761461,0.000226716901327253,0.00135224916285088,0.107663908962497

"WDR76",-0.696060187775,0.05280012178,-3.96429289353295,0.000228079403973048,0.00135997986862625,0.101970880235728

"PLEKHB1",0.608463691875,0.0497968125,3.96352757510434,0.000228639605589496,0.00136292354940867,0.099640084138624

"H4C8",-1.31110128495,0.16722911704,-3.9628708617597,0.000229121369027001,0.00136539808278801,0.0976402357030288

"AVPR1A",1.097608631525,0.24318588802,3.95989046745068,0.000231320127011626,0.00137729926248596,0.0885664118410343

"PSME3",-0.6448171298,0.07565811316,-3.95804995011983,0.000232688113043266,0.00138504185358234,0.0829647395560906

"BX322639.1",0.8412989931,0.01860966168,3.95736850330761,0.00023319658523922,0.00138726238048233,0.0808910819808224

"NECTIN1",-0.657659995425,0.01890560166,-3.95708137791645,0.000233411149451338,0.00138813574164267,0.0800174093344079

"LTBP1",0.7039887785625,-0.04056778925,3.95642091565171,0.000233905426134503,0.00139026816670835,0.0780078640125481

"PCDHGB7",0.7882872203125,0.00584707224999999,3.9563233771098,0.000233978507612586,0.00139029920648685,0.0777111048218746

"NDRG3",0.70459278095,-0.11222584484,3.95605934245537,0.00023417644817624,0.00139107192526379,0.0769078036272921

"KRT19P2",1.2291594058,0.29655733284,3.95578781076533,0.00023438017772273,0.00139187857425603,0.0760817228019537

"PSTPIP2",-0.64378657605,-0.20354158484,-3.95382357279863,0.000235859045564397,0.0013998493990508,0.0701068104359415

"LINC01829",1.674491170375,0.3011347513,3.95300612988562,0.000236477146685542,0.00140296340439972,0.0676207340366393

"CCNE1",-0.864002062725,0.17100662282,-3.95294864056689,0.00023652067545864,0.00140296340439972,0.0674459028533967

"TRIM17",0.6660675988375,-0.04344589843,3.95236528051149,0.00023696281172519,0.00140467217431796,0.0656719180944361

"IGFBP3",-1.073981023975,0.11049895482,-3.952084390841,0.000237175986210488,0.00140522313634484,0.064817788128555

"ABCC6P1",0.77465078325,-0.0307448564,3.95114683506793,0.000237888862890595,0.00140863208754408,0.061967097842806

"PLXDC2",0.63269033015,0.01940022612,3.94789057296408,0.000240380887051323,0.00142256603521303,0.0520690218088138

"AC093724.1",-0.594931877125,0.121409237,-3.94420289563215,0.000243233506424879,0.00143820146598353,0.0408647700563876

"CDH1",-1.799028460075,0.25811781634,-3.94253888941616,0.000244531379204107,0.00144462482616313,0.0358108387904092

"PLGLB2",0.812593035025,0.09408474002,3.94230564983967,0.000244713832065031,0.00144528595887817,0.0351025319727496

"MAL",-1.0501191679,0.05667338668,-3.93706438444706,0.000248848662221341,0.0014675910741482,0.0191915931539475

"TUBB8",0.617414382875,0.0057854174,3.93657554454984,0.000249237727145183,0.00146946260539583,0.0177081903017955

"TNFRSF19",0.5961612338625,-0.02616103626,3.93439037553828,0.000250984079702027,0.00147933309117812,0.0110784066007961

"OMD",1.390004401475,-0.07851219062,3.93390052249772,0.000251377179676734,0.00148037242231013,0.00959246441172823

"FAM111B",0.622560249675,-0.03868748856,3.93272217736194,0.000252325219732384,0.00148552847755088,0.00601842117630369

"HLA-DMB",1.0245307363,-0.21398424396,3.93057811762098,0.000254059085176638,0.00149444804395606,-0.000483278012478827

"AJAP1",0.7284570189125,-0.02950905232,3.93003676806587,0.000254498680092326,0.00149617473679529,-0.00212458216209477

"ADAMTSL1",0.5858518755375,-0.01318505357,3.92513021719747,0.000258516586355548,0.00151805325917239,-0.0169951544787281

"C12orf54",0.7341788757625,-0.14295312389,3.92299587842267,0.000260283405420192,0.00152755268125148,-0.0234607381730418

"AC092718.4",-0.7221189929,-0.04953509332,-3.92288423648496,0.00026037614322509,0.0015276593420377,-0.0237988852407618

"ABCC6",1.188051357375,0.1393896129,3.92179194995066,0.000261285158050221,0.00153167677199798,-0.0271069909024639

"TLR4",-0.6558642687375,0.13617323011,-3.92096554813412,0.000261974933836749,0.00153484199154913,-0.0296095106232066

"CNPY4",0.6679532424,-0.04442583108,3.9205508283136,0.000262321751189489,0.00153643454498864,-0.0308652642521219

"IRX6",1.17797278165,0.24858464522,3.92037103960507,0.000262472240515022,0.00153687661150951,-0.0314096346843771

"EPSTI1",0.970092751875,0.2264406475,3.91997871301593,0.000262800920565256,0.0015383615030117,-0.0325974884553473

"C1orf43",-0.6909003520625,-0.08179327935,-3.91976055567362,0.000262983858318191,0.00153899265616198,-0.0332579796927508

"NCF2",-0.9789025815,0.0520387658,-3.91859291873438,0.000263965080736421,0.00154429371048766,-0.0367927740297125

"FOXD2",0.74539338385,0.01498335948,3.91844460954823,0.000264089964688128,0.00154458327049111,-0.0372417111575833

"CEACAM20",-0.834644341175,-0.08971845794,-3.9180821008551,0.00026439545558456,0.00154592867979922,-0.0383389996160375

"RCAN3",0.645124670825,-0.11480489109,3.91749765981781,0.000264888689754851,0.00154837074912907,-0.0401079478137154

"RFK",-0.646572677375,-0.0785424919,-3.91686612421664,0.000265422666721329,0.00155009683884866,-0.0420192806371968

"IP6K3",-0.80727657885,0.05460651432,-3.91625938123899,0.000265936658875543,0.00155184000770212,-0.0438554241999061

"LDLRAP1",0.61445168025,0.0582202246,3.91410357728949,0.00026777068937259,0.00156120827656961,-0.0503781545041351

"MMP2",1.10486064445,-0.01884233434,3.9137974680855,0.000268032096081646,0.00156184345407303,-0.0513041815587458

"ZDHHC15",0.6572877877,-0.01663716024,3.91363627148007,0.000268169851346657,0.00156220185225769,-0.051791810350065

"LEMD1-AS1",0.96905937245,-0.15065166564,3.91314943806723,0.000268586303727869,0.0015641831127847,-0.0532644436977456

"ACAP1",-1.20511079335,0.14595574332,-3.91294002950736,0.00026876563009822,0.00156475168683391,-0.0538978582794067

"TIMP4",-1.925232252925,-0.11692853734,-3.9128570960271,0.000268836681845733,0.00156475168683391,-0.0541487087126749

"SERPINE2",1.098454136675,0.03809234484,3.91260513720804,0.000269052654117599,0.00156556398113075,-0.0549107958033233

"GFI1B",-1.661213619575,0.12179542034,-3.91199959814414,0.000269572390391119,0.00156769773895352,-0.0567422319895403

"ZNF600",0.619521870725,0.0914538351466667,3.9089540467993,0.000272201114268631,0.00158074161823575,-0.06595112136635

"TSPEAR-AS2",0.989494620875,0.0986886017,3.90876560705948,0.000272364572906057,0.00158124266639254,-0.0665207841173903

"CDC25C",-0.9614925024,-0.04686551392,-3.90854871350467,0.000272552830475265,0.00158188736282641,-0.0671764459951252

"COLEC11",0.9241293918,0.12548319744,3.90671280363319,0.000274151383762707,0.00158981419488546,-0.0727255598808156

"C8orf34-AS1",0.896455275575,0.12596743646,3.90563718563561,0.000275092136298098,0.00159346556077903,-0.0759760119012203

"CARS1",-0.649814326475,-0.01191926638,-3.90553093882107,0.000275185230201194,0.00159355426691975,-0.076297057300363

"LRRC9",0.88993939125,-0.013648635,3.90461183815771,0.00027599181916467,0.0015977734928075,-0.0790741034052687

"LRP11",0.672838116175,-0.02789940146,3.90435340209839,0.000276219029113175,0.00159863713798608,-0.0798549002665254

"RRP9",-0.789204219075,0.16682207224,-3.90129872364729,0.000278918322143818,0.00161243752372532,-0.0890817132947639

"HTR7",0.629761734325,-0.08807380854,3.90000885008478,0.000280065751836996,0.00161828551439549,-0.0929766801664682

"DSEL",0.79783963585,-0.05917349082,3.89988666281935,0.000280174681353211,0.0016183306657921,-0.0933456071447694

"RAD51-AS1",1.1307089025,-0.152736933,3.89911631658513,0.000280862383527044,0.0016215472160691,-0.0956714146923607

"FAM217B",0.64995312585,-0.11459694432,3.89890341381937,0.000281052732597118,0.00162157374977464,-0.0963141612224669

"C14orf178",0.710814007725,-0.11986717282,3.89650856256883,0.000283202473811184,0.00163305721459148,-0.103542841648044

"CRIM1",1.0111135354,-0.01473545818,3.89411057671029,0.000285370908624032,0.00164371076072229,-0.110778593692817

"C15orf65",0.98566808565,-0.09759595658,3.89309097233952,0.000286297751674818,0.0016476452014209,-0.113854452221138

"AC005082.1",0.730977277025,-0.16655251648,3.89203528598651,0.000287260451619215,0.0016522717947149,-0.117038703600103

"FAM53A",0.783273161875,-0.0868398175,3.89025419465669,0.000288891739749654,0.00166072221211866,-0.122409930498738

"C1orf61",1.1836455294,0.26259034722,3.8886864152472,0.000290335043693835,0.00166808311138511,-0.127136778963216

"CPLX1",0.8724104489,0.10369332372,3.88707400946,0.000291826680016322,0.00167571329040762,-0.131997105611324

"SLC9A5",0.655736635075,0.05713458956,3.88489080912503,0.00029385813448323,0.00168548865041781,-0.13857626579887

"HMCN1",1.252502482025,0.18744300702,3.88379293863023,0.00029488483981662,0.00169090416965097,-0.141883988808304

"THBS1",-1.0506991897,0.16890957474,-3.8816123005821,0.000296934389428255,0.00170218012607893,-0.148452436224129

"DGKE",0.7793555629,0.12915805032,3.88135911077214,0.000297173246449083,0.00170307285965002,-0.149214957396723

"AP003119.2",0.6286361209,-0.03421382908,3.87940975641059,0.000299018461196429,0.00171173239256564,-0.155084847166948

"GBE1",-0.71027686515,-0.02481977152,-3.87866925429544,0.00029972229346279,0.00171480322492758,-0.157314227428346

"NLRP12",-1.36565127545,0.22306107964,-3.87763674694403,0.000300706336276527,0.00171899314108077,-0.160422344516062

"PEG3",1.56027906295,0.19788680336,3.876341618997,0.000301945075081495,0.00172511173961786,-0.164320385888679

"PRRG4",-0.6874044271,-0.06797145038,-3.87487894345187,0.000303349979179281,0.00173120734635797,-0.168721860870297

"LOXL1",0.811950034325,-0.06092200654,3.87305729528868,0.000305108486243203,0.00173930513804974,-0.174202297580202

"RAPGEF3",0.622435176975,0.0915136218133333,3.87218896149323,0.000305950175226231,0.00174264935373827,-0.176814192794063

"LSM4",-0.6215633586,0.00236785052,-3.87160690769096,0.000306515620653131,0.0017453841122683,-0.178564797692166

"LALBA",-1.933120731925,0.07356017706,-3.87141226475885,0.000306704934179969,0.00174597685231431,-0.179150180625942

"AURKB",-1.275261053175,-0.03959174254,-3.87084171139541,0.00030726051488564,0.0017481681280136,-0.180866011357002

"DCSTAMP",1.47708556225,0.085294122,3.87055831475625,0.000307536834835677,0.0017492544897594,-0.181718221931807

"SLITRK5",-1.15634631375,0.0139068226,-3.87022553512398,0.000307861610272581,0.00175061578441983,-0.182718890441239

"PTGFRN",0.9150852076,-0.03762139372,3.86845892261021,0.000309591272186864,0.00175947461430359,-0.188030314302305

"AC111170.4",0.843768708825,-0.01516952594,3.86785514163698,0.000310184568245225,0.00176235758020193,-0.189845315691429

"TXN",-0.731456405225,-0.02599563818,-3.86737816564374,0.000310654035651205,0.00176404653060474,-0.191279025179697

"FLNB",0.73691499825,-0.0392735484,3.86693983330411,0.000311086071264481,0.0017660103707583,-0.19259649379963

"GZMA",0.9650167976,0.02467522508,3.86069031155736,0.000317309129407902,0.00179636071934487,-0.211371479728783

"BPIFB2",-0.782496241,0.0757330117,-3.86017356143761,0.00031782902491756,0.00179711637639328,-0.212923176216997

"LOXL2",0.85133473705,0.15189193564,3.86012843588908,0.000317874464072125,0.00179711637639328,-0.213058673785879

"AC110771.1",0.66371108335,-0.11248334732,3.8598385398732,0.000318166524013293,0.00179823330868534,-0.213929118133007

"TTR",-1.396823094075,0.00804812273999999,-3.85966703263877,0.000318339433132553,0.00179871437011025,-0.214444070540508

"RGS4",0.9009943576,-0.06955780892,3.85891170516576,0.000319102012226842,0.00180252606189786,-0.216711801730624

"CD59",-0.603946225275,0.09792383178,-3.8584092213904,0.000319610294427189,0.00180489958991848,-0.218220282718507

"GPR18",0.98624921195,-0.13150380824,3.85570919428059,0.00032235485882192,0.00181915978607459,-0.226324066346103

"AL033523.2",-0.64727729595,0.06248394224,-3.85566315485427,0.000322401853926046,0.00181915978607459,-0.226462220978148

"MN1",1.086888703375,0.0711660667,3.85340065310794,0.000324719467420048,0.00182786256178337,-0.233250405648168

"BNIP3",-0.901349428125,0.0912928585,-3.85333192827462,0.000324790116788174,0.00182786256178337,-0.233456566815961

"LPAR1",0.806074618975,-0.00422096482,3.85328694098174,0.000324836371898343,0.00182786256178337,-0.233591518882307

"CCL20",-1.2177512801,-0.00240121607999999,-3.85247599228449,0.000325671263173436,0.00183155444411127,-0.236024040846448

"HOXA11",0.627193891025,0.00797726682,3.8523879225014,0.00032576205743959,0.00183156230318944,-0.236288198196415

"LYPLAL1",0.720686967325,-0.14597959374,3.84975235497815,0.000328490465283365,0.00184488470172937,-0.244191820805556

"PCDHB9",0.80471585425,-0.1058687286,3.84975109932469,0.000328491770397732,0.00184488470172937,-0.244195585595397

"LINC00343",0.656736904325,-0.03955239954,3.84853461903593,0.00032975851695591,0.00185075536942687,-0.247842608877665

"SKIL",0.637691664275,0.13808583352,3.84850959566272,0.000329784623631397,0.00185075536942687,-0.247917622664136

"FCGR3A",-1.012145420875,0.0378313816,-3.84840055385267,0.000329898409271131,0.00185075536942687,-0.248244499509245

"LILRB2",-0.90251855125,-0.1131302257,-3.8465727466088,0.000331811371803976,0.00186046835947451,-0.253723001557062

"AC136944.2",-0.919681197275,0.23122897168,-3.84583051615055,0.000332591227194406,0.00186382085961679,-0.255947289369629

"ZNF566",0.605029352925,-0.00348285666,3.84541908285914,0.000333024276696224,0.00186522727746097,-0.257180155882629

"PCDHGA8",1.0186283783,-0.08273680456,3.84409332680615,0.000334423382617736,0.00187196299694689,-0.261152315002746

"PGAM1",-0.635042931875,-0.0240824885,-3.84396858981243,0.000334555311811752,0.00187196299694689,-0.261526006755817

"PLSCR1",-0.6706082453,0.03040508036,-3.84303120779341,0.0003355483467052,0.0018757747687029,-0.264334038775775

"TTC16",0.7118132471375,0.07404554201,3.83945269331565,0.000339365479045177,0.00189556159614983,-0.275050431398673

"SLAIN1",-1.224532892475,-0.12903684298,-3.83934961637389,0.000339476046277128,0.00189566237016239,-0.275359029636411

"DAPL1",1.43047928455,0.10632595834,3.83685008099212,0.000342167856352914,0.00190845238810779,-0.282840906879989

"A2ML1",-1.152888844125,-0.1863120623,-3.83671147293781,0.000342317726439846,0.00190845238810779,-0.283255725249088

"ELN",0.60728608175,-0.0304427751,3.83670438971762,0.000342325386875054,0.00190845238810779,-0.283276923290527

"DUSP13",-0.924788533125,0.0224478238,-3.83644211486806,0.000342609150834405,0.00190951476667445,-0.284061821405862

"C1RL-AS1",0.74746364025,0.01296722755,3.83589071842714,0.000343206463728602,0.00191232364124874,-0.285711864768249

"FAM155A",1.204034442425,0.10337709294,3.83439147776969,0.000344835623147959,0.00192087880561593,-0.290197656526546

"ETF1",-0.8579569042,-0.10192834636,-3.83413545051025,0.000345114579913135,0.00192138791121204,-0.290963604839458

"MORN4",0.6383797564625,0.12112333967,3.83267418372029,0.000346710878495682,0.00192870281797978,-0.295334691493408

"STAT3",-0.5959201971125,0.06393642811,-3.83255544574696,0.000346840900468573,0.0019289023802389,-0.295689832176278

"CYP19A1",-1.04429059834167,0.110335642493333,-3.83086463460104,0.000348697494185042,0.00193817532850329,-0.300746327592924

"MALRD1",0.632598279333333,-0.1291368762,3.83065866474195,0.000348924311706169,0.00193891003478058,-0.301362211946993

"CRYZ",0.7140852478625,-0.09214940111,3.82987433473551,0.000349789329887758,0.00194318974803156,-0.303707323477569

"TRIM10",-1.284598910025,0.14785121798,-3.82948865085594,0.000350215447728219,0.00194502957252799,-0.304860404238807

"RNF43",0.83181881225,-0.1458496592,3.82930060811507,0.000350423385443823,0.00194565699755367,-0.305422573301724

"MYOZ1",0.72017103305,0.02495269744,3.82880360186985,0.000350973547300318,0.00194818370010537,-0.306908340867985

"C21orf58",-0.760908381975,0.06339939902,-3.82776065559426,0.00035213074208995,0.00195258540017091,-0.310025815829993

"TM4SF20",-0.667220686325,0.11967103194,-3.82725418214446,0.000352694020338664,0.00195508525127125,-0.311539549301214

"FEZ1",0.62090445055,-0.01056851156,3.82634923970752,0.000353702618379106,0.0019590860355099,-0.314243941410831

"NCAPD3",-0.6424521535,-0.0459698988,-3.82488568720922,0.000355339688961258,0.00196708985340131,-0.318616977795352

"AC017116.2",0.793653629075,-0.03998306014,3.82471656714999,0.000355529329102554,0.00196760802124612,-0.319122242457934

"MCCC2",-0.7417324786,0.19033365212,-3.82395927507989,0.000356379701881957,0.00197124927974016,-0.321384584525693

"SLC25A22",-0.70944944565,0.09457518548,-3.82375515788271,0.000356609242309194,0.00197198654694488,-0.321994324146609

"LSP1P4",0.8924362612,-0.05385046064,3.82348208403045,0.000356916550577712,0.00197263021854473,-0.322810023320578

"PIP",0.7845896535,-0.0940784452,3.82348061295938,0.000356918206762267,0.00197263021854473,-0.322814417471615

"PLA2G4A",0.776404139875,0.0198794849,3.82332515392083,0.000357093269811884,0.00197306551022273,-0.32327877489792

"BTC",1.61685478745,0.36850552796,3.82071715585147,0.000360042495960695,0.00198766276745914,-0.331067336350039

"MSANTD3",0.7807689273,-0.08379540116,3.82064607559612,0.000360123203475611,0.00198766276745914,-0.331279570427982

"ZCCHC12",0.7833294872,-0.07911877724,3.81861080134338,0.000362441538730121,0.00199830523291247,-0.337355645042629

"TST",-0.6225781716,0.07001464772,-3.81754522876215,0.000363661028848302,0.00200395028484239,-0.340536076210467

"CPOX",-0.828233079025,0.11826137578,-3.81553761445207,0.0003659693686408,0.00201558613567546,-0.346526903813423

"KEL",-2.432000589425,0.13605491306,-3.81535655261725,0.000366178243776324,0.0020161945333215,-0.347067116448801

"SLC3A2",-0.7385208112,-0.08971838956,-3.81407103952499,0.000367664528584526,0.00202220425815833,-0.350902141880447

"FAR2P4",0.6780009399375,0.0673036673,3.81272738700209,0.000369224235718856,0.00202969308865251,-0.354909849831704

"GPR183",-1.09609955885,0.17764524792,-3.81247134144877,0.000369522173661402,0.00203078602306191,-0.355673467581902

"AMTN",1.8387219895,0.5812176236,3.81216086649406,0.000369883756618734,0.00203168321866075,-0.356599374914471

"TNFAIP8L1",0.666724853575,0.01423095846,3.81064229152013,0.000371657216907438,0.00204033040192915,-0.361127512498416

"RRM2",-1.4160157037625,0.12394992449,-3.80930795773466,0.000373222260712703,0.00204837333980227,-0.365105450366809

"APOL4",-0.640262486766667,0.11461466192,-3.80752597643251,0.00037532224036657,0.0020582446629096,-0.370416720700551

"PLGLA",1.062903863575,0.01421835886,3.80670197874683,0.000376297120508811,0.00206303864195465,-0.372872215568273

"DDR1",0.924865442,-0.0280412974,3.80650533846705,0.000376530127361034,0.00206376384413399,-0.373458155776166

"VHL",0.732884952075,-0.05311140064,3.80621448055466,0.000376875030872402,0.00206502925813054,-0.374324810893847

"TPBG",1.088000313675,0.08946039394,3.80503386436065,0.000378278145199162,0.00207159245734233,-0.37784225883307

"FANCI",-0.938302193425,0.24935385626,-3.80496268937996,0.000378362894151454,0.00207159245734233,-0.378054293438167

"RPF2",-0.6957813931,0.06382394752,-3.80456640273062,0.000378835091686757,0.00207329898401007,-0.37923481535288

"MST1L",0.93199907325,0.0630991376,3.8019763264135,0.000381935298067539,0.00208780426542362,-0.386948870234386

"ABLIM1",0.622511166416667,0.0551295579,3.80149216441674,0.000382517518928841,0.00209001327428463,-0.38839053259905

"UAP1",-1.078852435975,0.15065869322,-3.8014215491582,0.000382602507410432,0.00209001327428463,-0.38860079123756

"AXDND1",0.8259922318,-0.01213498116,3.80138588235408,0.000382645440835692,0.00209001327428463,-0.388706989185458

"PTGFR",0.976446443483333,-0.01765844678,3.80092570591925,0.000383199787582118,0.00209203325855471,-0.390077115466692

"KAT2A",0.740602064325,-0.05870899954,3.80090936597208,0.000383219485521077,0.00209203325855471,-0.390125764226571

"LINC00654",0.861681801325,0.09519533206,3.80026351893907,0.000383998839712357,0.00209461347924036,-0.392048545184378

"AC118758.1",0.7064581821125,0.10126378344,3.79987082725621,0.000384473452088458,0.00209664415394952,-0.393217557084452

"SET",-0.61643705065,0.09229327938,-3.79738380661971,0.000387492423721559,0.00211254517754319,-0.400619662904178

"CACNB1",0.6047829036,0.00402061438,3.7967992443602,0.000388205320098841,0.00211586874120379,-0.402359101844612

"SEPTIN7P2",0.89897588215,-0.07875312278,3.79565483459245,0.000389604625292617,0.00212180211669195,-0.405764007268585

"HMGB3P30",-0.778855248525,0.15720083418,-3.79452558666251,0.000390990144868474,0.00212821628269535,-0.409123243455851

"BMP8B",0.61175310625,-0.029761315,3.79356810852429,0.000392168623533536,0.00213293091557077,-0.411971071363484

"MAN1B1",0.60115019345,-0.02860614884,3.79311937191194,0.000392722110058074,0.00213537436063424,-0.413305611491187

"FBXO5",-0.8000922593,0.12643105656,-3.78603786496628,0.000401556753142764,0.00217705603556204,-0.434354338590156

"TMEM67",0.946604312825,-0.00702820474000001,3.78540476936243,0.000402355819464767,0.00218081111883443,-0.436235054322509

"NUF2",-1.423439646175,0.13253043606,-3.78473414043629,0.000403203925800102,0.00218482994757802,-0.43822707780493

"SAMD5",0.662926843475,0.01776199358,3.78432211258845,0.000403725845974606,0.00218707962250865,-0.439450859944889

"PCDHB16",0.701799857,-0.1064478508,3.78119819902921,0.000407704122343789,0.00220688033251771,-0.448726915802372

"F3",-1.037279099275,0.06642887058,-3.78085690139403,0.000408141038091067,0.00220866180359477,-0.449740095791799

"TIAM1",0.700718445,-0.0340927697,3.77837805733636,0.000411327887555099,0.00222297171200973,-0.457097287546043

"RNF123",-0.814103718725,0.12193577122,-3.77754543596911,0.00041240367706794,0.0022270233357322,-0.459567897143748

"CDHR4",0.7252210031625,-0.02926571807,3.77374611714617,0.00041734700721442,0.00225253042249974,-0.470837624971296

"FAM162B",0.66871509245,-0.09364516124,3.77357057295697,0.000417576780276922,0.00225261566146997,-0.471358179526253

"FAM131B",0.81910093915,-0.16325443568,3.77195334760589,0.0004196993231805,0.00226235545563087,-0.476153223440241

"APLN",0.6974287421,-0.10399486612,3.77193801314086,0.00041971949857736,0.00226235545563087,-0.476198684344778

"ZBTB14",0.657497961775,-0.01981532928,3.77109902650754,0.00042082476608966,0.00226712011770838,-0.478685805514772

"PPP1R27",-1.506239095625,0.245026246,-3.77077393866784,0.000421253782926935,0.00226867701556195,-0.479649423930211

"CYP4F12",1.23383764345,-0.21520038434,3.77071226348905,0.000421335222774952,0.00226867701556195,-0.479832234923953

"KLF9",-0.855863520475,0.11132739792,-3.76870919789212,0.000423988438355438,0.00228116468619386,-0.485768597802449

"PCDHA9",0.87928102455,-0.16271216206,3.76851518818514,0.000424246270297404,0.0022818127082564,-0.486343478596685

"ASF1B",-0.721625946925,-0.03795463894,-3.76646360176737,0.000426981990743989,0.00229364329189497,-0.49242163124312

"CERCAM",1.043331318875,0.0313962816,3.76342909044574,0.000431059504239026,0.00231160494885317,-0.501408451393043

"AC073349.2",-0.6914315257,-0.07057152006,-3.7629591590113,0.000431694293093783,0.00231378802720311,-0.502799807541259

"CRISPLD2",-1.2020536009,0.07497643128,-3.7622408264461,0.000432666360958707,0.00231667910095344,-0.504926432289513

"LINC00994",0.89593493145,0.18866557316,3.76218813665055,0.000432737744989693,0.00231667910095344,-0.505082411540209

"GNG4",1.4838613558625,0.27346276069,3.75875824631057,0.000437408971723104,0.00233904315027208,-0.515233382345114

"PALM3",0.650802466775,-0.04783803883,3.75870197645263,0.000437486008973185,0.00233904315027208,-0.515399873047325

"DLAT",-0.694719116775,0.10034000428,-3.75834806267297,0.000437970839780742,0.0023403677702326,-0.516446997526872

"ARMC4",0.785598024175,-0.13993086346,3.75818265297924,0.000438197613475497,0.00234040477186808,-0.516936376070411

"H2AC13",-2.35914643775,0.3984108048,-3.75806395140214,0.000438360420694713,0.00234066413843974,-0.51728755724195

"GTSF1",-0.8244111512,0.15729263964,-3.75773687388377,0.000438809330251375,0.00234245064048728,-0.518255190842258

"ATAD2",-0.771903295625,0.1278518475,-3.75623836290185,0.00044087166867079,0.0023516216474166,-0.522687814127385

"RUFY4",0.72232240855,-0.02516913371,3.75564529918763,0.000441690446311463,0.002354762910234,-0.524441832797203

"GRIA2",1.0326810887,-0.16240619884,3.75550875883004,0.000441879159249933,0.00235515614326551,-0.524845636364725

"AL845472.1",-1.2731499463,-0.04451139254,-3.7504464452841,0.000448930718774283,0.00238529371531315,-0.539811052305859

"TGFB3",1.2370084584,0.36107874172,3.74776902705548,0.000452703799529074,0.00240160420630545,-0.547721542791386

"NIPSNAP1",0.635730824,-0.0079726504,3.74682495493619,0.000454041455459594,0.00240683087696149,-0.550510064273229

"PTBP1",-0.772269486225,-0.14169263968,-3.74664070962393,0.000454302954932717,0.00240697153883152,-0.551054226461765

"BAIAP2-DT",0.714143746125,-0.1338911039,3.74528079928203,0.00045623755437977,0.00241534754887151,-0.555070207529631

"REP15",2.039651563225,0.31806697358,3.74200893671289,0.000460924548767423,0.00243701216907019,-0.564729041735646

"RAB33B",0.89044167565,-0.20261552818,3.74102321192338,0.000462345653516919,0.00244326483086269,-0.567638055054831

"AC092587.1",0.6491060586,-0.15156599932,3.73988046781465,0.000463998395933662,0.00245073450267823,-0.571009912048319

"DNAJB9",-0.7935730633,0.04386747636,-3.73874216552208,0.000465650352379384,0.00245882588132701,-0.574368083260017

"CASR",0.75889754565,-0.06946544448,3.73828741748164,0.000466311880730224,0.00246168456902882,-0.575709499931811

"LINC02593",0.71002155395,0.07316436656,3.73812155210849,0.000466553391893201,0.0024623250626244,-0.576198746950787

"TPM1",0.70137797795,0.11522692806,3.73752347456369,0.000467425230623563,0.00246629104429966,-0.57796277233879

"ROBO2",0.81060515105,-0.02457167616,3.73607922927092,0.000469537011119765,0.0024767956446503,-0.582221904345025

"BPGM",-0.9544856809,0.14046514828,-3.73218261568058,0.000475280441289634,0.00250515710860356,-0.593708505965577

"MAPK10",0.674116780575,0.18943995666,3.73192872057961,0.000475657001889017,0.00250649708197073,-0.594456712789619

"TMEM156",-1.3160321966,-0.04175526898,-3.73148822520715,0.000476310995297391,0.00250929793562689,-0.595754745913575

"AL136964.1",1.33662956495,0.07028621396,3.73122671457202,0.000476699662351521,0.00251069991831824,-0.596525313267132

"EAF2",-1.008243648325,0.22073857134,-3.73004549117667,0.000478459037513881,0.00251867131566916,-0.600005524824594

"DUXAP8",0.762187668325,-0.06343554234,3.7298035187132,0.000478820212108152,0.00251965673299555,-0.600718365600672

"F11R",-0.8640917067,-0.15143514736,-3.72936773144579,0.000479471339991785,0.00252087132811722,-0.602002110488814

"LARP1BP1",-0.7042529525,0.110432873,-3.72866038657264,0.000480530022909354,0.00252567960732859,-0.604085630194431

"TMEM132B",0.777871324675,-0.03345464676,3.72855219629107,0.000480692148968386,0.0025258837517477,-0.604404290294086

"ABCG8",0.738361745625,0.0355698381,3.72842945651597,0.000480876141398928,0.0025262026628157,-0.604765797636628

"SP6",1.057639215175,0.10113375914,3.72727967646851,0.00048260299863645,0.00253462451578149,-0.608151934230326

"C1QL4",0.747291773075,-0.14538256254,3.72681241641394,0.000483306476967686,0.00253766865712813,-0.609527859511349

"LMAN2",-0.608695173825,-0.09353219906,-3.72669170975541,0.000483488365462166,0.0025379732594386,-0.609883284501009

"ZBTB7C",0.701353695775,-0.01964171558,3.72633789875348,0.000484021889020037,0.00254004760334492,-0.610925055718898

"TMEM72-AS1",0.904534143575,-0.03714576814,3.72626526466908,0.000484131486287675,0.00254004760334492,-0.611138914637751

"Z97200.1",0.82454745685,-0.06718874752,3.72504565693344,0.000485975308412574,0.00254711233531768,-0.614729491836261

"FBXO39",0.8697202713,0.06621541104,3.72405867668544,0.000487472372213516,0.00255365225310931,-0.617634714597211

"AL358942.1",-0.627599029525,0.05869443828,-3.72316635323236,0.000488829667856537,0.00256010793329364,-0.620260933785778

"YWHAH",-0.59063832375,-0.043250308,-3.72200038724496,0.000490608661774071,0.00256811197302687,-0.623691977525578

"DDX17",0.6074244749375,-0.02076711405,3.72190749090194,0.00049075066696032,0.00256819914806718,-0.623965313759222

"HTR1F",1.0891602764,-0.01681140988,3.71930011242023,0.000494752540591031,0.00258649912008907,-0.631635624743275

"BCL3",-0.822584287825,0.10580957574,-3.71882232013575,0.000495489255501064,0.00258902929525779,-0.633040848488223

"HOXA5",0.616043589675,-0.09442089176,3.71841814736517,0.000496113277080927,0.00259096834586644,-0.634229471308036

"CRABP2",0.942212570625,-0.165703628,3.71823153440773,0.000496401652094057,0.00259181372275816,-0.634778252418011

"KLHL2",-0.64259923765,0.05068091288,-3.7173505966622,0.000497765147667378,0.00259827066125076,-0.63736865373142

"TRBV10-3",0.8577295126,-0.14190372392,3.71686630972108,0.000498516245984288,0.00260020388180399,-0.638792552603456

"KRT27",0.7308805615,-0.0064242749,3.71631033681462,0.000499379865419139,0.00260404547790973,-0.640427091933041

"SEC23B",-0.68848580575,-0.1561127178,-3.7145386308975,0.000502141526869789,0.00261711411918297,-0.645634910291895

"DUSP4",-0.67818840125,0.1690274622,-3.71066454836449,0.000508231422707287,0.00264683410992041,-0.65701759552057

"SOCS2",-0.645499642825,0.15237451664,-3.71052028906531,0.000508459555012416,0.00264707074755569,-0.657441321865625

"PAXIP1-AS2",0.9556078282,-0.00758719364000001,3.71047242907866,0.000508535262708863,0.00264707074755569,-0.657581896771252

"AC123767.1",1.0911113485,-0.0754820444,3.70897374304099,0.000510911449289834,0.00265741400686726,-0.661983330635985

"LINC02052",-0.873809344975,-0.06873560998,-3.70753631145791,0.000513200519651515,0.00266796555357022,-0.666203913921146

"SPAG5",-1.2019961907,0.22473337644,-3.7066102834981,0.000514680400267508,0.00267290416352041,-0.668922419706355

"RAC2",-0.619377811283333,0.03489491074,-3.70489992297887,0.000517424486847394,0.00268515523974909,-0.673942440175711

"ITGA11",0.9813732851,0.19627874108,3.70377658175153,0.000519234393695939,0.00269386534769369,-0.677238799168948

"AEN",-0.663524601375,-0.081733733,-3.70333581662232,0.000519946203673271,0.00269619281722551,-0.678532034471548

"RND2",0.742315578475,-0.03102260622,3.70274191280824,0.000520906803882903,0.00270049053591926,-0.680274449892186

"EDIL3",1.14796413735,0.13755989828,3.7016224279021,0.000522722126524501,0.00270853083668032,-0.683558398072835

"AC009060.1",1.107940322275,0.30739412482,3.7015192545764,0.00052288973424506,0.00270871425416252,-0.683861022798336

"ACSL5",-0.585343702225,0.07564391762,-3.7008064060167,0.000524049184065589,0.00271403429806263,-0.685951795550024

"SUN1",0.598595591591666,0.00808264587333333,3.70034192170216,0.000524805995065349,0.00271726692618117,-0.687313996037031

"CXCL10",1.736845676125,-0.2928266416,3.6990505213487,0.000526915659830694,0.00272612324207355,-0.691100791316851

"CICP26",0.834596502475,0.05881771098,3.69893465126915,0.00052710534522991,0.0027264161355896,-0.691440522087707

"AZGP1",-1.2100856132,0.25217300614,-3.69723942439702,0.000529888018596481,0.00273942612288789,-0.696410225133742

"TSC2",0.6423967983875,0.07361294571,3.69550252607679,0.000532753701766923,0.00275146403877003,-0.701500736193048

"AC005674.2",0.691478843725,-0.10947305762,3.6941158568383,0.00053505220860735,0.00276124676321093,-0.705563808442241

"PI15",-1.409490839375,0.2205292985,-3.69258721648192,0.000537597060603842,0.00277298302559202,-0.710041854860854

"AC012236.1",-1.39972940445,0.08813236944,-3.69055275712825,0.000541001995564489,0.00278914164195401,-0.716000011474247

"CDCA8",-1.1702336174,0.17878965008,-3.68969247664213,0.000542447999049766,0.00279518978987214,-0.718518877612785

"IL18R1",-0.803994185216667,0.120565372226667,-3.68648362329436,0.000547874357695482,0.00282031403026759,-0.727911289636246

"MFAP2",0.882919505975,0.07203075378,3.68591109310097,0.000548847994156578,0.00282390700760421,-0.729586608689449

"LRRC38",0.665683273425,-0.03958954976,3.68574625947964,0.000549128615149727,0.00282464149314276,-0.730068911650548

"KCNQ3",-0.62345713545,0.06095843204,-3.68483291552061,0.000550686031587635,0.00283194162027296,-0.732741129840001

"SIGLEC17P",0.5992150297,0.00683604176,3.68445951470402,0.000551323965968676,0.00283451076907559,-0.733833498313241

"CCBE1",0.796987875475,-0.09172121882,3.68349863491777,0.000552968833653608,0.00284168102558359,-0.736644218793383

"MGP",1.0451029234,-0.15717478728,3.6830380230426,0.000553758992052841,0.00284488822196053,-0.737991429295674

"RCC1",-0.9438415143,-0.07016687224,-3.68216394178884,0.000555261414438147,0.00285189166683599,-0.740547699697764

"ERC2-IT1",0.686935515775,-0.05369092658,3.68163325045417,0.000556175503274099,0.00285587060428064,-0.742099547906224

"TAS2R46",0.90817370215,-0.01347721928,3.67997281777049,0.000559044839124952,0.00286916599799399,-0.746954154095193

"POU2AF1",-1.66188540725,-0.2183383178,-3.67931153203357,0.000560191529623684,0.00287361143188033,-0.748887203212032

"PRG4",2.287916474625,0.6351142897,3.67668635354945,0.000564765947856878,0.0028949023616942,-0.756559062713374

"ZIC2",-1.093757378975,0.19799698182,-3.67551734682414,0.000566814461603366,0.00290322367233244,-0.759974367563784

"RABGGTB",-0.606252874375,0.1073968893,-3.67504156653975,0.000567650234182612,0.00290598191691305,-0.761364201114846

"PIEZO2",0.802708510975,0.04751343748,3.67412745713617,0.0005692593064346,0.0029128333342238,-0.764034175435735

"FAM133A",0.95712453915,-0.14441576368,3.67281680936352,0.000571574017842181,0.00292321729344748,-0.767861708245198

"PCDH10",0.69487649155,0.15742695304,3.67088538475204,0.000575001516202034,0.00293781323290456,-0.773500679450245

"ROPN1",0.8456000417875,0.03221468043,3.66909430257506,0.000578197555133963,0.00295119868200913,-0.778728374761257

"TCP1P3",-0.9269570367,-0.15742284806,-3.66716782967217,0.000581654184089864,0.0029673632777971,-0.784349591465986

"RPL31P11",0.795322515725,-0.03497116542,3.666851777722,0.000582223156369014,0.00296952651921542,-0.785271629902427

"CMTM2",-1.7297041241,0.19126962672,-3.66503493022387,0.000585504291456965,0.0029855181491713,-0.790571141619514

"KLHDC1",0.637205779425,0.02344373554,3.66318889205781,0.000588856282502479,0.00299962394726773,-0.795954239795582

"MDK",0.803422860975,0.07425921978,3.65999657235864,0.000594696242551401,0.00302036027286792,-0.805259420100177

"C5",0.7146113283,-0.09162680436,3.65997996666827,0.000594726765182759,0.00302036027286792,-0.805307811135903

"NFATC2",0.679947789175,0.01589438609,3.65995039233552,0.000594781128966555,0.00302036027286792,-0.805393994084075

"LINC01096",0.79770284555,-0.23286805356,3.65992881786294,0.000594820790343621,0.00302036027286792,-0.805456864280583

"LOXL3",0.9694107978,-0.14992708776,3.65991646324541,0.000594843503564137,0.00302036027286792,-0.805492866789546

"CCDC68",0.655524716125,0.0248237999,3.65964573055128,0.000595341437930967,0.00302213958878336,-0.806281789427209

"PLXNA2",0.99713912075,-0.0537146859,3.658297246361,0.00059782753625387,0.0030332567020231,-0.810210805387056

"PRPH2",1.0060531171,0.10024330718,3.65498930781287,0.000603968315620415,0.0030598671736971,-0.819845430220757

"LINC00526",0.735899743675,-0.12847992166,3.65360423044724,0.000606557442755736,0.00307070642134409,-0.823878069601944

"C9orf72",-0.6275403899,-0.01088690092,-3.65127745933804,0.000610930821503654,0.00308826811521512,-0.830650439941602

"HSD3B7",0.588430330725,0.09999784618,3.65070433178692,0.000612012692866582,0.00309221111009878,-0.832318216961481

"PTTG2",-1.122214634675,0.23030142526,-3.64859816606604,0.000616004202250061,0.00310701479460838,-0.838445761101752

"ATG2A",-0.6053078417625,-0.03533169066,-3.64570406647104,0.000621529644208471,0.00313088434480273,-0.846862309355338

"SERPINB2",-1.158793389625,0.08972670395,-3.64326516422775,0.000626222832658399,0.00315157292937983,-0.853952040501717

"GAB2",-0.7240610365,-0.0416074962,-3.64021046641871,0.00063214886791651,0.00317905400242353,-0.862827943915165

"STC1",-0.888719955875,0.0487386403,-3.63972278391657,0.000633099914825825,0.00318305547360773,-0.864244579476896

"UGT3A2",-1.078378605075,-0.02525522906,-3.63826450637874,0.00063595193128644,0.00319582613887579,-0.868479968723447

"RANBP3L",0.660436038325,-0.00257510133999999,3.63815922166155,0.000636158316466302,0.00319607571046051,-0.868785716959124

"LINC02796",0.76352887245,-0.15393950504,3.63732398762451,0.000637797863621518,0.00320196094826701,-0.871211064614132

"RPN1",-0.611512663525,0.03211898768,-3.63691925462872,0.000638593800183156,0.00320438642619459,-0.872386208813097

"ZNF454",0.87630633315,0.07883278762,3.6367827531436,0.000638862454505116,0.00320451605736315,-0.872782524292419

"SMR3A",-0.819778025875,0.0056677283,-3.63674719646146,0.000638932452866481,0.00320451605736315,-0.87288575737182

"SLC4A11",0.68703206885,0.08124735008,3.6362573233822,0.000639897584933658,0.0032077860827308,-0.874307964606277

"MEGF9",-0.759899229125,-0.0508526499,-3.63599222981268,0.000640420445399368,0.00320932358488684,-0.87507754171676

"MAP3K5",-0.6597846967375,-0.04526893684,-3.63594300577236,0.000640517578026273,0.00320932358488684,-0.875220437439792

"HSPA5",-0.6640116084,0.10833757068,-3.63559909783003,0.00064119659688293,0.00321194031220965,-0.876218758993177

"PRMT8",0.79235891955,0.07162716964,3.63526167258766,0.000641863485736893,0.00321449501241199,-0.877198208406403

"SCML2",-0.983573772225,-0.15434568578,-3.63458474603934,0.000643203368929291,0.00322041804071929,-0.879162973107057

"SLCO2A1",0.872265199,0.024759939,3.63432498702158,0.000643718236625386,0.00322142140497824,-0.87991686122174

"ACSL6",-2.513883436525,0.56849768378,-3.63386327293483,0.000644634372777107,0.00322521832221181,-0.881256797563572

"GRXCR2",0.99877572075,0.1805437086,3.63334905962892,0.000645656146967701,0.00322954178200055,-0.882748974166586

"TTC23",0.981376206,0.0331935108,3.63216462200753,0.000648015599306164,0.00323818136550846,-0.886185579717226

"VSIG4",-1.015299993925,0.13490088786,-3.63138521992023,0.00064957270193452,0.00324437969703424,-0.888446630544747

"AHSA1",-0.61309410865,-0.05385906242,-3.62682253826586,0.000658760184610104,0.00328345378346223,-0.901677297318906

"POLR2KP2",0.690779904825,-0.05159484114,3.62618922140072,0.000660045226232053,0.0032886689190278,-0.903512991393948

"SLBP",-0.634483431425,-0.03428295314,-3.62535090012512,0.000661749924499876,0.00329475879785017,-0.905942609682493

"UBAP1L",0.70380693505,-0.09977354996,3.62451980421651,0.000663444088023495,0.00330158913661048,-0.908350962095923

"LRRTM2",0.759255529125,0.02379055205,3.62316509082827,0.00066621452837354,0.00331296195565948,-0.912275960253208

"TMC6",-0.775293037425,-0.05837758794,-3.62299557776403,0.00066656196718291,0.00331388536366015,-0.912767028199625

"RFTN1P1",-1.406732835975,0.23952692222,-3.62265348378875,0.00066726366076095,0.0033157647057168,-0.913758010354702

"NINL",0.8595529929,0.05318075712,3.62220908204957,0.000668176260563691,0.00331793762641589,-0.915045276877118

"CKAP2L",-1.31913289405,0.26750161276,-3.62220396529028,0.000668186775019131,0.00331793762641589,-0.915060097685331

"LINC02764",0.851096162575,-0.06506137794,3.62112109480894,0.000670415536422508,0.0033257805109502,-0.918196379666291

"IQCF5-AS1",0.65467066665,0.01886435412,3.62073883709111,0.000671203995423786,0.00332888585287885,-0.919303368503126

"C6orf163",0.933139550725,-0.17272492542,3.62013306216514,0.000672455306481956,0.00333347793835043,-0.921057505283835

"TCTN2",0.595941718525,-0.12577758818,3.61997596355496,0.000672780179415889,0.00333428164389761,-0.921512386093872

"LRATD1",0.61966421961875,-0.083463251305,3.61917667224933,0.000674435404931406,0.0033408686112753,-0.923826563462392

"EVI2B",-0.69044110365,0.09339631308,-3.61815031422569,0.000676566568861418,0.00334899634279602,-0.926797723481544

"NTN1",1.161534127775,-0.06764006578,3.61697289702113,0.000679019331176199,0.00336032561766618,-0.930205567928903

"IGLV1-36",-1.666952456175,-0.00310255193999999,-3.61636576821727,0.000680287406297509,0.00336578806573856,-0.931962549506981

"DGCR5",0.744436643125,0.0027486895,3.61617762494437,0.000680680829905748,0.00336692149761202,-0.932506985679401

"CCDC85C",0.9422740721,0.01771316268,3.61570858253314,0.000681662585363258,0.00337096380616038,-0.933864196053438

"IL31RA",0.66169430165,0.06017881532,3.61461371980929,0.00068395952257775,0.00338075969243555,-0.937031862539719

"ALOX15",-1.048785043975,0.02719631512,-3.61385064558051,0.000685564769095091,0.00338780776392191,-0.939239261665287

"CTSE",-0.91138605385,-0.04002678808,-3.61365478147486,0.000685977380825163,0.00338902931718012,-0.939805807446584

"ZNF790-AS1",0.63506179645,0.02373275736,3.61322541863194,0.00068688271745733,0.00339186626060877,-0.941047695616517

"ARL6IP1",-0.784960842,-0.0908774176,-3.61312334932247,0.000687098104625516,0.00339211228134158,-0.941342907884366

"APOD",-1.85856561445,-0.17525123756,-3.61183808378686,0.000689815812466231,0.00340388881642778,-0.945059825809051

"NPM1P52",0.636838281775,-0.05443032358,3.61144646558399,0.000690645934885456,0.00340716443870292,-0.946192209461316

"ACP7",-0.60904976205,-0.10861184064,-3.61004492386783,0.000693624651305727,0.00341906382932577,-0.950244244637427

"NPAS3",0.6893529312125,-0.03501992228,3.60892834924692,0.000696006512368555,0.0034286562696338,-0.953471740137665

"GALNT9",0.6322846500375,-0.03818138837,3.60852447044173,0.000696869985669917,0.00343208467942434,-0.954639020055854

"AC111152.2",-0.647038320925,0.02323944146,-3.6084372307366,0.000697056634423823,0.00343217888153696,-0.954891147842437

"MTHFD1L",-0.713811204675,0.00735673126000001,-3.60585494968205,0.000702603107847384,0.00345776274600217,-0.962352460531029

"USH1C",0.83379756315,0.08983014752,3.60570310513671,0.00070293056461986,0.00345776274600217,-0.962791106136645

"LINGO1-AS1",0.94950588685,0.02428452148,3.60557566787901,0.000703205499061972,0.00345776274600217,-0.963159236022879

"OR10H2",-0.717739051025,0.08317960668,-3.60554800291763,0.000703265197315065,0.00345776274600217,-0.963239151190244

"LINC01220",-0.65338365805,0.11702681556,-3.60535785770309,0.00070367564304149,0.00345895071368379,-0.963788409708627

"HEBP2",-0.937919161075,0.17723844594,-3.6021662850635,0.000710599226210055,0.00348644156463165,-0.973005117682786

"RGS1",-1.6411542259,0.00173191827999999,-3.60159072191889,0.000711854726689665,0.00349161590625278,-0.974666730899824

"TCAM1P",0.881166195325,-0.05622789274,3.59870949096856,0.0007181716268746,0.00351670657060392,-0.982982296586798

"ZFP69B",0.595755580925,-0.00533332786,3.59766391073682,0.000720477214332028,0.00352715344497839,-0.985998988572809

"PKP3",0.830335439,0.0957934362,3.59383587640368,0.00072897885784403,0.00356246545758151,-0.997039146132951

"C2orf27B",1.051078160875,0.00577683920000001,3.59379001073952,0.00072908129947478,0.00356246545758151,-0.997171381783039

"PCDHGB8P",1.138955766775,0.15768523142,3.59347017922173,0.000729796030476015,0.00356425770498036,-0.998093462702065

"LINC00638",0.7565061866,0.08899758528,3.59286734955039,0.000731145001791153,0.00356999494678197,-0.999831300979986

"ARFGEF3",0.637662190816667,-0.00464739767999999,3.59269731488525,0.000731525924553654,0.00357049766137067,-1.00032144592644

"ZNF880",0.71949742905,-0.0372722579933333,3.59266581904417,0.000731596504414002,0.00357049766137067,-1.00041223488836

"CLBA1",0.616524062,-0.1068116004,3.59212806965661,0.000732802565949389,0.00357347795439369,-1.00196226227392

"LINC01625",0.8887618179,0.21531328852,3.59208250735851,0.000732904840119205,0.00357347795439369,-1.00209358629377

"SLC2A3",-1.3756574511,0.36063609862,-3.590270331634,0.00073698373083625,0.00358909500294107,-1.0073160119229

"AL021920.2",0.621037312175,0.02941661674,3.59004636285261,0.000737489349289394,0.00359070384701547,-1.00796134871115

"KIAA1549L",0.864039258466667,-0.0297960158933333,3.58955718722466,0.000738594835878435,0.00359358715472882,-1.00937076134794

"ATP10A",0.6347791899875,-0.02445018201,3.58955147847247,0.000738607746415613,0.00359358715472882,-1.00938720873009

"VKORC1L1",-0.6948249769,0.06143613648,-3.58943365095281,0.000738874265529702,0.00359403037800867,-1.00972667598911

"GMCL2",-0.839170711475,0.14551964142,-3.58915103344755,0.000739513904853729,0.00359628789049209,-1.01054088488747

"PGBD1",0.6917568462,-0.14037432404,3.58897094409829,0.000739921771446361,0.00359741747826128,-1.01105969472921

"HSPH1",-0.7486984612375,0.09144087701,-3.5880095169005,0.00074210285317754,0.00360716565976309,-1.01382915790942

"PDE9A",0.605677796475,0.09329286618,3.58782841552507,0.00074251438462596,0.00360830994122786,-1.01435078472276

"FAM138E",0.8591143594,-0.01603401838,3.58600160298848,0.000746677814463297,0.00362682196840304,-1.01961168403371

"GDF3",0.930315222325,0.16283748156,3.58321244538087,0.000753077555498464,0.00365184685374024,-1.02764089807732

"KRT16P6",0.654066062475,-0.03220979522,3.58106798957925,0.00075803363062947,0.00367208240317651,-1.0338116685058

"STXBP5L",0.711606216616667,-0.0521180329066667,3.58070622369143,0.00075887277796436,0.00367473067240222,-1.03485244995827

"ADAMTS16",0.794649017425,-0.12433094026,3.57944385703266,0.000761807904633051,0.00368458931778139,-1.0384837225293

"NOXA1",0.670853342175,-0.05347368926,3.5756743527355,0.000770637061746781,0.00372465489999246,-1.04932238879352

"VPREB1",-2.5698498204375,0.54937660065,-3.57509708216948,0.000771997781136245,0.00372947194999278,-1.05098164649485

"BCCIP",-0.676880502075,0.02576387164,-3.57282964089648,0.00077736475049658,0.00375274481813712,-1.05749744264738

"AFDN-DT",0.749813953575,-0.07665056884,3.57163827140238,0.000780198953133774,0.0037623677526348,-1.06092001466017

"LRRC4",1.293968699875,-0.2169090904,3.57160582148154,0.000780276287629113,0.0037623677526348,-1.06101322742466

"C2orf80",-1.1569038726,0.04868789052,-3.57131541496004,0.000780968709115331,0.00376393481824392,-1.06184740095025

"LBP",-1.4807866371,0.28749563932,-3.57051258475018,0.000782885967006606,0.00377140082107485,-1.06415326706027

"RGS20",0.9262354751,-0.10574008772,3.56850129013731,0.000787708947339939,0.0037919598010105,-1.06992869177117

"C14orf119",0.86040188285,0.000978574279999996,3.56770200185203,0.000789633467956672,0.00379765504495218,-1.07222330553137

"KLRF1",0.8534819450625,0.2187997111,3.56704460449661,0.000791219709532942,0.00380439084931023,-1.07411034572823

"TRGV2",1.1521347035,0.1828323288,3.56628791419799,0.000793049303709469,0.00381050519099428,-1.0762821463044

"FAM47C",1.0778435998,-0.07812696096,3.56579334854043,0.000794247291926082,0.00381422972788716,-1.07770146595956

"PTTG3P",-0.767439362525,0.01309886098,-3.56476370765456,0.000796746941822034,0.00382199750504562,-1.08065598348787

"CCDC73",-0.9924751276,0.27872188792,-3.5646680105038,0.000796979646132013,0.0038222188646893,-1.08093055712142

"HCG22",0.9829054008,-0.05213526566,3.56313982915519,0.000800704491061282,0.00383918408913259,-1.08531460786808

"AC009404.1",0.706769865975,0.05844147778,3.56291824055847,0.000801245977304056,0.00384088151216787,-1.0859502085121

"DNM1P33",0.84122360665,0.14587331632,3.56017774147302,0.000807971785009813,0.00386950115270712,-1.09380905064122

"SUN2",-0.7580937136,0.000126550819999998,-3.55862492678707,0.000811806636964968,0.00388332812938087,-1.09826040145938

"AK5",0.959895877958333,-0.0438315807,3.55739936817656,0.000814845557437839,0.00389695513090253,-1.10177280784364

"ADD2",-1.81498562205,0.24355154006,-3.5567452803636,0.000816471889316741,0.00390382171956158,-1.10364710308158

"SPATA19",0.952778882275,-0.11811909918,3.55515059691259,0.000820449927869772,0.00392192676672792,-1.1082158189845

"GPRASP1",0.778700057775,-0.14510663908,3.55433424516676,0.000822493523057683,0.00393077846988705,-1.11055416470504

"C22orf31",0.716284280075,-0.04696397874,3.55389239038256,0.000823601655991525,0.00393515641976548,-1.11181967262552

"SIK1B",-0.922982036725,0.24176402312,-3.55316531523229,0.000825428199409599,0.00394204497657433,-1.11390186941404

"MTCL1",0.752976752925,-0.11165887606,3.5530190646495,0.00082579607402002,0.00394205278853952,-1.1143206707558

"LINC00261",1.43039422275,-0.1467483228,3.55291509968708,0.000826057679739963,0.00394229437281909,-1.11461837723505

"TRGV10",0.825411667125,-0.0170484193,3.55224152607231,0.000827754502208263,0.00394947234309336,-1.11654704686487

"TNFAIP3",-0.64039673595,-0.08187343706,-3.55110495430337,0.000830625225711403,0.00396224671114674,-1.11980093966469

"LINC01686",0.934248685025,0.00718536702,3.54723705318405,0.000840466040350126,0.00400173558789063,-1.13086967417969

"CNKSR1",-0.816669878725,0.00724849401999999,-3.54483299555543,0.000846638477726621,0.00402644594513997,-1.13774568932033

"PCDHB15",0.65138993245,-0.17732518504,3.5445949070705,0.000847252120862673,0.00402842920683092,-1.1384265104876

"ACVRL1",0.610585897725,0.00968740707999999,3.5441964446151,0.000848280057509424,0.0040323809323093,-1.1395658643068

"GNB2",-0.6359367339,-0.06641311412,-3.54409779640612,0.000848534729751373,0.00403265589031458,-1.13984792469046

"THBS4",1.9067918375,0.24718669,3.54385766414838,0.000849154965151907,0.00403466765446017,-1.14053450430773

"SEMA3E",1.08073855465,-0.11274803228,3.54227753994407,0.000853247056846413,0.00404941526538553,-1.14505165438535

"ABCC8",0.840719903825,0.14920737166,3.5421071982185,0.000853689317577588,0.00405057590053951,-1.14553854317362

"COL17A1",-1.1672581016375,-0.03524010281,-3.54153228432554,0.000855183590653551,0.00405578736234027,-1.14718171899672

"MINAR1",0.69735268415,0.04240987832,3.5397906538489,0.000859725554371105,0.00407458431431622,-1.15215853666806

"KIF2C",-1.26435756805,0.19524917956,-3.53977536125289,0.000859765537391575,0.00407458431431622,-1.15220222968564

"FUT4",-1.064660051275,0.20087929598,-3.53976795666934,0.000859784897568564,0.00407458431431622,-1.15222338554264

"IGHV5-78",-1.81306628725,0.0711347782,-3.539020781207,0.000861740618273687,0.00408027311929542,-1.15435802583747

"H3C2",-1.9076789684,0.31284897528,-3.53819922073294,0.000863895940269473,0.00408953327732,-1.15670486676643

"BMP5",1.010645815625,-0.1706391775,3.53711496664321,0.000866748300254465,0.00410114068720866,-1.15980160648853

"LPAR3",1.1771565992,0.13401759136,3.53539286277274,0.000871297137263698,0.00411993770516438,-1.16471893384482

"AL160191.3",0.6313201045,-0.0244057754,3.5352543184845,0.000871664082185418,0.00412059384305834,-1.16511447302009

"PLA2G5",0.948551586725,0.03984226138,3.53266154009096,0.000878558509214629,0.00415031282840427,-1.17251504101276

"NKPD1",0.7836410422,0.05038185176,3.53226462969504,0.000879618509732613,0.00415436238529317,-1.17364765326324

"IGSF3",1.00676560575,0.1387128356,3.53185967740258,0.000880701244518322,0.00415851742283738,-1.17480313447019

"PRDM16-DT",0.972771749575,0.02024766481,3.53103895729556,0.000882899532775568,0.0041679367805313,-1.17714471231775

"KCNV1",-1.3220837394,0.28928621268,-3.52947325626743,0.000887107768906301,0.00418490996300997,-1.18161086670856

"RPGRIP1",1.19255691205,-0.22233991336,3.52795860830414,0.000891196989365288,0.0042003321642779,-1.18593025361973

"PARP8",-0.64428443295,-0.04355522046,-3.52714083345092,0.000893412271879889,0.00420980465185722,-1.18826187159477

"SLAMF9",1.157673054275,-0.16492408658,3.52619398476184,0.000895983765769021,0.0042190106626237,-1.19096109322186

"BCL9",0.67632419215,0.14254107582,3.52523257296594,0.000898602033180482,0.00423036729223385,-1.19370138207955

"GALNT6",-0.6629816189,0.05656167018,-3.52448115386245,0.000900653494552573,0.00423807735332869,-1.19584281912261

"ARSD",0.6338636595375,-0.01913747257,3.52277811025758,0.000905319547898046,0.00425804526507049,-1.20069522740016

"CADM3-AS1",0.795513737225,-0.01547085922,3.52018533423507,0.000912467610712001,0.00428776064409804,-1.20807998504065

"C1orf54",0.713650536675,0.16023336284,3.51966220662674,0.000913916339342422,0.00429161539318073,-1.20956956041825

"IGHG1",-1.6820807501,-0.13176556108,-3.51875725525042,0.000916427655905043,0.00430045117136567,-1.21214603997216

"TMEM45A",0.9059191025,0.0312836865,3.51444101399592,0.000928496399562032,0.00435210117461151,-1.22442924676648

"UNC13D",-0.912806397,0.0447923214,-3.51421538948066,0.000929131423277433,0.00435308589071759,-1.22507107994036

"MYCBPAP",0.64596845925,0.0290660094,3.51227754744327,0.000934602586857395,0.00437471734053788,-1.23058262153439

"DBIL5P2",0.705274215575,-0.01563386904,3.51152416948648,0.000936737900275197,0.00438371084989453,-1.23272485405941

"SLC15A1",0.6164714761125,-0.17803584716,3.51015655432528,0.000940626047934609,0.0043980410988259,-1.23661295859629

"NLRC3",0.89099382625,0.015564482,3.50671143293298,0.00095048887983117,0.00443893689764783,-1.24640329414774

"KLHL30",1.2544535086,0.34016698038,3.506225824862,0.000951886999577554,0.00444445324688809,-1.24778282357609

"GPR174",0.775936662725,-0.00813236072,3.5054343049112,0.00095417007610065,0.00445308348955584,-1.25003114717563

"N4BP3",0.84589715965,-0.07120245128,3.5049715864644,0.000955507167220076,0.00445729299681351,-1.25134536239577

"ADGRG6",0.883179229325,-0.04194899454,3.50387001869182,0.00095869749489962,0.00447115735841188,-1.25447361571795

"ZNF717",0.732467960425,-0.05798787226,3.50310252952923,0.000960926272211735,0.00447747497499978,-1.2566527923483

"ABI3BP",0.949240940433333,0.02209396108,3.50182131659044,0.000964657902530554,0.00448975718015584,-1.26028996705163

"LINC01431",0.832873150775,-0.05850526938,3.5012896694431,0.00096621041629236,0.00449596161912284,-1.26179899702413

"CTH",-0.78344851275,0.1005094167,-3.50103588266653,0.000966952362742968,0.00449839237236102,-1.26251929748336

"ASXL3",0.9736964443875,-0.21818027399,3.50008493344279,0.000969737297603292,0.00450930044332643,-1.26521800921594

"PTEN",-0.64094285545,0.01175323364,-3.49860173892678,0.000974096221137671,0.00452819390236066,-1.2694262950774

"PTK6",0.7779285625,0.0963104077,3.49847707647451,0.000974463437890869,0.00452819390236066,-1.26977995183009

"OAS1",0.924224519375,-0.00765454950000001,3.49815219011785,0.000975421071878622,0.00453161608177986,-1.27070159062724

"MED30",-0.62993372405,-0.00239773924,-3.4972487836455,0.000978088666027541,0.00454102654747985,-1.27326410300458

"P3H4",0.98777273575,0.2703295906,3.49608895992985,0.000981523605761468,0.0045548030882992,-1.27655335095623

"H3C6",-0.592466191925,0.02346443146,-3.49447194114563,0.000986331765226337,0.00457504306224976,-1.28113808812541

"IQCM",-0.674322803875,0.0811980999,-3.49428124481536,0.000986900272448225,0.00457664390717955,-1.28167868453398

"C4orf47",0.8500583786,-0.08199095612,3.49206090343521,0.000993542614486036,0.0045991190884749,-1.28797170388123

"C3orf80",0.936894224625,0.0400104435,3.48851618272541,0.00100423530571455,0.00463937049578233,-1.29801328911834

"H2BC15",-0.592147464025,0.06155967778,-3.48842806190377,0.00100450251460808,0.00463937049578233,-1.29826284085077

"AKR1B10",1.29325254185,0.34759498648,3.48775583494844,0.00100654313954589,0.00464774754181118,-1.30016641161343

"HCG18",0.7848096133,0.15033256714,3.48609241775802,0.00101160962937768,0.00466903764061496,-1.30487580882321

"FAM9C",-1.071882508575,0.17192095264,-3.48571332067911,0.00101276768968911,0.00467332982575461,-1.30594890026398

"TCEAL7",0.757958819225,-0.04146586262,3.48397366214594,0.00101809818574875,0.00469581171310679,-1.31087234988779

"RAD21-AS1",0.5877865267,-0.05578257364,3.48333485194963,0.00102006226662925,0.00470381177553458,-1.31267988425632

"TDRD6",0.719737333475,-0.16841863597,3.48312089413498,0.00102072090605501,0.00470578981171356,-1.31328523951555

"MXD3",-0.59537612875,0.065895079,-3.4823778314982,0.00102301146862509,0.00471381301701711,-1.31538742504348

"CTSV",0.9426467404,-0.02779592458,3.48203604621839,0.00102406669785839,0.00471684838284404,-1.3163542715456

"AXIN2",0.687879765825,0.00491587666,3.48136949870259,0.00102612758216659,0.00472433750638856,-1.31823964226819

"APOBEC3F",0.636581953025,-0.07164689208,3.47980542105986,0.00103097904087032,0.00474454146211839,-1.3226628636073

"H4C5",-1.344542625,-0.01689606,-3.47961450256587,0.0010315727259019,0.00474620750242041,-1.3232026988498

"TSPOAP1",0.861962277375,-0.1091537851,3.47892038767788,0.00103373390798581,0.0047525182689494,-1.32516520363678

"DUSP7",0.704122547625,-0.0969796189,3.47771705614133,0.00103749079716798,0.00476594427183354,-1.32856687240604

"TMEM229A",1.021363671725,-0.21799831262,3.47738592844207,0.00103852688104142,0.00476893508463479,-1.32950280261334

"TEX51",-0.7601921432,0.13734929144,-3.47661935794082,0.00104092922805843,0.00477793444508822,-1.33166929912952

"RSAD2",1.06016147395,0.12653560766,3.47617627013917,0.00104232022844522,0.00478276748944805,-1.33292142884058

"HSPA6",0.7566673004,-0.05063655108,3.47553339082074,0.00104434158841181,0.0047909696514512,-1.33473797878395

"GARS-DT",0.634011277875,0.02914271755,3.47540483994245,0.00104474622940107,0.00479175302170791,-1.33510119342817

"GHRLOS",0.701655841875,-0.0890196028,3.47450834953172,0.00104757227543983,0.00480219847678334,-1.33763395685379

"RNU105B",0.683624301025,-0.05560648008,3.47445938908425,0.00104772682503034,0.00480219847678334,-1.33777226828537

"HMGB3P24",-1.0325915468,0.30104793956,-3.47432665478765,0.00104814592615273,0.00480304489711859,-1.33814723168442

"ARPP21",-0.775788670975,0.00545596722000001,-3.47418162602114,0.00104860402885836,0.00480406962058367,-1.33855691588582

"BATF2",0.7622465406,0.01593380248,3.47337697459708,0.00105114914815716,0.00481465319638806,-1.34082974293371

"LINC00471",0.60028690965,-0.01928810028,3.47306236253575,0.00105214586728245,0.0048156526024096,-1.34171831159659

"ARSLP1",0.860020362825,-0.12394497854,3.47272903095807,0.00105320287322007,0.00481867361914535,-1.34265969642417

"CCDC112",0.703587447725,-0.07627322122,3.47170311005573,0.00105646245179802,0.00483250786167398,-1.34555672239372

"EXOC3L4",0.73504301585,0.01171581248,3.4695406608082,0.00106336451868287,0.00486082379713847,-1.35166138749446

"CLUH",-0.6192984627,0.01893110384,-3.4689374036805,0.00106529762900478,0.00486531828422871,-1.35336398487602

"COMP",1.8599157982,-0.47033954694,3.46834336448759,0.00106720446969805,0.00487185498555562,-1.355040388145

"LY9",-0.603536881275,0.14742306328,-3.46748350179765,0.0010699703488298,0.00488230568080735,-1.35746664347786

"AC008969.1",0.6869080666,0.02716412728,3.46623259794645,0.00107400625819363,0.00489744941417118,-1.36099562985678

"WDR72",0.877822025275,-0.02183848378,3.46547502590613,0.00107645752547729,0.00490753488695563,-1.36313247250445

"RNPC3",0.664455184675,0.04321437254,3.46498085338131,0.0010780593827903,0.00491374430135876,-1.3645262033437

"GUCY2EP",0.759475761025,0.05821709302,3.46255005998745,0.00108597186902908,0.00494650770401684,-1.37138006671538

"SESN1",-0.981894201875,0.0876715085,-3.46177512180781,0.00108850597158527,0.00495694828758369,-1.37356445883119

"ASB9",0.589371211125,-0.0906178367,3.46157491226245,0.00108916158517716,0.00495883167935769,-1.37412875957475

"CEBPB-AS1",0.7816954641,-0.08964998272,3.4613491455939,0.00108990133990704,0.00496109723439579,-1.37476507023002

"PNMT",-1.201231606425,0.13699168386,-3.45945841923552,0.00109611535638204,0.00498535754732271,-1.38009297156313

"AEBP1",1.035683689525,-0.000795039379999995,3.45943140026324,0.00109620440017029,0.00498535754732271,-1.38016909565034

"AL161729.1",0.73656376115,0.00270291092,3.45874077106108,0.00109848277754385,0.00499461055177948,-1.38211477120315

"KRTAP8-1",-0.67505701955,0.11088168236,-3.45810299090707,0.00110059080826695,0.00500308508537238,-1.38391134488632

"PARP15",0.72289960375,0.0253284,3.45779882532005,0.00110159751055672,0.00500524398539883,-1.38476808206665

"RGS18",-1.066025003725,0.06504088502,-3.45709901456859,0.00110391701499959,0.00501153374768702,-1.3867390485453

"B3GNT9",0.81800579245,0.05282286696,3.45635080895295,0.00110640206325468,0.00502170258572485,-1.38884604328983

"PTP4A3",-0.598640019575,0.10367222184,-3.45615982472892,0.00110703723984377,0.00502347264007069,-1.38938382194359

"NPTX2",0.660302948525,0.04809270912,3.4558672107884,0.00110801109032444,0.00502677839206533,-1.39020773677094

"H4C3",-1.1040799918125,0.20353872205,-3.45555934614171,0.00110903657676375,0.005030316888363,-1.3910745466038

"KCNK17",0.72856478905,0.05481652569,3.45518729843423,0.00111027705990375,0.00503482877452592,-1.39212200373666

"TRAM2",0.652564698,-0.0170136996,3.45487227651874,0.00111132843948473,0.00503848131625651,-1.39300885691582

"SPACA6",0.7319656766,0.07861663928,3.45355235719011,0.00111574395357681,0.00505290939895703,-1.39672416531398

"ARF1",-0.7621839072,-0.16223611926,-3.45305418209879,0.00111741482908675,0.0050593580151004,-1.39812620035728

"GDPD3",0.8404136412,-0.02697500304,3.45246336890786,0.00111939949804145,0.00506645751205271,-1.39978878867148

"TTC41P",0.593421917633333,-0.0886296508266667,3.45244019602266,0.00111947740907178,0.00506645751205271,-1.39985399515577

"SCAMP5",0.605850773375,0.0349998558,3.45159253886922,0.00112233091975308,0.00507712870035353,-1.40223904274296

"MIR3659HG",0.650109938025,-0.02465502238,3.44882932240467,0.00113168099622835,0.00511490850446206,-1.41001137285527

"AC004151.1",-0.66700109455,0.04587730396,-3.44682601173037,0.00113850601550931,0.00514074750236057,-1.41564384400684

"CNTNAP4",1.02394544175,-0.0581669906,3.44561796276282,0.00114264058671264,0.00515534471274358,-1.41903939283581

"MYH10",0.5965616678,-0.02060937876,3.44485433476314,0.00114526148163713,0.00516603197617384,-1.42118539591088

"LINC02860",0.844132920075,0.02357427606,3.44267820049777,0.00115276172822028,0.00519271657341392,-1.42729931051298

"CKS2",-0.9179184577,0.04837904284,-3.44219324566519,0.0011544395234388,0.00519827625411299,-1.42866147943451

"GADL1",0.887195839325,0.19402719846,3.44081324361456,0.00115922662502505,0.00521639250900794,-1.43253705727718

"CTNND2",0.6149776873,-0.10255105916,3.4380064320061,0.0011690215090441,0.00525585092764879,-1.44041669212475

"IGHV2-26",-0.8666132453,-0.03002283924,-3.43781026170947,0.00116970901571392,0.00525778813381894,-1.44096725688462

"INO80",0.780595742125,0.0498315497,3.43617914118344,0.00117544041735459,0.00527776096225308,-1.44554434796618

"GRB2",-0.586012767975,-0.18835987088,-3.43468504261501,0.00118071377821109,0.00530027692550151,-1.44973575763157

"ANGPT2",0.975206056425,-0.10760293886,3.4338005555682,0.00118384613145526,0.00531084728295498,-1.45221648434653

"SHISAL2B",1.93989960285,0.92802698568,3.43329523794511,0.00118563922566551,0.00531656302373277,-1.45363357384252

"SYT13",1.430903232825,0.21997993276,3.43316843067104,0.00118608960004724,0.00531741875837372,-1.4539891659839

"BCAR3-AS1",0.61512975765,0.02294792012,3.43165329458252,0.00119148342286341,0.00533692880796363,-1.4582372692587

"MAP2K1",-0.761290183425,-0.03545893974,-3.43027576619877,0.00119640760025005,0.00535313363483798,-1.46209853921662

"SPNS3",-0.955701862325,0.12626555614,-3.42876493499808,0.00120183053217073,0.00537504997666752,-1.466332353601

"BOLA3P3",-0.658281587675,0.00808322926,-3.42823949605537,0.00120372199726701,0.00538233441292156,-1.4678045233372

"GLDC",1.049641650275,0.32410209722,3.42701212474238,0.00120815129654187,0.00539742777225246,-1.47124281317877

"C6orf132",0.7376107092625,0.06597265941,3.42690999920449,0.00120852054135323,0.00539790033818291,-1.47152886744605

"PSAT1",-0.73306619055,0.15635891856,-3.42582793467365,0.00121243944622066,0.00541319545980304,-1.47455941082442

"BIN2",-1.020126481475,-0.09518783818,-3.42201431901241,0.00122634771394843,0.00546398771771473,-1.48523546798353

"DMTN",-1.363992450825,0.22528293504,-3.4216580830308,0.00122765462323386,0.00546667853093139,-1.48623235711782

"MAFF",-1.1308145078,0.32682679246,-3.4215109692552,0.00122819471999612,0.00546789513760985,-1.48664402074948

"OR2L13",-0.87802625235,0.14142065232,-3.41857724554849,0.00123901252870971,0.00550767817058028,-1.49485105739459

"CHADL",0.735961277825,0.01821418466,3.4177730900652,0.00124199354494025,0.00551614215233813,-1.49709989995715

"BMP4",0.83928945495,-0.17559111334,3.41739861919151,0.00124338403866553,0.00552112097619838,-1.49814700517088

"LINC02608",0.912627240325,0.05941712166,3.41644863574169,0.00124691817828009,0.00553321629556043,-1.50080305223332

"LIM2",0.79946848915,-0.23371688968,3.41554425652417,0.00125029152773075,0.00554506071244694,-1.50333116645198

"PRMT5",-0.64907243305,0.02818720756,-3.41551540631264,0.00125039928209219,0.00554506071244694,-1.50341180785229

"BZW2",-0.71821165565,0.000784386730000008,-3.41515451576796,0.0012517479393204,0.00554863950255222,-1.50442052425326

"RFPL2",-1.122556283275,0.01804565338,-3.41165210126635,0.00126490854454574,0.00559487181793032,-1.51420657725833

"GNAT2",0.6331694598,-0.10126558116,3.41130768273353,0.00126620980061883,0.00559744567807304,-1.51516857375849

"NELL1",1.0195459675,-0.051411203,3.40697962748721,0.00128267051288685,0.00566365376466074,-1.52725209786189

"ALDH1L2",0.956862943025,0.20745627542,3.40613753722898,0.00128589673340156,0.00567538886949772,-1.52960201866286

"ANGPTL3",-0.657477926725,0.09069790762,-3.40606910736952,0.00128615924058366,0.00567538886949772,-1.52979296172139

"AL450332.1",1.702982413475,0.23011773278,3.40586515977572,0.00128694191603861,0.00567720385193259,-1.53036203204061

"METTL27",0.875548056925,-0.08052959246,3.40581753415847,0.0012871247506944,0.00567720385193259,-1.53049491763977

"KIAA1324L",0.7400890974,-0.10134818008,3.40538537428068,0.0012887849397371,0.00568330302310237,-1.53170068238326

"MCM4",-0.942474273325,0.22782227334,-3.40475007228703,0.00129122921449186,0.00569285649806524,-1.53347305775729

"ARHGEF19",-0.92901948015,0.19836672118,-3.40411455837041,0.00129367870949729,0.00570120228009905,-1.53524581702654

"RPL15P20",-0.625423640675,-0.04348867554,-3.40333630226671,0.0012966843952208,0.00571076308884001,-1.53741647156227

"ATP8A2",0.68174364465,0.124664118853333,3.40321698310205,0.00129714580111135,0.00571156741310325,-1.53774924027077

"LINC02540",1.096409259025,-0.16071115368,3.40253313284769,0.00129979325184381,0.00572162452146491,-1.53965628622535

"LGALS2",1.323101098225,-0.11891738142,3.40242732657928,0.00130020332727206,0.00572162452146491,-1.53995132557825

"UROD",-0.6625712848,0.13201200416,-3.40241073112449,0.00130026765774798,0.00572162452146491,-1.53999760125823

"LOX",0.95171206575,0.0272945779,3.40194614319913,0.00130206980966526,0.00572709451705064,-1.54129302636583

"DEPDC1B",-0.82122556955,-0.29299554644,-3.40184812971533,0.00130245031010605,0.00572753851759022,-1.54156630627074

"DACT2",0.8226266599125,-0.14773761452,3.40016075013627,0.0013090175071565,0.00574793846858524,-1.5462702613449

"ADIRF-AS1",0.8584306415,0.0535298524,3.40004303094354,0.00130947683589091,0.00574856683388321,-1.5465983757798

"NCKAP1L",-0.69319643925,0.0098956026,-3.399337591392,0.00131223260028753,0.00575943048729452,-1.5485644724848

"TENT5C",-1.4450357547,0.17539297624,-3.39877871778463,0.00131441972106813,0.00576779422686739,-1.5501219005034

"UCHL1",0.868472008825,0.11380226006,3.39849793683896,0.001315519847701,0.00577014999779451,-1.55090429960455

"ZNF702P",1.22100924495,0.11497515726,3.39736611518536,0.00131996330556161,0.00578751010282325,-1.55405772121186

"MOXD1",0.79525627115,-0.02566025508,3.39734592661458,0.00132004269379603,0.00578751010282325,-1.55411396355242

"LINC00242",1.1232705136,0.00663595238,3.39684136170219,0.00132202828562713,0.00579497550618925,-1.55551953780391

"HASPIN",-0.761784078175,0.01043375846,-3.39638658231268,0.00132382038848646,0.00580034904176873,-1.55678631140335

"TMEM198B",0.62878007735,0.13376747088,3.39598266245267,0.0013254140083414,0.00580608984453681,-1.55791132805659

"LINC00305",-0.662303164275,0.12890409968,-3.39488857482913,0.00132973976090437,0.0058237939763593,-1.56095821028205

"ZNF780B",0.7507836288,0.16960363619,3.39290824176056,0.00133760362010032,0.00585323002319851,-1.56647159381623

"PSMA8",-0.95212675735,-0.13322692788,-3.39166334985458,0.00134256963901066,0.00586994595903764,-1.56993642307124

"DBNL",-0.602299361775,-0.07293913042,-3.39065264944614,0.00134661430965772,0.0058826085237244,-1.57274885361234

"TEF",1.402235194775,0.27184156582,3.38997339211751,0.00134933909172968,0.0058882341451241,-1.57463869608681

"RAMP1",0.723696658,-0.0242033871,3.38975822546886,0.00135020330514797,0.00589075070610554,-1.57523728707883

"IFI6",0.87421722615,0.23762003192,3.38848648116869,0.00135532198624695,0.00590930769402587,-1.57877477581049

"AL772363.1",0.778011560125,0.0263160804,3.38839311221662,0.00135569851464403,0.00590969173787805,-1.57903445831204

"COL5A3",0.67196455605,-0.06995552346,3.38610941867314,0.00136493892634389,0.00594364903781799,-1.58538458048361

"NDST4",1.302058374375,-0.3070895495,3.38446573631181,0.00137162665267981,0.0059689649235565,-1.58995339473304

"RAB5C",-0.67769087225,0.1527713672,-3.38424776244812,0.0013725158651192,0.00597156615938885,-1.59055917450145

"CTHRC1",1.6148324243,0.22726068944,3.38047034270748,0.00138801280896376,0.00603258515698973,-1.60105324302251

"GUCY1A2",1.04381310625,0.3035836232,3.37786441283389,0.0013988003239134,0.00606917006275683,-1.60828847888358

"CYP4Z1",1.082802714425,0.31048026704,3.37669859391879,0.00140365206482074,0.00608506633602355,-1.61152417698772

"LINC02694",-0.816730122475,-0.23651650598,-3.3750766181316,0.00141042874231022,0.00611056546256117,-1.61602475205762

"IGDCC4",0.856594345975,0.00975019578,3.3731156796201,0.00141866307139225,0.00613659512566562,-1.62146403849813

"KLF17",0.6307784858,0.03728737304,3.37235324097123,0.00142187698407974,0.00614846256855756,-1.62357836485142

"DENND2B",1.029616961325,0.05034433506,3.37119471526751,0.00142677375031651,0.00616573309354244,-1.62679050554036

"ZNF185",-0.77564669625,0.020449963,-3.37110543060587,0.00142715179500447,0.00616606621173588,-1.62703802816518

"ANKRD36C",0.6032306628,-0.06551955876,3.37084190525429,0.00142826815519807,0.00616828793501223,-1.62776857164232

"RNF165",0.86182239,0.047026652,3.37039848830132,0.00143014845169689,0.00617250505126731,-1.62899772803327

"PLAUR",-0.9273736412,-0.09327468286,-3.37031512391526,0.00143050221805204,0.00617273155927763,-1.62922880354892

"CLEC2L",-0.999236874049999,-0.28264155044,-3.36885246403985,0.00143672270513224,0.00619305171107706,-1.63328252284506

"MST1",0.61044412275,0.0073573474,3.36800217112763,0.00144035066622814,0.00620607874861875,-1.63563857288233

"FIGLA",0.733232581375,-0.0057573129,3.36720999455709,0.00144373847898309,0.00621675366906378,-1.63783325127596

"TFPI",-0.736151588316667,0.0740050262466667,-3.36621292950671,0.00144801325915099,0.00623385073618104,-1.64059509628326

"TMSB15B",0.724545452575,0.03655230226,3.36510494845077,0.00145277765839796,0.00625173464928744,-1.6436635679034

"APEH",-0.58835908725,0.0064810932,-3.36275682603782,0.00146292394439056,0.00629143278183747,-1.6501644028519

"EYA1",0.6016979948,-0.04404770906,3.36209011950849,0.00146581702418568,0.00630255177156688,-1.65200967187101

"NEUROG1",0.74825501165,0.06397194032,3.36102860552149,0.00147043451507826,0.00631842750522721,-1.65494718555215

"VN1R68P",0.59913323565,-0.10702601278,3.36045402589543,0.00147293963545986,0.00632750134605928,-1.65653696668294

"NOVA1",0.8177669378,-0.12835883426,3.35877145810462,0.00148029879266629,0.0063541281400983,-1.66119140321065

"CYP4A22",0.88613520205,-0.06717970136,3.35870192620672,0.00148060365779136,0.0063541281400983,-1.66138371536329

"MMP25",-0.8658503838625,0.22595803611,-3.35851913689456,0.00148140538815139,0.00635490653108158,-1.66188926416646

"KCNA1",1.278088972375,0.0267264659,3.35841359713029,0.00148186848248398,0.00635556237578644,-1.66218115238875

"KIF11",-0.863544743525,0.16020790018,-3.35728720118408,0.00148681951468069,0.00637412810562419,-1.66529602969779

"TBPL2",-0.651384306,0.1011236572,-3.35695548306106,0.00148828055658038,0.00637772266120451,-1.66621321940618

"MFSD13B",0.823572997275,-0.05870870598,3.35546583368649,0.00149485846846384,0.0064032323441119,-1.6703313411222

"SNHG16",-0.58965986415,-0.04835304212,-3.35525432598681,0.0014957946642385,0.00640456448922013,-1.67091595801241

"LOXL4",0.9006521856,0.24117041648,3.35470104348133,0.00149824628631519,0.00641015092671472,-1.67244514522371

"OR56A3",0.922521101825,-0.14587417454,3.35456746165533,0.00149883876463378,0.00641015092671472,-1.67281432069454

"RCN3",0.77731272645,0.03059976916,3.35429170598233,0.00150006253227633,0.00641324107199492,-1.67357638755201

"AC022148.1",0.8513949402,0.04246219656,3.35307710787488,0.00150546404384043,0.00643118167852432,-1.67693252606621

"CCNB1",-0.636804024175,0.08350876091,-3.3529319149506,0.00150611097239995,0.00643260404472175,-1.67733366677846

"AKAP6",1.1404875827,-0.18532322184,3.35261554715292,0.00150752150881195,0.00643594512868081,-1.67820769317764

"TCEANC",0.6005618017,-0.09832865764,3.35227153155975,0.00150905673508962,0.00643981553603753,-1.67915804235756

"PPP1R32",0.610198677025,0.04938607362,3.35201208474089,0.00151021553953466,0.00644341857017622,-1.67987472776759

"KIF5C",0.59952567645,0.06711749116,3.35142133301408,0.00151285724637942,0.00645334567225101,-1.68150646482618

"DYDC1",-0.65965309745,0.08653370804,-3.35040083169448,0.0015174310176536,0.00646849017556786,-1.68432479795198

"NEURL3",1.2132533505,-0.1810614306,3.3503474887952,0.00151767045460536,0.00646849017556786,-1.684472100781

"KCNAB1",0.635927396725,-0.01007139992,3.34904572652284,0.00152352470697592,0.0064893917248488,-1.68806636703798

"LRRC36",0.7130224445,0.0434323596,3.34807886732154,0.00152788668800981,0.00650661867884949,-1.69073536450442

"MEX3B",0.794624984975,0.13253159338,3.34771893988623,0.00152951352051517,0.00651084001834921,-1.69172881238385

"MMP9",1.38828453425,-0.2573777316,3.34751790166663,0.0015304229056896,0.00651335780884266,-1.69228367516218

"VPS9D1-AS1",-0.66992003245,0.02137374514,-3.3463292854671,0.00153581002543423,0.00653221419993698,-1.69556380613344

"H2AC4",-0.76059752915,0.09249882768,-3.34506589266135,0.00154155575400084,0.00655257142903925,-1.69904947851351

"ACAN",0.9368225479375,-0.04162388775,3.34420102396897,0.00154550079234796,0.0065652540397315,-1.70143514810724

"FOXG1",1.091670060375,-0.1641152643,3.34402804545609,0.00154629096912925,0.00656664445045063,-1.70191224800446

"AL590787.1",0.802595325775,-0.07744912238,3.34398905628966,0.00154646912697307,0.00656664445045063,-1.70201978362346

"C2orf78",-0.639732325,0.115017192,-3.34157812921632,0.00155752353343652,0.00660732311935639,-1.70866778340438

"SELP",0.8804510150375,-0.11139776467,3.34154812209637,0.00155766159051598,0.00660732311935639,-1.70875050713017

"BLNK",1.1452330233875,-0.27793511029,3.34077787042044,0.00156120934435043,0.00661963101139313,-1.71087377722473

"PCED1B-AS1",0.680104587,-0.0274756654,3.34033740274034,0.00156324156377857,0.00662413508969915,-1.71208782681055

"TTLL13P",0.5973603191,0.03808431828,3.33985787249036,0.00156545685899294,0.00662940887289113,-1.71340942738605

"LMNTD1",-0.641417040275,0.11050247278,-3.3391209110923,0.00156886721342266,0.0066414331938697,-1.7154402802798

"FBN2",-0.8471471519,-0.09934081102,-3.33910420906844,0.00156894458504146,0.0066414331938697,-1.71548630292121

"IGHV3-64",-1.23878274755,0.27876387896,-3.33823143420741,0.00157299272258661,0.00665444453858239,-1.71789104075529

"GLDN",-1.093770232625,-0.0143488705,-3.33641433099631,0.00158145265225701,0.00668747202052459,-1.72289637812195

"CSN1S2AP",-0.632374778375,0.0848863499,-3.3358816206111,0.0015839409641544,0.00669108916981347,-1.72436343596405

"RASGRF2",0.606519129475,0.01580407308,3.33538702004426,0.00158625458387067,0.0066981005594275,-1.72572540725789

"ASB5",0.865476576825,-0.17489345654,3.33452994712337,0.00159027134275657,0.00671091231110126,-1.72808520552166

"OR4X1",-1.050121883275,0.12436010578,-3.33376498946399,0.00159386453049256,0.00672330584738141,-1.73019105333833

"SORD",0.6829387669,-0.02591703148,3.33363320919122,0.00159448430980408,0.00672453572236847,-1.73055379935088

"SYNGR1",-0.58560234595,0.095621377815,-3.33299653438105,0.00159748188947547,0.0067338423433457,-1.73230621815667

"RPS2",-0.6556273028,0.10505928836,-3.33295511987046,0.00159767706125554,0.0067338423433457,-1.73242020232182

"OR11L1",-0.644284908325,0.09850158774,-3.33230470999903,0.00160074517496371,0.00674399900157445,-1.73421019100662

"AC009078.3",0.622215356,0.0277268588,3.331819668433,0.0016030368454045,0.00675087746940335,-1.73554492522574

"RPH3A",-0.68730330095,-0.04395513516,-3.33174288313421,0.00160339991685363,0.00675095442179815,-1.7357562111152

"AL355390.1",-0.6402073419,0.10367817958,-3.33166784731465,0.00160375479132035,0.00675095442179815,-1.7359626800494

"SNX20",-0.603511225725,0.05326203242,-3.33151159716493,0.00160449399968818,0.00675146294220815,-1.73639260919143

"KCNK2",1.545475323975,0.43005373168,3.33116693355829,0.00160612572006277,0.00675555404488728,-1.73734092061401

"GSN-AS1",0.748030171375,-0.0914185749,3.33013102517051,0.00161103942628002,0.0067744574901006,-1.74019075298864

"ATP8B4",-0.84325676775,-0.1481429472,-3.32994094075691,0.00161194261344399,0.0067744574901006,-1.74071362249849

"AC007368.1",-0.632177364875,0.0870294947,-3.3280386713692,0.00162100771177231,0.00680557499975226,-1.74594518567145

"STX3",-0.668669841625,-0.0478745672,-3.32673852727536,0.00162723118956525,0.00682618397623702,-1.74951970088472

"NUP62CL",-0.87208731895,0.01012752484,-3.32673476209266,0.0016272492454091,0.00682618397623702,-1.74953005128646

"FRS3",0.817242990875,-0.0853361993,3.32652927344834,0.00162823494911107,0.00682892070366174,-1.75009492352234

"ASPA",0.913379563925,0.20791374034,3.32637125800166,0.00162899331431731,0.00683070303392,-1.75052928044132

"FAM177A1P1",1.480124036875,0.1857847345,3.3260271400654,0.0016306460008115,0.00683623393996851,-1.75147515492254

"AP003057.1",-0.615777007225,-0.00489262478,-3.32545321018269,0.0016334059325778,0.00684640358974097,-1.75305257233892

"AC106822.1",0.799135256075,0.00429200185999999,3.3252886694644,0.00163419799664841,0.00684832246989825,-1.75350477205279

"TMEM238",-0.727043043,0.1052056316,-3.32469174960889,0.00163707449643451,0.00685897388199393,-1.75514513915243

"PTGDS",0.63704471815,0.00113872392,3.32385756110472,0.00164110240452833,0.00687163418434016,-1.7574372167209

"UTP18",-0.5909163098,-0.00448592083999999,-3.32323923582777,0.00164409406389303,0.00688275422579492,-1.75913593467006

"IL2RG",-0.6461922327,0.02878938284,-3.32051098759305,0.00165735598973609,0.00692977745259449,-1.76662879929414

"ZNF829",0.619538659675,-0.05478920826,3.32026490494684,0.00165855715675983,0.00693197042588645,-1.76730444687585

"HEY1",0.991409799925,0.29350320394,3.31810628444552,0.0016691290980391,0.00697046819417551,-1.77322980457055

"H2AC21",-0.847859408425,-0.06725418074,-3.31773765876654,0.0016709408246809,0.00697604840475948,-1.77424142522751

"PLTP",-0.816520438025,0.23020412748,-3.31768789875114,0.00167118552847431,0.00697604840475948,-1.77437797627136

"GLRB",-0.638917430125,0.0613693325,-3.31750556516254,0.00167208247653301,0.00697711319332143,-1.77487832343463

"AACS",-0.64183047615,0.08007505023,-3.31653405006225,0.00167686930991141,0.00699281465936598,-1.77754398987493

"LCAL1",0.7451553236,-0.03906055772,3.31506446387731,0.00168413489280919,0.00701740716326237,-1.78157532239863

"C9orf135",-0.639972105925,0.11616600996,-3.31506411571883,0.00168413661761817,0.00701740716326237,-1.78157627732232

"FANCA",-0.659984957025,0.04153389748,-3.31145521565728,0.00170210556604414,0.00708168586532977,-1.79147124801319

"LHX2",1.32684198375,0.356838013,3.31143710565554,0.00170219619284288,0.00708168586532977,-1.79152088498009

"DNAH10OS",0.672593911975,-0.05637299502,3.31141070165015,0.00170232833301196,0.00708168586532977,-1.7915932543312

"DNASE2B",1.47685926025,0.2068969647,3.30782633765743,0.00172035686078795,0.00714952475618048,-1.80141400188447

"LINC00475",0.827840277825,0.07513595626,3.30669975266227,0.00172606054620927,0.00716731424214341,-1.80449930143357

"FER1L6-AS1",0.93861616215,-0.21645555508,3.30658417688106,0.00172664669544181,0.00716829533864473,-1.80481578245649

"ANKRD30BP2",0.755381162375,-0.0682512861,3.30613608623209,0.00172892099432434,0.00717628308989408,-1.80604272116836

"RAB6C-AS1",-0.6424456203,0.09848795236,-3.30332598921268,0.00174324849635425,0.00722550580483631,-1.813734740567

"SPATA4",0.7462910259,0.07829808572,3.30219610238947,0.0017490409213506,0.00724365279899558,-1.81682636729394

"KCNN2",0.5854064607,0.02016928596,3.30131282662769,0.00175358175926526,0.00726099092235784,-1.81924273510549

"LINC00634",0.6970361469,-0.00704116908,3.30079386283281,0.00175625490309023,0.00727042373180267,-1.82066226417287

"DEFA9P",-0.916826776,-0.0210256588,-3.30033945391821,0.00175859869494365,0.00727588248424992,-1.82190509711331

"STAT5A",-0.665667224275,-0.03651242362,-3.29731884655448,0.00177425403815103,0.00733769009560723,-1.83016381400123

"PHF24",0.713917982225,-0.04579362022,3.29677262431313,0.00177709906337271,0.00734478356562973,-1.83165673229868

"MYOM1",1.339859724275,0.15486220942,3.29671923202205,0.00177737739114658,0.00734478356562973,-1.83180265394094

"AC020911.2",0.73191187905,-0.08695150416,3.29671427997995,0.00177740320765288,0.00734478356562973,-1.83181618784201

"LY6E-DT",0.626854825475,-0.05372100662,3.29589301292158,0.00178168963450772,0.00736101174264855,-1.83406052403919

"FOXD3",0.766685634775,0.02044175492,3.29557199852107,0.00178336775675786,0.00736497452135759,-1.83493768522383

"TGM4",-0.686622384325,0.12441539734,-3.29514935101575,0.00178557945239061,0.00737262229354671,-1.83609247129734

"PAGE4",0.76387692085,-0.03248524042,3.2940018680292,0.00179159727171814,0.007394489303578,-1.83922721911313

"MIR3142HG",0.619996632825,-0.16198113574,3.29380879809047,0.00179261168443305,0.00739476345134628,-1.83975458725998

"AL356414.1",0.5903390868,0.00169517403999999,3.29349690494747,0.00179425155634984,0.00739948186121085,-1.84060647730115

"FGD5P1",0.667189897675,-0.01275072086,3.29250000776857,0.00179950256356392,0.00741964349412306,-1.8433290048619

"PRKAR2B",-1.453592189375,-0.2810392915,-3.29168616261302,0.00180380014463588,0.0074343708234359,-1.84555122189504

"AL161756.1",-0.640272927675,0.09894078796,-3.29052518848158,0.00180994757433701,0.00745221179723004,-1.84872066498821

"ZIM3",-0.6399195868,0.09420823226,-3.29034044510781,0.0018109276273842,0.00745474889086751,-1.84922494509167

"CDC25A",-0.75757444605,-0.10310382884,-3.28972057178619,0.00181421968836379,0.00746680051731566,-1.85091683283613

"WNT10B",0.792157211825,0.10491950756,3.28924791038727,0.00181673372960543,0.00747564584297169,-1.85220678041894

"AC100803.2",0.64147047365,0.04123405492,3.28831780330046,0.00182169050467675,0.00749303253559871,-1.85474479979135

"INSL6",-0.70114659905,0.17524643176,-3.28799817129563,0.00182339685488816,0.00749704089162125,-1.85561688478085

"LEKR1",0.720021866675,-0.03848860846,3.2868632420488,0.00182946787529444,0.00751446227526716,-1.85871298585687

"IFNG",0.9033489429,-0.000152378180000003,3.28621262442782,0.00183295680606698,0.00752728383297261,-1.86048756660302

"SILC1",0.67988726915,-0.07900452863,3.28613032996708,0.00183339855729165,0.0075275891065714,-1.8607120112113

"LINC01141",-1.287208032725,-0.01269841058,-3.28579168524848,0.00183521743945676,0.00753203824110379,-1.86163557066744

"EZH2",-0.942869535325,0.15260357574,-3.28508156773667,0.00183903706722529,0.00754426775957247,-1.86357201879074

"CD48",-0.867998011725,-0.06033321438,-3.28494633917952,0.00183976529517224,0.00754465799989769,-1.86394074826292

"MFSD12",-0.60154953005,0.02368934696,-3.28400235673779,0.00184485639543674,0.00756402197912906,-1.86651444348104

"ADAM12",0.729989185308333,-0.0142226163533333,3.28307977930035,0.00184984492268223,0.007582957738278,-1.86902931628697

"LINC00243",0.796884258475,0.06317922678,3.28104142812295,0.00186091187463065,0.00762222360804335,-1.87458407502229

"LYVE1",-1.45701067375,-0.020007551,-3.28085671926938,0.00186191781347374,0.00762481954076552,-1.87508731888636

"NMU",-1.022288359525,0.06550924538,-3.27973965642038,0.00186801238894081,0.00764519333292435,-1.87813039263009

"DNM1P47",0.7698151816,-0.11375512857,3.27817571961994,0.00187657675185041,0.00767596902112595,-1.88238969882783

"FIGN",0.694421386758333,-0.0307278011266667,3.27662800369916,0.0018850888185696,0.0077043853272818,-1.8866035296197

"ARGLU1",0.6205356187875,0.08028879653,3.27629036914064,0.00188695057067999,0.00770884213202225,-1.88752260598277

"PCDH9",0.739158202275,0.01652840522,3.27572210964513,0.00189008793738841,0.00772011994840786,-1.88906932848074

"MCOLN2",0.724528231975,0.04880188058,3.27354485644489,0.00190215426922387,0.00776476124085646,-1.89499389203461

"CACTIN-AS1",0.92067180675,0.0090138324,3.27313183539532,0.00190445143086757,0.00777258982382763,-1.89611748224922

"FCGR2B",0.6669800955,0.0551167614,3.27220058680988,0.00190964052095125,0.0077906640767123,-1.89865053014552

"OR52E2",-1.261730158475,0.28286908172,-3.27164008117269,0.00191277020098164,0.00779892627196408,-1.90017491065536

"HSP90AB1",-0.63160305775,0.0285814868,-3.27144416135918,0.0019138652959194,0.00780168567106977,-1.90070770438985

"KCNH2",-1.4081409580125,0.18671658859,-3.27117875279364,0.00191534974365398,0.00780618371752191,-1.90142943606722

"GPBAR1",-0.682028035075,0.07145586714,-3.27099966660553,0.00191635199932039,0.00780871514758873,-1.90191640777657

"CPVL",-0.636321295675,0.01286259886,-3.27040019865475,0.00191971052846536,0.00781929012071538,-1.90354635665662

"AL354984.1",-0.71159410045,-0.06209276976,-3.2696937631364,0.00192367548473447,0.00783076958697392,-1.9054669000513

"MYH1",1.7123917106,0.16637460648,3.26820452403681,0.00193205938862693,0.00785865252217362,-1.90951471939924

"SPRY1",-1.0047189158375,0.23027714248,-3.26780525037877,0.00193431302437901,0.0078646964166456,-1.91059975922288

"RBMXL3",-0.640135122925,0.08612875026,-3.26721412781011,0.00193765408166043,0.00787359318228063,-1.91220599653069

"SOWAHD",-0.7452749761,0.00599597912,-3.26683341016828,0.00193980880479581,0.00788078580064577,-1.91324040748312

"SLC35B1",-0.596324259225,0.01140789802,-3.26587606390242,0.00194523704437173,0.00790127211837591,-1.91584117316578

"CAPN11",0.97478744915,0.00821246551999999,3.26560914696847,0.00194675304407243,0.00790586251079404,-1.9165662020106

"FKBP14",0.6387778333,0.06616237474,3.2595911865377,0.00198123081094202,0.00802996179121269,-1.93290258651457

"AP006587.6",0.8044646375,-0.039111901,3.25631367682054,0.00200024993732034,0.008100636630919,-1.94179145979992

"B3GNT3",0.7546812723,0.01866135884,3.25563359060271,0.0020042179666196,0.00811349885400164,-1.94363517744238

"IGHV1-45",-1.16730913545,0.08528983824,-3.25518641060155,0.0020068311318959,0.00812223607518713,-1.9448473479026

"NRAD1",0.68572057155,0.00474582323999999,3.25512860963005,0.00200716913552677,0.00812223607518713,-1.94500402100918

"MAN1A1",-0.71051415915,0.06301528988,-3.25488961486812,0.00200856727970038,0.00812628878880358,-1.945651811791

"SAMD9",0.730980759025,-0.08859332888,3.25461623073666,0.00201016773305645,0.00813115824577702,-1.94639277620173

"HOXC13",0.717927811625,-0.0842722917,3.25447663485359,0.00201098542376537,0.00813175914826015,-1.94677111305966

"ZNF41",0.655641063575,0.14043888086,3.25328814032511,0.00201795982987787,0.00815301932449965,-1.94999177627836

"BCAM",0.599681699825,0.13832227396,3.25243251104974,0.00202299502806187,0.00817175120956855,-1.95230994254742

"CDH15",0.8087877768,0.15276361944,3.25229025180799,0.0020238333431621,0.00817260072049625,-1.95269532850158

"HBA2",-1.973953567625,-0.1066844216,-3.25149422163557,0.00202853030156789,0.00818765343154512,-1.95485160255004

"FABP5P3",-0.706172001125,-0.0210321661,-3.25113831841467,0.00203063362884117,0.00819291488532844,-1.95581555594626

"HMBOX1",0.59719425865625,0.2179883662,3.25094255759689,0.0020317914195551,0.0081941217210316,-1.95634573893819

"TMEM117",0.6430550948,0.18634387144,3.25087918979715,0.00203216633006881,0.0081941217210316,-1.9565173547614

"PNPLA1",-1.07239503545,-0.09309477336,-3.25007137418653,0.00203695143426525,0.00820870593729865,-1.95870492936966

"SLC40A1",0.82420031955,-0.19146180936,3.24931026118434,0.00204146962749772,0.00822529591507834,-1.96076570729701

"MMP14",0.779882763575,0.18091991576,3.24866941549513,0.00204528121438755,0.00823741331243799,-1.96250060636235

"AC018450.2",-0.63814361815,0.00628303448,-3.2452878962959,0.00206550514569225,0.0083106820981171,-1.97165135346238

"COL9A2",0.6074908025875,0.03835587007,3.24436722292721,0.00207104404938809,0.00832338879432999,-1.97414171145699

"PRRX1",1.048896650875,0.1341699357,3.24351954806999,0.00207615617886092,0.00833881352528966,-1.97643420410704

"CCL4L2",1.934932574575,0.81136339621,3.24252772642396,0.00208215274603421,0.00835634582972553,-1.97911603579721

"LDLRAD2",1.256715350525,-0.02681691558,3.24100489865031,0.00209139161391216,0.00838521162149362,-1.98323263243918

"LINC00921",0.69235881095,0.05510580326,3.24070809890317,0.00209319677279632,0.00838916578267823,-1.98403481111654

"FAM227A",0.7477498996875,-0.1245439185,3.23977944902436,0.00209885439675591,0.00840526365925235,-1.98654441819587

"SLC5A8",-0.61152615685,0.08066770352,-3.23881851803772,0.00210472387944216,0.00842382942801541,-1.98914076577979

"AC004832.1",-1.139434536425,-0.00330782584000001,-3.23819196688724,0.00210855926938449,0.00843401040230163,-1.99083337743342

"NDUFA4L2",0.944677255975,-0.03862894122,3.23753975033313,0.00211255877471168,0.00844528861976447,-1.99259509518426

"MYLK4",0.65984390175,0.0152452364,3.23483411102808,0.00212922680378963,0.00850694203510959,-1.99990087391627

"MT1E",-0.7777344541875,0.15262489715,-3.23353664166425,0.00213726378610352,0.00853405972513913,-2.00340288278194

"IFITM5",1.806979929875,0.0170670671,3.23333757411223,0.00213849941060705,0.00853732968131668,-2.00394010575223

"SUSD5",1.103446019325,-0.10812801124,3.23287813326847,0.00214135376165898,0.00854705939389621,-2.00517991432

"CAP2",0.79303985455,0.07388339224,3.23273465690734,0.00214224586824953,0.00854895468420266,-2.00556706387973

"PTPRH",0.723476757475,0.00643705487999999,3.23206265734721,0.00214642889336664,0.00856231214316505,-2.00738020412148

"IZUMO3",-0.753923015025,0.13925976918,-3.23187503078023,0.00214759819623437,0.0085627299590864,-2.00788640019834

"AC124312.4",0.742452731625,-0.1530692077,3.23184706911067,0.00214777250691089,0.0085627299590864,-2.00796183608837

"NWD2",-0.901099104425,0.13391434531,-3.23049484064434,0.00215621813456225,0.00859027859156694,-2.01160940924111

"TNF",1.25526351475,0.4784137848,3.22882306541788,0.00216670287531444,0.00862136502416823,-2.01611755683296

"LINC01560",0.681641650975,0.08659115738,3.22662369886276,0.00218056974213987,0.00867485686931294,-2.02204608020987

"TSNAXIP1",0.70824317055,-0.01447784226,3.22636109601226,0.00218223101995508,0.00867978045754993,-2.02275376409797

"EIF1AY",-1.6516365106875,-0.9947603448,-3.22419791628776,0.00219596121946979,0.00872422987482976,-2.02858183654702

"MMP26",1.01367811765,-0.14340167618,3.22373468249429,0.00219891204492012,0.00873256638424568,-2.02982955349047

"LRRC74A",-0.928509156225,-0.07159327398,-3.22363881120716,0.00219952321736932,0.00873330071268654,-2.03008776737065

"DEFA6",-1.0136400495,-0.2355176451,-3.22350231375515,0.00220039365510749,0.00873506398098086,-2.03045539255613

"NUP210",-1.045747018325,0.13747785534,-3.22109264743901,0.00221581357860984,0.00879457353710932,-2.03694359742504

"PIANP",0.77625828585,-0.13506995232,3.2201524800474,0.00222185748547166,0.00881343972165407,-2.03947420014402

"ABCG1",-0.59482894225,0.0943003732,-3.21981274229927,0.00222404532586245,0.00881870343260497,-2.04038853583617

"OOSP2",-1.16930492755,0.32335920836,-3.21911515826166,0.00222854399203256,0.00883417499641313,-2.04226574323745

"MGAT4C",1.003684387875,0.1901390933,3.21887663375223,0.00223008418610211,0.00883417499641313,-2.04290755412569

"B3GAT1",0.777201920525,0.14968336172,3.21806424024653,0.00223533748662624,0.00884977670067601,-2.04509327014052

"ZNNT1",0.638833649875,-0.0527713317,3.21794966455493,0.00223607932267358,0.00885100331586866,-2.04540150269667

"TAGAP",-0.634304819775,0.03273073318,-3.21748667764941,0.00223907936005041,0.00885603434917236,-2.04664696047065

"EFCAB3",-0.660046046025,-0.11439671502,-3.21717267253088,0.00224111619286889,0.00886068500260103,-2.04749158252753

"AL080317.2",0.921324368075,-0.02579932354,3.21618929276277,0.00224750631603698,0.00888079255602035,-2.05013636010168

"GADD45B",-1.1185162174875,0.30427583236,-3.21592031190406,0.00224925717687149,0.00888428398684079,-2.05085968504567

"CRLF1",1.9194304124,-0.38182237708,3.21499831994023,0.00225526840774669,0.00890459416803125,-2.05333873968419

"CLEC4G",-1.0514642780375,0.17775158807,-3.21451024220258,0.00225845672016955,0.00890998855154524,-2.05465089446759

"AC004477.1",0.696783273325,-0.14991243434,3.21408745864987,0.002261221940268,0.00891607296231923,-2.05578740512146

"IGHG3",-1.744669607,-0.3690138206,-3.21284332131647,0.00226937776269991,0.00893791072706571,-2.05913127480378

"ABCB5",0.676946335358333,-0.0883346435466667,3.21223956090674,0.00227334564335532,0.00895181732482487,-2.06075369420351

"LRRC52-AS1",-0.6320704161,0.10012581052,-3.21064560801584,0.00228385246484276,0.00898628179368129,-2.06503598123873

"AKAP14",0.792078713475,0.14492438058,3.21035117793843,0.00228579825467126,0.00898959800055906,-2.06582683837465

"PGM5",1.003392189775,0.15793152262,3.2098250347815,0.00228927925073647,0.00899899334019355,-2.06723997153508

"FBLN2",0.62939144485,-0.15068263292,3.20910151654481,0.0022940742540458,0.00901440741254271,-2.06918297122445

"HDAC2-AS2",-0.646147176425,0.13689661766,-3.20698657281663,0.00230814505217698,0.0090540447690986,-2.07486096598342

"PDZRN4",0.9720495129625,-0.14689153713,3.20636007357805,0.00231232876686971,0.00906351478393469,-2.07654245445966

"TMEM170B",-0.7923743857625,0.08124071664,-3.20588220745172,0.00231552472483047,0.00907257038866448,-2.07782487388169

"POM121L3P",0.633760227075,0.07526155616,3.20432983296876,0.00232593572440647,0.00910639616312625,-2.0819900107513

"BDKRB2",0.5990007262,-0.02133763604,3.20366999725909,0.00233037425991232,0.0091202880300064,-2.08375999467782

"INSL5",0.7379422204,-0.07684173968,3.20343366746586,0.00233196592677311,0.00912477423753389,-2.08439388153486

"MTLN",1.215237849175,0.45376018404,3.19973472939408,0.00235701194033218,0.00920344208757875,-2.09431118283

"MIR4435-2HG",0.6016860212375,-0.03333998536,3.19923749064172,0.00236039807476053,0.009213152172927,-2.09564376096803

"MACROD2",0.7146720799,0.0672117322533333,3.1987065945876,0.00236401847048908,0.00922552579492957,-2.09706638759

"TAAR3P",-0.68650605045,0.09981344264,-3.19838864477321,0.00236618920374719,0.00923223850816462,-2.09791831342495

"FTL",-0.758304817075,-0.05253265366,-3.19771646703853,0.00237078453476895,0.00924840699701945,-2.09971918498708

"ABL2",-0.7455923123625,0.12406852481,-3.19754562922522,0.00237195380381073,0.00925120684037204,-2.10017684676431

"LYL1",-0.722117728925,0.15698536886,-3.19730972944591,0.00237356927106031,0.00925574557013392,-2.10080877803398
[truncated: 93,247 more chars]
