# Supplementary material for: Identification and development of the novel 7-genes diagnostic signature by integrating multi cohorts based on osteoarthritis
Source: Hereditas. 2022 Jan 29;159:10. doi: 10.1186/s41065-022-00226-z (PMC8801091; doi:10.1186/s41065-022-00226-z)
Supplement: Supplementary file 4 — Additional file 4. Tableshowing the integration and analysis of the differentially expressed genes inthe three datasets, 136 differentially expressed genes were obtained, of which91 were upregulated in the OA group [file 41065_2022_226_MOESM4_ESM.docx]

"","Name","Score"

"THY1","THY1",8.77975321555979e-07

"COL1A1","COL1A1",5.11009610937307e-06

"COL1A2","COL1A2",7.92692049818022e-05

"IL11","IL11",0.000701332459018378

"IGFBP3","IGFBP3",0.000807564064831623

"LRRC15","LRRC15",0.000975083874217741

"CLEC3A","CLEC3A",0.000980560393794905

"COL5A1","COL5A1",0.00110493753525187

"COL3A1","COL3A1",0.00159668712042738

"TNNT3","TNNT3",0.00226469202972246

"CXCL14","CXCL14",0.00226469202972246

"COL6A3","COL6A3",0.00239136130612538

"APCDD1L","APCDD1L",0.00254392390338509

"POSTN","POSTN",0.00285811379405387

"STMN2","STMN2",0.00285811379405387

"CRLF1","CRLF1",0.00492630422625617

"CRTAC1","CRTAC1",0.00560137196342048

"PRSS23","PRSS23",0.00563532966256874

"SPP1","SPP1",0.00563532966256874

"COL2A1","COL2A1",0.00563532966256874

"LGALS1","LGALS1",0.00588275808686001

"RAB31","RAB31",0.00722832046428612

"FNDC1","FNDC1",0.00754478366137701

"TGFBI","TGFBI",0.00836422025588246

"PLXDC1","PLXDC1",0.00870167485606577

"SPON2","SPON2",0.0103001527086125

"CD163","CD163",0.0110553350185223

"S100A4","S100A4",0.0110553350185223

"TNFRSF12A","TNFRSF12A",0.0112412977709306

"COL5A2","COL5A2",0.0116282439436961

"FN1","FN1",0.0122198128911769

"MXRA5","MXRA5",0.0128246779447022

"PENK","PENK",0.013235261885894

"EZR","EZR",0.0136516861474311

"CDK6","CDK6",0.0136516861474311

"FCGR3A","FCGR3A",0.0137145485422716

"OGN","OGN",0.0137145485422716

"SERPINH1","SERPINH1",0.0140739233121769

"EPDR1","EPDR1",0.0145019460763274

"COL8A1","COL8A1",0.0153752397463418

"BASP1","BASP1",0.0163457278172409

"P3H2","P3H2",0.0163457278172409

"EFHD2","EFHD2",0.0174232505039041

"SH3KBP1","SH3KBP1",0.0181311696957444

"COL15A1","COL15A1",0.0183699195236158

"MAP7D1","MAP7D1",0.0188515646187987

"HMCN1","HMCN1",0.0189516597881834

"MATN4","MATN4",0.0189516597881834

"CTHRC1","CTHRC1",0.0205805068495675

"CEMIP","CEMIP",0.0213418514602046

"PTGES","PTGES",0.0215344724251496

"ASPN","ASPN",0.0215344724251496

"CCND1","CCND1",0.0236981165297842

"LOXL1","LOXL1",0.0240958572680764

"MATN3","MATN3",0.0240958572680764

"CX3CR1","CX3CR1",0.0240958572680764

"HAPLN1","HAPLN1",0.0261609307420881

"NOP10","NOP10",0.026441054476552

"THBS2","THBS2",0.0266371995136541

"SMOC2","SMOC2",0.0266371995136541

"EMX2OS","EMX2OS",0.0266371995136541

"BFSP1","BFSP1",0.0267224623478823

"ARL4C","ARL4C",0.0291596597620907

"SLC36A2","SLC36A2",0.0291596597620907

"NKX3-2","NKX3-2",0.0296064906557115

"HLA-DRA","HLA-DRA",0.0316642282769587

"TNC","TNC",0.0316642282769587

"CNTNAP2","CNTNAP2",0.0316642282769587

"C3AR1","C3AR1",0.0341517625817926

"IGFBP7","IGFBP7",0.0341517625817926

"PNMA8A","PNMA8A",0.0341517625817926

"HLA-DRB1","HLA-DRB1",0.0366230144302204

"FLRT3","FLRT3",0.0366230144302204

"LOXL3","LOXL3",0.0390786497148075

"RSPO2","RSPO2",0.0390786497148075

"OSTC","OSTC",0.0393283069026302

"LPAR4","LPAR4",0.0406656844028855

"MEX3D","MEX3D",0.0415192635208684

"SYT11","SYT11",0.0415192635208684

"OPCML","OPCML",0.0415192635208684

"PPP1R14C","PPP1R14C",0.0420214177664483

"DKK3","DKK3",0.04236319779236

"TWIST1","TWIST1",0.0439453917446654

"NOX5","NOX5",0.0439453917446654

"NID2","NID2",0.0463575202198506

"IGFBP4","IGFBP4",0.0463575202198506

"SLC8A3","SLC8A3",0.0463575202198506

"U82695.1","U82695.1",0.0472657976620229

"MMP2","MMP2",0.0487560919977694

"CA9","CA9",0.0487560919977694

"GLI1","GLI1",0.0487560919977694
