# Supplementary material for: Identification and development of the novel 7-genes diagnostic signature by integrating multi cohorts based on osteoarthritis
Source: Hereditas. 2022 Jan 29;159:10. doi: 10.1186/s41065-022-00226-z (PMC8801091; doi:10.1186/s41065-022-00226-z)
Supplement: Supplementary file 5 — Additional file 5. Tableshowing that in the three datasets, 136 differentially expressed genes wereobtained, of which 45 were downregulated in the normal group [file 41065_2022_226_MOESM5_ESM.docx]

"","Name","Score"

"SOD3","SOD3",0.000783142980596452

"GDF10","GDF10",0.00426140126868924

"APOD","APOD",0.00426140126868924

"MPO","MPO",0.00426140126868924

"GBP2","GBP2",0.00462047875591176

"CCDC85A","CCDC85A",0.00837909564965023

"BTG2","BTG2",0.00837909564965023

"DEFA4","DEFA4",0.00837909564965023

"ALDH1L1","ALDH1L1",0.00969779196416898

"CFH","CFH",0.011964086320868

"LVRN","LVRN",0.0124105586585297

"S100A12","S100A12",0.0124105586585297

"ADM","ADM",0.0134896642527463

"IL17RB","IL17RB",0.0163744828754764

"LINC01554","LINC01554",0.0163744828754764

"AZU1","AZU1",0.0163744828754764

"BEX5","BEX5",0.0202812993375804

"BEX2","BEX2",0.0202812993375804

"S100A9","S100A9",0.0202812993375804

"GPX3","GPX3",0.0215439222803033

"GADD45A","GADD45A",0.0235115467642237

"SERPINA6","SERPINA6",0.0241378855386398

"DEPP1","DEPP1",0.0241378855386398

"PRTN3","PRTN3",0.0241378855386398

"BCL6","BCL6",0.0255535585153325

"TCEAL2","TCEAL2",0.0279492049609256

"MEG3","MEG3",0.0279492049609256

"LCN2","LCN2",0.0279492049609256

"SLC25A37","SLC25A37",0.0298554410949808

"TCEAL6","TCEAL6",0.0317190521260413

"CHRNA5","CHRNA5",0.0317190521260413

"S100A8","S100A8",0.0317190521260413

"PDE3B","PDE3B",0.0354504467060908

"LMOD3","LMOD3",0.0354504467060908

"ELANE","ELANE",0.0354504467060908

"USP53","USP53",0.0391458640681056

"TRIB1","TRIB1",0.0391458640681056

"S100P","S100P",0.0391458640681056

"MAP2K6","MAP2K6",0.0428073802429796

"CEACAM8","CEACAM8",0.0428073802429796

"DDIT3","DDIT3",0.0464367682061758

"RNASE2","RNASE2",0.0464367682061758

"ADHFE1","ADHFE1",0.0470652914138219

"SLC25A27","SLC25A27",0.0481441452815399

"MAATS1","MAATS1",0.0486872587537808
